# Current Inequities in Smoking Prevalence on District Level in Iran: A Systematic Analysis on the STEPS Survey

**DOI:** 10.34172/jrhs.2022.75

**Published:** 2021-12-28

**Authors:** Mohsen Abbasi-Kangevari, Masoud Masinaei, Nima Fattahi, Yekta Rahimi, Negar Rezaei, Sina Azadnajafabad, Ali Ghanbari, Roozbe Fakhimi, Zahra Jourahmad, Seyyed-Hadi Ghamari, Mohammad-Reza Malekpour, Naser Ahmadi, AmirAli Hajebi, Hamed Jafar-pour, Farshad Farzadfar

**Affiliations:** ^1^Non-Communicable Diseases Research Center, Endocrinology and Metabolism Population Sciences Institute, Tehran University of Medical Sciences, Tehran, Iran; ^2^Social Determinants of Health Research Center, Shahid Beheshti University of Medical Sciences, Tehran, Iran; ^3^Department of Epidemiology and Biostatistics, School of Public Health, Tehran University of Medical Sciences, Tehran, Iran; ^4^Endocrinology and Metabolism Research Center, Endocrinology and Metabolism Clinical Sciences Institute, Tehran University of Medical Sciences, Tehran, Iran; ^5^Student Research Committee, Kurdistan University of Medical Sciences, Sanandaj, Iran; ^6^Isfahan Neuroscience Research Center, Isfahan University of Medical Sciences, Isfahan, Iran; ^7^Medical Student, Student Research Committee, Faculty of Medicine, Mazandaran University of Medical Sciences, Sari, Iran

**Keywords:** Epidemiology, Public health, Smoking, Social determinants of health, Tobacco smoke pollution

## Abstract

**Background:** The prevalence of tobacco smoking and its burden on societies is not homogenous at the national and district levels. This nationwide study aimed to investigate current inequalities in the prevalence of smoking at the district level and the association of smoking behaviors with gender, wealth, education, and urbanization in Iran.

**Study design:** A cross-sectional study.

**Methods:** This study was conducted by analyzing the data of the STEPS survey 2016 with 30541 participants. The small-area estimation method using the Bayesian spatial hierarchical multilevel regression model was employed to generate district-level prevalence of all types of smoking by gender. The inequalities between the groups by wealth, education, and urbanization were investigated via concentration index.

**Results:** The prevalence rates of current daily cigarette smoking were found to be at the range of 4.6-40.9 and 0-4.5 among men and women, respectively. Current daily cigarette smoking was higher in men than in women: 19.0 (95% CI: 9.5-28.7) vs 0.7 (95% CI: 0-6.9). Women with lower wealth, education, or urbanization were more likely to smoke tobacco or be exposed to secondhand smoking. On the other hand, men with higher wealth or education indices were more likely to smoke tobacco. Men with lower wealth, education, or urbanization were more likely to be exposed to secondhand smoking.

**Conclusion:** The smoking behavior varied significantly at the district level in Iran. Gender, wealth, education, and urbanization were determinants of smoking prevalence.

## Background

 Tobacco use and exposure to tobacco smoke are major risk factors for mortality and disability-adjusted life-years (DALYs). Smoking has globally accounted for 13% of all deaths and more than 7% of all DALYs in 2017. Furthermore, more than 25% of all cancer deaths have been attributable to smoking.^1^ The prevalence of tobacco smoking has annually decreased by 1.6% globally^2^ and has stagnated in Iran in recent years,^3^ which is despite its adverse effects, far less than the 5.8% goal set by the World Health Organization.^4^ If the current trends persist, it is expected that the total number of annual deaths will increase and reach 10 million by 2030.^5^ Therefore, the 2030 Agenda for Sustainable Development Goals has called for a joint action to reduce the current prevalence of smoking.^6^

 The prevalence of tobacco smoking and its burden on societies are not homogenous at the national and district levels and could be affected by social determinants. Tobacco smoking imposes the most significant mortality and morbidity in low- and middle-income countries.^7^ While the prevalence of smoking among women is closer to men in high-income countries, there can be a wide gender gap in low- and middle-income countries in this regard.^8^ On the individual level, there is evidence that people’s smoking behavior in urban areas differs from that in rural areas.^9^ Education is reported to be a determinant of smoking behavior in societies.^10^ Nevertheless, the current inequities and the determinants of smoking prevalence on the district level of Iran have not been yet investigated. Therefore, defining the social determinants of tobacco smoking in Iran is an early step towards recognizing the most vulnerable groups and taking the necessary nationwide measures for proper resource allocations to address the issue. This national study aimed to investigate the prevalence of smoking at the district level, the existence of current inequities, and the association of smoking behaviors with gender, wealth, education, and urbanization in Iran.

## Methods

###  Overview

 This study was conducted by analyzing the data of STEPS survey 2016 with 30 541 participants. The small-area estimation method using the Bayesian spatial hierarchical multilevel regression model was employed to generate district-level prevalence of all types of smoking by gender. The inequalities between the groups by wealth, education, and urbanization were investigated via Blinder-Oaxaca decomposition and concentration index.

###  Data sources

 This study was a systematic analysis of the data of STEPS survey 2016, which was a cross-sectional national study carried out by the Non-Communicable Diseases Research Center (NCDRC), Endocrinology and Metabolism Research Institute, Tehran University of Medical Sciences, Tehran, Iran. The participants of the STEPS survey were selected via multistage cluster sampling and were representative of the general population aged ≥ 18 years living in all provinces of Iran. A detailed description of the study population and the sampling method of the 2016 version of the STEPS survey has been published.^11,12^

###  Variables

 Variables included sociodemographic characteristics, including gender, education, wealth, governmental employment, and complimentary insurance; and smoking, including current daily cigarette smoking (CDS), current tobacco smoking, ever cigarette smoking, ever tobacco smoking, exposure to secondhand smoking, exposure to secondhand smoking at home, and past daily cigarette smoking. CDS was defined as smoking any number of cigarettes daily. Current tobacco smoking was defined as the use of any smoked tobacco products, including cigarettes, cigars, pipes, or any other smoked tobacco products on a daily, non-daily, or occasional basis. The main tobacco-containing products used by Iranians are cigarettes and water-pipe while chewing tobacco and other forms are uncommon.^3^ Ever cigarette smoking was defined as the ever use of any number of cigarettes. Ever tobacco smoking was defined as the ever use of any smoked tobacco products, including cigarettes, cigars, pipes, or other smoked tobacco products. Exposure to secondhand smoking was defined as being exposed to the smoke of any tobacco products in the last 30 days. Exposure to secondhand smoking at home was defined as being exposed to the smoke of any tobacco products at home in the last 30 days. Past daily cigarette smoking was defined as the ex-use of any smoked tobacco products, including cigarettes, cigars, pipes, or any other smoked tobacco products daily.

###  Data analysis

 The small-area estimation method using Bayesian spatial hierarchical multilevel regression was utilized to generate district-level prevalence of all types of smoking by gender. To study the inequalities between the groups, Blinder-Oaxaca decomposition was used for linear regression models.^13,14^ This method is based on two regression models, fitted separately for the two population groups^15^ (in this study, the first and fifth quintiles of wealth, education, and urbanization indices). To calculate the wealth index, principal component analysis was applied to derive household wealth index based on questions on key dwelling characteristics and household ownership, as described in the protocol study of STEPS 2016.^12^ The principal component analysis is an approach to statistical analysis in which multiple datasets are combined as orthogonal components.^16^ The wealth index was used to divide the population into quintiles, whereby the first and fifth quintiles presented the least fortunate and wealthiest households, respectively. The education index was defined as the mean of successful years of schooling, calculated based on the Household Income and Expenditure Survey conducted by Statistical Center of Iran, Tehran, Iran, in 2016.^17^ The education index was used to divide the population into quintiles, in which the first and fifth quintiles presented the least and most educated, respectively. The urbanization index was defined as the proportion of residents in the urban area to the urban and rural areas. It was used to divide the population into quintiles, according to which the first and fifth quintiles present the least and most urbanized, respectively. The concentration index was used to quantify the degree of inequality in all types of smoking-related to wealth, education, and urbanization. A concentration index of zero would indicate no inequality related to wealth, education, and urbanization. Negative values of concentration index would mean a higher prevalence of smoking among people with lower wealth, education, or urbanization index. Positive concentration index values would indicate a higher prevalence of the type of smoking among people with higher wealth, education, or urbanization index.^18^

 The results were reported by terms of mean and prevalence, with their corresponding 95% uncertainty intervals (95% UI). Population derived from the National Population and Housing Census conducted by the Statistical Center of Iran in 2016 was considered the standard population in the direct age-standardized approach to allow comparisons between districts. In addition, in this study, the inter-district variation of smoking markers was assessed to check homogeneity across provinces. The two-sample *t* test was also used to check the significant differences in the prevalence of smoking determinants between the least fortunate and the wealthiest, the least educated and the most educated, and the least urbanized and the most urbanized groups. The *P* values of less than 0.05 were considered significant. The statistical analyses and data visualizations were carried out using Stata (version 16.0) and R statistical packages (version 3.4.3; http://www.r-project.org, RRID: SCR_001905).

## Results

 Responses of 30 541 participants were analyzed. The mean age of the subjects was estimated at 44.5 ± 16.3 years, which was 44.1 ± 16.0 years in women and 44.9 ± 16.5 years in men.

###  Age-standardized smoking prevalence by district

####  Current daily cigarette smoke

 In 196 of the 429 districts, the age-standardized prevalence (95% UI) of CDS was higher in men than in the national spectrum. The three districts with the highest age-standardized prevalence (95% UI) of CDS among men were Rabar in Kerman province: 40.9% (23.6-54.7), Abbas-Abad in Mazandaran province: 40.3% (26.3-51.8), and Pasargad in Fars province: 35.3% (21.8-49.7), Iran. The three districts with the lowest age-standardized prevalence (95% UI) of CDS among men were Dargaz in Razavi Khorasan province: 4.6% (0-17.5), Fariman in Razavi Khorasan province: 4.6% (0-16.6), and Eyvan in Ilam province: 4.7% (0-16.5), Iran. Provinces were sorted by the age-standardized prevalence of smoking among districts. Among 31 provinces in Iran, Alborz, Ardebil, West Azarbayjan, Golestan, North Khorasan, Markazi, Qazvin, and Qom had districts with the same level of age-standardized prevalence and thus had lower heterogeneity. Provinces with the most significant variability of the district-level age-standardized prevalence of CDS included Kerman (5.2-40.9%), Mazandaran (7.3-40.3%), and Fars (8.6-35.2%). Provinces that showed the lowest variability of the district-level age-standardized prevalence of CDS included Golestan (7.9-11.0%), Qazvin (22.3-26.6%), and Boushehr (11.6-16.1%).

 Among 429 districts, the age-standardized prevalence (95% UI) of CDS among 132 districts among women was higher than the national age-standardized prevalence. The three districts with the highest age-standardized prevalence (95% UI) of CDS among women were Dashti in Boushehr province: 4.5% (0-14.8), Saravan in Sistan and Balouchestan Province: 4.1% (0-19.7), and Kangan in Boushehr Province: 3.7% (0-14.7), Iran. The three districts with the lowest age-standardized prevalence (95% UI) of CDS among women were Razan in Hamedan province: 0% (0-4.9), Famenin in Hamedan province: 0% (0-7.1), and Dalfan in Lorestan province: 0.04% (0-1.3), Iran. Among 31 provinces, Boushehr, Qom, and Sistan and Balouchestan were the only provinces with districts with the same level of age-standardized prevalence and thus had lower heterogeneity. Provinces that showed the most significant variability of the district-level age-standardized prevalence of CDS included Boushehr, where estimates ranged from 1.3-4.5%, Sistan and Balouchestan 1.1-4.1%, and Markazi 0.5-3.2%. Provinces that showed the lowest variability of the district-level age-standardized prevalence of CDS included Alborz (0.2-0.7%), Lorestan (0.04-0.5%), and Chaharmahal (0.1-0.6%), Iran.

**Figure 1:**
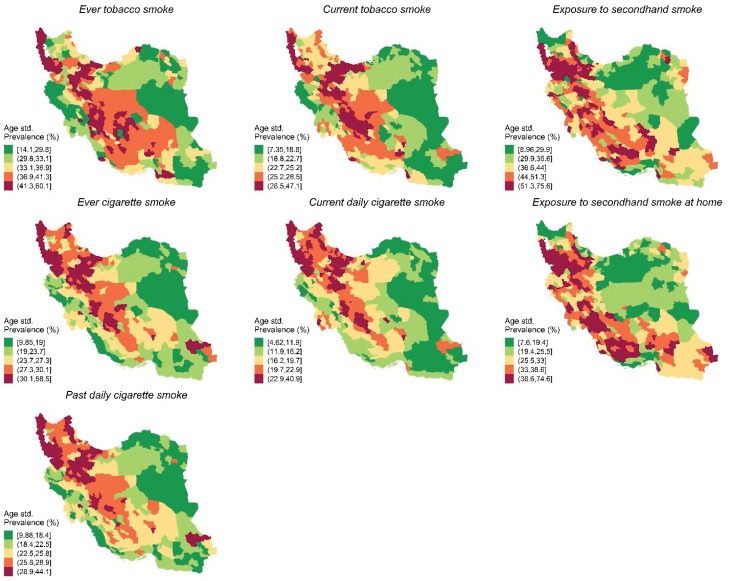
Geographical distribution of tobacco smoking among men on district level in Iran in 2016.

**Figure 2:**
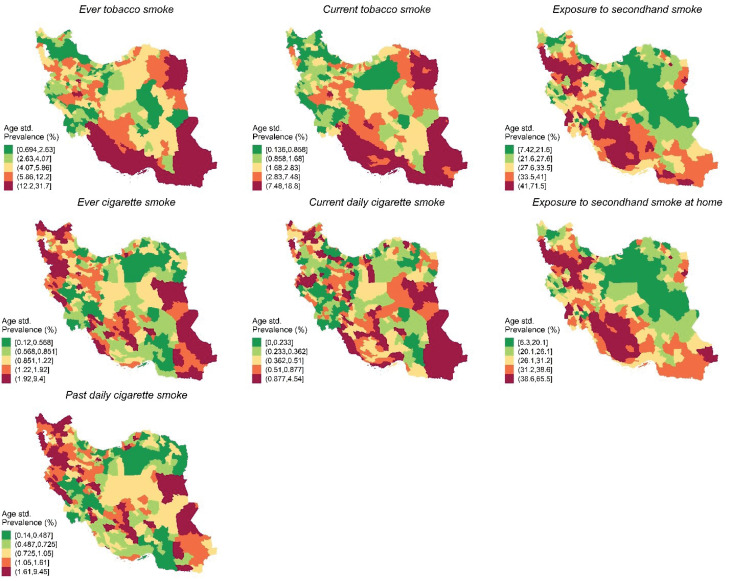
Geographical distribution of tobacco smoking among women on district level in Iran in 2016.

 The age-standardized prevalence of current tobacco smoke, ever cigarette smoke, ever tobacco smoke, secondhand smoking exposure, secondhand smoking at home, and past daily cigarette smoke varied significantly on a district level (Figures 1 and 2, Supplementary Files 1 and 2).

###  All-ages smoking prevalence by gender

 The prevalence of smoking among all age groups and both genders is presented in Table 1. Men had a higher prevalence of current daily cigarette smoke, current tobacco smoke, ever cigarette smoke, exposure to secondhand smoking, exposure to secondhand smoking at home, and past daily cigarette smoke. The prevalence of all types of smoking was significantly higher among men, except for exposure to secondhand smoking at home.

**Table 1:** All-ages prevalence of smoking by gender

**Type of smoking by gender**	**All ages prevalence (95% UI)**	***P*** ** value**
Current daily cigarette smoke		0.001
Female	0.7 (0-6.9)	
Male	19.0 (9.5-28.7)	
Total	9.8 (4.8-17.8)	
Current tobacco smoke		0.001
Female	4.0 (0.8-10.1)	
Male	24.5 (13.5-35.8)	
Total	14.3 (7.1-22.9)	
Ever cigarette smoke		0.001
Female	1.3 (0-6.3)	
Male	27.7 (16.5-39.1)	
Total	14.5 (8.2-22.7)	
Ever tobacco smoke		0.001
Female	7.3 (2.1-15.2)	
Male	37.1 (22.4-51.9)	
Both	22.2 (12.2-33.5)	
Exposure to secondhand smoking		0.001
Female	31.2 (12.8-50.4)	
Male	39.5 (18.7-60.7)	
Total	35.4 (15.8-55.6)	
Exposure to secondhand smoking at home		0.720
Female	29.8 (11.8-48.8)	
Male	29.3 (11.1-48.5)	
Total	29.6 (11.5-48.6)	
Past daily cigarette smoke		0.001
Female	1.1 (0-6.7)	
Male	26.5 (15.4-37.7)	
Total	13.8 (7.7-22.2)	

###  All-ages smoking prevalence by wealth index

 The prevalence (95% UI) of current daily cigarette smoke ranged from 7.6% (2.2-16.4) among people with the lowest wealth index to 11.7% (6.9-19.1) among those with the highest wealth index. The prevalence (95% UI) of ever cigarette smoke ranged from 11.9% (5.2-21.3) among people with the lowest wealth index to 16.2% (10.3-23.7) among those with the highest wealth index. The prevalence (95% UI) of past daily cigarette smoke ranged from 11.0% (4.7-20.6) among people with the lowest wealth index to 15.5% (9.7-23.1) among those with the highest wealth index. In contrast, the prevalence (95% UI) of exposure to secondhand smoking at home ranged from 26.9% (10.2-44.6) among people with the highest wealth index to 30.6% (11.5-50.5) among those with the lowest wealth index. There were no significant differences between the prevalence of current tobacco smoke, ever tobacco smoke, exposure to secondhand smoking, and wealth index groups. The prevalence of smoking by wealth index is presented in Table 2.

**Table 2:** Prevalence of smoking among groups by wealth, education, and urbanization indices

**Wealth index**	**All-ages prevalence (95% UI)**	***P*** ** value**	**Education** **index**	**All-ages prevalence (95% UI)**	***P*** ** value**	**Urbanization** **index**	**All-ages prevalence (95% UI)**	***P*** ** value**
Current daily cigarette smoke		0.001			0.910			0.001
1	7.6 (2.2-16.4)		1	9.6 (4.2-17.9)		1	8.5 (3.1-17.1)	
2	9.4 (4.3-17.1)		2	10.0 (4.9-18.0)		2	10.1 (4.9-18.2)	
3	9.5 (4.7-17.1)		3	10.0 (5.0-17.9)		3	9.9 (4.8-17.8)	
4	11.0 (5.8-19.1)		4	9.9 (5.0-17.8)		4	10.1 (5.1-18.1)	
5^a^	11.7 (6.9-19.1)		5^b^	9.7 (4.8-17.3)		5^c^	10.5 (5.8-17.6)	
Current tobacco smoke		0.590			0.520			0.270
1	14.3 (5.7-23.9)		1	13.8 (6.1-23.1)		1	13.6 (5.5-23.1)	
2	13.4 (6.4-21.8)		2	13.9 (6.8-22.6)		2	14.8 (7.4-23.7)	
3	13.7 (6.8-22.1)		3	14.7 (7.5-23.5)		3	14.4 (7.3-23.2)	
4	15.0 (8.1-23.9)		4	14.9 (7.8-23.2)		4	14.1 (7.3-22.5)	
5	15.0 (8.6-23.0)		5	14.2 (7.5-22.3)		5	14.5 (8.1-22.2)	
Ever cigarette smoke		0.001			0.580			0.090
1	11.9 (5.2-21.3)		1	14.4 (7.6-23.3)		1	12.9 (6.2-22.0)	
2	14.4 (8.0-22.4)		2	14.8 (8.5-22.7)		2	14.9 (8.6-23.2)	
3	14.4 (8.3-22.1)		3	14.6 (8.5-22.5)		3	15.1 (8.7-23.5)	
4	15.8 (9.4-23.9)		4	14.6 (8.5-22.8)		4	14.8 (8.6- 22.8)	
5	16.2 (10.3-23.7)		5	14.2 (8.1-22.2)		5	14.9 (9.1-22.0)	
Ever tobacco smoke		0.500			0.830			0.760
1	22.4 (11.0-34.6)		1	21.9 (11.2-33.9)		1	21.7 (10.6-34.2)	
2	21.8 (11.8-33.1)		2	21.8 (12.0-33.2)		2	22.9 (12.9-34.4)	
3	22.1 (12.2-33.0)		3	22.6 (12.6-33.9)		3	23.1 (12.9-34.6)	
4	22.8 (13.0-34.4)		4	22.9 (12.9-34.1)		4	21.5 (11.9-32.7)	
5	21.9 (12.9 - 32.6)		5	21.7 (12.2-32.6)		5	21.7 (12.6-31.8)	
Exposure to secondhand smoking		0.050			0.001			0.001
1	35.7 (15.2-56.7)		1	35.6 (15.4-56.6)		1	35.8 (14.3-58.0)	
2	38.6 (18.6-59.2)		2	36.2 (16.2-56.8)		2	37.0 (17.4-57.3)	
3	34.0 (15.1-53.4)		3	37.3 (18.0-57.1)		3	36.0 (16.0-56.5)	
4	35.0 (14.9-55.9)		4	37.5 (18.0-57.4)		4	35.3 (16.2-55.0)	
5	33.6 (15.1-52.6)		5	30.1 (11.2-49.8)		5	32.7 (15.0-50.9)	
Exposure to secondhand smoking at home		0.001			0.001			0.001
1	30.6 (11.5-50.5)		1	30.7 (11.8-50.7)		1	30.6 (10.6-51.9)	
2	33.6 (14.6-53.5)		2	30.5 (11.7-50.1)		2	31.3 (13.0-50.6)	
3	28.3 (10.9-46.5)		3	31.2 (13.2-49.9)		3	30.5 (12.1-49.9)	
4	28.4 (10.2-48.0)		4	31.6 (13.3-50.4)		4	29.3 (11.7-47.8)	
5	26.9 (10.2-44.6)		5	23.9 (7.2-42.0)		5	26.1 (10.0-42.9)	
Past daily cigarette smoke		0.001			0.520			0.120
1	11.0 (4.7-20.6)		1	13.6 (7.1-22.9)		1	12.3 (5.8-21.6)	
2	13.7 (7.5-22.1)		2	14.2 (8.1-22.4)		2	14.2 (8.1-22.8)	
3	13.7 (7.8-21.6)		3	14.0 (7.9-22.2)		3	14.3 (8.1-22.9)	
4	15.1 (8.8-23.5)		4	13.8 (8.0-22.0)		4	14.0 (8.1-22.1)	
5	15.5 (9.7-23.1)		5	13.3 (7.5-21.4)		5	14.1 (8.4-21.4)	

^a^Wealthiest. ^b^Most educated. ^c^Most urbanized.

 The investigation of the gap in women’s exposure to secondhand smoking between the first and fifth quintiles of the wealth index showed that having complementary insurance significantly contributed to the gap between the wealthiest and least fortunate (Supplementary File 3).

###  All-ages smoking prevalence by education index

 The prevalence (95% UI) of exposure to secondhand smoking ranged from 30.1% (11.2- 49.8) among people with the highest education index to 35.6% (15.4-56.6) among those with the lowest education index. The prevalence (95% UI) of exposure to secondhand smoking at home ranged from 23.9% (7.2-42.0) among people with the highest education index to 30.7% (11.8-50.7) among those with the lowest education index. The prevalence rates of current daily cigarette smoke, current tobacco smoke, ever cigarette smoke, ever tobacco smoke, exposure to secondhand smoking, and past daily cigarette smoke were not significantly different among various education index groups (Table 2).

 The study of the gap in the exposure to secondhand smoking between the first and fifth quintiles of education index among both genders revealed that having complementary insurance made a significant contribution to the gap between the most and the least educated (Supplementary File 4).

###  All-ages smoking prevalence by urbanization index

 The prevalence (95% UI) of current daily cigarette smoke ranged from 8.5% (3.1-17.1) among people with the lowest urbanization index to 10.5% (5.8-17.6) among those with the highest urbanization index. In contrast, the prevalence (95% UI) of exposure to secondhand smoking ranged from 32.7% (15.0-50.9) among people with the highest urbanization index to 35.8% (14.3-58.0) among those with the lowest urbanization index. The prevalence (95% UI) of exposure to secondhand smoking at home ranged from 26.1% (10.0-42.9) among people with the highest urbanization index to 30.6% (10.6-51.9) among those with the lowest urbanization index. No differences were found between urbanization index groups in current tobacco smoke, ever cigarette smoke, ever tobacco smoke, exposure to secondhand smoking, exposure to secondhand smoking at home, and past daily cigarette smoke (Table 2).

 The examination of the gap in the prevalence of CDS between the first and fifth quintiles of the urbanization index among both genders showed that the wealth index made a significant contribution to the gap between the most and the least urbanized (Supplementary File 5).

###  Determinants of all types of smoking among women and men

 Wealth, education, and urbanization played varying roles in the prevalence of all types of smoking among men and women. The lower wealth index or education index was associated with higher smoking or exposure to secondhand smoking among women. While men with higher wealth or education indexes were more likely to smoke, lower wealth and education indexes were associated with higher exposure to secondhand smoking. The urbanization index had a paradoxical effect on current tobacco smoking among women and men; regarding, women with a lower urbanization index and men with higher urbanization index were more likely to smoke tobacco (Table 3).

**Table 3:** Concentration index for wealth, education, and urbanization for all types of smoking among women and men

**Type of smoking by gender**	**Concentration index**		
	**Wealth index**	**Education index**	**Urbanization index**
Current daily cigarette smoke			
Female	-0.12 (-0.16, -0.08)	-0.03 (-0.08, 0.01)	0.01 (-0.03, 0.05)
Male	0.07 (0.06, 0.08)	0.11 (0.09, 0.12)	0.04 (0.03, 0.05)
Total	0.07 (0.04, 0.09)	0.04 (0.01, 0.07)	0.04 (0.01, 0.07)
Current tobacco smoke			
Female	-0.20 (-0.24, -0.17)	-0.13 (-0.18, -0.09)	-0.09 (-0.13, -0.05)
Male	0.05 (0.04, 0.06)	0.53 (0.53, 0.54)	0.03 (0.02, 0.03)
Total	0.01 (-0.01, 0.03)	0.20 (0.18, 0.22)	0.01 (-0.01, 0.03)
Ever cigarette smoke			
Female	-0.06 (-0.09, -0.02)	-0.15 (-0.18, -0.11)	0.002 (-0.03, 0.04)
Male	0.05 (0.04, 0.06)	-0.04 (-0.05, -0.03)	0.02 (0.01, 0.03)
Total	0.04 (0.01, 0.07)	-0.09 (-0.12, -0.07)	0.02 (-0.003, 0.05)
Ever tobacco smoke			
Female	-0.17 (-0.2, -0.14)	-0.40 (-0.44, -0.37)	-0.09 (-0.12, -0.06)
Male	0.03 (0.02, 0.04)	0.46 (0.45, 0.47)	0.01 (0.004, 0.02)
Total	-0.003 (-0.02, 0.02)	0.03 (0.01, 0.05)	-0.01 (-0.03, 0.01)
Exposure to secondhand smoking			
Female	-0.03 (-0.04, -0.02)	-0.97 (-0.98, -0.96)	-0.03 (-0.04, -0.01)
Male	-0.01 (-0.02, 0.01)	-0.63 (-0.64, -0.62)	-0.001 (-0.01, 0.01)
Total	-0.02 (-0.03, -0.01)	-0.80 (-0.81, -0.79)	-0.01 (-0.02, -0.003)
Exposure to secondhand smoking at home			
Female	-0.03 (-0.05, -0.02)	-1.18 (-1.19, -1.16)	-0.03 (-0.04, -0.01)
Male	-0.04 (-0.05, -0.02)	-0.84 (-0.86, -0.83)	-0.02 (-0.03, -0.01)
Total	-0.04 (-0.05, -0.03)	-1.01 (-1.02, -1.00)	-0.02 (-0.03, -0.01)
Past daily cigarette smoke			
Female	0.01 (-0.02, 0.04)	-0.12 (-0.15, -0.09)	0.03 (0.0, 0.07)
Male	0.05 (0.04, 0.05)	-0.14 (-0.15, -0.13)	0.02 (0.01, 0.03)
Total	0.04 (0.02, 0.07)	-0.13 (-0.15, -0.10)	0.02 (-0.01, 0.05)

## Discussion

 The results of the study showed that gender, wealth, education, and urbanization were all determinants of smoking prevalence at the district level in Iran. Wealth, education, and urbanization had varying effects on the prevalence of all types of smoking among men and women. Accordingly, women with lower wealth, education, or urbanization were more likely to smoke tobacco or be exposed to secondhand smoking. On the other hand, men with higher wealth or education index were more likely to smoke tobacco. Nevertheless, men with lower wealth, education, or urbanization were more likely to be exposed to secondhand smoking.

 While estimates on current tobacco smoke on the province level had a 3-fold range (from 9% to 27%),^19^ districts showed much greater variability, particularly at the higher end, with a 9-fold range of estimates (from 4.6% to 40.9%). Although these findings could shed light on the areas with the most significant burden, they highlight the necessity of identifying the underlying causes for such heterogeneity. The witnessed heterogeneities need to be addressed while implementing the tobacco control policies in the future.^20^ The lower prevalence of cigarette smoking in some states has been associated with tobacco control measures in the United States, where the policies are determined by the state government commensurate with the states’ current needs.^21^ However, such conditions do not apply to Iran, where the government generally makes the policies in Tehran. Therefore, more research is needed to investigate such heterogeneities in Iran to pave the way for effective policies to bridge the existing gaps.

 The higher prevalence of smoking among men is similar to reports from high-, low-, and middle-income countries.^22–24^ Despite the accomplishments of the past three decades worldwide,^25^ the reductions in smoking prevalence were smaller in women in the last decade than in men.^23^ This could be particularly problematic, with the emerging evidence that women are more vulnerable to the adverse effects of smoking.^26^ Furthermore, Iranian women had higher exposure to secondhand smoking at home. Secondhand smoking remains to take its toll on the health of non-pregnant and pregnant women.^27–29^ Consequently, more effective interventions and actions are required to address active and passive smoking among women.

 On the national level, the findings of some studies have indicated that daily smoking is more common in high-income countries than in low- and middle-income countries.^22^ However, on the district level, a meta-analysis found an association between higher prevalence of cigarette smoking and lower-income levels.^30^ In this study, men with higher and women with lower wealth were more likely to smoke. The less fortunate in both genders were more prone to exposure to secondhand smoking. While taxation is considered among successful strategies to reduce inequities in cigarette smoking to the benefit of the poor,^31^ the existing inequalities among the target population need to be taken into account prior to implementing interventions. It is also worth mentioning that such strategies could lose color in a country where people with a higher wealth index tend to smoke more than those with a lower wealth index. Therefore, future research needs to investigate the responsiveness of various economic groups of society to price variations aimed to discourage people from smoking. Moreover, the marketing and branding of tobacco companies, alongside the public health measures, need closer evaluation.^32^

 Lower education was associated with higher smoking among women and higher exposure to secondhand smoking among both women and men. The results of some studies have reported the inverse association between the level of education and passive smoking.^33,34^ Nevertheless, there is still controversy in the existing literature considering education as a determinant of smoking behavior. Although passive smoking has been reported not to be significantly associated with education,^35^ the results of some studies have indicated an association between lower educational levels and increased risk of smoking.^36–38^ This could probably be explained by the previous reports suggesting that low education is associated with low health literacy.^39^ Higher education is considered a strong determinant of smoking avoidance^10,40^; however, some evidence suggests a bi-directional relationship between higher education enrollment and smoking.^37^ Therefore, the association between education and smoking needs further investigation.

 While women with a lower urbanization index were more likely to smoke tobacco, men with higher urbanization index were more likely to smoke tobacco. A review of the Global Adult Tobacco Survey results reported that rural residence was associated with a higher risk of tobacco smoking.^24^ The findings of another study reported that rural Americans were more likely to smoke or be exposed to cigarette smoke than their urban or suburban counterparts.^41^ Similarly, the results of a study conducted in China reported that people residing in urban areas were less likely to smoke passively than in rural areas.^35^ The pattern of cigarette smoking seems to be strongly determined by the domestic factors of each society. Environmental advertisements, marketing strategies, peer pressure, and pricing strategies vary among rural and urban areas.

 This was the first nationwide study to report the prevalence of all types of smoking at the district level in Iran. A better understanding of the patterns of geographic variation empowers public health authorities and policymakers to target areas with a known higher prevalence. The STEPS data 2016 used in this study were cross-sectional. Consequently, the causal inferences from the results must be interpreted with caution. However, the current study design was consistent with the primary objective of this study, which was to investigate the variability in smoking prevalence across socioeconomic and geographic dimensions. It is worth mentioning that depending on the statistical tool employed for each analysis, there could be slight variations in the reported prevalence among study groups. The main tobacco-containing product used by Iranians is the cigarette. Therefore, this study focused only on cigarette smoking, and other forms of tobacco products, such as water-pipe or chewing tobacco, were excluded. Moreover, due to the complex nature of smoking, not all possible determinants ^42^ could be included in this study.

## Conclusions

 There were significant heterogeneities among the prevalence of all types of smoking on the district level in Iran. It was revealed that the smoking behavior varied significantly on the district level. Gender, wealth, education, and urbanization were determinants of smoking prevalence. It could be suggested that adopting legislation and control laws based on district prevalence and social determinants of smoking could minimize tobacco consumption.

## Acknowledgments

 The authors would like to thank all the participants, researchers, and staff of medical universities and the Ministry of Health and Medical Education of Iran, which helped conduct the Iran STEPS 2016 survey. Also, we appreciate the aid of all colleagues in the Non-Communicable Diseases Research Center (NCDRC), and Endocrinology and Metabolism Research Institute, Tehran University of Medical Sciences.

## Authors’ Contribution

 Conceptualization, F.F., N.R., M.A-K.; Data collection, M.M., N.F., Y.R., R.F., Z.J. H.J., A-A.H.; Data analysis, M.M., A.G., M-R.M., N.A.; Data visualization, M.M., M-R.M.; Writing, Original Draft, M.A-K., N.F., M-R.M., S-H.G.; Writing, Review, and Editing, M.A-K., F.F., N.R., S.A.; Resources, F.F.; Supervision, F.F., All authors have read and approved the manuscript prior to submission.

## Availability of Data and Materials

 The data supporting the findings of this study are available in the NCDRC; nevertheless, restrictions apply to the availability of these data; therefore, they are not publicly available. Data are, however, available from the corresponding author upon reasonable request.

## Conflict of Interests

 The authors declare that there is no conflict of interest.

## Ethical Considerations

 This study was approved by the Ethical Committee of Endocrinology and Metabolism Research Institute of Tehran University of Medical Sciences, under code IR.TUMS.EMRI.REC.1397.026.

## Funding

 This research was funded by the Ministry of Health and Medical Education of Iran with grant No.1397-01-101-2264.

**Highlights**

- Lower urbanization was associated with higher current daily cigarette smoking
- Women’s low wealth, education, or urbanization predicted active/passive smoking
- Men’s high wealth or education predicted tobacco smoking
- Men’s lower wealth, education, or urbanization predicted passive smoking

## Supplementary files


Supplementary file 1. The prevalence of smoking among provinces in Iran.

Supplementary file 2. The prevalence of smoking among districts in Iran.

Supplementary file 3. Decomposition of the gap in exposure to secondhand smoking at home between the first and fifth quintiles of wealth index among women.

Supplementary file 4. Decomposition of the gap in exposure to secondhand smoking between the first and fifth quintiles of education index among both men and women.

Supplementary file 5. Decomposition of the gap in current daily cigarette smoking between the first and fifth quintiles of urbanization index among both genders.
