# Supplementary material for: Current Inequities in Smoking Prevalence on District Level in Iran: A Systematic Analysis on the STEPS Survey
Source: J Res Health Sci. 2021 Dec 28;22(1):e00540. doi: 10.34172/jrhs.2022.75 (PMC9315459; doi:10.34172/jrhs.2022.75)
Supplement: Supplementary file 1 — The prevalence of smoking among provinces in Iran. [file jrhs-22-e00540-s001.pdf]

Supplementary file 1: The prevalence of smoking among provinces in Iran

Type of smoking	Province	Gender	Prevalence (%)		
			Max	Min	Range
Current daily cigarette smoke	Alborz	Female	0.68	0.17	0.51
		Male	29.64	24.43	5.21
	Ardebil	Female	0.98	0.23	0.75
		Male	27.72	20.51	7.21
	Azararbayjan_East	Female	1.75	0.25	1.50
		Male	23.57	16.23	7.34
	Azarbayjan_West	Female	2.07	0.27	1.80
		Male	32.39	20.83	11.56
	Boushehr	Female	4.54	1.35	3.19
		Male	16.14	11.65	4.49
	Chaharmahal	Female	0.58	0.12	0.46
		Male	24.75	17.35	7.40
	Fars	Female	2.13	0.24	1.89
		Male	35.28	8.62	26.66
	Gilan	Female	0.86	0.12	0.75
		Male	26.46	16.30	10.16
	Golestan	Female	1.11	0.12	0.99
		Male	10.97	7.25	3.73
	Hamedan	Female	0.89	0.00	0.89
		Male	24.78	17.62	7.16
	Hormozgan	Female	1.39	0.28	1.11
		Male	21.07	8.45	12.61
	Ilam	Female	0.78	0.18	0.61
		Male	12.62	4.72	7.90
	Isfahan	Female	1.44	0.15	1.29
		Male	23.11	15.29	7.82
	Kerman	Female	2.20	0.18	2.02
		Male	40.91	5.17	35.74
	Kermanshah	Female	1.46	0.49	0.97
		Male	21.47	6.72	14.75
	Khorasan_North	Female	0.97	0.28	0.69
		Male	11.66	7.79	3.87
	Khorasan_South	Female	2.49	0.67	1.82
		Male	16.18	4.99	11.19
	Khorasan_razavi	Female	0.86	0.19	0.67
		Male	18.85	4.62	14.23
	Khuzestan	Female	0.76	0.06	0.70
		Male	23.00	6.90	16.11
	Kohkiluye and Bouyer Ahmad	Female	0.99	0.35	0.64
		Male	19.71	10.20	9.51
	Kordestan	Female	0.79	0.12	0.68
		Male	29.46	16.07	13.40
	Lorestan	Female	0.52	0.04	0.48
		Male	22.55	15.20	7.35
	Markazi	Female	3.19	0.46	2.72

		Male	30.97	22.03	8.93
	Mazandaran	Female	1.00	0.14	0.86
		Male	40.35	7.35	32.99
	Qazvin	Female	1.01	0.31	0.70
		Male	26.57	22.35	4.22
	Qom	Female	0.81	0.81	0.00
		Male	18.69	18.69	0.00
	Semnan	Female	1.03	0.16	0.87
		Male	22.86	14.03	8.84
	Sistan and Balouchestan	Female	4.14	1.10	3.05
		Male	22.62	5.63	16.99
	Tehran	Female	1.45	0.31	1.15
		Male	22.30	11.06	11.24
	Yazd	Female	1.24	0.36	0.87
		Male	20.46	12.85	7.61
	Zanjan	Female	0.98	0.20	0.77
		Male	22.49	9.42	13.07
Current tobacco smoke	Alborz	Female	1.56	0.40	1.16
		Male	34.51	28.97	5.54
	Ardebil	Female	1.21	0.33	0.88
		Male	27.09	23.36	3.74
	Azararbayjan_East	Female	2.42	0.31	2.11
		Male	25.35	19.61	5.74
	Azarbayjan_West	Female	3.88	0.58	3.30
		Male	31.82	23.43	8.39
	Boushehr	Female	18.25	11.93	6.32
		Male	33.22	23.24	9.98
	Chaharmahal	Female	1.30	0.41	0.89
		Male	35.16	27.34	7.83
	Fars	Female	14.50	3.81	10.68
		Male	41.31	19.54	21.77
	Gilan	Female	1.75	0.30	1.45
		Male	27.79	19.48	8.31
	Golestan	Female	2.60	0.96	1.64
		Male	21.51	11.82	9.69
	Hamedan	Female	3.91	0.46	3.44
		Male	32.03	24.36	7.67
	Hormozgan	Female	14.61	11.11	3.50
		Male	35.15	13.58	21.56
	Ilam	Female	0.71	0.14	0.57
		Male	17.68	7.35	10.33
	Isfahan	Female	4.35	1.02	3.32
		Male	30.83	23.06	7.77
	Kerman	Female	4.59	0.66	3.93
		Male	47.15	9.62	37.53
	Kermanshah	Female	4.27	0.68	3.58
		Male	24.52	15.49	9.03
	Khorasan_North	Female	5.32	1.81	3.50

		Male	22.41	15.43	6.99
	Khorasan_South	Female	3.89	1.38	2.51
		Male	18.17	9.18	8.99
	Khorasan_razavi	Female	18.81	4.51	14.30
		Male	22.68	8.49	14.19
	Khuzestan	Female	2.59	0.36	2.24
		Male	27.21	10.91	16.30
	Kohkiluye and Bouyer Ahmad	Female	10.53	6.63	3.90
		Male	28.74	16.65	12.10
	Kordestan	Female	2.30	0.46	1.84
		Male	31.53	18.04	13.48
	Lorestan	Female	5.44	1.84	3.60
		Male	32.12	21.64	10.48
	Markazi	Female	11.36	1.74	9.62
		Male	33.88	25.02	8.86
	Mazandaran	Female	3.95	0.87	3.09
		Male	45.16	12.90	32.27
	Qazvin	Female	3.82	1.32	2.50
		Male	43.35	32.18	11.18
	Qom	Female	3.82	3.82	0.00
		Male	24.38	24.38	0.00
	Semnan	Female	0.77	0.18	0.58
		Male	23.65	18.90	4.75
	Sistan and Balouchestan	Female	15.19	5.20	9.99
		Male	26.10	9.10	17.00
	Tehran	Female	2.93	0.89	2.04
		Male	25.97	13.53	12.44
	Yazd	Female	11.07	2.02	9.04
		Male	29.36	19.09	10.27
	Zanjan	Female	2.54	0.72	1.82
		Male	25.76	22.73	3.03
Ever cigarette smoke	Alborz	Female	1.22	0.54	0.68
		Male	34.18	29.34	4.84
	Ardebil	Female	2.30	0.90	1.40
		Male	32.25	28.49	3.75
	Azararbayjan_East	Female	9.40	0.74	8.66
		Male	29.61	21.40	8.22
	Azarbayjan_West	Female	6.26	1.18	5.08
		Male	58.55	32.69	25.85
	Boushehr	Female	4.75	1.40	3.35
		Male	18.85	14.58	4.26
	Chaharmahal	Female	0.35	0.12	0.23
		Male	33.35	23.94	9.41
	Fars	Female	2.43	0.62	1.80
		Male	42.95	13.94	29.01
	Gilan	Female	0.97	0.29	0.68
		Male	31.14	25.89	5.25
	Golestan	Female	2.29	0.66	1.63

		Male	18.01	12.64	5.37
	Hamedan	Female	6.91	0.82	6.09
		Male	35.15	28.49	6.66
	Hormozgan	Female	1.42	0.40	1.02
		Male	28.01	12.40	15.61
	Ilam	Female	3.18	1.04	2.13
		Male	19.29	14.75	4.53
	Isfahan	Female	1.49	0.52	0.97
		Male	30.45	23.89	6.55
	Kerman	Female	2.91	0.36	2.55
		Male	30.96	20.62	10.34
	Kermanshah	Female	2.58	0.91	1.66
		Male	33.21	16.95	16.26
	Khorasan_North	Female	1.15	0.42	0.73
		Male	16.47	11.21	5.26
	Khorasan_South	Female	3.37	1.20	2.17
		Male	18.34	9.85	8.50
	Khorasan_razavi	Female	1.00	0.28	0.72
		Male	27.82	10.44	17.38
	Khuzestan	Female	1.91	0.24	1.68
		Male	39.02	11.39	27.63
	Kohkiluye and Bouyer Ahmad	Female	2.60	0.93	1.67
		Male	24.17	15.62	8.56
	Kordestan	Female	4.99	2.48	2.52
		Male	41.84	28.99	12.86
	Lorestan	Female	0.92	0.29	0.63
		Male	30.08	24.02	6.06
	Markazi	Female	4.89	0.93	3.96
		Male	38.30	29.32	8.98
	Mazandaran	Female	1.24	0.27	0.96
		Male	43.21	19.80	23.41
	Qazvin	Female	1.44	0.72	0.72
		Male	36.08	29.61	6.47
	Qom	Female	1.51	1.51	0.00
		Male	26.05	26.05	0.00
	Semnan	Female	0.90	0.25	0.65
		Male	25.55	19.61	5.94
	Sistan and Balouchestan	Female	6.03	1.77	4.27
		Male	32.83	10.89	21.93
	Tehran	Female	1.92	0.68	1.24
		Male	26.67	20.23	6.44
	Yazd	Female	2.41	0.77	1.64
		Male	42.56	18.42	24.14
	Zanjan	Female	1.34	0.44	0.90
		Male	29.27	23.42	5.85
Ever tobacco smoke	Alborz	Female	2.84	1.38	1.46
		Male	40.86	35.20	5.65
	Ardebil	Female	3.08	1.18	1.90

		Male	37.62	32.57	5.05
	Azararbayjan_East	Female	10.48	0.69	9.78
		Male	35.28	27.85	7.44
	Azarbayjan_West	Female	8.54	2.16	6.38
		Male	53.13	38.47	14.66
	Boushehr	Female	24.84	15.24	9.60
		Male	49.44	27.63	21.81
	Chaharmahal	Female	2.77	0.97	1.79
		Male	45.02	39.93	5.10
	Fars	Female	31.75	9.17	22.58
		Male	50.98	26.72	24.26
	Gilan	Female	2.52	0.83	1.69
		Male	36.50	30.24	6.26
	Golestan	Female	7.03	2.93	4.10
		Male	45.37	30.27	15.10
	Hamedan	Female	14.34	2.50	11.85
		Male	45.54	33.22	12.33
	Hormozgan	Female	20.57	12.85	7.72
		Male	44.56	20.64	23.92
	Ilam	Female	5.56	1.38	4.18
		Male	28.81	23.16	5.65
	Isfahan	Female	9.67	2.16	7.52
		Male	45.40	36.61	8.79
	Kerman	Female	6.70	1.71	4.99
		Male	60.04	30.00	30.03
	Kermanshah	Female	6.76	1.64	5.11
		Male	33.70	24.49	9.21
	Khorasan_North	Female	6.81	3.26	3.55
		Male	30.12	22.30	7.82
	Khorasan_South	Female	6.28	2.28	4.00
		Male	22.65	14.06	8.59
	Khorasan_razavi	Female	20.86	6.37	14.49
		Male	41.05	24.54	16.51
	Khuzestan	Female	4.34	1.23	3.11
		Male	41.34	15.92	25.42
	Kohkiluye and Bouyer Ahmad	Female	23.20	15.86	7.35
		Male	46.25	27.61	18.64
	Kordestan	Female	8.61	2.70	5.91
		Male	47.53	31.73	15.80
	Lorestan	Female	12.54	3.76	8.78
		Male	39.38	32.51	6.87
	Markazi	Female	13.43	3.26	10.16
		Male	42.80	31.70	11.10
	Mazandaran	Female	6.44	1.38	5.07
		Male	47.37	31.97	15.40
	Qazvin	Female	10.35	3.36	6.99
		Male	60.11	48.60	11.51
	Qom	Female	6.59	6.59	0.00

		Male	34.82	34.82	0.00
	Semnan	Female	4.96	1.43	3.53
		Male	33.07	28.44	4.63
	Sistan and Balouchestan	Female	25.99	13.03	12.96
		Male	35.11	17.62	17.49
	Tehran	Female	4.63	1.31	3.32
		Male	34.28	25.91	8.37
	Yazd	Female	9.05	2.89	6.16
		Male	52.98	27.04	25.94
	Zanjan	Female	10.54	2.55	7.99
		Male	41.35	33.36	8.00
Exposure to secondhand smoke	Alborz	Female	33.85	16.72	17.13
		Male	40.51	30.45	10.07
	Ardebil	Female	40.80	31.02	9.78
		Male	66.49	35.76	30.73
	Azararbayjan_East	Female	41.00	9.17	31.83
		Male	57.50	14.65	42.85
	Azarbayjan_West	Female	66.34	22.47	43.87
		Male	65.42	11.36	54.06
	Boushehr	Female	35.40	30.46	4.95
		Male	59.36	22.30	37.06
	Chaharmahal	Female	41.78	26.86	14.93
		Male	52.91	39.94	12.98
	Fars	Female	71.45	35.09	36.37
		Male	63.19	32.79	30.40
	Gilan	Female	30.56	8.68	21.88
		Male	44.06	14.75	29.32
	Golestan	Female	31.73	11.38	20.35
		Male	34.68	26.43	8.25
	Hamedan	Female	56.17	27.51	28.67
		Male	59.78	24.48	35.30
	Hormozgan	Female	46.52	29.19	17.33
		Male	54.29	15.70	38.59
	Ilam	Female	29.90	13.77	16.13
		Male	70.96	27.08	43.88
	Isfahan	Female	45.10	18.03	27.07
		Male	62.21	30.28	31.93
	Kerman	Female	49.22	20.21	29.01
		Male	61.51	21.88	39.63
	Kermanshah	Female	60.33	9.50	50.83
		Male	51.34	31.92	19.42
	Khorasan_North	Female	30.18	18.01	12.16
		Male	41.00	27.54	13.45
	Khorasan_South	Female	21.89	12.56	9.32
		Male	31.81	15.52	16.29
	Khorasan_razavi	Female	53.36	15.15	38.22
		Male	52.96	22.40	30.56
	Khuzestan	Female	61.75	19.20	42.55

		Male	63.07	31.83	31.24
	Kohkiluye and Bouyer Ahmad	Female	57.13	38.95	18.18
		Male	75.62	49.83	25.79
	Kordestan	Female	56.36	21.84	34.52
		Male	62.57	21.71	40.86
	Lorestan	Female	42.34	19.71	22.63
		Male	50.64	26.29	24.35
	Markazi	Female	62.18	15.87	46.32
		Male	62.41	33.04	29.37
	Mazandaran	Female	41.41	8.61	32.80
		Male	53.44	20.76	32.68
	Qazvin	Female	54.45	43.93	10.52
		Male	61.85	46.14	15.72
	Qom	Female	28.78	28.78	0.00
		Male	37.75	37.75	0.00
	Semnan	Female	21.93	14.59	7.34
		Male	29.20	24.77	4.43
	Sistan and Balouchestan	Female	41.01	16.78	24.23
		Male	48.84	8.96	39.88
	Tehran	Female	26.72	7.42	19.30
		Male	34.59	17.46	17.13
	Yazd	Female	55.13	20.38	34.75
		Male	51.11	31.19	19.92
	Zanjan	Female	60.99	31.73	29.27
		Male	64.36	45.41	18.95
Exposure to secondhand smoke at home	Alborz	Female	33.56	15.55	18.01
		Male	28.07	18.31	9.76
	Ardebil	Female	37.21	29.38	7.83
		Male	47.81	23.34	24.46
	Azararbayjan_East	Female	37.65	8.31	29.34
		Male	41.74	9.97	31.77
	Azarbayjan_West	Female	65.53	21.28	44.25
		Male	59.25	7.60	51.65
	Boushehr	Female	38.15	29.23	8.92
		Male	54.76	20.85	33.91
	Chaharmahal	Female	40.44	25.05	15.39
		Male	44.59	22.93	21.65
	Fars	Female	58.25	35.02	23.23
		Male	56.39	24.62	31.77
	Gilan	Female	25.66	8.59	17.07
		Male	27.12	12.57	14.55
	Golestan	Female	31.56	6.30	25.25
		Male	32.97	12.14	20.83
	Hamedan	Female	54.65	27.17	27.48
		Male	44.51	33.99	10.52
	Hormozgan	Female	35.91	27.48	8.43
		Male	47.63	11.44	36.19
	Ilam	Female	28.87	12.89	15.98

		Male	45.79	25.33	20.45
	Isfahan	Female	41.91	8.45	33.46
		Male	38.22	14.94	23.28
	Kerman	Female	37.25	19.46	17.80
		Male	57.57	19.19	38.38
	Kermanshah	Female	60.05	8.47	51.58
		Male	48.09	24.15	23.94
	Khorasan_North	Female	30.43	18.13	12.30
		Male	36.72	16.67	20.05
	Khorasan_South	Female	21.52	11.73	9.79
		Male	23.69	10.91	12.78
	Khorasan_razavi	Female	51.35	11.24	40.11
		Male	35.19	17.08	18.11
	Khuzestan	Female	61.36	17.90	43.46
		Male	58.01	24.33	33.68
	Kohkiluye and Bouyer Ahmad	Female	56.85	37.91	18.95
		Male	74.63	44.07	30.56
	Kordestan	Female	56.19	21.51	34.68
		Male	52.20	19.62	32.59
	Lorestan	Female	39.35	18.53	20.81
		Male	44.74	21.42	23.32
	Markazi	Female	62.84	15.23	47.61
		Male	49.69	26.84	22.85
	Mazandaran	Female	40.51	7.17	33.34
		Male	32.94	11.68	21.26
	Qazvin	Female	51.34	42.70	8.64
		Male	46.54	35.42	11.12
	Qom	Female	27.13	27.13	0.00
		Male	26.83	26.83	0.00
	Semnan	Female	19.68	12.48	7.20
		Male	18.57	14.14	4.43
	Sistan and Balouchestan	Female	38.88	16.60	22.27
		Male	42.61	7.71	34.90
	Tehran	Female	25.23	6.33	18.90
		Male	23.41	8.19	15.23
	Yazd	Female	54.67	19.61	35.06
		Male	37.79	14.77	23.02
	Zanjan	Female	58.63	30.19	28.44
		Male	49.43	33.11	16.32
Past daily cigarette smoke	Alborz	Female	1.13	0.49	0.64
		Male	32.40	27.95	4.45
	Ardebil	Female	2.62	0.85	1.77
		Male	31.85	28.89	2.96
	Azararbayjan_East	Female	9.45	0.76	8.69
		Male	29.15	21.07	8.08
	Azarbayjan_West	Female	6.49	1.08	5.41
		Male	44.14	32.39	11.75
	Boushehr	Female	1.83	0.50	1.33

		Male	16.13	11.64	4.49
	Chaharmahal	Female	0.46	0.14	0.32
		Male	31.32	23.84	7.48
	Fars	Female	2.23	0.49	1.73
		Male	30.25	14.13	16.12
	Gilan	Female	1.00	0.28	0.73
		Male	29.33	23.20	6.13
	Golestan	Female	2.33	0.59	1.74
		Male	17.25	12.56	4.68
	Hamedan	Female	6.98	0.82	6.16
		Male	34.09	27.05	7.03
	Hormozgan	Female	1.15	0.25	0.90
		Male	27.91	13.01	14.90
	Ilam	Female	3.16	0.99	2.17
		Male	18.77	14.66	4.12
	Isfahan	Female	1.75	0.47	1.28
		Male	29.00	24.10	4.90
	Kerman	Female	3.35	0.32	3.03
		Male	28.90	20.72	8.18
	Kermanshah	Female	2.38	0.81	1.57
		Male	31.42	17.21	14.20
	Khorasan_North	Female	0.99	0.44	0.54
		Male	16.13	11.46	4.67
	Khorasan_South	Female	2.69	0.97	1.72
		Male	17.82	9.88	7.94
	Khorasan_razavi	Female	1.05	0.20	0.85
		Male	26.93	10.65	16.29
	Khuzestan	Female	2.61	0.23	2.37
		Male	37.66	11.78	25.88
	Kohkiluye and Bouyer Ahmad	Female	2.71	0.88	1.82
		Male	23.40	16.18	7.23
	Kordestan	Female	5.45	1.52	3.93
		Male	37.38	27.98	9.40
	Lorestan	Female	0.74	0.20	0.55
		Male	28.20	23.30	4.89
	Markazi	Female	4.99	0.94	4.04
		Male	36.65	28.61	8.04
	Mazandaran	Female	1.15	0.19	0.96
		Male	40.23	19.54	20.69
	Qazvin	Female	1.44	0.71	0.73
		Male	34.73	28.61	6.12
	Qom	Female	1.20	1.20	0.00
		Male	24.16	24.16	0.00
	Semnan	Female	0.93	0.27	0.66
		Male	23.49	18.70	4.79
	Sistan and Balouchestan	Female	6.32	0.64	5.68
		Male	31.48	11.59	19.89
	Tehran	Female	1.99	0.43	1.57

		Male	24.12	18.59	5.53
	Yazd	Female	2.43	0.78	1.66
		Male	40.42	18.49	21.93
	Zanjan	Female	1.26	0.49	0.77
		Male	27.71	22.52	5.19
