# Supplementary material for: Current Inequities in Smoking Prevalence on District Level in Iran: A Systematic Analysis on the STEPS Survey
Source: J Res Health Sci. 2021 Dec 28;22(1):e00540. doi: 10.34172/jrhs.2022.75 (PMC9315459; doi:10.34172/jrhs.2022.75)
Supplement: Supplementary file 2 — The prevalence of smoking among districts in Iran. [file jrhs-22-e00540-s002.pdf]

Supplementary file 2. The prevalence of smoking among districts in Iran.

Row	Smoking type	Province	District	Sex	Prevalence (%)	95% UI (lower limit)	95% UI (upper limit)
0	Current daily cigarette smoke	Khuzestan	Abadan	Female	0.08	0.00	3.35
1	Current daily cigarette smoke	Fars	Abadeh	Female	0.24	0.00	6.81
2	Current daily cigarette smoke	Yazd	Abarkuh	Female	0.85	0.00	9.68
3	Current daily cigarette smoke	Mazandaran	Abbas abad	Female	0.48	0.00	7.04
4	Current daily cigarette smoke	Ilam	Abdanan	Female	0.44	0.00	5.20
5	Current daily cigarette smoke	Zanjan	Abhar	Female	0.22	0.00	4.16
6	Current daily cigarette smoke	Hormozgan	Abumusa	Female	0.84	0.00	7.73
7	Current daily cigarette smoke	Qazvin	Abyek	Female	0.60	0.00	4.57
8	Current daily cigarette smoke	Azararbayjan_East	Ahar	Female	1.58	0.00	6.22
9	Current daily cigarette smoke	Khuzestan	Ahvaz	Female	0.65	0.00	3.75
10	Current daily cigarette smoke	Azararbayjan_East	Ajabshir	Female	0.56	0.00	5.90
11	Current daily cigarette smoke	Qazvin	Alborz	Female	1.01	0.00	4.66
12	Current daily cigarette smoke	Golestan	Aliabad	Female	0.22	0.00	6.06
13	Current daily cigarette smoke	Lorestan	Aligudarz	Female	0.07	0.00	1.36
14	Current daily cigarette smoke	Gilan	Amlash	Female	0.17	0.00	4.47
15	Current daily cigarette smoke	Mazandaran	Amol	Female	0.19	0.00	4.48
16	Current daily cigarette smoke	Kerman	Anar	Female	0.43	0.00	2.39
17	Current daily cigarette smoke	Kerman	Anbarabad	Female	0.18	0.00	1.50
18	Current daily cigarette smoke	Khuzestan	Andika	Female	0.28	0.00	4.44
19	Current daily cigarette smoke	Khuzestan	Andimeshk	Female	0.26	0.00	3.33
20	Current daily cigarette smoke	Golestan	Aq Qala	Female	0.12	0.00	6.16
21	Current daily cigarette smoke	Khuzestan	Aqajari	Female	0.19	0.00	4.62
22	Current daily cigarette smoke	Semnan	Aradan	Female	0.34	0.00	4.99
23	Current daily cigarette smoke	Markazi	Arak	Female	0.51	0.00	1.20
24	Current daily cigarette smoke	Isfahan	Aran and Bidgol	Female	0.23	0.00	6.52
25	Current daily cigarette smoke	Ardebil	Ardabil	Female	0.98	0.00	4.75
26	Current daily cigarette smoke	Yazd	Ardakan	Female	0.36	0.00	6.51
27	Current daily cigarette smoke	Chaharmahal	Ardal	Female	0.13	0.00	2.15
28	Current daily cigarette smoke	Isfahan	Ardestan	Female	0.37	0.00	8.60
29	Current daily cigarette smoke	Fars	Arsanjan	Female	0.80	0.00	9.70
30	Current daily cigarette smoke	Kerman	Arzouyeh	Female	0.41	0.00	2.28
31	Current daily cigarette smoke	Hamedan	Asadabad	Female	0.29	0.00	5.54
32	Current daily cigarette smoke	Boushehr	Asaluyeh	Female	2.54	0.00	13.18
33	Current daily cigarette smoke	Markazi	Ashtiyan	Female	0.79	0.00	1.77
34	Current daily cigarette smoke	Gilan	Astaneh-ye-Ashrafiyeh	Female	0.65	0.00	4.96
35	Current daily cigarette smoke	Gilan	Astara	Female	0.39	0.00	5.52
36	Current daily cigarette smoke	Qazvin	Avaj	Female	0.57	0.00	4.70
37	Current daily cigarette smoke	Golestan	Azadshahr	Female	0.31	0.00	8.62
38	Current daily cigarette smoke	Azararbayjan_East	Azarshahr	Female	0.32	0.00	4.59
39	Current daily cigarette smoke	Lorestan	Azna	Female	0.41	0.00	2.32
40	Current daily cigarette smoke	Mazandaran	Babol	Female	0.86	0.00	5.47
41	Current daily cigarette smoke	Mazandaran	Babolsar	Female	0.43	0.00	6.83
42	Current daily cigarette smoke	Ilam	Badreh	Female	0.37	0.00	4.81
43	Current daily cigarette smoke	Yazd	Bafq	Female	0.52	0.00	6.77
44	Current daily cigarette smoke	Kerman	Baft	Female	0.30	0.00	1.62
45	Current daily cigarette smoke	Khuzestan	Baghemalek	Female	0.10	0.00	3.04
46	Current daily cigarette smoke	Yazd	Bahabad	Female	0.71	0.00	9.60
47	Current daily cigarette smoke	Hamedan	Bahar	Female	0.89	0.00	7.81
48	Current daily cigarette smoke	Tehran	Baharestan (Golestan)	Female	1.05	0.00	4.84
49	Current daily cigarette smoke	Kohkiluye and Bouyer Ahmad	Bahmani	Female	0.36	0.00	3.09
50	Current daily cigarette smoke	Khorasan_razavi	Bajestan	Female	0.37	0.00	4.29
51	Current daily cigarette smoke	Khorasan_razavi	Bakhraz	Female	0.21	0.00	3.07
52	Current daily cigarette smoke	Kerman	Bam	Female	0.20	0.00	1.51
53	Current daily cigarette smoke	Hormozgan	Bandar-e-Abbas	Female	0.99	0.00	5.28
54	Current daily cigarette smoke	Gilan	Bandar-e-Anzali	Female	0.86	0.00	4.65
55	Current daily cigarette smoke	Golestan	Bandar-e-Gaz	Female	0.31	0.00	10.13
56	Current daily cigarette smoke	Hormozgan	Bandar-e-Jask	Female	0.40	0.00	6.41
57	Current daily cigarette smoke	Hormozgan	Bandar-e-Lengeh	Female	1.39	0.00	6.91
58	Current daily cigarette smoke	Khuzestan	Bandar-e-Mahshahr	Female	0.09	0.00	3.11
59	Current daily cigarette smoke	Golestan	Bandar-e-Torkaman	Female	0.25	0.00	9.25
60	Current daily cigarette smoke	Kordestan	Baneh	Female	0.12	0.00	3.50
61	Current daily cigarette smoke	Khorasan_razavi	Bardeskan	Female	0.33	0.00	4.26
62	Current daily cigarette smoke	Kerman	Bardsir	Female	2.20	0.81	3.53
63	Current daily cigarette smoke	Hormozgan	Bashagerd	Female	0.39	0.00	6.15
64	Current daily cigarette smoke	Kohkiluye and Bouyer Ahmad	Basht	Female	0.65	0.00	4.77
65	Current daily cigarette smoke	Hormozgan	Bastak	Female	0.84	0.00	6.83
66	Current daily cigarette smoke	Khuzestan	Bavi	Female	0.27	0.00	4.77
67	Current daily cigarette smoke	Khuzestan	Behbahan	Female	0.11	0.00	3.13
68	Current daily cigarette smoke	Mazandaran	Behshahr	Female	0.23	0.00	4.69
69	Current daily cigarette smoke	Kordestan	Bijar	Female	0.27	0.00	4.48
70	Current daily cigarette smoke	Ardebil	Bilehsavar	Female	0.54	0.00	5.83
71	Current daily cigarette smoke	Khorasan_razavi	Binaloud	Female	0.39	0.00	4.41
72	Current daily cigarette smoke	Khorasan_South	Birjand	Female	1.61	0.00	8.22
73	Current daily cigarette smoke	Khorasan_North	Bojnurd	Female	0.28	0.00	6.22
74	Current daily cigarette smoke	Chaharmahal	Bon	Female	0.23	0.00	3.35
75	Current daily cigarette smoke	Azararbayjan_East	Bonab	Female	0.35	0.00	4.33
76	Current daily cigarette smoke	Isfahan	Borkhar	Female	0.15	0.00	6.45
77	Current daily cigarette smoke	Isfahan	Borkhar and Meymeh	Female	1.12	0.00	8.82
78	Current daily cigarette smoke	Chaharmahal	Borujen	Female	0.13	0.00	2.19
79	Current daily cigarette smoke	Lorestan	Borujerd	Female	0.22	0.00	1.54
80	Current daily cigarette smoke	Khorasan_South	Boshruyeh	Female	1.11	0.00	10.36
81	Current daily cigarette smoke	Azararbayjan_East	Bostanabad	Female	0.50	0.00	4.18
82	Current daily cigarette smoke	Fars	Bovanat	Female	0.79	0.00	9.72
83	Current daily cigarette smoke	Kohkiluye and Bouyer Ahmad	Boyer Ahmad	Female	0.98	0.00	4.20
84	Current daily cigarette smoke	Qazvin	Boyinzahra	Female	0.32	0.00	3.23
85	Current daily cigarette smoke	Isfahan	Buein va Miandasht	Female	0.88	0.00	9.78
86	Current daily cigarette smoke	Azarbayjan_West	Bukan	Female	0.36	0.00	5.76
87	Current daily cigarette smoke	Boushehr	Bushehr	Female	3.54	0.00	13.74
88	Current daily cigarette smoke	Isfahan	Chadegan	Female	0.69	0.00	9.15
89	Current daily cigarette smoke	Sistan and Balouchestan	Chahbahar	Female	3.18	0.00	17.96
90	Current daily cigarette smoke	Azarbayjan_West	Chaipareh	Female	0.70	0.00	8.36
91	Current daily cigarette smoke	Azarbayjan_West	Chaldoran	Female	0.76	0.00	8.71
92	Current daily cigarette smoke	Mazandaran	Chalus	Female	0.21	0.00	4.52
93	Current daily cigarette smoke	Azararbayjan_East	Charoimaq	Female	0.67	0.00	5.68

**94**	Current daily cigarette smoke	Khorasan_razavi	Chenaran	Female	0.20	0.00	3.02
**95**	Current daily cigarette smoke	Kohkiluye and Bouyer Ahmad	Cheram	Female	0.66	0.00	4.61
**96**	Current daily cigarette smoke	Kermanshah	Dalaho	Female	0.88	0.00	6.94
**97**	Current daily cigarette smoke	Lorestan	Dalfan	Female	0.04	0.00	1.33
**98**	Current daily cigarette smoke	Sistan and Balouchestan	Dalgan	Female	1.70	0.00	15.90
**99**	Current daily cigarette smoke	Tehran	Damavand	Female	0.32	0.00	3.54
**100**	Current daily cigarette smoke	Semnan	Damghan	Female	0.26	0.00	3.49
**101**	Current daily cigarette smoke	Fars	Darab	Female	1.37	0.00	9.49
**102**	Current daily cigarette smoke	Khorasan_South	Darmian	Female	2.49	0.00	11.52
**103**	Current daily cigarette smoke	Khorasan_razavi	Darrehgaz	Female	0.20	0.00	3.07
**104**	Current daily cigarette smoke	Ilam	Darrehshahr	Female	0.23	0.00	3.55
**105**	Current daily cigarette smoke	Khuzestan	Dasht-e-Azadegan	Female	0.13	0.00	3.28
**106**	Current daily cigarette smoke	Boushehr	Dashtestan	Female	1.57	0.00	8.76
**107**	Current daily cigarette smoke	Boushehr	Dashti	Female	4.54	0.00	14.83
**108**	Current daily cigarette smoke	Khorasan_razavi	Davarzan	Female	0.30	0.00	4.19
**109**	Current daily cigarette smoke	Boushehr	Dayyer	Female	2.84	0.00	14.33
**110**	Current daily cigarette smoke	Kordestan	Dehgolan	Female	0.38	0.00	4.75
**111**	Current daily cigarette smoke	Ilam	Dehloran	Female	0.78	0.00	4.94
**112**	Current daily cigarette smoke	Markazi	Delijan	Female	0.80	0.00	1.77
**113**	Current daily cigarette smoke	Kohkiluye and Bouyer Ahmad	Dena	Female	0.35	0.00	3.28
**114**	Current daily cigarette smoke	Boushehr	Deylam	Female	2.33	0.00	12.96
**115**	Current daily cigarette smoke	Khuzestan	Dezful	Female	0.22	0.00	3.19
**116**	Current daily cigarette smoke	Kordestan	Divandarreh	Female	0.30	0.00	4.76
**117**	Current daily cigarette smoke	Lorestan	Dorud	Female	0.52	0.00	2.19
**118**	Current daily cigarette smoke	Lorestan	Doureh	Female	0.11	0.00	1.92
**119**	Current daily cigarette smoke	Fars	Eqlid	Female	0.66	0.00	9.04
**120**	Current daily cigarette smoke	Khorasan_North	Esfarayen	Female	0.31	0.00	6.20
**121**	Current daily cigarette smoke	Alborz	Eshtehard	Female	0.32	0.00	4.79
**122**	Current daily cigarette smoke	Kermanshah	Eslamabad-e-Gharb	Female	1.46	0.00	7.69
**123**	Current daily cigarette smoke	Tehran	Eslamshahr	Female	0.39	0.00	3.73
**124**	Current daily cigarette smoke	Fars	Estahban	Female	0.83	0.00	9.68
**125**	Current daily cigarette smoke	Ilam	Eyvan	Female	0.18	0.00	3.53
**126**	Current daily cigarette smoke	Kerman	Fahraj	Female	0.38	0.00	2.20
**127**	Current daily cigarette smoke	Isfahan	Falavarjan	Female	0.30	0.00	6.47
**128**	Current daily cigarette smoke	Hamedan	Famenin	Female	0.00	0.00	7.09
**129**	Current daily cigarette smoke	Markazi	Farahan	Female	0.78	0.00	1.77
**130**	Current daily cigarette smoke	Fars	Farashband	Female	0.86	0.00	9.11
**131**	Current daily cigarette smoke	Alborz	Fardis	Female	0.47	0.00	5.57
**132**	Current daily cigarette smoke	Isfahan	Faridan	Female	0.46	0.00	6.57
**133**	Current daily cigarette smoke	Khorasan_razavi	Fariman	Female	0.27	0.00	3.12
**134**	Current daily cigarette smoke	Khorasan_North	Faroj	Female	0.38	0.00	6.25
**135**	Current daily cigarette smoke	Chaharmahal	Farsan	Female	0.12	0.00	2.16
**136**	Current daily cigarette smoke	Kerman	Faryab	Female	0.38	0.00	2.20
**137**	Current daily cigarette smoke	Fars	Fasa	Female	0.47	0.00	6.81
**138**	Current daily cigarette smoke	Khorasan_South	Ferdows	Female	1.13	0.00	10.57
**139**	Current daily cigarette smoke	Mazandaran	Fereydunkenar	Female	0.27	0.00	4.80
**140**	Current daily cigarette smoke	Isfahan	Fereydunshahr	Female	0.90	0.00	9.65
**141**	Current daily cigarette smoke	Fars	Firozabad	Female	0.53	0.00	6.67
**142**	Current daily cigarette smoke	Tehran	Firuzkuh	Female	0.37	0.00	3.61
**143**	Current daily cigarette smoke	Sistan and Balouchestan	Fonuj	Female	1.73	0.00	16.05
**144**	Current daily cigarette smoke	Gilan	Fuman	Female	0.15	0.00	2.95
**145**	Current daily cigarette smoke	Kohkiluye and Bouyer Ahmad	Gachsaran	Female	0.99	0.00	4.48
**146**	Current daily cigarette smoke	Golestan	Galikesh	Female	0.20	0.00	6.34
**147**	Current daily cigarette smoke	Mazandaran	Galugah	Female	0.45	0.00	7.75
**148**	Current daily cigarette smoke	Semnan	Garmsar	Female	0.16	0.00	3.36
**149**	Current daily cigarette smoke	Boushehr	Genaveh	Female	1.35	0.00	8.73
**150**	Current daily cigarette smoke	Fars	Gerash	Female	0.64	0.00	10.06
**151**	Current daily cigarette smoke	Khorasan_North	Germeh	Female	0.42	0.00	9.19
**152**	Current daily cigarette smoke	Ardebil	Germi	Female	0.54	0.00	5.15
**153**	Current daily cigarette smoke	Kerman	Ghaleye-Ganj	Female	0.19	0.00	1.48
**154**	Current daily cigarette smoke	Kermanshah	Gilan-e-Gharb	Female	0.91	0.00	7.14
**155**	Current daily cigarette smoke	Isfahan	Golpayegan	Female	0.42	0.00	6.95
**156**	Current daily cigarette smoke	Golestan	Gomishan	Female	0.29	0.00	10.02
**157**	Current daily cigarette smoke	Khorasan_razavi	Gonabad	Female	0.40	0.00	4.23
**158**	Current daily cigarette smoke	Golestan	Gonbad-e-Kavus	Female	0.25	0.00	6.22
**159**	Current daily cigarette smoke	Golestan	Gorgan	Female	0.20	0.00	6.50
**160**	Current daily cigarette smoke	Khuzestan	Guotvand	Female	0.30	0.00	4.74
**161**	Current daily cigarette smoke	Khuzestan	Haftgol	Female	0.27	0.00	4.43
**162**	Current daily cigarette smoke	Hormozgan	Hajiabad	Female	0.46	0.00	4.38
**163**	Current daily cigarette smoke	Hamedan	Hamadan	Female	0.20	0.00	5.20
**164**	Current daily cigarette smoke	Khuzestan	Hamidiyeh	Female	0.26	0.00	4.57
**165**	Current daily cigarette smoke	Sistan and Balouchestan	Hamoon	Female	2.28	0.00	17.29
**166**	Current daily cigarette smoke	Azararbayjan_East	Haris	Female	1.75	0.00	6.64
**167**	Current daily cigarette smoke	Kermanshah	Harsin	Female	1.06	0.00	7.61
**168**	Current daily cigarette smoke	Azararbayjan_East	Hashtrud	Female	0.72	0.00	6.20
**169**	Current daily cigarette smoke	Khuzestan	Hendijan	Female	0.23	0.00	4.46
**170**	Current daily cigarette smoke	Sistan and Balouchestan	Hirmand	Female	2.89	0.00	18.47
**171**	Current daily cigarette smoke	Khuzestan	Hoveizeh	Female	0.27	0.00	4.80
**172**	Current daily cigarette smoke	Zanjan	Ijerod	Female	0.42	0.00	5.90
**173**	Current daily cigarette smoke	Ilam	Ilam	Female	0.19	0.00	3.49
**174**	Current daily cigarette smoke	Sistan and Balouchestan	Iranshahr	Female	1.10	0.00	10.71
**175**	Current daily cigarette smoke	Isfahan	Isfahan	Female	0.33	0.00	6.21
**176**	Current daily cigarette smoke	Khuzestan	Izeh	Female	0.15	0.00	3.16
**177**	Current daily cigarette smoke	Fars	Jahrom	Female	0.47	0.00	6.65
**178**	Current daily cigarette smoke	Khorasan_North	Jajarm	Female	0.74	0.00	8.98
**179**	Current daily cigarette smoke	Boushehr	Jam	Female	1.49	0.00	9.00
**180**	Current daily cigarette smoke	Kermanshah	Javanrud	Female	0.49	0.00	5.02
**181**	Current daily cigarette smoke	Kerman	Jiroft	Female	0.22	0.00	1.49
**182**	Current daily cigarette smoke	Khorasan_razavi	Joghatai	Female	0.23	0.00	4.30
**183**	Current daily cigarette smoke	Azararbayjan_East	Jolfa	Female	0.80	0.00	6.15
**184**	Current daily cigarette smoke	Khorasan_razavi	Jowayin	Female	0.31	0.00	4.34
**185**	Current daily cigarette smoke	Mazandaran	Juybar	Female	0.49	0.00	6.84
**186**	Current daily cigarette smoke	Hamedan	Kabudarahang	Female	0.05	0.00	5.13
**187**	Current daily cigarette smoke	Kerman	Kahnuj	Female	0.33	0.00	2.06
**188**	Current daily cigarette smoke	Golestan	Kalaleh	Female	1.11	0.00	10.02
**189**	Current daily cigarette smoke	Khorasan_razavi	Kalat	Female	0.36	0.00	4.60
**190**	Current daily cigarette smoke	Azararbayjan_East	Kaleibar	Female	0.99	0.00	6.53
**191**	Current daily cigarette smoke	Kordestan	Kamyaran	Female	0.26	0.00	3.36

**192**	Current daily cigarette smoke	Boushehr	Kangan	Female	3.67	0.00	14.73
**193**	Current daily cigarette smoke	Kermanshah	Kangavar	Female	0.67	0.00	4.91
**194**	Current daily cigarette smoke	Alborz	Karaj	Female	0.68	0.00	4.23
**195**	Current daily cigarette smoke	Khuzestan	Karun	Female	0.28	0.00	4.85
**196**	Current daily cigarette smoke	Isfahan	Kashan	Female	0.25	0.00	6.36
**197**	Current daily cigarette smoke	Khorasan_razavi	Kashmar	Female	0.19	0.00	2.91
**198**	Current daily cigarette smoke	Fars	Kavar	Female	0.85	0.00	9.97
**199**	Current daily cigarette smoke	Fars	Kazerun	Female	0.40	0.00	6.56
**200**	Current daily cigarette smoke	Mazandaran	Kelardasht	Female	0.40	0.00	6.67
**201**	Current daily cigarette smoke	Kerman	Kerman	Female	0.26	0.00	1.50
**202**	Current daily cigarette smoke	Kermanshah	Kermanshah	Female	1.43	0.00	5.92
**203**	Current daily cigarette smoke	Khorasan_razavi	Khaf	Female	0.24	0.00	2.99
**204**	Current daily cigarette smoke	Khorasan_razavi	Khalilabad	Female	0.35	0.00	4.45
**205**	Current daily cigarette smoke	Ardebil	Khalkhal	Female	0.23	0.00	3.43
**206**	Current daily cigarette smoke	Hormozgan	Khamir	Female	0.75	0.00	6.61
**207**	Current daily cigarette smoke	Isfahan	Khansar	Female	0.71	0.00	9.39
**208**	Current daily cigarette smoke	Sistan and Balouchestan	Khash	Female	1.29	0.00	10.95
**209**	Current daily cigarette smoke	Yazd	Khatam	Female	0.94	0.00	9.59
**210**	Current daily cigarette smoke	Fars	Kherameh	Female	0.88	0.00	9.21
**211**	Current daily cigarette smoke	Azararbayjan_East	Khodaafarin	Female	0.87	0.00	6.41
**212**	Current daily cigarette smoke	Zanjan	Khodabandeh	Female	0.26	0.00	3.93
**213**	Current daily cigarette smoke	Markazi	Khomeyn	Female	0.54	0.00	1.24
**214**	Current daily cigarette smoke	Isfahan	Khomeynishahr	Female	0.33	0.00	6.65
**215**	Current daily cigarette smoke	Markazi	Khondab	Female	0.55	0.00	1.25
**216**	Current daily cigarette smoke	Fars	Khonj	Female	0.79	0.00	9.41
**217**	Current daily cigarette smoke	Isfahan	Khoor va Biabanak	Female	0.43	0.00	9.22
**218**	Current daily cigarette smoke	Lorestan	Khorramabad	Female	0.09	0.00	1.34
**219**	Current daily cigarette smoke	Fars	Khorrambid	Female	0.78	0.00	9.82
**220**	Current daily cigarette smoke	Zanjan	Khorramdarreh	Female	0.22	0.00	4.16
**221**	Current daily cigarette smoke	Khuzestan	Khorramshahr	Female	0.06	0.00	3.14
**222**	Current daily cigarette smoke	Khorasan_razavi	Khoshab	Female	0.35	0.00	4.29
**223**	Current daily cigarette smoke	Azarbayjan_West	Khoy	Female	0.37	0.00	5.81
**224**	Current daily cigarette smoke	Khorasan_South	Khusef	Female	1.29	0.00	9.88
**225**	Current daily cigarette smoke	Chaharmahal	Kiaar	Female	0.13	0.00	2.13
**226**	Current daily cigarette smoke	Kohkiluye and Bouyer Ahmad	Kohgiluyeh	Female	0.38	0.00	3.18
**227**	Current daily cigarette smoke	Markazi	Komeijan	Female	0.79	0.00	1.73
**228**	Current daily cigarette smoke	Sistan and Balouchestan	Konarak	Female	1.85	0.00	16.49
**229**	Current daily cigarette smoke	Golestan	Kordkuy	Female	0.25	0.00	9.07
**230**	Current daily cigarette smoke	Ardebil	Kowsar	Female	0.42	0.00	5.14
**231**	Current daily cigarette smoke	Kerman	Kuhbonan	Female	0.37	0.00	2.27
**232**	Current daily cigarette smoke	Lorestan	Kuhdasht	Female	0.05	0.00	1.35
**233**	Current daily cigarette smoke	Chaharmahal	Kuhrang	Female	0.28	0.00	2.97
**234**	Current daily cigarette smoke	Gilan	Lahijan	Female	0.18	0.00	3.14
**235**	Current daily cigarette smoke	Khuzestan	Lali	Female	0.36	0.00	4.77
**236**	Current daily cigarette smoke	Fars	Lamard	Female	0.42	0.00	6.71
**237**	Current daily cigarette smoke	Kohkiluye and Bouyer Ahmad	Landeh	Female	0.54	0.00	4.99
**238**	Current daily cigarette smoke	Gilan	Langrud	Female	0.12	0.00	3.09
**239**	Current daily cigarette smoke	Isfahan	Lanjan	Female	0.42	0.00	6.36
**240**	Current daily cigarette smoke	Fars	Lar (Larestan)	Female	0.45	0.00	6.74
**241**	Current daily cigarette smoke	Chaharmahal	Lordakan	Female	0.58	0.00	3.03
**242**	Current daily cigarette smoke	Azarbayjan_West	Mahabad	Female	1.28	0.00	8.60
**243**	Current daily cigarette smoke	Markazi	Mahalat	Female	0.47	0.00	1.20
**244**	Current daily cigarette smoke	Mazandaran	Mahmudabad	Female	0.15	0.00	4.70
**245**	Current daily cigarette smoke	Zanjan	Mahneshan	Female	0.50	0.00	6.08
**246**	Current daily cigarette smoke	Khorasan_razavi	Mahvelat	Female	0.43	0.00	4.13
**247**	Current daily cigarette smoke	Azarbayjan_West	Maku	Female	1.74	0.00	9.30
**248**	Current daily cigarette smoke	Tehran	Malard	Female	0.34	0.00	3.63
**249**	Current daily cigarette smoke	Hamedan	Malayer	Female	0.80	0.00	5.83
**250**	Current daily cigarette smoke	Azararbayjan_East	Malekan	Female	0.25	0.00	4.20
**251**	Current daily cigarette smoke	Ilam	Malekshahi	Female	0.38	0.00	4.96
**252**	Current daily cigarette smoke	Fars	Mamasany	Female	0.42	0.00	6.56
**253**	Current daily cigarette smoke	Khorasan_North	Maneh and Samalqan	Female	0.32	0.00	6.29
**254**	Current daily cigarette smoke	Kerman	Manujan	Female	0.32	0.00	2.18
**255**	Current daily cigarette smoke	Azararbayjan_East	Maragheh	Female	0.35	0.00	4.00
**256**	Current daily cigarette smoke	Azararbayjan_East	Marand	Female	0.43	0.00	4.31
**257**	Current daily cigarette smoke	Golestan	Maravehtapeh	Female	0.42	0.00	9.73
**258**	Current daily cigarette smoke	Kordestan	Marivan	Female	0.79	0.00	4.63
**259**	Current daily cigarette smoke	Fars	Marvdasht	Female	0.40	0.00	6.56
**260**	Current daily cigarette smoke	Gilan	Masal	Female	0.28	0.00	4.55
**261**	Current daily cigarette smoke	Khorasan_razavi	Mashhad	Female	0.60	0.00	3.37
**262**	Current daily cigarette smoke	Khuzestan	Masjed Soleyman	Female	0.33	0.00	4.49
**263**	Current daily cigarette smoke	Semnan	Mayamey	Female	0.49	0.00	5.08
**264**	Current daily cigarette smoke	Semnan	Mehdishahr	Female	0.52	0.00	4.91
**265**	Current daily cigarette smoke	Ilam	Mehran	Female	0.22	0.00	3.64
**266**	Current daily cigarette smoke	Yazd	Mehriz	Female	0.80	0.00	9.59
**267**	Current daily cigarette smoke	Ardebil	Meshkinshahr	Female	0.36	0.00	3.37
**268**	Current daily cigarette smoke	Yazd	Meybod	Female	0.41	0.00	6.89
**269**	Current daily cigarette smoke	Hormozgan	Minab	Female	0.28	0.00	4.27
**270**	Current daily cigarette smoke	Golestan	Minudasht	Female	0.19	0.00	6.42
**271**	Current daily cigarette smoke	Sistan and Balouchestan	Mirjaveh	Female	2.19	0.00	17.66
**272**	Current daily cigarette smoke	Azarbayjan_West	Miyandoab	Female	0.27	0.00	5.49
**273**	Current daily cigarette smoke	Mazandaran	Miyandorud	Female	0.48	0.00	7.76
**274**	Current daily cigarette smoke	Azararbayjan_East	Miyaneh	Female	0.49	0.00	4.21
**275**	Current daily cigarette smoke	Isfahan	Mobarakeh	Female	1.44	0.00	9.47
**276**	Current daily cigarette smoke	Fars	Mohr	Female	0.84	0.00	9.50
**277**	Current daily cigarette smoke	Hamedan	Nahavand	Female	0.46	0.00	5.62
**278**	Current daily cigarette smoke	Isfahan	Najafabad	Female	1.43	0.00	9.25
**279**	Current daily cigarette smoke	Ardebil	Namin	Female	0.44	0.00	5.43
**280**	Current daily cigarette smoke	Azarbayjan_West	Naqadeh	Female	0.40	0.00	5.85
**281**	Current daily cigarette smoke	Kerman	Narmashir	Female	0.34	0.00	2.25
**282**	Current daily cigarette smoke	Isfahan	Natanz	Female	0.23	0.00	6.44
**283**	Current daily cigarette smoke	Isfahan	Nayin	Female	0.45	0.00	9.13
**284**	Current daily cigarette smoke	Alborz	Nazarabad	Female	0.17	0.00	3.56
**285**	Current daily cigarette smoke	Ardebil	Neer	Female	0.47	0.00	5.00
**286**	Current daily cigarette smoke	Khorasan_South	Nehbandan	Female	0.72	0.00	7.19
**287**	Current daily cigarette smoke	Mazandaran	Neka	Female	0.18	0.00	4.65
**288**	Current daily cigarette smoke	Fars	Neyriz	Female	1.71	0.00	9.58
**289**	Current daily cigarette smoke	Khorasan_razavi	Neyshabur	Female	0.23	0.00	2.89

**290**	Current daily cigarette smoke	Sistan and Balouchestan	Nikshahr	Female	1.10	0.00	10.67
**291**	Current daily cigarette smoke	Sistan and Balouchestan	Nimruz	Female	2.23	0.00	16.50
**292**	Current daily cigarette smoke	Mazandaran	Noshahr	Female	0.14	0.00	4.86
**293**	Current daily cigarette smoke	Mazandaran	Nur	Female	0.23	0.00	4.57
**294**	Current daily cigarette smoke	Khuzestan	Omidiyeh	Female	0.14	0.00	4.27
**295**	Current daily cigarette smoke	Azarbayjan_West	Orumiyeh	Female	2.07	0.00	8.82
**296**	Current daily cigarette smoke	Azarbayjan_West	Oshnaviyeh	Female	0.87	0.00	8.93
**297**	Current daily cigarette smoke	Azararbayjan_East	Osku	Female	0.69	0.00	5.94
**298**	Current daily cigarette smoke	Tehran	Pakdasht	Female	0.35	0.00	3.52
**299**	Current daily cigarette smoke	Tehran	Pardis	Female	0.57	0.00	5.01
**300**	Current daily cigarette smoke	Ardebil	Parsabad	Female	0.23	0.00	3.54
**301**	Current daily cigarette smoke	Hormozgan	Parsian (Gavbandi)	Female	0.87	0.00	6.82
**302**	Current daily cigarette smoke	Fars	Pasargad	Female	0.74	0.00	9.69
**303**	Current daily cigarette smoke	Kermanshah	Paveh	Female	0.82	0.00	7.22
**304**	Current daily cigarette smoke	Azarbayjan_West	Piranshahr	Female	0.74	0.00	8.99
**305**	Current daily cigarette smoke	Tehran	Pishva	Female	0.31	0.00	3.79
**306**	Current daily cigarette smoke	Azarbayjan_West	Poldasht	Female	0.43	0.00	5.81
**307**	Current daily cigarette smoke	Lorestan	Poldokhtar	Female	0.05	0.00	1.32
**308**	Current daily cigarette smoke	Mazandaran	Qaemshahr	Female	0.27	0.00	4.76
**309**	Current daily cigarette smoke	Tehran	Qarchak	Female	0.62	0.00	5.41
**310**	Current daily cigarette smoke	Sistan and Balouchestan	Qasr qand	Female	1.87	0.00	16.42
**311**	Current daily cigarette smoke	Kermanshah	Qasr-e-Shirin	Female	0.86	0.00	7.96
**312**	Current daily cigarette smoke	Khorasan_South	Qayenat	Female	0.67	0.00	7.03
**313**	Current daily cigarette smoke	Qazvin	Qazvin	Female	0.88	0.00	4.17
**314**	Current daily cigarette smoke	Hormozgan	Qeshm	Female	0.44	0.00	4.52
**315**	Current daily cigarette smoke	Fars	Qirokarzin	Female	0.41	0.00	6.76
**316**	Current daily cigarette smoke	Qom	Qom	Female	0.81	0.00	12.93
**317**	Current daily cigarette smoke	Kordestan	Qorveh	Female	0.25	0.00	3.43
**318**	Current daily cigarette smoke	Khorasan_razavi	Quchan	Female	0.75	0.00	3.86
**319**	Current daily cigarette smoke	Kerman	Rabar	Female	0.54	0.00	2.37
**320**	Current daily cigarette smoke	Kerman	Rafsanjan	Female	0.30	0.00	1.55
**321**	Current daily cigarette smoke	Khuzestan	Ramhormoz	Female	0.11	0.00	3.09
**322**	Current daily cigarette smoke	Mazandaran	Ramsar	Female	0.36	0.00	7.01
**323**	Current daily cigarette smoke	Khuzestan	Ramshir	Female	0.24	0.00	4.50
**324**	Current daily cigarette smoke	Golestan	Ramyan	Female	0.40	0.00	9.07
**325**	Current daily cigarette smoke	Gilan	Rasht	Female	0.17	0.00	2.92
**326**	Current daily cigarette smoke	Khorasan_razavi	Rashtkhar	Female	0.38	0.00	4.28
**327**	Current daily cigarette smoke	Kermanshah	Ravansar	Female	0.94	0.00	7.15
**328**	Current daily cigarette smoke	Kerman	Ravar	Female	0.20	0.00	1.56
**329**	Current daily cigarette smoke	Khorasan_North	Raz va Jergolan	Female	0.50	0.00	10.08
**330**	Current daily cigarette smoke	Hamedan	Razan	Female	0.00	0.00	4.93
**331**	Current daily cigarette smoke	Tehran	Rey	Female	0.35	0.00	3.54
**332**	Current daily cigarette smoke	Kerman	Reygan	Female	0.32	0.00	2.06
**333**	Current daily cigarette smoke	Gilan	Rezvanshahr	Female	0.33	0.00	4.29
**334**	Current daily cigarette smoke	Tehran	Robatkarim	Female	0.37	0.00	3.66
**335**	Current daily cigarette smoke	Fars	Rostam	Female	0.45	0.00	6.99
**336**	Current daily cigarette smoke	Kerman	Roudbar-e-Jonub	Female	0.19	0.00	1.50
**337**	Current daily cigarette smoke	Hormozgan	Rudan	Female	0.35	0.00	4.14
**338**	Current daily cigarette smoke	Gilan	Rudbar	Female	0.12	0.00	2.89
**339**	Current daily cigarette smoke	Gilan	Rudsar	Female	0.14	0.00	3.05
**340**	Current daily cigarette smoke	Lorestan	Rumshekan	Female	0.05	0.00	1.92
**341**	Current daily cigarette smoke	Khorasan_razavi	Sabzevar	Female	0.19	0.00	2.97
**342**	Current daily cigarette smoke	Yazd	Sadugh	Female	0.40	0.00	6.62
**343**	Current daily cigarette smoke	Kermanshah	Sahneh	Female	1.11	0.00	7.04
**344**	Current daily cigarette smoke	Kermanshah	Salas-e-Babajani	Female	0.96	0.00	6.99
**345**	Current daily cigarette smoke	Azarbayjan_West	Salmas	Female	0.43	0.00	6.01
**346**	Current daily cigarette smoke	Chaharmahal	Saman	Female	0.22	0.00	3.11
**347**	Current daily cigarette smoke	Kordestan	Sanandaj	Female	0.24	0.00	3.33
**348**	Current daily cigarette smoke	Kordestan	Saqqez	Female	0.13	0.00	3.19
**349**	Current daily cigarette smoke	Kermanshah	Sar-e-Pol-e-Zohab	Female	0.52	0.00	5.01
**350**	Current daily cigarette smoke	Azararbayjan_East	Sarab	Female	1.51	0.00	6.33
**351**	Current daily cigarette smoke	Khorasan_razavi	Sarakhs	Female	0.46	0.00	4.67
**352**	Current daily cigarette smoke	Sistan and Balouchestan	Saravan	Female	4.14	0.00	19.74
**353**	Current daily cigarette smoke	Khorasan_South	Sarayan	Female	1.14	0.00	10.18
**354**	Current daily cigarette smoke	Sistan and Balouchestan	Sarbaz	Female	1.22	0.00	11.09
**355**	Current daily cigarette smoke	Khorasan_South	Sarbisheh	Female	1.91	0.00	11.11
**356**	Current daily cigarette smoke	Azarbayjan_West	Sardasht	Female	0.37	0.00	5.83
**357**	Current daily cigarette smoke	Ardebil	Sarein	Female	0.56	0.00	5.25
**358**	Current daily cigarette smoke	Mazandaran	Sari	Female	1.00	0.00	5.89
**359**	Current daily cigarette smoke	Kordestan	Sarvabad	Female	0.38	0.00	4.86
**360**	Current daily cigarette smoke	Fars	Sarvestan	Female	0.80	0.00	9.78
**361**	Current daily cigarette smoke	Mazandaran	Savadkuh	Female	0.53	0.00	6.61
**362**	Current daily cigarette smoke	Mazandaran	Savadkuh_North	Female	0.52	0.00	7.09
**363**	Current daily cigarette smoke	Markazi	Saveh	Female	0.46	0.00	1.17
**364**	Current daily cigarette smoke	Alborz	Savojbolagh	Female	0.20	0.00	3.41
**365**	Current daily cigarette smoke	Lorestan	Selseleh	Female	0.16	0.00	1.95
**366**	Current daily cigarette smoke	Isfahan	Semirom	Female	0.48	0.00	6.60
**367**	Current daily cigarette smoke	Isfahan	Semirom-e-Sofla	Female	0.62	0.00	9.52
**368**	Current daily cigarette smoke	Semnan	Semnan	Female	1.03	0.00	5.25
**369**	Current daily cigarette smoke	Fars	Sepidan	Female	0.49	0.00	6.74
**370**	Current daily cigarette smoke	Azararbayjan_East	Shabestar	Female	0.45	0.00	4.13
**371**	Current daily cigarette smoke	Khuzestan	Shadegan	Female	0.09	0.00	3.11
**372**	Current daily cigarette smoke	Gilan	Shaft	Female	0.12	0.00	3.03
**373**	Current daily cigarette smoke	Azarbayjan_West	Shahindezh	Female	0.31	0.00	5.61
**374**	Current daily cigarette smoke	Tehran	Shahr-e Qods	Female	1.38	0.00	5.38
**375**	Current daily cigarette smoke	Kerman	Shahr-e-Babak	Female	0.27	0.00	1.58
**376**	Current daily cigarette smoke	Chaharmahal	Shahr-e-Kord	Female	0.13	0.00	2.18
**377**	Current daily cigarette smoke	Isfahan	Shahreza	Female	0.39	0.00	6.47
**378**	Current daily cigarette smoke	Tehran	Shahriyar	Female	0.43	0.00	3.62
**379**	Current daily cigarette smoke	Semnan	Shahrud	Female	0.28	0.00	3.50
**380**	Current daily cigarette smoke	Markazi	Shazand	Female	3.19	2.30	4.07
**381**	Current daily cigarette smoke	Tehran	Shemiranat	Female	0.89	0.00	5.14
**382**	Current daily cigarette smoke	Fars	Shiraz	Female	2.13	0.00	9.37
**383**	Current daily cigarette smoke	Khorasan_North	Shirvan	Female	0.97	0.00	7.34
**384**	Current daily cigarette smoke	Ilam	Shirvan and Chard-e-Aval	Female	0.25	0.00	3.44
**385**	Current daily cigarette smoke	Azarbayjan_West	Showt	Female	0.49	0.00	6.28
**386**	Current daily cigarette smoke	Khuzestan	Shush	Female	0.18	0.00	3.14
**387**	Current daily cigarette smoke	Khuzestan	Shushtar	Female	0.76	0.00	4.65

**388**	Current daily cigarette smoke	Gilan	Siahkal	Female	0.19	0.00	4.02
**389**	Current daily cigarette smoke	Sistan and Balouchestan	Sib o Soran	Female	2.52	0.00	17.72
**390**	Current daily cigarette smoke	Mazandaran	Simorgh	Female	0.38	0.00	6.85
**391**	Current daily cigarette smoke	Hormozgan	Sirik	Female	0.54	0.00	6.84
**392**	Current daily cigarette smoke	Kerman	Sirjan	Female	0.50	0.00	1.82
**393**	Current daily cigarette smoke	Ilam	Sirvan	Female	0.35	0.00	4.78
**394**	Current daily cigarette smoke	Zanjan	Soltaniyeh	Female	0.43	0.00	5.84
**395**	Current daily cigarette smoke	Kermanshah	Sonqor	Female	1.25	0.00	7.19
**396**	Current daily cigarette smoke	Semnan	Sorkheh	Female	0.45	0.00	4.99
**397**	Current daily cigarette smoke	Gilan	Sume'eh Sara	Female	0.20	0.00	3.07
**398**	Current daily cigarette smoke	Khorasan_South	Tabas	Female	0.69	0.00	6.70
**399**	Current daily cigarette smoke	Azararbayjan_East	Tabriz	Female	1.32	0.00	5.48
**400**	Current daily cigarette smoke	Markazi	Tafresh	Female	0.76	0.00	1.78
**401**	Current daily cigarette smoke	Yazd	Taft	Female	1.24	0.00	9.62
**402**	Current daily cigarette smoke	Azarbayjan_West	Takab	Female	0.36	0.00	5.72
**403**	Current daily cigarette smoke	Qazvin	Takestan	Female	0.31	0.00	3.29
**404**	Current daily cigarette smoke	Khorasan_razavi	Takht-e-Jolgeh (Firuzeh)	Female	0.32	0.00	4.64
**405**	Current daily cigarette smoke	Alborz	Taleghan	Female	0.22	0.00	3.52
**406**	Current daily cigarette smoke	Boushehr	Tangestan	Female	3.46	0.00	14.83
**407**	Current daily cigarette smoke	Zanjan	Tarom	Female	0.20	0.00	3.89
**408**	Current daily cigarette smoke	Gilan	Tavalesh	Female	0.22	0.00	3.23
**409**	Current daily cigarette smoke	Khorasan_razavi	Taybad	Female	0.20	0.00	3.04
**410**	Current daily cigarette smoke	Tehran	Tehran	Female	1.45	0.00	4.75
**411**	Current daily cigarette smoke	Isfahan	Tiran and Karvan	Female	0.63	0.00	9.62
**412**	Current daily cigarette smoke	Mazandaran	Tonekabon	Female	0.24	0.00	4.70
**413**	Current daily cigarette smoke	Khorasan_razavi	Torbat-e-Heydariyeh	Female	0.86	0.00	4.08
**414**	Current daily cigarette smoke	Khorasan_razavi	Torbat-e-Jam	Female	0.76	0.00	3.97
**415**	Current daily cigarette smoke	Hamedan	Tuyserkan	Female	0.35	0.00	5.42
**416**	Current daily cigarette smoke	Tehran	Varamin	Female	0.91	0.00	4.39
**417**	Current daily cigarette smoke	Azararbayjan_East	Varzaqan	Female	0.92	0.00	5.95
**418**	Current daily cigarette smoke	Yazd	Yazd	Female	0.89	0.00	7.20
**419**	Current daily cigarette smoke	Sistan and Balouchestan	Zabol	Female	1.21	0.00	11.64
**420**	Current daily cigarette smoke	Sistan and Balouchestan	Zaboli (Mehrestan)	Female	1.95	0.00	15.45
**421**	Current daily cigarette smoke	Sistan and Balouchestan	Zahedan	Female	3.40	0.00	15.83
**422**	Current daily cigarette smoke	Zanjan	Zanjan	Female	0.98	0.00	5.56
**423**	Current daily cigarette smoke	Kerman	Zarand	Female	0.58	0.00	2.26
**424**	Current daily cigarette smoke	Markazi	Zarandiyeh	Female	0.76	0.00	1.71
**425**	Current daily cigarette smoke	Fars	Zarrindasht	Female	0.72	0.00	9.63
**426**	Current daily cigarette smoke	Khorasan_razavi	Zave	Female	0.41	0.00	4.17
**427**	Current daily cigarette smoke	Sistan and Balouchestan	Zehak	Female	2.12	0.00	18.53
**428**	Current daily cigarette smoke	Khorasan_South	Zir kuh	Female	1.29	0.00	10.66
**429**	Current daily cigarette smoke	Khuzestan	Abadan	Male	20.81	2.11	39.54
**430**	Current daily cigarette smoke	Fars	Abadeh	Male	29.09	18.24	39.82
**431**	Current daily cigarette smoke	Yazd	Abarkuh	Male	20.46	7.94	33.38
**432**	Current daily cigarette smoke	Mazandaran	Abbas abad	Male	40.35	26.28	51.84
**433**	Current daily cigarette smoke	Ilam	Abdanan	Male	11.09	0.21	22.71
**434**	Current daily cigarette smoke	Zanjan	Abhar	Male	16.96	6.07	27.77
**435**	Current daily cigarette smoke	Hormozgan	Abumusa	Male	13.37	0.00	35.15
**436**	Current daily cigarette smoke	Qazvin	Abyek	Male	25.55	9.73	42.09
**437**	Current daily cigarette smoke	Azararbayjan_East	Ahar	Male	23.14	9.36	37.50
**438**	Current daily cigarette smoke	Khuzestan	Ahvaz	Male	20.21	7.65	33.25
**439**	Current daily cigarette smoke	Azararbayjan_East	Ajabshir	Male	22.70	7.58	37.74
**440**	Current daily cigarette smoke	Qazvin	Alborz	Male	24.47	9.15	40.43
**441**	Current daily cigarette smoke	Golestan	Aliabad	Male	10.93	0.00	23.75
**442**	Current daily cigarette smoke	Lorestan	Aligudarz	Male	16.44	3.42	28.79
**443**	Current daily cigarette smoke	Gilan	Amlash	Male	22.82	5.47	40.34
**444**	Current daily cigarette smoke	Mazandaran	Amol	Male	19.37	6.52	31.96
**445**	Current daily cigarette smoke	Kerman	Anar	Male	26.83	17.04	35.23
**446**	Current daily cigarette smoke	Kerman	Anbarabad	Male	11.25	1.00	20.87
**447**	Current daily cigarette smoke	Khuzestan	Andika	Male	6.90	0.00	19.07
**448**	Current daily cigarette smoke	Khuzestan	Andimeshk	Male	13.34	0.00	26.23
**449**	Current daily cigarette smoke	Golestan	Aq Qala	Male	10.46	0.00	23.84
**450**	Current daily cigarette smoke	Khuzestan	Aqajari	Male	16.90	0.00	38.09
**451**	Current daily cigarette smoke	Semnan	Aradan	Male	22.30	3.45	41.34
**452**	Current daily cigarette smoke	Markazi	Arak	Male	26.90	11.96	42.41
**453**	Current daily cigarette smoke	Isfahan	Aran and Bidgol	Male	19.06	6.34	32.00
**454**	Current daily cigarette smoke	Ardebil	Ardabil	Male	20.66	8.71	32.32
**455**	Current daily cigarette smoke	Yazd	Ardakan	Male	13.08	3.09	23.03
**456**	Current daily cigarette smoke	Chaharmahal	Ardal	Male	21.48	5.06	38.13
**457**	Current daily cigarette smoke	Isfahan	Ardestan	Male	19.14	4.68	33.52
**458**	Current daily cigarette smoke	Fars	Arsanjan	Male	34.55	21.99	45.25
**459**	Current daily cigarette smoke	Kerman	Arzouyeh	Male	16.57	5.59	28.30
**460**	Current daily cigarette smoke	Hamedan	Asadabad	Male	18.27	4.44	32.20
**461**	Current daily cigarette smoke	Boushehr	Asaluyeh	Male	12.05	0.00	28.17
**462**	Current daily cigarette smoke	Markazi	Ashtiyan	Male	25.80	7.26	45.46
**463**	Current daily cigarette smoke	Gilan	Astaneh-ye-Ashrafiyeh	Male	25.37	9.92	41.63
**464**	Current daily cigarette smoke	Gilan	Astara	Male	16.30	0.00	32.88
**465**	Current daily cigarette smoke	Qazvin	Avaj	Male	22.35	6.43	37.34
**466**	Current daily cigarette smoke	Golestan	Azadshahr	Male	9.84	0.00	22.49
**467**	Current daily cigarette smoke	Azararbayjan_East	Azarshahr	Male	22.82	5.98	40.58
**468**	Current daily cigarette smoke	Lorestan	Azna	Male	20.53	3.83	36.99
**469**	Current daily cigarette smoke	Mazandaran	Babol	Male	18.90	6.86	30.78
**470**	Current daily cigarette smoke	Mazandaran	Babolsar	Male	20.14	3.03	37.20
**471**	Current daily cigarette smoke	Ilam	Badreh	Male	11.51	0.00	26.77
**472**	Current daily cigarette smoke	Yazd	Bafq	Male	12.85	4.90	21.21
**473**	Current daily cigarette smoke	Kerman	Baft	Male	17.13	6.83	27.65
**474**	Current daily cigarette smoke	Khuzestan	Baghemalek	Male	7.26	0.00	19.69
**475**	Current daily cigarette smoke	Yazd	Bahabad	Male	13.83	0.57	27.55
**476**	Current daily cigarette smoke	Hamedan	Bahar	Male	24.78	10.79	39.57
**477**	Current daily cigarette smoke	Tehran	Baharestan (Golestan)	Male	21.23	8.58	34.68
**478**	Current daily cigarette smoke	Kohkiluye and Bouyer Ahmad	Bahmani	Male	10.20	0.00	20.80
**479**	Current daily cigarette smoke	Khorasan_razavi	Bajestan	Male	13.94	0.00	27.75
**480**	Current daily cigarette smoke	Khorasan_razavi	Bakhraz	Male	11.89	0.00	30.52
**481**	Current daily cigarette smoke	Kerman	Bam	Male	13.68	3.80	23.62
**482**	Current daily cigarette smoke	Hormozgan	Bandar-e-Abbas	Male	14.59	2.94	26.21
**483**	Current daily cigarette smoke	Gilan	Bandar-e-Anzali	Male	21.24	6.51	35.94
**484**	Current daily cigarette smoke	Golestan	Bandar-e-Gaz	Male	9.49	0.00	25.49
**485**	Current daily cigarette smoke	Hormozgan	Bandar-e-Jask	Male	16.08	0.25	32.70

**486**	Current daily cigarette smoke	Hormozgan	Bandar-e-Lengeh	Male	12.94	0.00	26.58
**487**	Current daily cigarette smoke	Khuzestan	Bandar-e-Mahshahr	Male	20.60	3.80	37.63
**488**	Current daily cigarette smoke	Golestan	Bandar-e-Torkaman	Male	8.27	0.00	19.82
**489**	Current daily cigarette smoke	Kordestan	Baneh	Male	29.46	7.71	51.83
**490**	Current daily cigarette smoke	Khorasan_razavi	Bardeskan	Male	12.45	0.00	25.75
**491**	Current daily cigarette smoke	Kerman	Bardsir	Male	19.73	8.84	31.54
**492**	Current daily cigarette smoke	Hormozgan	Bashagerd	Male	11.06	0.00	22.71
**493**	Current daily cigarette smoke	Kohkiluye and Bouyer Ahmad	Basht	Male	11.86	3.21	20.75
**494**	Current daily cigarette smoke	Hormozgan	Bastak	Male	12.86	0.00	29.56
**495**	Current daily cigarette smoke	Khuzestan	Bavi	Male	12.80	0.00	27.89
**496**	Current daily cigarette smoke	Khuzestan	Behbahan	Male	18.49	2.47	35.43
**497**	Current daily cigarette smoke	Mazandaran	Behshahr	Male	7.35	0.00	19.28
**498**	Current daily cigarette smoke	Kordestan	Bijar	Male	21.32	7.08	35.28
**499**	Current daily cigarette smoke	Ardebil	Bilehsavar	Male	24.24	4.80	43.57
**500**	Current daily cigarette smoke	Khorasan_razavi	Binaloud	Male	13.48	0.00	33.60
**501**	Current daily cigarette smoke	Khorasan_South	Birjand	Male	9.69	0.00	20.35
**502**	Current daily cigarette smoke	Khorasan_North	Bojnurd	Male	8.59	0.00	19.15
**503**	Current daily cigarette smoke	Chaharmahal	Bon	Male	23.30	2.55	44.24
**504**	Current daily cigarette smoke	Azararbayjan_East	Bonab	Male	23.20	9.44	37.04
**505**	Current daily cigarette smoke	Isfahan	Borkhar	Male	20.42	4.89	36.17
**506**	Current daily cigarette smoke	Isfahan	Borkhar and Meymeh	Male	20.37	9.04	31.99
**507**	Current daily cigarette smoke	Chaharmahal	Borujen	Male	24.75	10.34	39.36
**508**	Current daily cigarette smoke	Lorestan	Borujerd	Male	22.55	7.50	37.74
**509**	Current daily cigarette smoke	Khorasan_South	Boshruyeh	Male	8.99	0.00	21.96
**510**	Current daily cigarette smoke	Azararbayjan_East	Bostanabad	Male	23.57	10.58	37.79
**511**	Current daily cigarette smoke	Fars	Bovanat	Male	19.11	7.37	30.50
**512**	Current daily cigarette smoke	Kohkiluye and Bouyer Ahmad	Boyer Ahmad	Male	17.11	6.21	28.12
**513**	Current daily cigarette smoke	Qazvin	Boyinzahra	Male	26.57	12.01	41.44
**514**	Current daily cigarette smoke	Isfahan	Buein va Miandasht	Male	19.66	4.22	36.08
**515**	Current daily cigarette smoke	Azarbayjan_West	Bukan	Male	22.97	8.03	37.34
**516**	Current daily cigarette smoke	Boushehr	Bushehr	Male	15.40	0.15	30.88
**517**	Current daily cigarette smoke	Isfahan	Chadegan	Male	20.49	6.14	35.06
**518**	Current daily cigarette smoke	Sistan and Balouchestan	Chahbahar	Male	12.02	0.12	23.66
**519**	Current daily cigarette smoke	Azarbayjan_West	Chaipareh	Male	27.16	9.13	45.90
**520**	Current daily cigarette smoke	Azarbayjan_West	Chaldoran	Male	27.62	6.24	48.34
**521**	Current daily cigarette smoke	Mazandaran	Chalus	Male	26.73	12.13	41.61
**522**	Current daily cigarette smoke	Azararbayjan_East	Charoimaq	Male	20.95	7.64	34.64
**523**	Current daily cigarette smoke	Khorasan_razavi	Chenaran	Male	12.61	0.00	30.32
**524**	Current daily cigarette smoke	Kohkiluye and Bouyer Ahmad	Cheram	Male	19.71	10.25	28.45
**525**	Current daily cigarette smoke	Kermanshah	Dalaho	Male	14.67	0.00	34.51
**526**	Current daily cigarette smoke	Lorestan	Dalfan	Male	18.07	2.48	33.45
**527**	Current daily cigarette smoke	Sistan and Balouchestan	Dalgan	Male	9.35	0.00	20.54
**528**	Current daily cigarette smoke	Tehran	Damavand	Male	19.01	5.54	33.02
**529**	Current daily cigarette smoke	Semnan	Damghan	Male	19.28	2.63	35.95
**530**	Current daily cigarette smoke	Fars	Darab	Male	20.14	7.88	32.80
**531**	Current daily cigarette smoke	Khorasan_South	Darmian	Male	6.69	0.00	16.95
**532**	Current daily cigarette smoke	Khorasan_razavi	Darrehgaz	Male	4.62	0.00	17.47
**533**	Current daily cigarette smoke	Ilam	Darrehshahr	Male	8.75	0.00	21.18
**534**	Current daily cigarette smoke	Khuzestan	Dasht-e-Azadegan	Male	17.68	0.00	37.89
**535**	Current daily cigarette smoke	Boushehr	Dashtestan	Male	16.14	3.44	29.21
**536**	Current daily cigarette smoke	Boushehr	Dashti	Male	13.05	0.00	26.48
**537**	Current daily cigarette smoke	Khorasan_razavi	Davarzan	Male	14.01	0.00	32.78
**538**	Current daily cigarette smoke	Boushehr	Dayyer	Male	11.99	0.00	27.90
**539**	Current daily cigarette smoke	Kordestan	Dehgolan	Male	24.86	7.43	42.19
**540**	Current daily cigarette smoke	Ilam	Dehloran	Male	12.62	0.00	27.57
**541**	Current daily cigarette smoke	Markazi	Delijan	Male	29.38	16.38	42.19
**542**	Current daily cigarette smoke	Kohkiluye and Bouyer Ahmad	Dena	Male	17.95	3.82	32.37
**543**	Current daily cigarette smoke	Boushehr	Deylam	Male	16.09	0.00	32.09
**544**	Current daily cigarette smoke	Khuzestan	Dezful	Male	14.67	1.46	27.60
**545**	Current daily cigarette smoke	Kordestan	Divandarreh	Male	28.85	9.73	48.38
**546**	Current daily cigarette smoke	Lorestan	Dorud	Male	21.06	3.95	38.58
**547**	Current daily cigarette smoke	Lorestan	Doureh	Male	18.99	2.14	36.08
**548**	Current daily cigarette smoke	Fars	Eqlid	Male	24.02	11.24	37.27
**549**	Current daily cigarette smoke	Khorasan_North	Esfarayen	Male	7.85	0.00	17.63
**550**	Current daily cigarette smoke	Alborz	Eshtehard	Male	25.83	9.62	42.30
**551**	Current daily cigarette smoke	Kermanshah	Eslamabad-e-Gharb	Male	11.53	0.00	25.65
**552**	Current daily cigarette smoke	Tehran	Eslamshahr	Male	21.09	8.51	33.62
**553**	Current daily cigarette smoke	Fars	Estahban	Male	20.08	5.74	35.44
**554**	Current daily cigarette smoke	Ilam	Eyvan	Male	4.72	0.00	16.54
**555**	Current daily cigarette smoke	Kerman	Fahraj	Male	12.65	5.88	19.61
**556**	Current daily cigarette smoke	Isfahan	Falavarjan	Male	23.11	9.10	37.39
**557**	Current daily cigarette smoke	Hamedan	Famenin	Male	17.62	4.58	30.69
**558**	Current daily cigarette smoke	Markazi	Farahan	Male	24.97	6.38	44.82
**559**	Current daily cigarette smoke	Fars	Farashband	Male	15.68	2.22	29.87
**560**	Current daily cigarette smoke	Alborz	Fardis	Male	24.43	6.59	42.60
**561**	Current daily cigarette smoke	Isfahan	Faridan	Male	21.92	7.73	36.70
**562**	Current daily cigarette smoke	Khorasan_razavi	Fariman	Male	4.63	0.00	16.64
**563**	Current daily cigarette smoke	Khorasan_North	Faroj	Male	7.79	0.00	17.57
**564**	Current daily cigarette smoke	Chaharmahal	Farsan	Male	22.99	6.68	39.72
**565**	Current daily cigarette smoke	Kerman	Faryab	Male	13.24	1.46	24.79
**566**	Current daily cigarette smoke	Fars	Fasa	Male	18.35	6.51	30.49
**567**	Current daily cigarette smoke	Khorasan_South	Ferdows	Male	7.90	0.00	18.20
**568**	Current daily cigarette smoke	Mazandaran	Fereydunkenar	Male	16.60	2.51	31.00
**569**	Current daily cigarette smoke	Isfahan	Fereydunshahr	Male	18.14	4.36	31.90
**570**	Current daily cigarette smoke	Fars	Firozabad	Male	15.98	3.89	28.36
**571**	Current daily cigarette smoke	Tehran	Firuzkuh	Male	16.13	3.58	28.51
**572**	Current daily cigarette smoke	Sistan and Balouchestan	Fonuj	Male	9.59	0.00	22.24
**573**	Current daily cigarette smoke	Gilan	Fuman	Male	20.35	7.72	33.41
**574**	Current daily cigarette smoke	Kohkiluye and Bouyer Ahmad	Gachsaran	Male	13.48	2.29	24.94
**575**	Current daily cigarette smoke	Golestan	Galikesh	Male	10.16	0.00	23.38
**576**	Current daily cigarette smoke	Mazandaran	Galugah	Male	11.36	0.00	30.61
**577**	Current daily cigarette smoke	Semnan	Garmsar	Male	22.86	7.12	38.55
**578**	Current daily cigarette smoke	Boushehr	Genaveh	Male	15.76	0.58	31.01
**579**	Current daily cigarette smoke	Fars	Gerash	Male	14.22	0.00	30.81
**580**	Current daily cigarette smoke	Khorasan_North	Germeh	Male	10.53	0.00	27.15
**581**	Current daily cigarette smoke	Ardebil	Germi	Male	26.01	9.95	42.47
**582**	Current daily cigarette smoke	Kerman	Ghaleye-Ganj	Male	5.17	0.00	13.55
**583**	Current daily cigarette smoke	Kermanshah	Gilan-e-Gharb	Male	13.75	0.00	30.33

**584**	Current daily cigarette smoke	Isfahan	Golpayegan	Male	21.54	7.98	35.71
**585**	Current daily cigarette smoke	Golestan	Gomishan	Male	7.44	0.00	20.09
**586**	Current daily cigarette smoke	Khorasan_razavi	Gonabad	Male	12.48	0.00	29.75
**587**	Current daily cigarette smoke	Golestan	Gonbad-e-Kavus	Male	10.24	0.00	21.47
**588**	Current daily cigarette smoke	Golestan	Gorgan	Male	9.66	0.00	20.52
**589**	Current daily cigarette smoke	Khuzestan	Guotvand	Male	14.79	0.00	32.68
**590**	Current daily cigarette smoke	Khuzestan	Haftgol	Male	15.36	0.00	31.57
**591**	Current daily cigarette smoke	Hormozgan	Hajiabad	Male	18.41	4.07	32.33
**592**	Current daily cigarette smoke	Hamedan	Hamadan	Male	21.05	7.66	34.15
**593**	Current daily cigarette smoke	Khuzestan	Hamidiyeh	Male	17.37	0.00	37.75
**594**	Current daily cigarette smoke	Sistan and Balouchestan	Hamoon	Male	8.94	0.00	22.36
**595**	Current daily cigarette smoke	Azararbayjan_East	Haris	Male	22.21	8.52	36.00
**596**	Current daily cigarette smoke	Kermanshah	Harsin	Male	6.72	0.00	20.45
**597**	Current daily cigarette smoke	Azararbayjan_East	Hashtrud	Male	23.39	9.63	37.28
**598**	Current daily cigarette smoke	Khuzestan	Hendijan	Male	18.42	0.00	37.81
**599**	Current daily cigarette smoke	Sistan and Balouchestan	Hirmand	Male	7.73	0.00	23.04
**600**	Current daily cigarette smoke	Khuzestan	Hoveizeh	Male	17.53	0.00	39.24
**601**	Current daily cigarette smoke	Zanjan	Ijerod	Male	22.49	13.48	30.76
**602**	Current daily cigarette smoke	Ilam	Ilam	Male	10.14	0.00	22.43
**603**	Current daily cigarette smoke	Sistan and Balouchestan	Iranshahr	Male	11.90	2.24	21.50
**604**	Current daily cigarette smoke	Isfahan	Isfahan	Male	20.22	10.22	30.42
**605**	Current daily cigarette smoke	Khuzestan	Izeh	Male	17.06	2.07	31.95
**606**	Current daily cigarette smoke	Fars	Jahrom	Male	14.97	3.53	26.59
**607**	Current daily cigarette smoke	Khorasan_North	Jajarm	Male	9.86	0.00	24.05
**608**	Current daily cigarette smoke	Boushehr	Jam	Male	12.28	0.00	25.85
**609**	Current daily cigarette smoke	Kermanshah	Javanrud	Male	14.61	0.00	32.93
**610**	Current daily cigarette smoke	Kerman	Jiroft	Male	13.03	4.46	21.11
**611**	Current daily cigarette smoke	Khorasan_razavi	Joghatai	Male	12.88	0.00	31.24
**612**	Current daily cigarette smoke	Azararbayjan_East	Jolfa	Male	21.78	6.31	37.16
**613**	Current daily cigarette smoke	Khorasan_razavi	Jowayin	Male	13.79	0.00	32.43
**614**	Current daily cigarette smoke	Mazandaran	Juybar	Male	19.50	3.53	35.91
**615**	Current daily cigarette smoke	Hamedan	Kabudarahang	Male	20.14	6.11	34.03
**616**	Current daily cigarette smoke	Kerman	Kahnuj	Male	8.88	0.40	17.20
**617**	Current daily cigarette smoke	Golestan	Kalaleh	Male	8.88	0.00	21.33
**618**	Current daily cigarette smoke	Khorasan_razavi	Kalat	Male	9.78	0.00	30.99
**619**	Current daily cigarette smoke	Azararbayjan_East	Kaleibar	Male	22.15	6.15	38.38
**620**	Current daily cigarette smoke	Kordestan	Kamyaran	Male	23.08	4.50	41.87
**621**	Current daily cigarette smoke	Boushehr	Kangan	Male	11.65	0.00	26.27
**622**	Current daily cigarette smoke	Kermanshah	Kangavar	Male	21.47	6.01	37.30
**623**	Current daily cigarette smoke	Alborz	Karaj	Male	25.84	14.79	37.04
**624**	Current daily cigarette smoke	Khuzestan	Karun	Male	18.49	0.00	42.49
**625**	Current daily cigarette smoke	Isfahan	Kashan	Male	19.73	6.73	32.95
**626**	Current daily cigarette smoke	Khorasan_razavi	Kashmar	Male	11.49	0.00	24.29
**627**	Current daily cigarette smoke	Fars	Kavar	Male	16.04	2.88	28.92
**628**	Current daily cigarette smoke	Fars	Kazerun	Male	16.66	5.25	28.00
**629**	Current daily cigarette smoke	Mazandaran	Kelardasht	Male	27.75	10.45	45.96
**630**	Current daily cigarette smoke	Kerman	Kerman	Male	13.74	5.80	21.22
**631**	Current daily cigarette smoke	Kermanshah	Kermanshah	Male	18.46	5.49	31.96
**632**	Current daily cigarette smoke	Khorasan_razavi	Khaf	Male	5.82	0.00	17.49
**633**	Current daily cigarette smoke	Khorasan_razavi	Khalilabad	Male	16.16	0.00	34.00
**634**	Current daily cigarette smoke	Ardebil	Khalkhal	Male	23.39	9.15	37.90
**635**	Current daily cigarette smoke	Hormozgan	Khamir	Male	12.59	0.00	25.89
**636**	Current daily cigarette smoke	Isfahan	Khansar	Male	19.86	5.31	35.22
**637**	Current daily cigarette smoke	Sistan and Balouchestan	Khash	Male	22.62	12.68	31.36
**638**	Current daily cigarette smoke	Yazd	Khatam	Male	18.65	5.95	31.61
**639**	Current daily cigarette smoke	Fars	Kherameh	Male	18.97	7.08	30.11
**640**	Current daily cigarette smoke	Azararbayjan_East	Khodaafarin	Male	21.90	5.06	38.73
**641**	Current daily cigarette smoke	Zanjan	Khodabandeh	Male	17.00	6.08	28.17
**642**	Current daily cigarette smoke	Markazi	Khomeyn	Male	30.97	15.28	47.09
**643**	Current daily cigarette smoke	Isfahan	Khomeynishahr	Male	21.78	7.63	35.91
**644**	Current daily cigarette smoke	Markazi	Khondab	Male	22.03	5.25	38.05
**645**	Current daily cigarette smoke	Fars	Khonj	Male	15.29	1.09	29.52
**646**	Current daily cigarette smoke	Isfahan	Khoor va Biabanak	Male	15.29	0.00	30.34
**647**	Current daily cigarette smoke	Lorestan	Khorramabad	Male	18.42	4.98	31.72
**648**	Current daily cigarette smoke	Fars	Khorrambid	Male	25.95	11.42	41.37
**649**	Current daily cigarette smoke	Zanjan	Khorramdarreh	Male	9.42	0.00	20.83
**650**	Current daily cigarette smoke	Khuzestan	Khorramshahr	Male	23.00	4.38	42.14
**651**	Current daily cigarette smoke	Khorasan_razavi	Khoshab	Male	12.93	0.00	30.91
**652**	Current daily cigarette smoke	Azarbayjan_West	Khoy	Male	27.73	12.33	43.77
**653**	Current daily cigarette smoke	Khorasan_South	Khusef	Male	10.84	0.00	24.06
**654**	Current daily cigarette smoke	Chaharmahal	Kiaar	Male	24.13	7.25	40.53
**655**	Current daily cigarette smoke	Kohkiluye and Bouyer Ahmad	Kohgiluyeh	Male	14.96	3.21	26.38
**656**	Current daily cigarette smoke	Markazi	Komeijan	Male	24.50	5.96	42.68
**657**	Current daily cigarette smoke	Sistan and Balouchestan	Konarak	Male	12.10	0.00	24.68
**658**	Current daily cigarette smoke	Golestan	Kordkuy	Male	10.80	0.00	24.80
**659**	Current daily cigarette smoke	Ardebil	Kowsar	Male	23.22	7.76	39.17
**660**	Current daily cigarette smoke	Kerman	Kuhbonan	Male	13.27	0.40	25.06
**661**	Current daily cigarette smoke	Lorestan	Kuhdasht	Male	18.13	2.97	33.63
**662**	Current daily cigarette smoke	Chaharmahal	Kuhrang	Male	17.35	3.94	30.42
**663**	Current daily cigarette smoke	Gilan	Lahijan	Male	26.46	13.95	39.53
**664**	Current daily cigarette smoke	Khuzestan	Lali	Male	15.59	0.00	33.85
**665**	Current daily cigarette smoke	Fars	Lamard	Male	8.62	0.00	18.59
**666**	Current daily cigarette smoke	Kohkiluye and Bouyer Ahmad	Landeh	Male	13.35	0.00	31.50
**667**	Current daily cigarette smoke	Gilan	Langrud	Male	22.94	8.61	37.29
**668**	Current daily cigarette smoke	Isfahan	Lanjan	Male	22.26	8.95	35.85
**669**	Current daily cigarette smoke	Fars	Lar (Larestan)	Male	16.38	3.86	28.82
**670**	Current daily cigarette smoke	Chaharmahal	Lordakan	Male	23.26	9.98	36.77
**671**	Current daily cigarette smoke	Azarbayjan_West	Mahabad	Male	24.80	8.79	39.92
**672**	Current daily cigarette smoke	Markazi	Mahalat	Male	27.29	9.41	46.14
**673**	Current daily cigarette smoke	Mazandaran	Mahmudabad	Male	18.38	3.27	33.02
**674**	Current daily cigarette smoke	Zanjan	Mahneshan	Male	19.78	6.95	32.83
**675**	Current daily cigarette smoke	Khorasan_razavi	Mahvelat	Male	12.88	0.00	30.84
**676**	Current daily cigarette smoke	Azarbayjan_West	Maku	Male	27.54	7.66	48.09
**677**	Current daily cigarette smoke	Tehran	Malard	Male	22.30	9.52	35.50
**678**	Current daily cigarette smoke	Hamedan	Malayer	Male	20.94	6.68	35.60
**679**	Current daily cigarette smoke	Azararbayjan_East	Malekan	Male	20.87	6.95	34.88
**680**	Current daily cigarette smoke	Ilam	Malekshahi	Male	11.41	0.00	28.73
**681**	Current daily cigarette smoke	Fars	Mamasany	Male	17.61	5.89	29.25

**682**	Current daily cigarette smoke	Khorasan_North	Maneh and Samalqan	Male	11.66	0.00	25.12
**683**	Current daily cigarette smoke	Kerman	Manujan	Male	10.24	0.00	22.04
**684**	Current daily cigarette smoke	Azararbayjan_East	Maragheh	Male	22.79	10.70	34.71
**685**	Current daily cigarette smoke	Azararbayjan_East	Marand	Male	18.09	5.22	30.77
**686**	Current daily cigarette smoke	Golestan	Maravehtapeh	Male	10.97	0.00	28.03
**687**	Current daily cigarette smoke	Kordestan	Marivan	Male	27.33	7.76	48.29
**688**	Current daily cigarette smoke	Fars	Marvdasht	Male	21.82	10.14	33.70
**689**	Current daily cigarette smoke	Gilan	Masal	Male	21.30	7.22	36.09
**690**	Current daily cigarette smoke	Khorasan_razavi	Mashhad	Male	11.91	0.88	22.95
**691**	Current daily cigarette smoke	Khuzestan	Masjed Soleyman	Male	13.12	0.00	27.08
**692**	Current daily cigarette smoke	Semnan	Mayamey	Male	14.03	0.00	32.59
**693**	Current daily cigarette smoke	Semnan	Mehdishahr	Male	20.36	3.79	36.48
**694**	Current daily cigarette smoke	Ilam	Mehran	Male	11.51	0.00	30.55
**695**	Current daily cigarette smoke	Yazd	Mehriz	Male	17.25	6.28	28.30
**696**	Current daily cigarette smoke	Ardebil	Meshkinshahr	Male	25.09	10.01	40.37
**697**	Current daily cigarette smoke	Yazd	Meybod	Male	16.82	4.52	29.22
**698**	Current daily cigarette smoke	Hormozgan	Minab	Male	12.53	0.00	25.09
**699**	Current daily cigarette smoke	Golestan	Minudasht	Male	7.25	0.00	17.57
**700**	Current daily cigarette smoke	Sistan and Balouchestan	Mirjaveh	Male	14.51	0.00	30.18
**701**	Current daily cigarette smoke	Azarbayjan_West	Miyandoab	Male	25.34	11.56	39.45
**702**	Current daily cigarette smoke	Mazandaran	Miyandorud	Male	16.88	0.00	38.52
**703**	Current daily cigarette smoke	Azararbayjan_East	Miyaneh	Male	21.62	9.47	34.04
**704**	Current daily cigarette smoke	Isfahan	Mobarakeh	Male	22.06	8.13	37.18
**705**	Current daily cigarette smoke	Fars	Mohr	Male	13.94	0.00	28.91
**706**	Current daily cigarette smoke	Hamedan	Nahavand	Male	20.58	5.80	35.38
**707**	Current daily cigarette smoke	Isfahan	Najafabad	Male	21.92	8.48	35.54
**708**	Current daily cigarette smoke	Ardebil	Namin	Male	20.51	1.87	37.72
**709**	Current daily cigarette smoke	Azarbayjan_West	Naqadeh	Male	27.57	11.18	44.66
**710**	Current daily cigarette smoke	Kerman	Narmashir	Male	13.60	2.10	24.99
**711**	Current daily cigarette smoke	Isfahan	Natanz	Male	19.08	4.39	33.37
**712**	Current daily cigarette smoke	Isfahan	Nayin	Male	17.10	4.24	30.50
**713**	Current daily cigarette smoke	Alborz	Nazarabad	Male	27.39	9.81	45.76
**714**	Current daily cigarette smoke	Ardebil	Neer	Male	27.72	16.46	39.13
**715**	Current daily cigarette smoke	Khorasan_South	Nehbandan	Male	4.99	0.00	14.55
**716**	Current daily cigarette smoke	Mazandaran	Neka	Male	14.66	0.00	29.85
**717**	Current daily cigarette smoke	Fars	Neyriz	Male	20.74	9.32	32.14
**718**	Current daily cigarette smoke	Khorasan_razavi	Neyshabur	Male	11.39	0.00	22.67
**719**	Current daily cigarette smoke	Sistan and Balouchestan	Nikshahr	Male	8.77	0.00	17.30
**720**	Current daily cigarette smoke	Sistan and Balouchestan	Nimruz	Male	8.83	0.00	21.34
**721**	Current daily cigarette smoke	Mazandaran	Noshahr	Male	24.26	4.40	44.72
**722**	Current daily cigarette smoke	Mazandaran	Nur	Male	21.49	7.23	36.05
**723**	Current daily cigarette smoke	Khuzestan	Omidiyeh	Male	19.54	4.10	35.99
**724**	Current daily cigarette smoke	Azarbayjan_West	Orumiyeh	Male	28.95	14.24	43.89
**725**	Current daily cigarette smoke	Azarbayjan_West	Oshnaviyeh	Male	28.93	9.83	48.57
**726**	Current daily cigarette smoke	Azararbayjan_East	Osku	Male	20.74	7.33	34.33
**727**	Current daily cigarette smoke	Tehran	Pakdasht	Male	18.84	6.94	30.90
**728**	Current daily cigarette smoke	Tehran	Pardis	Male	19.02	3.05	35.46
**729**	Current daily cigarette smoke	Ardebil	Parsabad	Male	24.67	6.70	42.74
**730**	Current daily cigarette smoke	Hormozgan	Parsian (Gavbandi)	Male	12.97	0.00	26.41
**731**	Current daily cigarette smoke	Fars	Pasargad	Male	35.28	21.78	49.70
**732**	Current daily cigarette smoke	Kermanshah	Paveh	Male	20.22	0.00	40.94
**733**	Current daily cigarette smoke	Azarbayjan_West	Piranshahr	Male	28.01	9.75	47.22
**734**	Current daily cigarette smoke	Tehran	Pishva	Male	18.33	5.24	31.44
**735**	Current daily cigarette smoke	Azarbayjan_West	Poldasht	Male	27.48	11.02	44.35
**736**	Current daily cigarette smoke	Lorestan	Poldokhtar	Male	16.26	0.23	32.32
**737**	Current daily cigarette smoke	Mazandaran	Qaemshahr	Male	19.68	6.75	32.54
**738**	Current daily cigarette smoke	Tehran	Qarchak	Male	19.20	1.69	36.45
**739**	Current daily cigarette smoke	Sistan and Balouchestan	Qasr qand	Male	11.03	0.00	24.04
**740**	Current daily cigarette smoke	Kermanshah	Qasr-e-Shirin	Male	13.49	0.00	38.73
**741**	Current daily cigarette smoke	Khorasan_South	Qayenat	Male	9.06	0.00	20.49
**742**	Current daily cigarette smoke	Qazvin	Qazvin	Male	24.54	12.15	37.16
**743**	Current daily cigarette smoke	Hormozgan	Qeshm	Male	13.76	0.00	28.70
**744**	Current daily cigarette smoke	Fars	Qirokarzin	Male	15.86	0.68	30.49
**745**	Current daily cigarette smoke	Qom	Qom	Male	18.69	4.91	32.66
**746**	Current daily cigarette smoke	Kordestan	Qorveh	Male	16.07	2.40	29.84
**747**	Current daily cigarette smoke	Khorasan_razavi	Quchan	Male	12.39	0.00	28.76
**748**	Current daily cigarette smoke	Kerman	Rabar	Male	40.91	23.56	54.75
**749**	Current daily cigarette smoke	Kerman	Rafsanjan	Male	14.97	6.27	23.29
**750**	Current daily cigarette smoke	Khuzestan	Ramhormoz	Male	17.25	2.16	32.21
**751**	Current daily cigarette smoke	Mazandaran	Ramsar	Male	24.58	7.34	41.82
**752**	Current daily cigarette smoke	Khuzestan	Ramshir	Male	19.68	1.68	38.36
**753**	Current daily cigarette smoke	Golestan	Ramyan	Male	10.88	0.00	24.07
**754**	Current daily cigarette smoke	Gilan	Rasht	Male	21.82	11.09	32.26
**755**	Current daily cigarette smoke	Khorasan_razavi	Rashtkhar	Male	11.28	0.00	26.96
**756**	Current daily cigarette smoke	Kermanshah	Ravansar	Male	17.75	0.00	37.38
**757**	Current daily cigarette smoke	Kerman	Ravar	Male	16.97	9.08	24.60
**758**	Current daily cigarette smoke	Khorasan_North	Raz va Jergolan	Male	9.88	0.00	28.45
**759**	Current daily cigarette smoke	Hamedan	Razan	Male	22.98	7.71	38.92
**760**	Current daily cigarette smoke	Tehran	Rey	Male	19.80	8.03	31.97
**761**	Current daily cigarette smoke	Kerman	Reygan	Male	11.46	2.55	20.32
**762**	Current daily cigarette smoke	Gilan	Rezvanshahr	Male	20.66	6.44	34.55
**763**	Current daily cigarette smoke	Tehran	Robatkarim	Male	11.06	0.66	22.17
**764**	Current daily cigarette smoke	Fars	Rostam	Male	17.30	3.37	31.24
**765**	Current daily cigarette smoke	Kerman	Roudbar-e-Jonub	Male	10.49	0.74	19.95
**766**	Current daily cigarette smoke	Hormozgan	Rudan	Male	8.45	0.00	19.25
**767**	Current daily cigarette smoke	Gilan	Rudbar	Male	22.20	8.64	35.77
**768**	Current daily cigarette smoke	Gilan	Rudsar	Male	22.06	8.81	35.58
**769**	Current daily cigarette smoke	Lorestan	Rumshekan	Male	15.20	0.00	34.57
**770**	Current daily cigarette smoke	Khorasan_razavi	Sabzevar	Male	15.83	2.61	29.83
**771**	Current daily cigarette smoke	Yazd	Sadugh	Male	17.40	3.38	31.58
**772**	Current daily cigarette smoke	Kermanshah	Sahneh	Male	17.81	5.42	30.08
**773**	Current daily cigarette smoke	Kermanshah	Salas-e-Babajani	Male	14.95	0.00	31.41
**774**	Current daily cigarette smoke	Azarbayjan_West	Salmas	Male	25.62	9.44	41.26
**775**	Current daily cigarette smoke	Chaharmahal	Saman	Male	23.63	4.91	43.06
**776**	Current daily cigarette smoke	Kordestan	Sanandaj	Male	24.76	8.61	40.93
**777**	Current daily cigarette smoke	Kordestan	Saqqez	Male	29.20	11.11	47.74
**778**	Current daily cigarette smoke	Kermanshah	Sar-e-Pol-e-Zohab	Male	13.33	0.00	29.90
**779**	Current daily cigarette smoke	Azararbayjan_East	Sarab	Male	22.50	9.97	35.47

**780**	Current daily cigarette smoke	Khorasan_razavi	Sarakhs	Male	11.05	0.00	31.12
**781**	Current daily cigarette smoke	Sistan and Balouchestan	Saravan	Male	11.66	0.08	23.79
**782**	Current daily cigarette smoke	Khorasan_South	Sarayan	Male	9.48	0.00	22.50
**783**	Current daily cigarette smoke	Sistan and Balouchestan	Sarbaz	Male	10.08	0.14	19.94
**784**	Current daily cigarette smoke	Khorasan_South	Sarbisheh	Male	16.18	4.03	29.22
**785**	Current daily cigarette smoke	Azarbayjan_West	Sardasht	Male	26.66	9.32	44.77
**786**	Current daily cigarette smoke	Ardebil	Sarein	Male	24.44	8.29	41.75
**787**	Current daily cigarette smoke	Mazandaran	Sari	Male	19.39	5.67	33.04
**788**	Current daily cigarette smoke	Kordestan	Sarvabad	Male	23.66	1.53	45.80
**789**	Current daily cigarette smoke	Fars	Sarvestan	Male	17.99	3.38	32.48
**790**	Current daily cigarette smoke	Mazandaran	Savadkuh	Male	23.16	8.57	38.88
**791**	Current daily cigarette smoke	Mazandaran	Savadkuh_North	Male	19.60	2.73	37.17
**792**	Current daily cigarette smoke	Markazi	Saveh	Male	24.92	9.66	40.52
**793**	Current daily cigarette smoke	Alborz	Savojbolagh	Male	27.20	11.37	44.41
**794**	Current daily cigarette smoke	Lorestan	Selseleh	Male	19.61	2.21	36.44
**795**	Current daily cigarette smoke	Isfahan	Semirom	Male	21.21	8.70	33.74
**796**	Current daily cigarette smoke	Isfahan	Semirom-e-Sofla	Male	21.80	5.11	38.74
**797**	Current daily cigarette smoke	Semnan	Semnan	Male	19.30	3.37	35.48
**798**	Current daily cigarette smoke	Fars	Sepidan	Male	21.62	9.06	34.72
**799**	Current daily cigarette smoke	Azararbayjan_East	Shabestar	Male	16.23	4.69	27.49
**800**	Current daily cigarette smoke	Khuzestan	Shadegan	Male	17.92	2.50	33.55
**801**	Current daily cigarette smoke	Gilan	Shaft	Male	23.52	11.69	36.08
**802**	Current daily cigarette smoke	Azarbayjan_West	Shahindezh	Male	20.83	7.30	34.32
**803**	Current daily cigarette smoke	Tehran	Shahr-e Qods	Male	18.43	6.37	30.44
**804**	Current daily cigarette smoke	Kerman	Shahr-e-Babak	Male	15.02	6.81	22.96
**805**	Current daily cigarette smoke	Chaharmahal	Shahr-e-Kord	Male	24.39	10.67	38.44
**806**	Current daily cigarette smoke	Isfahan	Shahreza	Male	22.26	8.11	36.64
**807**	Current daily cigarette smoke	Tehran	Shahriyar	Male	21.70	9.60	34.03
**808**	Current daily cigarette smoke	Semnan	Shahrud	Male	17.80	3.55	32.81
**809**	Current daily cigarette smoke	Markazi	Shazand	Male	27.84	11.76	44.78
**810**	Current daily cigarette smoke	Tehran	Shemiranat	Male	18.05	4.93	30.89
**811**	Current daily cigarette smoke	Fars	Shiraz	Male	18.23	8.03	27.84
**812**	Current daily cigarette smoke	Khorasan_North	Shirvan	Male	8.08	0.00	19.05
**813**	Current daily cigarette smoke	Ilam	Shirvan and Chard-e-Aval	Male	12.16	0.00	25.61
**814**	Current daily cigarette smoke	Azarbayjan_West	Showt	Male	32.39	12.94	51.61
**815**	Current daily cigarette smoke	Khuzestan	Shush	Male	15.38	0.87	29.20
**816**	Current daily cigarette smoke	Khuzestan	Shushtar	Male	15.49	0.97	30.45
**817**	Current daily cigarette smoke	Gilan	Siahkal	Male	23.62	9.36	38.41
**818**	Current daily cigarette smoke	Sistan and Balouchestan	Sib o Soran	Male	11.50	0.47	22.05
**819**	Current daily cigarette smoke	Mazandaran	Simorgh	Male	19.36	1.54	37.34
**820**	Current daily cigarette smoke	Hormozgan	Sirik	Male	21.07	10.19	31.08
**821**	Current daily cigarette smoke	Kerman	Sirjan	Male	17.15	7.74	26.59
**822**	Current daily cigarette smoke	Ilam	Sirvan	Male	10.99	0.00	27.59
**823**	Current daily cigarette smoke	Zanjan	Soltaniyeh	Male	19.37	5.77	33.09
**824**	Current daily cigarette smoke	Kermanshah	Sonqor	Male	21.40	3.99	39.83
**825**	Current daily cigarette smoke	Semnan	Sorkheh	Male	21.20	2.79	39.32
**826**	Current daily cigarette smoke	Gilan	Sume'eh Sara	Male	21.21	8.42	33.48
**827**	Current daily cigarette smoke	Khorasan_South	Tabas	Male	8.40	0.00	17.15
**828**	Current daily cigarette smoke	Azararbayjan_East	Tabriz	Male	21.44	10.76	31.97
**829**	Current daily cigarette smoke	Markazi	Tafresh	Male	22.26	5.25	39.40
**830**	Current daily cigarette smoke	Yazd	Taft	Male	19.63	11.02	28.19
**831**	Current daily cigarette smoke	Azarbayjan_West	Takab	Male	24.10	7.98	40.75
**832**	Current daily cigarette smoke	Qazvin	Takestan	Male	24.02	7.39	40.27
**833**	Current daily cigarette smoke	Khorasan_razavi	Takht-e-Jolgeh (Firuzeh)	Male	18.85	5.41	32.70
**834**	Current daily cigarette smoke	Alborz	Taleghan	Male	29.64	14.56	45.16
**835**	Current daily cigarette smoke	Boushehr	Tangestan	Male	14.91	0.00	33.20
**836**	Current daily cigarette smoke	Zanjan	Tarom	Male	20.14	8.20	31.95
**837**	Current daily cigarette smoke	Gilan	Tavalesh	Male	16.31	5.19	27.28
**838**	Current daily cigarette smoke	Khorasan_razavi	Taybad	Male	14.79	0.00	34.31
**839**	Current daily cigarette smoke	Tehran	Tehran	Male	19.05	10.31	28.31
**840**	Current daily cigarette smoke	Isfahan	Tiran and Karvan	Male	21.51	6.02	37.42
**841**	Current daily cigarette smoke	Mazandaran	Tonekabon	Male	19.78	7.62	32.14
**842**	Current daily cigarette smoke	Khorasan_razavi	Torbat-e-Heydariyeh	Male	13.19	0.00	28.39
**843**	Current daily cigarette smoke	Khorasan_razavi	Torbat-e-Jam	Male	9.03	0.00	21.66
**844**	Current daily cigarette smoke	Hamedan	Tuyserkan	Male	19.23	5.82	32.10
**845**	Current daily cigarette smoke	Tehran	Varamin	Male	19.73	8.04	31.48
**846**	Current daily cigarette smoke	Azararbayjan_East	Varzaqan	Male	21.34	7.37	35.79
**847**	Current daily cigarette smoke	Yazd	Yazd	Male	14.94	5.49	24.18
**848**	Current daily cigarette smoke	Sistan and Balouchestan	Zabol	Male	5.63	0.00	15.23
**849**	Current daily cigarette smoke	Sistan and Balouchestan	Zaboli (Mehrestan)	Male	12.96	0.81	25.61
**850**	Current daily cigarette smoke	Sistan and Balouchestan	Zahedan	Male	11.40	2.83	19.74
**851**	Current daily cigarette smoke	Zanjan	Zanjan	Male	20.86	10.25	31.27
**852**	Current daily cigarette smoke	Kerman	Zarand	Male	8.31	0.13	16.86
**853**	Current daily cigarette smoke	Markazi	Zarandiyeh	Male	29.08	12.82	45.98
**854**	Current daily cigarette smoke	Fars	Zarrindasht	Male	17.90	3.18	32.38
**855**	Current daily cigarette smoke	Khorasan_razavi	Zave	Male	11.21	0.00	28.39
**856**	Current daily cigarette smoke	Sistan and Balouchestan	Zehak	Male	7.95	0.00	23.12
**857**	Current daily cigarette smoke	Khorasan_South	Zir kuh	Male	8.33	0.00	23.14
**858**	Current tobacco smoke	Khuzestan	Abadan	Female	2.59	0.00	8.22
**859**	Current tobacco smoke	Fars	Abadeh	Female	3.81	0.00	9.04
**860**	Current tobacco smoke	Yazd	Abarkuh	Female	11.07	6.41	15.52
**861**	Current tobacco smoke	Mazandaran	Abbas abad	Female	2.97	0.00	14.77
**862**	Current tobacco smoke	Ilam	Abdanan	Female	0.59	0.00	3.57
**863**	Current tobacco smoke	Zanjan	Abhar	Female	0.74	0.00	7.33
**864**	Current tobacco smoke	Hormozgan	Abumusa	Female	13.05	0.33	26.43
**865**	Current tobacco smoke	Qazvin	Abyek	Female	2.76	0.00	13.71
**866**	Current tobacco smoke	Azararbayjan_East	Ahar	Female	1.55	0.00	6.81
**867**	Current tobacco smoke	Khuzestan	Ahvaz	Female	1.60	0.00	5.41
**868**	Current tobacco smoke	Azararbayjan_East	Ajabshir	Female	0.59	0.00	7.30
**869**	Current tobacco smoke	Qazvin	Alborz	Female	3.76	0.00	14.57
**870**	Current tobacco smoke	Golestan	Aliabad	Female	1.05	0.00	6.21
**871**	Current tobacco smoke	Lorestan	Aligudarz	Female	2.24	0.00	9.25
**872**	Current tobacco smoke	Gilan	Amlash	Female	0.56	0.00	10.28
**873**	Current tobacco smoke	Mazandaran	Amol	Female	2.17	0.00	9.84
**874**	Current tobacco smoke	Kerman	Anar	Female	1.67	0.00	8.31
**875**	Current tobacco smoke	Kerman	Anbarabad	Female	1.14	0.00	5.37
**876**	Current tobacco smoke	Khuzestan	Andika	Female	1.00	0.00	6.05
**877**	Current tobacco smoke	Khuzestan	Andimeshk	Female	0.36	0.00	4.14

**878**	Current tobacco smoke	Golestan	Aq Qala	Female	0.96	0.00	6.18
**879**	Current tobacco smoke	Khuzestan	Aqajari	Female	1.96	0.00	7.95
**880**	Current tobacco smoke	Semnan	Aradan	Female	0.44	0.00	3.27
**881**	Current tobacco smoke	Markazi	Arak	Female	2.23	0.00	6.36
**882**	Current tobacco smoke	Isfahan	Aran and Bidgol	Female	1.02	0.00	5.45
**883**	Current tobacco smoke	Ardebil	Ardabil	Female	1.21	0.00	5.51
**884**	Current tobacco smoke	Yazd	Ardakan	Female	2.10	0.00	5.94
**885**	Current tobacco smoke	Chaharmahal	Ardal	Female	0.48	0.00	2.81
**886**	Current tobacco smoke	Isfahan	Ardestan	Female	2.23	0.00	8.43
**887**	Current tobacco smoke	Fars	Arsanjan	Female	7.46	0.13	14.94
**888**	Current tobacco smoke	Kerman	Arzouyeh	Female	2.47	0.00	8.62
**889**	Current tobacco smoke	Hamedan	Asadabad	Female	1.28	0.00	6.86
**890**	Current tobacco smoke	Boushehr	Asaluyeh	Female	16.07	4.11	29.16
**891**	Current tobacco smoke	Markazi	Ashtiyan	Female	3.35	0.00	9.47
**892**	Current tobacco smoke	Gilan	Astaneh-ye-Ashrafiyeh	Female	1.75	0.00	10.00
**893**	Current tobacco smoke	Gilan	Astara	Female	0.89	0.00	11.88
**894**	Current tobacco smoke	Qazvin	Avaj	Female	2.52	0.00	12.98
**895**	Current tobacco smoke	Golestan	Azadshahr	Female	2.02	0.00	9.48
**896**	Current tobacco smoke	Azararbayjan_East	Azarshahr	Female	0.31	0.00	5.36
**897**	Current tobacco smoke	Lorestan	Azna	Female	4.61	0.00	15.58
**898**	Current tobacco smoke	Mazandaran	Babol	Female	3.05	0.00	11.53
**899**	Current tobacco smoke	Mazandaran	Babolsar	Female	2.26	0.00	14.14
**900**	Current tobacco smoke	Ilam	Badreh	Female	0.47	0.00	3.37
**901**	Current tobacco smoke	Yazd	Bafq	Female	2.23	0.00	6.03
**902**	Current tobacco smoke	Kerman	Baft	Female	1.36	0.00	5.51
**903**	Current tobacco smoke	Khuzestan	Baghemalek	Female	0.80	0.00	4.33
**904**	Current tobacco smoke	Yazd	Bahabad	Female	3.65	0.00	9.19
**905**	Current tobacco smoke	Hamedan	Bahar	Female	2.44	0.00	9.14
**906**	Current tobacco smoke	Tehran	Baharestan (Golestan)	Female	1.92	0.00	7.40
**907**	Current tobacco smoke	Kohkiluye and Bouyer Ahmad	Bahmani	Female	10.22	2.92	17.73
**908**	Current tobacco smoke	Khorasan_razavi	Bajestan	Female	7.16	0.01	14.33
**909**	Current tobacco smoke	Khorasan_razavi	Bakhraz	Female	8.98	1.33	16.61
**910**	Current tobacco smoke	Kerman	Bam	Female	2.56	0.00	8.23
**911**	Current tobacco smoke	Hormozgan	Bandar-e-Abbas	Female	12.64	2.64	22.78
**912**	Current tobacco smoke	Gilan	Bandar-e-Anzali	Female	1.38	0.00	10.48
**913**	Current tobacco smoke	Golestan	Bandar-e-Gaz	Female	1.41	0.00	10.39
**914**	Current tobacco smoke	Hormozgan	Bandar-e-Jask	Female	11.59	0.00	23.69
**915**	Current tobacco smoke	Hormozgan	Bandar-e-Lengeh	Female	14.08	2.90	25.44
**916**	Current tobacco smoke	Khuzestan	Bandar-e-Mahshahr	Female	2.09	0.00	7.07
**917**	Current tobacco smoke	Golestan	Bandar-e-Torkaman	Female	1.56	0.00	9.42
**918**	Current tobacco smoke	Kordestan	Baneh	Female	0.46	0.00	6.51
**919**	Current tobacco smoke	Khorasan_razavi	Bardeskan	Female	6.81	0.00	13.96
**920**	Current tobacco smoke	Kerman	Bardsir	Female	4.59	0.19	8.95
**921**	Current tobacco smoke	Hormozgan	Bashagerd	Female	12.52	1.62	23.94
**922**	Current tobacco smoke	Kohkiluye and Bouyer Ahmad	Basht	Female	10.20	0.00	22.04
**923**	Current tobacco smoke	Hormozgan	Bastak	Female	13.69	2.66	25.35
**924**	Current tobacco smoke	Khuzestan	Bavi	Female	1.35	0.00	7.25
**925**	Current tobacco smoke	Khuzestan	Behbahan	Female	1.78	0.00	5.65
**926**	Current tobacco smoke	Mazandaran	Behshahr	Female	1.29	0.00	9.11
**927**	Current tobacco smoke	Kordestan	Bijar	Female	1.25	0.00	9.44
**928**	Current tobacco smoke	Ardebil	Bilehsavar	Female	0.64	0.00	7.53
**929**	Current tobacco smoke	Khorasan_razavi	Binaloud	Female	18.81	12.59	24.69
**930**	Current tobacco smoke	Khorasan_South	Birjand	Female	3.35	0.00	9.03
**931**	Current tobacco smoke	Khorasan_North	Bojnurd	Female	4.34	0.00	12.34
**932**	Current tobacco smoke	Chaharmahal	Bon	Female	0.74	0.00	4.65
**933**	Current tobacco smoke	Azararbayjan_East	Bonab	Female	0.36	0.00	4.91
**934**	Current tobacco smoke	Isfahan	Borkhar	Female	3.17	0.00	9.31
**935**	Current tobacco smoke	Isfahan	Borkhar and Meymeh	Female	2.88	0.00	8.23
**936**	Current tobacco smoke	Chaharmahal	Borujen	Female	1.23	0.00	3.92
**937**	Current tobacco smoke	Lorestan	Borujerd	Female	5.44	0.00	15.48
**938**	Current tobacco smoke	Khorasan_South	Boshruyeh	Female	2.64	0.00	9.60
**939**	Current tobacco smoke	Azararbayjan_East	Bostanabad	Female	0.60	0.00	4.95
**940**	Current tobacco smoke	Fars	Bovanat	Female	7.47	0.08	14.69
**941**	Current tobacco smoke	Kohkiluye and Bouyer Ahmad	Boyer Ahmad	Female	10.53	0.66	21.04
**942**	Current tobacco smoke	Qazvin	Boyinzahra	Female	1.61	0.00	9.24
**943**	Current tobacco smoke	Isfahan	Buein va Miandasht	Female	2.65	0.00	9.21
**944**	Current tobacco smoke	Azarbayjan_West	Bukan	Female	0.78	0.00	8.90
**945**	Current tobacco smoke	Boushehr	Bushehr	Female	14.82	3.42	27.77
**946**	Current tobacco smoke	Isfahan	Chadegan	Female	2.55	0.00	8.86
**947**	Current tobacco smoke	Sistan and Balouchestan	Chahbahar	Female	15.17	0.00	36.76
**948**	Current tobacco smoke	Azarbayjan_West	Chaipareh	Female	1.10	0.00	12.54
**949**	Current tobacco smoke	Azarbayjan_West	Chaldoran	Female	1.13	0.00	13.10
**950**	Current tobacco smoke	Mazandaran	Chalus	Female	3.48	0.00	14.11
**951**	Current tobacco smoke	Azararbayjan_East	Charoimaq	Female	0.99	0.00	7.11
**952**	Current tobacco smoke	Khorasan_razavi	Chenaran	Female	9.97	3.15	16.94
**953**	Current tobacco smoke	Kohkiluye and Bouyer Ahmad	Cheram	Female	9.61	0.00	20.76
**954**	Current tobacco smoke	Kermanshah	Dalaho	Female	1.47	0.00	4.58
**955**	Current tobacco smoke	Lorestan	Dalfan	Female	1.84	0.00	8.76
**956**	Current tobacco smoke	Sistan and Balouchestan	Dalgan	Female	12.61	0.00	34.08
**957**	Current tobacco smoke	Tehran	Damavand	Female	1.05	0.00	6.02
**958**	Current tobacco smoke	Semnan	Damghan	Female	0.31	0.00	2.22
**959**	Current tobacco smoke	Fars	Darab	Female	8.15	0.97	15.24
**960**	Current tobacco smoke	Khorasan_South	Darmian	Female	3.89	0.00	11.01
**961**	Current tobacco smoke	Khorasan_razavi	Darrehgaz	Female	4.57	0.00	9.80
**962**	Current tobacco smoke	Ilam	Darrehshahr	Female	0.29	0.00	2.49
**963**	Current tobacco smoke	Khuzestan	Dasht-e-Azadegan	Female	0.52	0.00	4.51
**964**	Current tobacco smoke	Boushehr	Dashtestan	Female	15.50	4.57	27.07
**965**	Current tobacco smoke	Boushehr	Dashti	Female	15.87	4.03	28.47
**966**	Current tobacco smoke	Khorasan_razavi	Davarzan	Female	6.42	0.00	13.68
**967**	Current tobacco smoke	Boushehr	Dayyer	Female	16.41	3.89	29.78
**968**	Current tobacco smoke	Kordestan	Dehgolan	Female	1.66	0.00	10.20
**969**	Current tobacco smoke	Ilam	Dehloran	Female	0.71	0.00	3.02
**970**	Current tobacco smoke	Markazi	Delijan	Female	3.83	0.00	9.57
**971**	Current tobacco smoke	Kohkiluye and Bouyer Ahmad	Dena	Female	9.26	0.00	20.57
**972**	Current tobacco smoke	Boushehr	Deylam	Female	11.93	0.42	24.37
**973**	Current tobacco smoke	Khuzestan	Dezful	Female	0.54	0.00	4.14
**974**	Current tobacco smoke	Kordestan	Divandarreh	Female	1.30	0.00	9.66
**975**	Current tobacco smoke	Lorestan	Dorud	Female	4.24	0.00	12.27

**976**	Current tobacco smoke	Lorestan	Doureh	Female	3.46	0.00	13.71
**977**	Current tobacco smoke	Fars	Eqlid	Female	7.03	0.30	13.61
**978**	Current tobacco smoke	Khorasan_North	Esfarayen	Female	5.32	0.00	14.40
**979**	Current tobacco smoke	Alborz	Eshtehard	Female	0.92	0.00	7.43
**980**	Current tobacco smoke	Kermanshah	Eslamabad-e-Gharb	Female	1.86	0.00	4.91
**981**	Current tobacco smoke	Tehran	Eslamshahr	Female	2.58	0.00	8.42
**982**	Current tobacco smoke	Fars	Estahban	Female	7.82	0.17	15.44
**983**	Current tobacco smoke	Ilam	Eyvan	Female	0.14	0.00	2.20
**984**	Current tobacco smoke	Kerman	Fahraj	Female	2.01	0.00	8.45
**985**	Current tobacco smoke	Isfahan	Falavarjan	Female	3.57	0.00	9.21
**986**	Current tobacco smoke	Hamedan	Famenin	Female	1.38	0.00	9.22
**987**	Current tobacco smoke	Markazi	Farahan	Female	3.18	0.00	9.39
**988**	Current tobacco smoke	Fars	Farashband	Female	9.29	2.76	16.08
**989**	Current tobacco smoke	Alborz	Fardis	Female	1.29	0.00	8.77
**990**	Current tobacco smoke	Isfahan	Faridan	Female	1.46	0.00	6.00
**991**	Current tobacco smoke	Khorasan_razavi	Fariman	Female	4.51	0.00	9.70
**992**	Current tobacco smoke	Khorasan_North	Faroj	Female	2.61	0.00	9.62
**993**	Current tobacco smoke	Chaharmahal	Farsan	Female	0.41	0.00	2.82
**994**	Current tobacco smoke	Kerman	Faryab	Female	2.46	0.00	8.56
**995**	Current tobacco smoke	Fars	Fasa	Female	7.77	1.23	14.05
**996**	Current tobacco smoke	Khorasan_South	Ferdows	Female	2.79	0.00	10.00
**997**	Current tobacco smoke	Mazandaran	Fereydunkenar	Female	1.18	0.00	8.74
**998**	Current tobacco smoke	Isfahan	Fereydunshahr	Female	2.59	0.00	9.07
**999**	Current tobacco smoke	Fars	Firozabad	Female	10.31	3.34	17.59
**1000**	Current tobacco smoke	Tehran	Firuzkuh	Female	1.20	0.00	6.13
**1001**	Current tobacco smoke	Sistan and Balouchestan	Fonuj	Female	12.78	0.00	33.48
**1002**	Current tobacco smoke	Gilan	Fuman	Female	0.30	0.00	6.08
**1003**	Current tobacco smoke	Kohkiluye and Bouyer Ahmad	Gachsaran	Female	10.33	0.00	21.10
**1004**	Current tobacco smoke	Golestan	Galikesh	Female	2.60	0.00	9.49
**1005**	Current tobacco smoke	Mazandaran	Galugah	Female	2.61	0.00	15.58
**1006**	Current tobacco smoke	Semnan	Garmsar	Female	0.18	0.00	2.09
**1007**	Current tobacco smoke	Boushehr	Genaveh	Female	14.32	2.98	26.88
**1008**	Current tobacco smoke	Fars	Gerash	Female	9.57	1.36	18.12
**1009**	Current tobacco smoke	Khorasan_North	Germeh	Female	3.05	0.00	13.49
**1010**	Current tobacco smoke	Ardebil	Germi	Female	0.69	0.00	6.60
**1011**	Current tobacco smoke	Kerman	Ghaleye-Ganj	Female	1.43	0.00	5.69
**1012**	Current tobacco smoke	Kermanshah	Gilan-e-Gharb	Female	1.51	0.00	4.68
**1013**	Current tobacco smoke	Isfahan	Golpayegan	Female	3.01	0.00	9.10
**1014**	Current tobacco smoke	Golestan	Gomishan	Female	1.45	0.00	10.66
**1015**	Current tobacco smoke	Khorasan_razavi	Gonabad	Female	7.56	0.55	14.49
**1016**	Current tobacco smoke	Golestan	Gonbad-e-Kavus	Female	2.13	0.00	7.59
**1017**	Current tobacco smoke	Golestan	Gorgan	Female	2.37	0.00	8.79
**1018**	Current tobacco smoke	Khuzestan	Guotvand	Female	1.05	0.00	6.67
**1019**	Current tobacco smoke	Khuzestan	Haftgol	Female	1.29	0.00	6.39
**1020**	Current tobacco smoke	Hormozgan	Hajiabad	Female	11.11	1.01	21.35
**1021**	Current tobacco smoke	Hamedan	Hamadan	Female	2.49	0.00	8.65
**1022**	Current tobacco smoke	Khuzestan	Hamidiyeh	Female	1.10	0.00	6.55
**1023**	Current tobacco smoke	Sistan and Balouchestan	Hamoon	Female	10.01	0.00	31.12
**1024**	Current tobacco smoke	Azararbayjan_East	Haris	Female	2.42	0.00	8.39
**1025**	Current tobacco smoke	Kermanshah	Harsin	Female	1.83	0.00	5.29
**1026**	Current tobacco smoke	Azararbayjan_East	Hashtrud	Female	0.84	0.00	7.58
**1027**	Current tobacco smoke	Khuzestan	Hendijan	Female	2.10	0.00	7.75
**1028**	Current tobacco smoke	Sistan and Balouchestan	Hirmand	Female	9.98	0.00	30.94
**1029**	Current tobacco smoke	Khuzestan	Hoveizeh	Female	1.17	0.00	7.17
**1030**	Current tobacco smoke	Zanjan	Ijerod	Female	1.67	0.00	10.90
**1031**	Current tobacco smoke	Ilam	Ilam	Female	0.19	0.00	2.29
**1032**	Current tobacco smoke	Sistan and Balouchestan	Iranshahr	Female	13.12	0.00	31.65
**1033**	Current tobacco smoke	Isfahan	Isfahan	Female	3.13	0.00	7.72
**1034**	Current tobacco smoke	Khuzestan	Izeh	Female	0.71	0.00	4.34
**1035**	Current tobacco smoke	Fars	Jahrom	Female	7.81	2.09	13.78
**1036**	Current tobacco smoke	Khorasan_North	Jajarm	Female	3.96	0.00	13.59
**1037**	Current tobacco smoke	Boushehr	Jam	Female	18.25	7.02	30.30
**1038**	Current tobacco smoke	Kermanshah	Javanrud	Female	0.68	0.00	3.02
**1039**	Current tobacco smoke	Kerman	Jiroft	Female	2.06	0.00	6.47
**1040**	Current tobacco smoke	Khorasan_razavi	Joghatai	Female	6.57	0.00	13.93
**1041**	Current tobacco smoke	Azararbayjan_East	Jolfa	Female	0.80	0.00	7.40
**1042**	Current tobacco smoke	Khorasan_razavi	Jowayin	Female	6.73	0.00	14.10
**1043**	Current tobacco smoke	Mazandaran	Juybar	Female	2.42	0.00	14.11
**1044**	Current tobacco smoke	Hamedan	Kabudarahang	Female	0.76	0.00	5.92
**1045**	Current tobacco smoke	Kerman	Kahnuj	Female	2.40	0.00	8.14
**1046**	Current tobacco smoke	Golestan	Kalaleh	Female	2.46	0.00	10.45
**1047**	Current tobacco smoke	Khorasan_razavi	Kalat	Female	8.25	0.39	15.84
**1048**	Current tobacco smoke	Azararbayjan_East	Kaleibar	Female	1.04	0.00	8.00
**1049**	Current tobacco smoke	Kordestan	Kamyaran	Female	0.87	0.00	6.78
**1050**	Current tobacco smoke	Boushehr	Kangan	Female	16.29	4.20	29.21
**1051**	Current tobacco smoke	Kermanshah	Kangavar	Female	4.27	1.96	6.60
**1052**	Current tobacco smoke	Alborz	Karaj	Female	1.56	0.00	6.53
**1053**	Current tobacco smoke	Khuzestan	Karun	Female	1.38	0.00	7.59
**1054**	Current tobacco smoke	Isfahan	Kashan	Female	2.11	0.00	7.07
**1055**	Current tobacco smoke	Khorasan_razavi	Kashmar	Female	7.79	0.98	14.75
**1056**	Current tobacco smoke	Fars	Kavar	Female	13.32	7.69	18.78
**1057**	Current tobacco smoke	Fars	Kazerun	Female	9.61	2.74	16.99
**1058**	Current tobacco smoke	Mazandaran	Kelardasht	Female	2.55	0.00	14.87
**1059**	Current tobacco smoke	Kerman	Kerman	Female	2.59	0.00	7.29
**1060**	Current tobacco smoke	Kermanshah	Kermanshah	Female	1.28	0.00	3.32
**1061**	Current tobacco smoke	Khorasan_razavi	Khaf	Female	8.46	1.45	15.63
**1062**	Current tobacco smoke	Khorasan_razavi	Khalilabad	Female	7.25	0.00	14.81
**1063**	Current tobacco smoke	Ardebil	Khalkhal	Female	0.34	0.00	4.04
**1064**	Current tobacco smoke	Hormozgan	Khamir	Female	12.77	1.97	24.09
**1065**	Current tobacco smoke	Isfahan	Khansar	Female	2.67	0.00	9.20
**1066**	Current tobacco smoke	Sistan and Balouchestan	Khash	Female	14.48	0.00	34.49
**1067**	Current tobacco smoke	Yazd	Khatam	Female	5.33	0.00	10.85
**1068**	Current tobacco smoke	Fars	Kherameh	Female	8.00	1.23	14.99
**1069**	Current tobacco smoke	Azararbayjan_East	Khodaafarin	Female	0.88	0.00	8.15
**1070**	Current tobacco smoke	Zanjan	Khodabandeh	Female	0.89	0.00	7.23
**1071**	Current tobacco smoke	Markazi	Khomeyn	Female	2.50	0.00	6.79
**1072**	Current tobacco smoke	Isfahan	Khomeynishahr	Female	3.85	0.00	10.22
**1073**	Current tobacco smoke	Markazi	Khondab	Female	2.31	0.00	6.58

**1074**	Current tobacco smoke	Fars	Khonj	Female	9.71	2.40	17.26
**1075**	Current tobacco smoke	Isfahan	Khoor va Biabanak	Female	2.37	0.00	9.15
**1076**	Current tobacco smoke	Lorestan	Khorramabad	Female	4.67	0.00	13.38
**1077**	Current tobacco smoke	Fars	Khorrambid	Female	7.31	0.03	14.68
**1078**	Current tobacco smoke	Zanjan	Khorramdarreh	Female	0.72	0.00	7.39
**1079**	Current tobacco smoke	Khuzestan	Khorramshahr	Female	0.75	0.00	4.76
**1080**	Current tobacco smoke	Khorasan_razavi	Khoshab	Female	7.07	0.02	14.12
**1081**	Current tobacco smoke	Azarbayjan_West	Khoy	Female	0.58	0.00	8.91
**1082**	Current tobacco smoke	Khorasan_South	Khusef	Female	2.88	0.00	9.57
**1083**	Current tobacco smoke	Chaharmahal	Kiaar	Female	0.52	0.00	2.99
**1084**	Current tobacco smoke	Kohkiluye and Bouyer Ahmad	Kohgiluyeh	Female	6.63	0.00	14.48
**1085**	Current tobacco smoke	Markazi	Komeijan	Female	3.32	0.00	9.21
**1086**	Current tobacco smoke	Sistan and Balouchestan	Konarak	Female	14.64	0.00	36.28
**1087**	Current tobacco smoke	Golestan	Kordkuy	Female	1.52	0.00	9.18
**1088**	Current tobacco smoke	Ardebil	Kowsar	Female	0.75	0.00	6.33
**1089**	Current tobacco smoke	Kerman	Kuhbonan	Female	1.04	0.00	7.33
**1090**	Current tobacco smoke	Lorestan	Kuhdasht	Female	3.82	0.00	13.09
**1091**	Current tobacco smoke	Chaharmahal	Kuhrang	Female	0.78	0.00	3.96
**1092**	Current tobacco smoke	Gilan	Lahijan	Female	0.36	0.00	6.51
**1093**	Current tobacco smoke	Khuzestan	Lali	Female	1.04	0.00	6.57
**1094**	Current tobacco smoke	Fars	Lamard	Female	14.50	7.39	22.28
**1095**	Current tobacco smoke	Kohkiluye and Bouyer Ahmad	Landeh	Female	8.47	0.00	21.23
**1096**	Current tobacco smoke	Gilan	Langrud	Female	0.36	0.00	6.24
**1097**	Current tobacco smoke	Isfahan	Lanjan	Female	1.68	0.00	6.23
**1098**	Current tobacco smoke	Fars	Lar (Larestan)	Female	5.29	0.29	10.36
**1099**	Current tobacco smoke	Chaharmahal	Lordakan	Female	1.30	0.00	3.95
**1100**	Current tobacco smoke	Azarbayjan_West	Mahabad	Female	2.89	0.00	13.84
**1101**	Current tobacco smoke	Markazi	Mahalat	Female	4.62	0.00	10.70
**1102**	Current tobacco smoke	Mazandaran	Mahmudabad	Female	0.87	0.00	8.87
**1103**	Current tobacco smoke	Zanjan	Mahneshan	Female	2.34	0.00	10.17
**1104**	Current tobacco smoke	Khorasan_razavi	Mahvelat	Female	7.52	0.60	14.41
**1105**	Current tobacco smoke	Azarbayjan_West	Maku	Female	2.68	0.00	13.45
**1106**	Current tobacco smoke	Tehran	Malard	Female	2.57	0.00	8.71
**1107**	Current tobacco smoke	Hamedan	Malayer	Female	3.91	0.00	10.87
**1108**	Current tobacco smoke	Azararbayjan_East	Malekan	Female	0.36	0.00	5.15
**1109**	Current tobacco smoke	Ilam	Malekshahi	Female	0.46	0.00	3.51
**1110**	Current tobacco smoke	Fars	Mamasany	Female	8.33	1.55	15.22
**1111**	Current tobacco smoke	Khorasan_North	Maneh and Samalqan	Female	1.81	0.00	8.94
**1112**	Current tobacco smoke	Kerman	Manujan	Female	2.92	0.00	9.31
**1113**	Current tobacco smoke	Azararbayjan_East	Maragheh	Female	0.47	0.00	4.80
**1114**	Current tobacco smoke	Azararbayjan_East	Marand	Female	0.44	0.00	5.01
**1115**	Current tobacco smoke	Golestan	Maravehtapeh	Female	2.18	0.00	10.30
**1116**	Current tobacco smoke	Kordestan	Marivan	Female	1.67	0.00	8.13
**1117**	Current tobacco smoke	Fars	Marvdasht	Female	7.17	0.99	13.36
**1118**	Current tobacco smoke	Gilan	Masal	Female	0.58	0.00	9.36
**1119**	Current tobacco smoke	Khorasan_razavi	Mashhad	Female	8.97	3.64	14.51
**1120**	Current tobacco smoke	Khuzestan	Masjed Soleyman	Female	1.13	0.00	6.37
**1121**	Current tobacco smoke	Semnan	Mayamey	Female	0.74	0.00	3.61
**1122**	Current tobacco smoke	Semnan	Mehdishahr	Female	0.44	0.00	3.20
**1123**	Current tobacco smoke	Ilam	Mehran	Female	0.23	0.00	2.42
**1124**	Current tobacco smoke	Yazd	Mehriz	Female	4.34	0.00	9.84
**1125**	Current tobacco smoke	Ardebil	Meshkinshahr	Female	0.50	0.00	4.39
**1126**	Current tobacco smoke	Yazd	Meybod	Female	2.02	0.00	5.96
**1127**	Current tobacco smoke	Hormozgan	Minab	Female	11.92	1.06	23.12
**1128**	Current tobacco smoke	Golestan	Minudasht	Female	1.22	0.00	6.36
**1129**	Current tobacco smoke	Sistan and Balouchestan	Mirjaveh	Female	12.99	0.00	36.48
**1130**	Current tobacco smoke	Azarbayjan_West	Miyandoab	Female	0.75	0.00	8.75
**1131**	Current tobacco smoke	Mazandaran	Miyandorud	Female	2.63	0.00	15.91
**1132**	Current tobacco smoke	Azararbayjan_East	Miyaneh	Female	0.61	0.00	5.09
**1133**	Current tobacco smoke	Isfahan	Mobarakeh	Female	3.64	0.00	9.68
**1134**	Current tobacco smoke	Fars	Mohr	Female	10.50	2.98	18.22
**1135**	Current tobacco smoke	Hamedan	Nahavand	Female	2.83	0.00	8.71
**1136**	Current tobacco smoke	Isfahan	Najafabad	Female	3.24	0.00	9.07
**1137**	Current tobacco smoke	Ardebil	Namin	Female	0.77	0.00	7.31
**1138**	Current tobacco smoke	Azarbayjan_West	Naqadeh	Female	1.09	0.00	9.51
**1139**	Current tobacco smoke	Kerman	Narmashir	Female	2.05	0.00	8.36
**1140**	Current tobacco smoke	Isfahan	Natanz	Female	1.23	0.00	5.63
**1141**	Current tobacco smoke	Isfahan	Nayin	Female	2.26	0.00	8.51
**1142**	Current tobacco smoke	Alborz	Nazarabad	Female	0.40	0.00	5.37
**1143**	Current tobacco smoke	Ardebil	Neer	Female	0.84	0.00	6.30
**1144**	Current tobacco smoke	Khorasan_South	Nehbandan	Female	1.67	0.00	6.41
**1145**	Current tobacco smoke	Mazandaran	Neka	Female	1.48	0.00	9.41
**1146**	Current tobacco smoke	Fars	Neyriz	Female	6.67	0.62	12.61
**1147**	Current tobacco smoke	Khorasan_razavi	Neyshabur	Female	7.97	2.15	13.79
**1148**	Current tobacco smoke	Sistan and Balouchestan	Nikshahr	Female	14.43	0.00	33.71
**1149**	Current tobacco smoke	Sistan and Balouchestan	Nimruz	Female	10.23	0.00	29.88
**1150**	Current tobacco smoke	Mazandaran	Noshahr	Female	3.14	0.00	16.40
**1151**	Current tobacco smoke	Mazandaran	Nur	Female	1.40	0.00	9.01
**1152**	Current tobacco smoke	Khuzestan	Omidiyeh	Female	1.89	0.00	7.03
**1153**	Current tobacco smoke	Azarbayjan_West	Orumiyeh	Female	3.88	0.00	14.74
**1154**	Current tobacco smoke	Azarbayjan_West	Oshnaviyeh	Female	2.05	0.00	15.74
**1155**	Current tobacco smoke	Azararbayjan_East	Osku	Female	0.73	0.00	7.28
**1156**	Current tobacco smoke	Tehran	Pakdasht	Female	1.01	0.00	5.84
**1157**	Current tobacco smoke	Tehran	Pardis	Female	1.91	0.00	9.02
**1158**	Current tobacco smoke	Ardebil	Parsabad	Female	0.33	0.00	4.37
**1159**	Current tobacco smoke	Hormozgan	Parsian (Gavbandi)	Female	14.61	3.06	27.16
**1160**	Current tobacco smoke	Fars	Pasargad	Female	7.02	0.23	14.04
**1161**	Current tobacco smoke	Kermanshah	Paveh	Female	1.38	0.00	4.73
**1162**	Current tobacco smoke	Azarbayjan_West	Piranshahr	Female	1.89	0.00	14.35
**1163**	Current tobacco smoke	Tehran	Pishva	Female	0.89	0.00	6.15
**1164**	Current tobacco smoke	Azarbayjan_West	Poldasht	Female	0.67	0.00	9.10
**1165**	Current tobacco smoke	Lorestan	Poldokhtar	Female	3.25	0.00	11.38
**1166**	Current tobacco smoke	Mazandaran	Qaemshahr	Female	1.32	0.00	9.07
**1167**	Current tobacco smoke	Tehran	Qarchak	Female	1.94	0.00	9.71
**1168**	Current tobacco smoke	Sistan and Balouchestan	Qasr qand	Female	13.32	0.00	34.68
**1169**	Current tobacco smoke	Kermanshah	Qasr-e-Shirin	Female	1.38	0.00	5.16
**1170**	Current tobacco smoke	Khorasan_South	Qayenat	Female	3.12	0.00	8.49
**1171**	Current tobacco smoke	Qazvin	Qazvin	Female	3.82	0.00	13.59

**1172**	Current tobacco smoke	Hormozgan	Qeshm	Female	11.46	1.86	21.69
**1173**	Current tobacco smoke	Fars	Qirokarzin	Female	9.74	2.85	17.01
**1174**	Current tobacco smoke	Qom	Qom	Female	3.82	0.00	19.90
**1175**	Current tobacco smoke	Kordestan	Qorveh	Female	1.11	0.00	6.99
**1176**	Current tobacco smoke	Khorasan_razavi	Quchan	Female	8.37	1.18	15.47
**1177**	Current tobacco smoke	Kerman	Rabar	Female	2.31	0.00	8.42
**1178**	Current tobacco smoke	Kerman	Rafsanjan	Female	1.07	0.00	5.11
**1179**	Current tobacco smoke	Khuzestan	Ramhormoz	Female	0.83	0.00	4.37
**1180**	Current tobacco smoke	Mazandaran	Ramsar	Female	2.21	0.00	15.21
**1181**	Current tobacco smoke	Khuzestan	Ramshir	Female	1.58	0.00	7.10
**1182**	Current tobacco smoke	Golestan	Ramyan	Female	1.99	0.00	9.63
**1183**	Current tobacco smoke	Gilan	Rasht	Female	0.35	0.00	6.02
**1184**	Current tobacco smoke	Khorasan_razavi	Rashtkhar	Female	7.87	0.80	15.14
**1185**	Current tobacco smoke	Kermanshah	Ravansar	Female	1.41	0.00	4.55
**1186**	Current tobacco smoke	Kerman	Ravar	Female	0.66	0.00	4.94
**1187**	Current tobacco smoke	Khorasan_North	Raz va Jergolan	Female	3.26	0.00	14.58
**1188**	Current tobacco smoke	Hamedan	Razan	Female	0.46	0.00	5.88
**1189**	Current tobacco smoke	Tehran	Rey	Female	2.77	0.00	9.14
**1190**	Current tobacco smoke	Kerman	Reygan	Female	2.04	0.00	7.95
**1191**	Current tobacco smoke	Gilan	Rezvanshahr	Female	0.69	0.00	9.40
**1192**	Current tobacco smoke	Tehran	Robatkarim	Female	1.16	0.00	6.18
**1193**	Current tobacco smoke	Fars	Rostam	Female	7.62	0.00	15.69
**1194**	Current tobacco smoke	Kerman	Roudbar-e-Jonub	Female	1.22	0.00	5.48
**1195**	Current tobacco smoke	Hormozgan	Rudan	Female	11.37	0.76	22.28
**1196**	Current tobacco smoke	Gilan	Rudbar	Female	0.33	0.00	6.04
**1197**	Current tobacco smoke	Gilan	Rudsar	Female	0.43	0.00	6.26
**1198**	Current tobacco smoke	Lorestan	Rumshekan	Female	2.97	0.00	13.70
**1199**	Current tobacco smoke	Khorasan_razavi	Sabzevar	Female	4.71	0.00	9.68
**1200**	Current tobacco smoke	Yazd	Sadugh	Female	3.78	0.00	9.40
**1201**	Current tobacco smoke	Kermanshah	Sahneh	Female	1.98	0.00	5.09
**1202**	Current tobacco smoke	Kermanshah	Salas-e-Babajani	Female	1.35	0.00	4.49
**1203**	Current tobacco smoke	Azarbayjan_West	Salmas	Female	0.62	0.00	8.88
**1204**	Current tobacco smoke	Chaharmahal	Saman	Female	0.76	0.00	4.42
**1205**	Current tobacco smoke	Kordestan	Sanandaj	Female	2.30	0.00	9.83
**1206**	Current tobacco smoke	Kordestan	Saqqez	Female	0.53	0.00	6.41
**1207**	Current tobacco smoke	Kermanshah	Sar-e-Pol-e-Zohab	Female	0.78	0.00	3.06
**1208**	Current tobacco smoke	Azararbayjan_East	Sarab	Female	1.69	0.00	6.85
**1209**	Current tobacco smoke	Khorasan_razavi	Sarakhs	Female	8.72	1.17	16.53
**1210**	Current tobacco smoke	Sistan and Balouchestan	Saravan	Female	15.19	0.00	39.04
**1211**	Current tobacco smoke	Khorasan_South	Sarayan	Female	2.88	0.00	9.35
**1212**	Current tobacco smoke	Sistan and Balouchestan	Sarbaz	Female	7.26	0.00	20.80
**1213**	Current tobacco smoke	Khorasan_South	Sarbisheh	Female	3.79	0.00	10.74
**1214**	Current tobacco smoke	Azarbayjan_West	Sardasht	Female	2.31	0.00	14.37
**1215**	Current tobacco smoke	Ardebil	Sarein	Female	0.83	0.00	6.66
**1216**	Current tobacco smoke	Mazandaran	Sari	Female	3.95	0.00	13.69
**1217**	Current tobacco smoke	Kordestan	Sarvabad	Female	1.25	0.00	10.26
**1218**	Current tobacco smoke	Fars	Sarvestan	Female	8.88	1.70	16.39
**1219**	Current tobacco smoke	Mazandaran	Savadkuh	Female	2.57	0.00	13.58
**1220**	Current tobacco smoke	Mazandaran	Savadkuh_North	Female	2.48	0.00	14.44
**1221**	Current tobacco smoke	Markazi	Saveh	Female	1.74	0.00	5.95
**1222**	Current tobacco smoke	Alborz	Savojbolagh	Female	0.53	0.00	5.30
**1223**	Current tobacco smoke	Lorestan	Selseleh	Female	3.89	0.00	14.27
**1224**	Current tobacco smoke	Isfahan	Semirom	Female	4.35	0.00	10.36
**1225**	Current tobacco smoke	Isfahan	Semirom-e-Sofla	Female	3.65	0.00	10.40
**1226**	Current tobacco smoke	Semnan	Semnan	Female	0.77	0.00	3.09
**1227**	Current tobacco smoke	Fars	Sepidan	Female	7.97	0.76	15.20
**1228**	Current tobacco smoke	Azararbayjan_East	Shabestar	Female	0.49	0.00	4.95
**1229**	Current tobacco smoke	Khuzestan	Shadegan	Female	0.85	0.00	4.60
**1230**	Current tobacco smoke	Gilan	Shaft	Female	0.33	0.00	6.34
**1231**	Current tobacco smoke	Azarbayjan_West	Shahindezh	Female	0.88	0.00	8.86
**1232**	Current tobacco smoke	Tehran	Shahr-e Qods	Female	2.55	0.00	8.03
**1233**	Current tobacco smoke	Kerman	Shahr-e-Babak	Female	1.15	0.00	5.34
**1234**	Current tobacco smoke	Chaharmahal	Shahr-e-Kord	Female	0.76	0.00	3.17
**1235**	Current tobacco smoke	Isfahan	Shahreza	Female	4.16	0.00	10.46
**1236**	Current tobacco smoke	Tehran	Shahriyar	Female	2.82	0.00	8.55
**1237**	Current tobacco smoke	Semnan	Shahrud	Female	0.45	0.00	2.36
**1238**	Current tobacco smoke	Markazi	Shazand	Female	11.36	6.25	16.55
**1239**	Current tobacco smoke	Tehran	Shemiranat	Female	2.40	0.00	8.90
**1240**	Current tobacco smoke	Fars	Shiraz	Female	9.65	3.54	15.90
**1241**	Current tobacco smoke	Khorasan_North	Shirvan	Female	3.18	0.00	10.05
**1242**	Current tobacco smoke	Ilam	Shirvan and Chard-e-Aval	Female	0.16	0.00	2.21
**1243**	Current tobacco smoke	Azarbayjan_West	Showt	Female	0.71	0.00	8.91
**1244**	Current tobacco smoke	Khuzestan	Shush	Female	1.37	0.00	5.74
**1245**	Current tobacco smoke	Khuzestan	Shushtar	Female	1.63	0.00	6.04
**1246**	Current tobacco smoke	Gilan	Siahkal	Female	0.67	0.00	8.74
**1247**	Current tobacco smoke	Sistan and Balouchestan	Sib o Soran	Female	13.55	0.00	37.56
**1248**	Current tobacco smoke	Mazandaran	Simorgh	Female	2.20	0.00	13.81
**1249**	Current tobacco smoke	Hormozgan	Sirik	Female	12.73	1.09	25.14
**1250**	Current tobacco smoke	Kerman	Sirjan	Female	1.73	0.00	5.98
**1251**	Current tobacco smoke	Ilam	Sirvan	Female	0.39	0.00	3.28
**1252**	Current tobacco smoke	Zanjan	Soltaniyeh	Female	1.57	0.00	10.87
**1253**	Current tobacco smoke	Kermanshah	Sonqor	Female	2.00	0.00	4.91
**1254**	Current tobacco smoke	Semnan	Sorkheh	Female	0.52	0.00	3.37
**1255**	Current tobacco smoke	Gilan	Sume'eh Sara	Female	0.42	0.00	6.25
**1256**	Current tobacco smoke	Khorasan_South	Tabas	Female	1.38	0.00	5.93
**1257**	Current tobacco smoke	Azararbayjan_East	Tabriz	Female	1.22	0.00	5.88
**1258**	Current tobacco smoke	Markazi	Tafresh	Female	3.12	0.00	9.07
**1259**	Current tobacco smoke	Yazd	Taft	Female	4.64	0.00	9.71
**1260**	Current tobacco smoke	Azarbayjan_West	Takab	Female	1.04	0.00	9.15
**1261**	Current tobacco smoke	Qazvin	Takestan	Female	1.32	0.00	9.03
**1262**	Current tobacco smoke	Khorasan_razavi	Takht-e-Jolgeh (Firuzeh)	Female	7.09	0.00	14.47
**1263**	Current tobacco smoke	Alborz	Taleghan	Female	0.58	0.00	5.21
**1264**	Current tobacco smoke	Boushehr	Tangestan	Female	15.67	3.70	27.90
**1265**	Current tobacco smoke	Zanjan	Tarom	Female	0.84	0.00	7.32
**1266**	Current tobacco smoke	Gilan	Tavalesh	Female	0.43	0.00	6.37
**1267**	Current tobacco smoke	Khorasan_razavi	Taybad	Female	9.62	1.91	17.69
**1268**	Current tobacco smoke	Tehran	Tehran	Female	2.93	0.00	7.92
**1269**	Current tobacco smoke	Isfahan	Tiran and Karvan	Female	2.56	0.00	9.05

**1270**	Current tobacco smoke	Mazandaran	Tonekabon	Female	3.58	0.00	13.34
**1271**	Current tobacco smoke	Khorasan_razavi	Torbat-e-Heydariyeh	Female	7.49	1.50	13.60
**1272**	Current tobacco smoke	Khorasan_razavi	Torbat-e-Jam	Female	11.51	4.76	18.77
**1273**	Current tobacco smoke	Hamedan	Tuyserkan	Female	1.53	0.00	6.79
**1274**	Current tobacco smoke	Tehran	Varamin	Female	2.31	0.00	7.32
**1275**	Current tobacco smoke	Azararbayjan_East	Varzaqan	Female	0.98	0.00	7.19
**1276**	Current tobacco smoke	Yazd	Yazd	Female	3.65	0.00	7.95
**1277**	Current tobacco smoke	Sistan and Balouchestan	Zabol	Female	5.20	0.00	19.50
**1278**	Current tobacco smoke	Sistan and Balouchestan	Zaboli (Mehrestan)	Female	12.84	0.00	33.56
**1279**	Current tobacco smoke	Sistan and Balouchestan	Zahedan	Female	14.07	0.00	32.15
**1280**	Current tobacco smoke	Zanjan	Zanjan	Female	2.54	0.00	10.21
**1281**	Current tobacco smoke	Kerman	Zarand	Female	1.95	0.00	7.20
**1282**	Current tobacco smoke	Markazi	Zarandiyeh	Female	2.93	0.00	8.94
**1283**	Current tobacco smoke	Fars	Zarrindasht	Female	8.26	0.63	15.75
**1284**	Current tobacco smoke	Khorasan_razavi	Zave	Female	8.18	1.10	15.44
**1285**	Current tobacco smoke	Sistan and Balouchestan	Zehak	Female	9.19	0.00	31.83
**1286**	Current tobacco smoke	Khorasan_South	Zir kuh	Female	3.37	0.00	10.79
**1287**	Current tobacco smoke	Khuzestan	Abadan	Male	25.23	6.87	43.03
**1288**	Current tobacco smoke	Fars	Abadeh	Male	34.53	22.31	47.29
**1289**	Current tobacco smoke	Yazd	Abarkuh	Male	29.36	13.46	45.64
**1290**	Current tobacco smoke	Mazandaran	Abbas abad	Male	45.16	28.91	59.32
**1291**	Current tobacco smoke	Ilam	Abdanan	Male	13.24	1.52	25.11
**1292**	Current tobacco smoke	Zanjan	Abhar	Male	22.95	10.38	34.64
**1293**	Current tobacco smoke	Hormozgan	Abumusa	Male	23.09	0.00	45.58
**1294**	Current tobacco smoke	Qazvin	Abyek	Male	32.18	12.34	50.21
**1295**	Current tobacco smoke	Azararbayjan_East	Ahar	Male	24.45	10.47	38.24
**1296**	Current tobacco smoke	Khuzestan	Ahvaz	Male	25.31	12.91	38.30
**1297**	Current tobacco smoke	Azararbayjan_East	Ajabshir	Male	24.54	9.67	40.03
**1298**	Current tobacco smoke	Qazvin	Alborz	Male	35.18	15.57	54.95
**1299**	Current tobacco smoke	Golestan	Aliabad	Male	17.66	3.19	31.51
**1300**	Current tobacco smoke	Lorestan	Aligudarz	Male	26.42	10.41	41.50
**1301**	Current tobacco smoke	Gilan	Amlash	Male	25.25	8.13	42.40
**1302**	Current tobacco smoke	Mazandaran	Amol	Male	29.32	14.20	44.55
**1303**	Current tobacco smoke	Kerman	Anar	Male	22.81	7.99	38.77
**1304**	Current tobacco smoke	Kerman	Anbarabad	Male	18.69	5.60	32.21
**1305**	Current tobacco smoke	Khuzestan	Andika	Male	10.91	0.00	25.26
**1306**	Current tobacco smoke	Khuzestan	Andimeshk	Male	18.46	3.41	32.55
**1307**	Current tobacco smoke	Golestan	Aq Qala	Male	20.61	4.27	37.67
**1308**	Current tobacco smoke	Khuzestan	Aqajari	Male	22.26	2.26	42.22
**1309**	Current tobacco smoke	Semnan	Aradan	Male	23.65	6.92	40.37
**1310**	Current tobacco smoke	Markazi	Arak	Male	28.82	13.38	43.66
**1311**	Current tobacco smoke	Isfahan	Aran and Bidgol	Male	24.36	9.80	38.38
**1312**	Current tobacco smoke	Ardebil	Ardabil	Male	23.36	9.82	36.39
**1313**	Current tobacco smoke	Yazd	Ardakan	Male	26.32	12.35	41.47
**1314**	Current tobacco smoke	Chaharmahal	Ardal	Male	29.32	12.71	46.41
**1315**	Current tobacco smoke	Isfahan	Ardestan	Male	26.11	9.24	43.12
**1316**	Current tobacco smoke	Fars	Arsanjan	Male	38.50	25.00	50.99
**1317**	Current tobacco smoke	Kerman	Arzouyeh	Male	34.99	21.81	47.21
**1318**	Current tobacco smoke	Hamedan	Asadabad	Male	24.36	7.81	40.35
**1319**	Current tobacco smoke	Boushehr	Asaluyeh	Male	24.90	5.69	43.34
**1320**	Current tobacco smoke	Markazi	Ashtiyan	Male	28.71	9.40	47.87
**1321**	Current tobacco smoke	Gilan	Astaneh-ye-Ashrafiyeh	Male	26.36	11.32	42.21
**1322**	Current tobacco smoke	Gilan	Astara	Male	19.48	2.29	35.52
**1323**	Current tobacco smoke	Qazvin	Avaj	Male	35.51	14.81	57.03
**1324**	Current tobacco smoke	Golestan	Azadshahr	Male	16.01	1.05	30.55
**1325**	Current tobacco smoke	Azararbayjan_East	Azarshahr	Male	25.32	7.93	43.41
**1326**	Current tobacco smoke	Lorestan	Azna	Male	27.84	10.18	45.81
**1327**	Current tobacco smoke	Mazandaran	Babol	Male	29.77	14.80	45.49
**1328**	Current tobacco smoke	Mazandaran	Babolsar	Male	32.82	13.16	53.48
**1329**	Current tobacco smoke	Ilam	Badreh	Male	15.06	0.00	30.60
**1330**	Current tobacco smoke	Yazd	Bafq	Male	19.09	7.82	30.87
**1331**	Current tobacco smoke	Kerman	Baft	Male	23.16	9.83	36.28
**1332**	Current tobacco smoke	Khuzestan	Baghemalek	Male	20.23	5.62	34.51
**1333**	Current tobacco smoke	Yazd	Bahabad	Male	22.81	4.68	40.04
**1334**	Current tobacco smoke	Hamedan	Bahar	Male	31.68	15.65	49.17
**1335**	Current tobacco smoke	Tehran	Baharestan (Golestan)	Male	24.33	10.56	38.92
**1336**	Current tobacco smoke	Kohkiluye and Bouyer Ahmad	Bahmani	Male	16.65	1.94	30.69
**1337**	Current tobacco smoke	Khorasan_razavi	Bajestan	Male	17.54	3.69	30.78
**1338**	Current tobacco smoke	Khorasan_razavi	Bakhraz	Male	18.63	0.96	35.94
**1339**	Current tobacco smoke	Kerman	Bam	Male	22.18	9.55	35.39
**1340**	Current tobacco smoke	Hormozgan	Bandar-e-Abbas	Male	23.37	9.95	36.88
**1341**	Current tobacco smoke	Gilan	Bandar-e-Anzali	Male	23.74	8.61	39.34
**1342**	Current tobacco smoke	Golestan	Bandar-e-Gaz	Male	16.93	0.00	34.61
**1343**	Current tobacco smoke	Hormozgan	Bandar-e-Jask	Male	24.93	8.02	42.55
**1344**	Current tobacco smoke	Hormozgan	Bandar-e-Lengeh	Male	23.74	6.61	40.57
**1345**	Current tobacco smoke	Khuzestan	Bandar-e-Mahshahr	Male	25.68	9.54	43.44
**1346**	Current tobacco smoke	Golestan	Bandar-e-Torkaman	Male	18.75	1.80	35.44
**1347**	Current tobacco smoke	Kordestan	Baneh	Male	31.32	10.59	53.35
**1348**	Current tobacco smoke	Khorasan_razavi	Bardeskan	Male	20.54	4.10	37.08
**1349**	Current tobacco smoke	Kerman	Bardsir	Male	25.31	11.84	40.29
**1350**	Current tobacco smoke	Hormozgan	Bashagerd	Male	23.15	6.88	38.69
**1351**	Current tobacco smoke	Kohkiluye and Bouyer Ahmad	Basht	Male	17.48	5.30	30.31
**1352**	Current tobacco smoke	Hormozgan	Bastak	Male	22.78	4.90	40.58
**1353**	Current tobacco smoke	Khuzestan	Bavi	Male	20.06	2.40	37.14
**1354**	Current tobacco smoke	Khuzestan	Behbahan	Male	23.35	7.93	39.78
**1355**	Current tobacco smoke	Mazandaran	Behshahr	Male	12.90	0.00	28.90
**1356**	Current tobacco smoke	Kordestan	Bijar	Male	22.77	7.82	36.81
**1357**	Current tobacco smoke	Ardebil	Bilehsavar	Male	25.23	6.01	44.21
**1358**	Current tobacco smoke	Khorasan_razavi	Binaloud	Male	21.31	2.76	40.98
**1359**	Current tobacco smoke	Khorasan_South	Birjand	Male	12.88	2.19	23.54
**1360**	Current tobacco smoke	Khorasan_North	Bojnurd	Male	18.21	5.57	30.15
**1361**	Current tobacco smoke	Chaharmahal	Bon	Male	29.48	9.91	50.14
**1362**	Current tobacco smoke	Azararbayjan_East	Bonab	Male	25.29	11.80	39.22
**1363**	Current tobacco smoke	Isfahan	Borkhar	Male	28.32	11.30	46.22
**1364**	Current tobacco smoke	Isfahan	Borkhar and Meymeh	Male	27.03	12.80	41.22
**1365**	Current tobacco smoke	Chaharmahal	Borujen	Male	30.44	15.41	45.58
**1366**	Current tobacco smoke	Lorestan	Borujerd	Male	32.12	16.29	48.57
**1367**	Current tobacco smoke	Khorasan_South	Boshruyeh	Male	11.54	0.00	24.22

**1368**	Current tobacco smoke	Azararbayjan_East	Bostanabad	Male	25.35	11.71	40.15
**1369**	Current tobacco smoke	Fars	Bovanat	Male	22.87	8.37	35.96
**1370**	Current tobacco smoke	Kohkiluye and Bouyer Ahmad	Boyer Ahmad	Male	27.56	13.31	42.90
**1371**	Current tobacco smoke	Qazvin	Boyinzahra	Male	43.35	24.81	64.07
**1372**	Current tobacco smoke	Isfahan	Buein va Miandasht	Male	26.66	9.41	43.98
**1373**	Current tobacco smoke	Azarbayjan_West	Bukan	Male	26.62	10.08	42.62
**1374**	Current tobacco smoke	Boushehr	Bushehr	Male	25.72	8.46	43.36
**1375**	Current tobacco smoke	Isfahan	Chadegan	Male	27.52	11.76	43.75
**1376**	Current tobacco smoke	Sistan and Balouchestan	Chahbahar	Male	18.05	3.47	32.39
**1377**	Current tobacco smoke	Azarbayjan_West	Chaipareh	Male	28.50	9.44	48.60
**1378**	Current tobacco smoke	Azarbayjan_West	Chaldoran	Male	28.95	7.61	50.55
**1379**	Current tobacco smoke	Mazandaran	Chalus	Male	34.61	16.94	52.65
**1380**	Current tobacco smoke	Azararbayjan_East	Charoimaq	Male	23.33	9.26	37.84
**1381**	Current tobacco smoke	Khorasan_razavi	Chenaran	Male	20.06	3.40	37.08
**1382**	Current tobacco smoke	Kohkiluye and Bouyer Ahmad	Cheram	Male	25.75	14.53	36.99
**1383**	Current tobacco smoke	Kermanshah	Dalaho	Male	18.26	0.00	36.45
**1384**	Current tobacco smoke	Lorestan	Dalfan	Male	24.99	7.98	41.27
**1385**	Current tobacco smoke	Sistan and Balouchestan	Dalgan	Male	16.57	7.04	26.22
**1386**	Current tobacco smoke	Tehran	Damavand	Male	22.89	8.95	37.37
**1387**	Current tobacco smoke	Semnan	Damghan	Male	22.22	7.50	37.26
**1388**	Current tobacco smoke	Fars	Darab	Male	28.16	12.95	43.91
**1389**	Current tobacco smoke	Khorasan_South	Darmian	Male	12.19	0.00	24.43
**1390**	Current tobacco smoke	Khorasan_razavi	Darrehgaz	Male	8.63	0.00	22.45
**1391**	Current tobacco smoke	Ilam	Darrehshahr	Male	10.72	0.00	23.71
**1392**	Current tobacco smoke	Khuzestan	Dasht-e-Azadegan	Male	21.72	2.09	40.74
**1393**	Current tobacco smoke	Boushehr	Dashtestan	Male	27.48	11.26	44.40
**1394**	Current tobacco smoke	Boushehr	Dashti	Male	23.24	6.98	38.11
**1395**	Current tobacco smoke	Khorasan_razavi	Davarzan	Male	20.71	2.64	38.82
**1396**	Current tobacco smoke	Boushehr	Dayyer	Male	33.22	15.04	53.85
**1397**	Current tobacco smoke	Kordestan	Dehgolan	Male	26.67	10.21	44.19
**1398**	Current tobacco smoke	Ilam	Dehloran	Male	16.62	1.86	31.83
**1399**	Current tobacco smoke	Markazi	Delijan	Male	30.06	17.71	42.76
**1400**	Current tobacco smoke	Kohkiluye and Bouyer Ahmad	Dena	Male	28.74	10.58	47.94
**1401**	Current tobacco smoke	Boushehr	Deylam	Male	25.87	6.94	44.42
**1402**	Current tobacco smoke	Khuzestan	Dezful	Male	19.15	5.89	31.93
**1403**	Current tobacco smoke	Kordestan	Divandarreh	Male	30.47	11.68	49.29
**1404**	Current tobacco smoke	Lorestan	Dorud	Male	28.25	10.11	46.47
**1405**	Current tobacco smoke	Lorestan	Doureh	Male	25.01	7.02	43.43
**1406**	Current tobacco smoke	Fars	Eqlid	Male	29.91	14.83	45.86
**1407**	Current tobacco smoke	Khorasan_North	Esfarayen	Male	22.41	10.57	35.14
**1408**	Current tobacco smoke	Alborz	Eshtehard	Male	29.97	12.95	47.55
**1409**	Current tobacco smoke	Kermanshah	Eslamabad-e-Gharb	Male	17.13	2.44	31.62
**1410**	Current tobacco smoke	Tehran	Eslamshahr	Male	25.16	11.96	38.71
**1411**	Current tobacco smoke	Fars	Estahban	Male	26.83	10.00	43.33
**1412**	Current tobacco smoke	Ilam	Eyvan	Male	7.35	0.00	19.88
**1413**	Current tobacco smoke	Kerman	Fahraj	Male	16.97	7.43	26.65
**1414**	Current tobacco smoke	Isfahan	Falavarjan	Male	29.67	13.98	46.61
**1415**	Current tobacco smoke	Hamedan	Famenin	Male	28.98	11.52	46.92
**1416**	Current tobacco smoke	Markazi	Farahan	Male	28.47	8.69	47.88
**1417**	Current tobacco smoke	Fars	Farashband	Male	24.80	8.73	41.14
**1418**	Current tobacco smoke	Alborz	Fardis	Male	28.97	10.59	47.76
**1419**	Current tobacco smoke	Isfahan	Faridan	Male	28.89	13.07	45.06
**1420**	Current tobacco smoke	Khorasan_razavi	Fariman	Male	8.49	0.00	21.77
**1421**	Current tobacco smoke	Khorasan_North	Faroj	Male	19.58	4.25	35.16
**1422**	Current tobacco smoke	Chaharmahal	Farsan	Male	29.61	12.76	46.58
**1423**	Current tobacco smoke	Kerman	Faryab	Male	21.39	7.10	35.74
**1424**	Current tobacco smoke	Fars	Fasa	Male	28.12	12.97	44.19
**1425**	Current tobacco smoke	Khorasan_South	Ferdows	Male	9.78	0.00	20.12
**1426**	Current tobacco smoke	Mazandaran	Fereydunkenar	Male	28.54	10.26	47.15
**1427**	Current tobacco smoke	Isfahan	Fereydunshahr	Male	24.19	8.58	38.50
**1428**	Current tobacco smoke	Fars	Firozabad	Male	27.73	12.77	44.00
**1429**	Current tobacco smoke	Tehran	Firuzkuh	Male	19.60	5.85	32.48
**1430**	Current tobacco smoke	Sistan and Balouchestan	Fonuj	Male	16.83	0.88	32.34
**1431**	Current tobacco smoke	Gilan	Fuman	Male	22.58	9.05	35.51
**1432**	Current tobacco smoke	Kohkiluye and Bouyer Ahmad	Gachsaran	Male	24.38	8.16	40.84
**1433**	Current tobacco smoke	Golestan	Galikesh	Male	17.04	2.05	31.19
**1434**	Current tobacco smoke	Mazandaran	Galugah	Male	21.17	0.00	43.11
**1435**	Current tobacco smoke	Semnan	Garmsar	Male	23.45	9.21	37.41
**1436**	Current tobacco smoke	Boushehr	Genaveh	Male	26.73	9.12	45.22
**1437**	Current tobacco smoke	Fars	Gerash	Male	24.11	4.70	43.14
**1438**	Current tobacco smoke	Khorasan_North	Germeh	Male	18.76	1.17	35.76
**1439**	Current tobacco smoke	Ardebil	Germi	Male	27.09	11.07	43.83
**1440**	Current tobacco smoke	Kerman	Ghaleye-Ganj	Male	9.62	0.00	21.19
**1441**	Current tobacco smoke	Kermanshah	Gilan-e-Gharb	Male	16.98	0.42	33.04
**1442**	Current tobacco smoke	Isfahan	Golpayegan	Male	28.92	13.63	45.10
**1443**	Current tobacco smoke	Golestan	Gomishan	Male	19.27	0.00	39.66
**1444**	Current tobacco smoke	Khorasan_razavi	Gonabad	Male	18.36	1.40	35.88
**1445**	Current tobacco smoke	Golestan	Gonbad-e-Kavus	Male	17.33	3.77	30.62
**1446**	Current tobacco smoke	Golestan	Gorgan	Male	21.51	8.67	35.36
**1447**	Current tobacco smoke	Khuzestan	Guotvand	Male	20.35	2.60	37.99
**1448**	Current tobacco smoke	Khuzestan	Haftgol	Male	21.83	4.55	38.67
**1449**	Current tobacco smoke	Hormozgan	Hajiabad	Male	27.04	12.23	42.15
**1450**	Current tobacco smoke	Hamedan	Hamadan	Male	30.87	14.42	47.28
**1451**	Current tobacco smoke	Khuzestan	Hamidiyeh	Male	22.06	2.31	41.41
**1452**	Current tobacco smoke	Sistan and Balouchestan	Hamoon	Male	14.29	0.00	31.35
**1453**	Current tobacco smoke	Azararbayjan_East	Haris	Male	23.99	9.32	38.35
**1454**	Current tobacco smoke	Kermanshah	Harsin	Male	19.90	2.05	37.20
**1455**	Current tobacco smoke	Azararbayjan_East	Hashtrud	Male	25.13	11.62	39.64
**1456**	Current tobacco smoke	Khuzestan	Hendijan	Male	23.33	4.53	42.23
**1457**	Current tobacco smoke	Sistan and Balouchestan	Hirmand	Male	13.29	0.00	31.90
**1458**	Current tobacco smoke	Khuzestan	Hoveizeh	Male	22.35	1.46	42.52
**1459**	Current tobacco smoke	Zanjan	Ijerod	Male	25.76	15.83	35.63
**1460**	Current tobacco smoke	Ilam	Ilam	Male	14.85	1.04	27.88
**1461**	Current tobacco smoke	Sistan and Balouchestan	Iranshahr	Male	18.78	5.98	31.65
**1462**	Current tobacco smoke	Isfahan	Isfahan	Male	28.40	17.09	39.96
**1463**	Current tobacco smoke	Khuzestan	Izeh	Male	22.57	8.09	37.28
**1464**	Current tobacco smoke	Fars	Jahrom	Male	25.69	10.67	40.62
**1465**	Current tobacco smoke	Khorasan_North	Jajarm	Male	21.99	6.71	37.94

**1466**	Current tobacco smoke	Boushehr	Jam	Male	26.16	8.99	43.21
**1467**	Current tobacco smoke	Kermanshah	Javanrud	Male	15.81	0.00	32.42
**1468**	Current tobacco smoke	Kerman	Jiroft	Male	20.84	9.40	31.96
**1469**	Current tobacco smoke	Khorasan_razavi	Joghatai	Male	20.79	2.33	39.18
**1470**	Current tobacco smoke	Azararbayjan_East	Jolfa	Male	22.87	6.81	39.02
**1471**	Current tobacco smoke	Khorasan_razavi	Jowayin	Male	21.33	4.12	39.61
**1472**	Current tobacco smoke	Mazandaran	Juybar	Male	28.14	9.09	46.96
**1473**	Current tobacco smoke	Hamedan	Kabudarahang	Male	27.66	10.53	44.17
**1474**	Current tobacco smoke	Kerman	Kahnuj	Male	14.06	2.82	24.71
**1475**	Current tobacco smoke	Golestan	Kalaleh	Male	16.27	1.80	30.10
**1476**	Current tobacco smoke	Khorasan_razavi	Kalat	Male	16.20	0.00	36.05
**1477**	Current tobacco smoke	Azararbayjan_East	Kaleibar	Male	23.54	6.93	40.65
**1478**	Current tobacco smoke	Kordestan	Kamyaran	Male	25.52	7.18	43.43
**1479**	Current tobacco smoke	Boushehr	Kangan	Male	27.45	8.67	46.63
**1480**	Current tobacco smoke	Kermanshah	Kangavar	Male	22.71	8.31	37.36
**1481**	Current tobacco smoke	Alborz	Karaj	Male	30.64	18.79	42.95
**1482**	Current tobacco smoke	Khuzestan	Karun	Male	23.03	0.94	44.41
**1483**	Current tobacco smoke	Isfahan	Kashan	Male	25.82	10.47	40.43
**1484**	Current tobacco smoke	Khorasan_razavi	Kashmar	Male	20.00	3.62	36.21
**1485**	Current tobacco smoke	Fars	Kavar	Male	27.16	10.29	43.80
**1486**	Current tobacco smoke	Fars	Kazerun	Male	25.61	11.41	39.80
**1487**	Current tobacco smoke	Mazandaran	Kelardasht	Male	35.74	15.03	57.61
**1488**	Current tobacco smoke	Kerman	Kerman	Male	21.03	10.30	31.87
**1489**	Current tobacco smoke	Kermanshah	Kermanshah	Male	21.40	8.70	34.56
**1490**	Current tobacco smoke	Khorasan_razavi	Khaf	Male	18.67	3.01	34.68
**1491**	Current tobacco smoke	Khorasan_razavi	Khalilabad	Male	21.63	4.49	39.20
**1492**	Current tobacco smoke	Ardebil	Khalkhal	Male	25.87	10.83	40.62
**1493**	Current tobacco smoke	Hormozgan	Khamir	Male	20.46	9.92	32.01
**1494**	Current tobacco smoke	Isfahan	Khansar	Male	25.96	9.50	41.78
**1495**	Current tobacco smoke	Sistan and Balouchestan	Khash	Male	26.10	12.73	40.48
**1496**	Current tobacco smoke	Yazd	Khatam	Male	26.94	10.66	43.04
**1497**	Current tobacco smoke	Fars	Kherameh	Male	24.67	10.31	38.59
**1498**	Current tobacco smoke	Azararbayjan_East	Khodaafarin	Male	23.51	6.52	40.31
**1499**	Current tobacco smoke	Zanjan	Khodabandeh	Male	22.73	10.15	34.73
**1500**	Current tobacco smoke	Markazi	Khomeyn	Male	33.88	18.64	49.83
**1501**	Current tobacco smoke	Isfahan	Khomeynishahr	Male	29.62	13.73	46.01
**1502**	Current tobacco smoke	Markazi	Khondab	Male	25.02	8.23	40.31
**1503**	Current tobacco smoke	Fars	Khonj	Male	24.52	7.72	40.76
**1504**	Current tobacco smoke	Isfahan	Khoor va Biabanak	Male	23.06	5.87	40.23
**1505**	Current tobacco smoke	Lorestan	Khorramabad	Male	26.76	11.55	42.96
**1506**	Current tobacco smoke	Fars	Khorrambid	Male	31.31	14.92	48.55
**1507**	Current tobacco smoke	Zanjan	Khorramdarreh	Male	24.42	8.60	40.11
**1508**	Current tobacco smoke	Khuzestan	Khorramshahr	Male	27.21	9.45	46.43
**1509**	Current tobacco smoke	Khorasan_razavi	Khoshab	Male	20.56	2.87	38.07
**1510**	Current tobacco smoke	Azarbayjan_West	Khoy	Male	30.92	14.83	47.95
**1511**	Current tobacco smoke	Khorasan_South	Khusef	Male	13.82	0.87	26.93
**1512**	Current tobacco smoke	Chaharmahal	Kiaar	Male	35.16	19.70	52.13
**1513**	Current tobacco smoke	Kohkiluye and Bouyer Ahmad	Kohgiluyeh	Male	23.43	8.18	38.55
**1514**	Current tobacco smoke	Markazi	Komeijan	Male	28.37	9.96	46.40
**1515**	Current tobacco smoke	Sistan and Balouchestan	Konarak	Male	20.18	5.61	35.35
**1516**	Current tobacco smoke	Golestan	Kordkuy	Male	19.48	3.93	35.98
**1517**	Current tobacco smoke	Ardebil	Kowsar	Male	25.31	8.32	42.19
**1518**	Current tobacco smoke	Kerman	Kuhbonan	Male	18.84	2.62	34.16
**1519**	Current tobacco smoke	Lorestan	Kuhdasht	Male	23.60	7.59	40.37
**1520**	Current tobacco smoke	Chaharmahal	Kuhrang	Male	27.34	11.50	44.27
**1521**	Current tobacco smoke	Gilan	Lahijan	Male	27.79	14.89	41.40
**1522**	Current tobacco smoke	Khuzestan	Lali	Male	21.10	3.69	38.46
**1523**	Current tobacco smoke	Fars	Lamard	Male	21.23	6.20	35.36
**1524**	Current tobacco smoke	Kohkiluye and Bouyer Ahmad	Landeh	Male	22.21	0.00	43.68
**1525**	Current tobacco smoke	Gilan	Langrud	Male	24.84	10.60	39.72
**1526**	Current tobacco smoke	Isfahan	Lanjan	Male	28.49	13.86	43.79
**1527**	Current tobacco smoke	Fars	Lar (Larestan)	Male	24.94	10.24	39.33
**1528**	Current tobacco smoke	Chaharmahal	Lordakan	Male	30.58	16.20	45.97
**1529**	Current tobacco smoke	Azarbayjan_West	Mahabad	Male	27.53	11.26	43.32
**1530**	Current tobacco smoke	Markazi	Mahalat	Male	29.90	11.64	48.81
**1531**	Current tobacco smoke	Mazandaran	Mahmudabad	Male	25.75	9.32	42.38
**1532**	Current tobacco smoke	Zanjan	Mahneshan	Male	24.22	10.25	38.70
**1533**	Current tobacco smoke	Khorasan_razavi	Mahvelat	Male	18.56	1.39	36.17
**1534**	Current tobacco smoke	Azarbayjan_West	Maku	Male	28.05	7.93	48.31
**1535**	Current tobacco smoke	Tehran	Malard	Male	25.97	12.31	39.79
**1536**	Current tobacco smoke	Hamedan	Malayer	Male	32.03	15.12	49.97
**1537**	Current tobacco smoke	Azararbayjan_East	Malekan	Male	22.19	8.24	36.00
**1538**	Current tobacco smoke	Ilam	Malekshahi	Male	15.61	0.00	32.26
**1539**	Current tobacco smoke	Fars	Mamasany	Male	26.98	12.83	42.13
**1540**	Current tobacco smoke	Khorasan_North	Maneh and Samalqan	Male	19.35	4.87	33.66
**1541**	Current tobacco smoke	Kerman	Manujan	Male	18.62	4.62	32.24
**1542**	Current tobacco smoke	Azararbayjan_East	Maragheh	Male	24.94	12.89	37.72
**1543**	Current tobacco smoke	Azararbayjan_East	Marand	Male	19.61	6.54	32.20
**1544**	Current tobacco smoke	Golestan	Maravehtapeh	Male	18.36	0.41	36.61
**1545**	Current tobacco smoke	Kordestan	Marivan	Male	28.33	9.15	47.45
**1546**	Current tobacco smoke	Fars	Marvdasht	Male	27.61	13.27	41.41
**1547**	Current tobacco smoke	Gilan	Masal	Male	23.92	9.03	38.75
**1548**	Current tobacco smoke	Khorasan_razavi	Mashhad	Male	17.39	6.00	29.20
**1549**	Current tobacco smoke	Khuzestan	Masjed Soleyman	Male	21.13	4.74	37.44
**1550**	Current tobacco smoke	Semnan	Mayamey	Male	18.90	2.06	35.90
**1551**	Current tobacco smoke	Semnan	Mehdishahr	Male	22.25	9.06	35.45
**1552**	Current tobacco smoke	Ilam	Mehran	Male	17.68	6.97	28.43
**1553**	Current tobacco smoke	Yazd	Mehriz	Male	26.30	11.26	41.98
**1554**	Current tobacco smoke	Ardebil	Meshkinshahr	Male	26.58	11.22	43.16
**1555**	Current tobacco smoke	Yazd	Meybod	Male	28.56	12.35	45.93
**1556**	Current tobacco smoke	Hormozgan	Minab	Male	22.49	7.15	37.31
**1557**	Current tobacco smoke	Golestan	Minudasht	Male	11.82	0.00	24.25
**1558**	Current tobacco smoke	Sistan and Balouchestan	Mirjaveh	Male	19.13	1.59	37.69
**1559**	Current tobacco smoke	Azarbayjan_West	Miyandoab	Male	28.74	13.63	44.83
**1560**	Current tobacco smoke	Mazandaran	Miyandorud	Male	25.13	0.91	48.00
**1561**	Current tobacco smoke	Azararbayjan_East	Miyaneh	Male	22.87	9.61	35.25
**1562**	Current tobacco smoke	Isfahan	Mobarakeh	Male	30.83	14.71	47.76
**1563**	Current tobacco smoke	Fars	Mohr	Male	19.54	8.41	30.99

**1564**	Current tobacco smoke	Hamedan	Nahavand	Male	28.70	11.87	46.51
**1565**	Current tobacco smoke	Isfahan	Najafabad	Male	28.54	13.39	43.51
**1566**	Current tobacco smoke	Ardebil	Namin	Male	23.87	5.07	42.03
**1567**	Current tobacco smoke	Azarbayjan_West	Naqadeh	Male	30.72	13.43	48.85
**1568**	Current tobacco smoke	Kerman	Narmashir	Male	19.69	5.30	33.76
**1569**	Current tobacco smoke	Isfahan	Natanz	Male	25.09	8.83	40.86
**1570**	Current tobacco smoke	Isfahan	Nayin	Male	25.32	9.31	41.47
**1571**	Current tobacco smoke	Alborz	Nazarabad	Male	30.71	12.59	48.48
**1572**	Current tobacco smoke	Ardebil	Neer	Male	26.06	9.26	43.28
**1573**	Current tobacco smoke	Khorasan_South	Nehbandan	Male	9.18	0.00	19.23
**1574**	Current tobacco smoke	Mazandaran	Neka	Male	21.86	3.70	39.05
**1575**	Current tobacco smoke	Fars	Neyriz	Male	26.08	11.65	39.82
**1576**	Current tobacco smoke	Khorasan_razavi	Neyshabur	Male	17.39	4.66	29.71
**1577**	Current tobacco smoke	Sistan and Balouchestan	Nikshahr	Male	15.13	2.90	26.67
**1578**	Current tobacco smoke	Sistan and Balouchestan	Nimruz	Male	14.62	0.00	30.63
**1579**	Current tobacco smoke	Mazandaran	Noshahr	Male	33.44	11.34	57.23
**1580**	Current tobacco smoke	Mazandaran	Nur	Male	29.56	12.27	46.23
**1581**	Current tobacco smoke	Khuzestan	Omidiyeh	Male	23.45	8.76	39.24
**1582**	Current tobacco smoke	Azarbayjan_West	Orumiyeh	Male	31.59	16.67	46.61
**1583**	Current tobacco smoke	Azarbayjan_West	Oshnaviyeh	Male	31.82	13.14	51.18
**1584**	Current tobacco smoke	Azararbayjan_East	Osku	Male	22.98	9.16	36.91
**1585**	Current tobacco smoke	Tehran	Pakdasht	Male	21.81	9.12	34.49
**1586**	Current tobacco smoke	Tehran	Pardis	Male	22.78	6.91	39.65
**1587**	Current tobacco smoke	Ardebil	Parsabad	Male	25.91	7.63	43.81
**1588**	Current tobacco smoke	Hormozgan	Parsian (Gavbandi)	Male	22.75	8.35	36.61
**1589**	Current tobacco smoke	Fars	Pasargad	Male	41.31	25.41	59.34
**1590**	Current tobacco smoke	Kermanshah	Paveh	Male	21.37	2.61	39.80
**1591**	Current tobacco smoke	Azarbayjan_West	Piranshahr	Male	31.38	13.15	50.43
**1592**	Current tobacco smoke	Tehran	Pishva	Male	20.64	6.36	33.98
**1593**	Current tobacco smoke	Azarbayjan_West	Poldasht	Male	28.41	10.81	45.24
**1594**	Current tobacco smoke	Lorestan	Poldokhtar	Male	22.37	5.25	39.35
**1595**	Current tobacco smoke	Mazandaran	Qaemshahr	Male	29.36	13.78	45.95
**1596**	Current tobacco smoke	Tehran	Qarchak	Male	22.51	5.05	40.31
**1597**	Current tobacco smoke	Sistan and Balouchestan	Qasr qand	Male	17.95	1.32	34.39
**1598**	Current tobacco smoke	Kermanshah	Qasr-e-Shirin	Male	16.99	0.00	38.90
**1599**	Current tobacco smoke	Khorasan_South	Qayenat	Male	13.66	1.87	25.80
**1600**	Current tobacco smoke	Qazvin	Qazvin	Male	39.91	22.12	58.02
**1601**	Current tobacco smoke	Hormozgan	Qeshm	Male	23.86	6.40	41.88
**1602**	Current tobacco smoke	Fars	Qirokarzin	Male	21.30	6.33	35.37
**1603**	Current tobacco smoke	Qom	Qom	Male	24.38	8.86	39.98
**1604**	Current tobacco smoke	Kordestan	Qorveh	Male	18.04	2.86	32.99
**1605**	Current tobacco smoke	Khorasan_razavi	Quchan	Male	20.77	3.00	39.11
**1606**	Current tobacco smoke	Kerman	Rabar	Male	47.15	26.71	66.09
**1607**	Current tobacco smoke	Kerman	Rafsanjan	Male	21.48	9.64	34.00
**1608**	Current tobacco smoke	Khuzestan	Ramhormoz	Male	22.01	7.19	37.18
**1609**	Current tobacco smoke	Mazandaran	Ramsar	Male	34.64	14.63	56.44
**1610**	Current tobacco smoke	Khuzestan	Ramshir	Male	24.60	6.81	42.61
**1611**	Current tobacco smoke	Golestan	Ramyan	Male	18.87	2.36	35.32
**1612**	Current tobacco smoke	Gilan	Rasht	Male	23.63	12.01	34.88
**1613**	Current tobacco smoke	Khorasan_razavi	Rashtkhar	Male	16.10	0.44	30.57
**1614**	Current tobacco smoke	Kermanshah	Ravansar	Male	19.67	0.66	38.13
**1615**	Current tobacco smoke	Kerman	Ravar	Male	21.86	11.00	33.22
**1616**	Current tobacco smoke	Khorasan_North	Raz va Jergolan	Male	18.63	0.00	38.71
**1617**	Current tobacco smoke	Hamedan	Razan	Male	31.85	14.21	49.79
**1618**	Current tobacco smoke	Tehran	Rey	Male	23.15	10.10	36.67
**1619**	Current tobacco smoke	Kerman	Reygan	Male	16.73	5.13	27.79
**1620**	Current tobacco smoke	Gilan	Rezvanshahr	Male	23.45	9.05	38.29
**1621**	Current tobacco smoke	Tehran	Robatkarim	Male	13.53	1.41	26.50
**1622**	Current tobacco smoke	Fars	Rostam	Male	23.53	7.37	38.88
**1623**	Current tobacco smoke	Kerman	Roudbar-e-Jonub	Male	17.45	5.43	29.61
**1624**	Current tobacco smoke	Hormozgan	Rudan	Male	13.58	0.56	27.04
**1625**	Current tobacco smoke	Gilan	Rudbar	Male	25.12	11.21	39.86
**1626**	Current tobacco smoke	Gilan	Rudsar	Male	24.83	11.65	38.44
**1627**	Current tobacco smoke	Lorestan	Rumshekan	Male	21.64	0.40	41.88
**1628**	Current tobacco smoke	Khorasan_razavi	Sabzevar	Male	21.14	7.93	34.84
**1629**	Current tobacco smoke	Yazd	Sadugh	Male	27.73	10.11	46.29
**1630**	Current tobacco smoke	Kermanshah	Sahneh	Male	19.07	6.78	31.20
**1631**	Current tobacco smoke	Kermanshah	Salas-e-Babajani	Male	16.81	1.30	31.71
**1632**	Current tobacco smoke	Azarbayjan_West	Salmas	Male	30.03	13.33	47.71
**1633**	Current tobacco smoke	Chaharmahal	Saman	Male	29.54	10.27	48.64
**1634**	Current tobacco smoke	Kordestan	Sanandaj	Male	25.88	10.14	41.69
**1635**	Current tobacco smoke	Kordestan	Saqqez	Male	31.53	13.42	50.69
**1636**	Current tobacco smoke	Kermanshah	Sar-e-Pol-e-Zohab	Male	15.49	0.07	30.28
**1637**	Current tobacco smoke	Azararbayjan_East	Sarab	Male	23.82	10.82	37.12
**1638**	Current tobacco smoke	Khorasan_razavi	Sarakhs	Male	17.70	0.00	36.74
**1639**	Current tobacco smoke	Sistan and Balouchestan	Saravan	Male	16.03	1.35	30.30
**1640**	Current tobacco smoke	Khorasan_South	Sarayan	Male	12.81	0.00	25.83
**1641**	Current tobacco smoke	Sistan and Balouchestan	Sarbaz	Male	16.96	4.39	29.60
**1642**	Current tobacco smoke	Khorasan_South	Sarbisheh	Male	18.17	6.48	30.97
**1643**	Current tobacco smoke	Azarbayjan_West	Sardasht	Male	31.79	13.92	50.91
**1644**	Current tobacco smoke	Ardebil	Sarein	Male	25.54	7.56	43.52
**1645**	Current tobacco smoke	Mazandaran	Sari	Male	28.07	12.78	44.12
**1646**	Current tobacco smoke	Kordestan	Sarvabad	Male	25.86	4.16	47.08
**1647**	Current tobacco smoke	Fars	Sarvestan	Male	26.95	9.43	44.54
**1648**	Current tobacco smoke	Mazandaran	Savadkuh	Male	31.63	14.39	49.87
**1649**	Current tobacco smoke	Mazandaran	Savadkuh_North	Male	28.65	8.52	48.27
**1650**	Current tobacco smoke	Markazi	Saveh	Male	29.83	14.27	45.75
**1651**	Current tobacco smoke	Alborz	Savojbolagh	Male	31.13	14.66	48.61
**1652**	Current tobacco smoke	Lorestan	Selseleh	Male	26.10	8.52	43.99
**1653**	Current tobacco smoke	Isfahan	Semirom	Male	29.92	14.92	45.71
**1654**	Current tobacco smoke	Isfahan	Semirom-e-Sofla	Male	29.99	12.17	49.39
**1655**	Current tobacco smoke	Semnan	Semnan	Male	21.56	6.49	35.80
**1656**	Current tobacco smoke	Fars	Sepidan	Male	29.14	13.57	45.31
**1657**	Current tobacco smoke	Azararbayjan_East	Shabestar	Male	21.65	8.14	34.48
**1658**	Current tobacco smoke	Khuzestan	Shadegan	Male	20.87	5.74	36.05
**1659**	Current tobacco smoke	Gilan	Shaft	Male	26.46	14.02	39.84
**1660**	Current tobacco smoke	Azarbayjan_West	Shahindezh	Male	23.43	8.56	37.80
**1661**	Current tobacco smoke	Tehran	Shahr-e Qods	Male	21.76	9.09	34.33

**1662**	Current tobacco smoke	Kerman	Shahr-e-Babak	Male	18.66	7.98	28.82
**1663**	Current tobacco smoke	Chaharmahal	Shahr-e-Kord	Male	30.70	15.83	45.52
**1664**	Current tobacco smoke	Isfahan	Shahreza	Male	30.72	14.79	48.58
**1665**	Current tobacco smoke	Tehran	Shahriyar	Male	25.20	12.25	38.71
**1666**	Current tobacco smoke	Semnan	Shahrud	Male	21.04	7.67	34.46
**1667**	Current tobacco smoke	Markazi	Shazand	Male	31.38	15.98	47.93
**1668**	Current tobacco smoke	Tehran	Shemiranat	Male	22.71	8.10	36.48
**1669**	Current tobacco smoke	Fars	Shiraz	Male	28.00	15.91	40.77
**1670**	Current tobacco smoke	Khorasan_North	Shirvan	Male	15.43	2.09	28.33
**1671**	Current tobacco smoke	Ilam	Shirvan and Chard-e-Aval	Male	15.51	1.90	29.16
**1672**	Current tobacco smoke	Azarbayjan_West	Showt	Male	31.13	10.68	52.29
**1673**	Current tobacco smoke	Khuzestan	Shush	Male	20.37	5.65	34.99
**1674**	Current tobacco smoke	Khuzestan	Shushtar	Male	21.09	5.85	36.17
**1675**	Current tobacco smoke	Gilan	Siahkal	Male	25.94	11.06	40.73
**1676**	Current tobacco smoke	Sistan and Balouchestan	Sib o Soran	Male	15.10	1.94	28.11
**1677**	Current tobacco smoke	Mazandaran	Simorgh	Male	29.34	8.98	50.97
**1678**	Current tobacco smoke	Hormozgan	Sirik	Male	35.15	20.42	48.06
**1679**	Current tobacco smoke	Kerman	Sirjan	Male	22.59	10.69	34.58
**1680**	Current tobacco smoke	Ilam	Sirvan	Male	14.82	0.00	30.62
**1681**	Current tobacco smoke	Zanjan	Soltaniyeh	Male	24.47	10.00	38.79
**1682**	Current tobacco smoke	Kermanshah	Sonqor	Male	24.52	8.56	41.47
**1683**	Current tobacco smoke	Semnan	Sorkheh	Male	23.29	6.28	39.59
**1684**	Current tobacco smoke	Gilan	Sume'eh Sara	Male	23.92	10.45	37.44
**1685**	Current tobacco smoke	Khorasan_South	Tabas	Male	10.22	0.65	19.27
**1686**	Current tobacco smoke	Azararbayjan_East	Tabriz	Male	23.65	12.63	34.49
**1687**	Current tobacco smoke	Markazi	Tafresh	Male	25.17	7.58	41.84
**1688**	Current tobacco smoke	Yazd	Taft	Male	27.15	15.77	38.81
**1689**	Current tobacco smoke	Azarbayjan_West	Takab	Male	27.68	10.47	45.33
**1690**	Current tobacco smoke	Qazvin	Takestan	Male	38.78	18.03	60.50
**1691**	Current tobacco smoke	Khorasan_razavi	Takht-e-Jolgeh (Firuzeh)	Male	22.68	9.56	35.62
**1692**	Current tobacco smoke	Alborz	Taleghan	Male	34.51	18.70	52.02
**1693**	Current tobacco smoke	Boushehr	Tangestan	Male	26.10	4.93	47.27
**1694**	Current tobacco smoke	Zanjan	Tarom	Male	24.42	11.12	37.48
**1695**	Current tobacco smoke	Gilan	Tavalesh	Male	22.78	9.22	36.32
**1696**	Current tobacco smoke	Khorasan_razavi	Taybad	Male	20.80	3.18	39.71
**1697**	Current tobacco smoke	Tehran	Tehran	Male	22.69	13.16	32.24
**1698**	Current tobacco smoke	Isfahan	Tiran and Karvan	Male	28.17	11.75	45.54
**1699**	Current tobacco smoke	Mazandaran	Tonekabon	Male	35.60	17.18	55.27
**1700**	Current tobacco smoke	Khorasan_razavi	Torbat-e-Heydariyeh	Male	18.86	5.01	33.27
**1701**	Current tobacco smoke	Khorasan_razavi	Torbat-e-Jam	Male	18.84	4.20	33.76
**1702**	Current tobacco smoke	Hamedan	Tuyserkan	Male	25.59	9.84	40.73
**1703**	Current tobacco smoke	Tehran	Varamin	Male	22.87	11.01	35.14
**1704**	Current tobacco smoke	Azararbayjan_East	Varzaqan	Male	23.22	9.12	38.05
**1705**	Current tobacco smoke	Yazd	Yazd	Male	25.48	12.45	38.62
**1706**	Current tobacco smoke	Sistan and Balouchestan	Zabol	Male	9.10	0.00	21.22
**1707**	Current tobacco smoke	Sistan and Balouchestan	Zaboli (Mehrestan)	Male	18.13	8.41	27.82
**1708**	Current tobacco smoke	Sistan and Balouchestan	Zahedan	Male	18.31	6.55	29.89
**1709**	Current tobacco smoke	Zanjan	Zanjan	Male	25.17	14.08	36.68
**1710**	Current tobacco smoke	Kerman	Zarand	Male	12.23	0.51	23.92
**1711**	Current tobacco smoke	Markazi	Zarandiyeh	Male	32.14	16.18	49.05
**1712**	Current tobacco smoke	Fars	Zarrindasht	Male	26.56	9.15	44.08
**1713**	Current tobacco smoke	Khorasan_razavi	Zave	Male	17.54	1.49	34.13
**1714**	Current tobacco smoke	Sistan and Balouchestan	Zehak	Male	13.24	0.00	31.76
**1715**	Current tobacco smoke	Khorasan_South	Zir kuh	Male	13.00	0.00	27.28
**1716**	Ever cigarette smoke	Khuzestan	Abadan	Female	0.24	0.00	2.27
**1717**	Ever cigarette smoke	Fars	Abadeh	Female	0.87	0.00	7.02
**1718**	Ever cigarette smoke	Yazd	Abarkuh	Female	1.62	0.00	9.96
**1719**	Ever cigarette smoke	Mazandaran	Abbas abad	Female	1.15	0.00	7.96
**1720**	Ever cigarette smoke	Ilam	Abdanan	Female	2.31	0.00	9.69
**1721**	Ever cigarette smoke	Zanjan	Abhar	Female	0.47	0.00	3.00
**1722**	Ever cigarette smoke	Hormozgan	Abumusa	Female	0.84	0.00	7.99
**1723**	Ever cigarette smoke	Qazvin	Abyek	Female	1.13	0.00	6.64
**1724**	Ever cigarette smoke	Azararbayjan_East	Ahar	Female	2.39	0.00	4.92
**1725**	Ever cigarette smoke	Khuzestan	Ahvaz	Female	0.77	0.00	2.70
**1726**	Ever cigarette smoke	Azararbayjan_East	Ajabshir	Female	1.38	0.00	4.82
**1727**	Ever cigarette smoke	Qazvin	Alborz	Female	1.34	0.00	5.70
**1728**	Ever cigarette smoke	Golestan	Aliabad	Female	1.85	0.00	8.81
**1729**	Ever cigarette smoke	Lorestan	Aligudarz	Female	0.33	0.00	2.69
**1730**	Ever cigarette smoke	Gilan	Amlash	Female	0.55	0.00	4.49
**1731**	Ever cigarette smoke	Mazandaran	Amol	Female	1.03	0.00	5.78
**1732**	Ever cigarette smoke	Kerman	Anar	Female	1.01	0.00	5.52
**1733**	Ever cigarette smoke	Kerman	Anbarabad	Female	0.36	0.00	3.29
**1734**	Ever cigarette smoke	Khuzestan	Andika	Female	0.66	0.00	3.33
**1735**	Ever cigarette smoke	Khuzestan	Andimeshk	Female	0.45	0.00	2.28
**1736**	Ever cigarette smoke	Golestan	Aq Qala	Female	0.79	0.00	5.97
**1737**	Ever cigarette smoke	Khuzestan	Aqajari	Female	0.53	0.00	3.36
**1738**	Ever cigarette smoke	Semnan	Aradan	Female	0.66	0.00	4.53
**1739**	Ever cigarette smoke	Markazi	Arak	Female	1.38	0.00	3.44
**1740**	Ever cigarette smoke	Isfahan	Aran and Bidgol	Female	1.16	0.00	4.52
**1741**	Ever cigarette smoke	Ardebil	Ardabil	Female	1.39	0.00	4.09
**1742**	Ever cigarette smoke	Yazd	Ardakan	Female	0.77	0.00	6.57
**1743**	Ever cigarette smoke	Chaharmahal	Ardal	Female	0.12	0.00	1.29
**1744**	Ever cigarette smoke	Isfahan	Ardestan	Female	0.95	0.00	5.30
**1745**	Ever cigarette smoke	Fars	Arsanjan	Female	1.44	0.00	10.36
**1746**	Ever cigarette smoke	Kerman	Arzouyeh	Female	0.75	0.00	4.97
**1747**	Ever cigarette smoke	Hamedan	Asadabad	Female	0.85	0.00	2.58
**1748**	Ever cigarette smoke	Boushehr	Asaluyeh	Female	2.60	0.00	12.94
**1749**	Ever cigarette smoke	Markazi	Ashtiyan	Female	1.56	0.00	4.30
**1750**	Ever cigarette smoke	Gilan	Astaneh-ye-Ashrafiyeh	Female	0.85	0.00	4.44
**1751**	Ever cigarette smoke	Gilan	Astara	Female	0.95	0.00	5.34
**1752**	Ever cigarette smoke	Qazvin	Avaj	Female	1.39	0.00	6.76
**1753**	Ever cigarette smoke	Golestan	Azadshahr	Female	1.11	0.00	8.41
**1754**	Ever cigarette smoke	Azararbayjan_East	Azarshahr	Female	0.74	0.00	3.27
**1755**	Ever cigarette smoke	Lorestan	Azna	Female	0.90	0.00	4.35
**1756**	Ever cigarette smoke	Mazandaran	Babol	Female	1.24	0.00	6.32
**1757**	Ever cigarette smoke	Mazandaran	Babolsar	Female	0.71	0.00	7.61
**1758**	Ever cigarette smoke	Ilam	Badreh	Female	1.85	0.00	9.83
**1759**	Ever cigarette smoke	Yazd	Bafq	Female	0.81	0.00	6.63

**1760**	Ever cigarette smoke	Kerman	Baft	Female	0.55	0.00	3.54
**1761**	Ever cigarette smoke	Khuzestan	Baghemalek	Female	0.38	0.00	2.25
**1762**	Ever cigarette smoke	Yazd	Bahabad	Female	1.15	0.00	9.58
**1763**	Ever cigarette smoke	Hamedan	Bahar	Female	1.87	0.00	4.11
**1764**	Ever cigarette smoke	Tehran	Baharestan (Golestan)	Female	1.92	0.00	6.44
**1765**	Ever cigarette smoke	Kohkiluye and Bouyer Ahmad	Bahmani	Female	1.02	0.00	6.38
**1766**	Ever cigarette smoke	Khorasan_razavi	Bajestan	Female	0.60	0.00	5.63
**1767**	Ever cigarette smoke	Khorasan_razavi	Bakhraz	Female	0.34	0.00	3.94
**1768**	Ever cigarette smoke	Kerman	Bam	Female	0.46	0.00	3.45
**1769**	Ever cigarette smoke	Hormozgan	Bandar-e-Abbas	Female	0.83	0.00	5.19
**1770**	Ever cigarette smoke	Gilan	Bandar-e-Anzali	Female	0.97	0.00	3.93
**1771**	Ever cigarette smoke	Golestan	Bandar-e-Gaz	Female	1.45	0.00	9.40
**1772**	Ever cigarette smoke	Hormozgan	Bandar-e-Jask	Female	0.57	0.00	6.94
**1773**	Ever cigarette smoke	Hormozgan	Bandar-e-Lengeh	Female	1.42	0.00	6.61
**1774**	Ever cigarette smoke	Khuzestan	Bandar-e-Mahshahr	Female	0.33	0.00	2.23
**1775**	Ever cigarette smoke	Golestan	Bandar-e-Torkaman	Female	1.41	0.00	9.03
**1776**	Ever cigarette smoke	Kordestan	Baneh	Female	4.99	1.05	8.90
**1777**	Ever cigarette smoke	Khorasan_razavi	Bardeskan	Female	0.59	0.00	5.55
**1778**	Ever cigarette smoke	Kerman	Bardsir	Female	2.91	0.00	5.94
**1779**	Ever cigarette smoke	Hormozgan	Bashagerd	Female	0.51	0.00	6.72
**1780**	Ever cigarette smoke	Kohkiluye and Bouyer Ahmad	Basht	Female	1.77	0.00	9.54
**1781**	Ever cigarette smoke	Hormozgan	Bastak	Female	0.99	0.00	7.12
**1782**	Ever cigarette smoke	Khuzestan	Bavi	Female	0.70	0.00	3.50
**1783**	Ever cigarette smoke	Khuzestan	Behbahan	Female	0.35	0.00	2.24
**1784**	Ever cigarette smoke	Mazandaran	Behshahr	Female	0.27	0.00	5.18
**1785**	Ever cigarette smoke	Kordestan	Bijar	Female	2.83	0.00	7.61
**1786**	Ever cigarette smoke	Ardebil	Bilehsavar	Female	2.30	0.00	6.43
**1787**	Ever cigarette smoke	Khorasan_razavi	Binaloud	Female	0.52	0.00	5.75
**1788**	Ever cigarette smoke	Khorasan_South	Birjand	Female	2.85	0.00	9.63
**1789**	Ever cigarette smoke	Khorasan_North	Bojnurd	Female	0.93	0.00	5.40
**1790**	Ever cigarette smoke	Chaharmahal	Bon	Female	0.28	0.00	2.12
**1791**	Ever cigarette smoke	Azararbayjan_East	Bonab	Female	0.85	0.00	3.22
**1792**	Ever cigarette smoke	Isfahan	Borkhar	Female	0.56	0.00	3.65
**1793**	Ever cigarette smoke	Isfahan	Borkhar and Meymeh	Female	1.38	0.00	5.11
**1794**	Ever cigarette smoke	Chaharmahal	Borujen	Female	0.13	0.00	1.28
**1795**	Ever cigarette smoke	Lorestan	Borujerd	Female	0.45	0.00	2.82
**1796**	Ever cigarette smoke	Khorasan_South	Boshruyeh	Female	2.11	0.00	11.58
**1797**	Ever cigarette smoke	Azararbayjan_East	Bostanabad	Female	1.05	0.00	3.33
**1798**	Ever cigarette smoke	Fars	Bovanat	Female	1.65	0.00	10.36
**1799**	Ever cigarette smoke	Kohkiluye and Bouyer Ahmad	Boyer Ahmad	Female	1.99	0.00	7.63
**1800**	Ever cigarette smoke	Qazvin	Boyinzahra	Female	0.75	0.00	4.60
**1801**	Ever cigarette smoke	Isfahan	Buein va Miandasht	Female	1.07	0.00	5.51
**1802**	Ever cigarette smoke	Azarbayjan_West	Bukan	Female	2.81	0.00	6.83
**1803**	Ever cigarette smoke	Boushehr	Bushehr	Female	3.15	0.00	11.89
**1804**	Ever cigarette smoke	Isfahan	Chadegan	Female	0.98	0.00	5.45
**1805**	Ever cigarette smoke	Sistan and Balouchestan	Chahbahar	Female	5.18	0.00	23.46
**1806**	Ever cigarette smoke	Azarbayjan_West	Chaipareh	Female	2.52	0.00	7.35
**1807**	Ever cigarette smoke	Azarbayjan_West	Chaldoran	Female	2.48	0.00	7.49
**1808**	Ever cigarette smoke	Mazandaran	Chalus	Female	0.49	0.00	5.15
**1809**	Ever cigarette smoke	Azararbayjan_East	Charoimaq	Female	1.74	0.00	4.81
**1810**	Ever cigarette smoke	Khorasan_razavi	Chenaran	Female	0.28	0.00	3.91
**1811**	Ever cigarette smoke	Kohkiluye and Bouyer Ahmad	Cheram	Female	1.80	0.00	9.46
**1812**	Ever cigarette smoke	Kermanshah	Dalaho	Female	1.62	0.00	7.30
**1813**	Ever cigarette smoke	Lorestan	Dalfan	Female	0.30	0.00	2.67
**1814**	Ever cigarette smoke	Sistan and Balouchestan	Dalgan	Female	2.76	0.00	21.73
**1815**	Ever cigarette smoke	Tehran	Damavand	Female	0.69	0.00	4.26
**1816**	Ever cigarette smoke	Semnan	Damghan	Female	0.32	0.00	2.94
**1817**	Ever cigarette smoke	Fars	Darab	Female	2.02	0.00	9.78
**1818**	Ever cigarette smoke	Khorasan_South	Darmian	Female	3.37	0.00	12.63
**1819**	Ever cigarette smoke	Khorasan_razavi	Darrehgaz	Female	0.32	0.00	4.15
**1820**	Ever cigarette smoke	Ilam	Darrehshahr	Female	1.06	0.00	7.14
**1821**	Ever cigarette smoke	Khuzestan	Dasht-e-Azadegan	Female	0.44	0.00	2.37
**1822**	Ever cigarette smoke	Boushehr	Dashtestan	Female	1.66	0.00	8.45
**1823**	Ever cigarette smoke	Boushehr	Dashti	Female	4.75	0.00	13.80
**1824**	Ever cigarette smoke	Khorasan_razavi	Davarzan	Female	0.46	0.00	5.58
**1825**	Ever cigarette smoke	Boushehr	Dayyer	Female	2.97	0.00	13.83
**1826**	Ever cigarette smoke	Kordestan	Dehgolan	Female	2.76	0.00	7.76
**1827**	Ever cigarette smoke	Ilam	Dehloran	Female	3.18	0.00	10.12
**1828**	Ever cigarette smoke	Markazi	Delijan	Female	1.59	0.00	4.07
**1829**	Ever cigarette smoke	Kohkiluye and Bouyer Ahmad	Dena	Female	0.93	0.00	6.27
**1830**	Ever cigarette smoke	Boushehr	Deylam	Female	2.42	0.00	12.74
**1831**	Ever cigarette smoke	Khuzestan	Dezful	Female	0.97	0.00	3.13
**1832**	Ever cigarette smoke	Kordestan	Divandarreh	Female	3.09	0.00	7.84
**1833**	Ever cigarette smoke	Lorestan	Dorud	Female	0.91	0.00	3.53
**1834**	Ever cigarette smoke	Lorestan	Doureh	Female	0.49	0.00	3.73
**1835**	Ever cigarette smoke	Fars	Eqlid	Female	1.37	0.00	9.50
**1836**	Ever cigarette smoke	Khorasan_North	Esfarayen	Female	0.87	0.00	5.42
**1837**	Ever cigarette smoke	Alborz	Eshtehard	Female	0.98	0.00	5.01
**1838**	Ever cigarette smoke	Kermanshah	Eslamabad-e-Gharb	Female	2.19	0.00	7.60
**1839**	Ever cigarette smoke	Tehran	Eslamshahr	Female	0.79	0.00	4.57
**1840**	Ever cigarette smoke	Fars	Estahban	Female	1.44	0.00	10.38
**1841**	Ever cigarette smoke	Ilam	Eyvan	Female	2.59	0.00	10.55
**1842**	Ever cigarette smoke	Kerman	Fahraj	Female	0.93	0.00	5.21
**1843**	Ever cigarette smoke	Isfahan	Falavarjan	Female	0.60	0.00	3.70
**1844**	Ever cigarette smoke	Hamedan	Famenin	Female	6.91	4.15	9.91
**1845**	Ever cigarette smoke	Markazi	Farahan	Female	1.66	0.00	4.41
**1846**	Ever cigarette smoke	Fars	Farashband	Female	1.26	0.00	9.69
**1847**	Ever cigarette smoke	Alborz	Fardis	Female	0.93	0.00	5.16
**1848**	Ever cigarette smoke	Isfahan	Faridan	Female	0.65	0.00	3.77
**1849**	Ever cigarette smoke	Khorasan_razavi	Fariman	Female	0.32	0.00	3.96
**1850**	Ever cigarette smoke	Khorasan_North	Faroj	Female	0.42	0.00	4.90
**1851**	Ever cigarette smoke	Chaharmahal	Farsan	Female	0.15	0.00	1.32
**1852**	Ever cigarette smoke	Kerman	Faryab	Female	0.71	0.00	4.82
**1853**	Ever cigarette smoke	Fars	Fasa	Female	0.76	0.00	6.93
**1854**	Ever cigarette smoke	Khorasan_South	Ferdows	Female	2.12	0.00	11.70
**1855**	Ever cigarette smoke	Mazandaran	Fereydunkenar	Female	0.40	0.00	5.26
**1856**	Ever cigarette smoke	Isfahan	Fereydunshahr	Female	0.98	0.00	5.33
**1857**	Ever cigarette smoke	Fars	Firozabad	Female	0.67	0.00	6.69

**1858**	Ever cigarette smoke	Tehran	Firuzkuh	Female	0.68	0.00	4.26
**1859**	Ever cigarette smoke	Sistan and Balouchestan	Fonuj	Female	3.00	0.00	22.25
**1860**	Ever cigarette smoke	Gilan	Fuman	Female	0.35	0.00	2.97
**1861**	Ever cigarette smoke	Kohkiluye and Bouyer Ahmad	Gachsaran	Female	2.60	0.00	9.36
**1862**	Ever cigarette smoke	Golestan	Galikesh	Female	0.68	0.00	5.78
**1863**	Ever cigarette smoke	Mazandaran	Galugah	Female	0.44	0.00	8.22
**1864**	Ever cigarette smoke	Semnan	Garmsar	Female	0.46	0.00	3.18
**1865**	Ever cigarette smoke	Boushehr	Genaveh	Female	1.40	0.00	8.66
**1866**	Ever cigarette smoke	Fars	Gerash	Female	1.10	0.00	10.52
**1867**	Ever cigarette smoke	Khorasan_North	Germeh	Female	0.86	0.00	7.18
**1868**	Ever cigarette smoke	Ardebil	Germi	Female	2.30	0.00	5.78
**1869**	Ever cigarette smoke	Kerman	Ghaleye-Ganj	Female	0.39	0.00	3.33
**1870**	Ever cigarette smoke	Kermanshah	Gilan-e-Gharb	Female	1.52	0.00	7.01
**1871**	Ever cigarette smoke	Isfahan	Golpayegan	Female	1.36	0.00	5.66
**1872**	Ever cigarette smoke	Golestan	Gomishan	Female	1.34	0.00	9.96
**1873**	Ever cigarette smoke	Khorasan_razavi	Gonabad	Female	0.65	0.00	5.85
**1874**	Ever cigarette smoke	Golestan	Gonbad-e-Kavus	Female	0.73	0.00	5.72
**1875**	Ever cigarette smoke	Golestan	Gorgan	Female	2.29	0.00	8.32
**1876**	Ever cigarette smoke	Khuzestan	Guotvand	Female	0.74	0.00	3.52
**1877**	Ever cigarette smoke	Khuzestan	Haftgol	Female	0.67	0.00	3.28
**1878**	Ever cigarette smoke	Hormozgan	Hajiabad	Female	0.57	0.00	4.65
**1879**	Ever cigarette smoke	Hamedan	Hamadan	Female	1.43	0.00	3.35
**1880**	Ever cigarette smoke	Khuzestan	Hamidiyeh	Female	0.69	0.00	3.40
**1881**	Ever cigarette smoke	Sistan and Balouchestan	Hamoon	Female	3.71	0.00	23.80
**1882**	Ever cigarette smoke	Azararbayjan_East	Haris	Female	2.62	0.00	5.45
**1883**	Ever cigarette smoke	Kermanshah	Harsin	Female	1.63	0.00	7.56
**1884**	Ever cigarette smoke	Azararbayjan_East	Hashtrud	Female	1.61	0.00	4.89
**1885**	Ever cigarette smoke	Khuzestan	Hendijan	Female	0.57	0.00	3.21
**1886**	Ever cigarette smoke	Sistan and Balouchestan	Hirmand	Female	4.99	0.00	25.99
**1887**	Ever cigarette smoke	Khuzestan	Hoveizeh	Female	0.70	0.00	3.52
**1888**	Ever cigarette smoke	Zanjan	Ijerod	Female	1.04	0.00	5.01
**1889**	Ever cigarette smoke	Ilam	Ilam	Female	1.60	0.00	7.64
**1890**	Ever cigarette smoke	Sistan and Balouchestan	Iranshahr	Female	1.77	0.00	14.89
**1891**	Ever cigarette smoke	Isfahan	Isfahan	Female	1.08	0.00	4.20
**1892**	Ever cigarette smoke	Khuzestan	Izeh	Female	0.37	0.00	2.19
**1893**	Ever cigarette smoke	Fars	Jahrom	Female	0.71	0.00	6.83
**1894**	Ever cigarette smoke	Khorasan_North	Jajarm	Female	1.15	0.00	7.06
**1895**	Ever cigarette smoke	Boushehr	Jam	Female	1.68	0.00	8.87
**1896**	Ever cigarette smoke	Kermanshah	Javanrud	Female	2.58	0.00	8.04
**1897**	Ever cigarette smoke	Kerman	Jiroft	Female	0.50	0.00	3.33
**1898**	Ever cigarette smoke	Khorasan_razavi	Joghatai	Female	0.46	0.00	5.51
**1899**	Ever cigarette smoke	Azararbayjan_East	Jolfa	Female	2.31	0.00	5.65
**1900**	Ever cigarette smoke	Khorasan_razavi	Jowayin	Female	0.38	0.00	5.58
**1901**	Ever cigarette smoke	Mazandaran	Juybar	Female	0.81	0.00	7.86
**1902**	Ever cigarette smoke	Hamedan	Kabudarahang	Female	1.05	0.00	2.82
**1903**	Ever cigarette smoke	Kerman	Kahnuj	Female	0.65	0.00	4.65
**1904**	Ever cigarette smoke	Golestan	Kalaleh	Female	1.79	0.00	8.62
**1905**	Ever cigarette smoke	Khorasan_razavi	Kalat	Female	0.51	0.00	6.03
**1906**	Ever cigarette smoke	Azararbayjan_East	Kaleibar	Female	9.40	5.72	13.03
**1907**	Ever cigarette smoke	Kordestan	Kamyaran	Female	2.94	0.00	7.37
**1908**	Ever cigarette smoke	Boushehr	Kangan	Female	3.78	0.00	13.79
**1909**	Ever cigarette smoke	Kermanshah	Kangavar	Female	0.91	0.00	4.90
**1910**	Ever cigarette smoke	Alborz	Karaj	Female	0.84	0.00	3.84
**1911**	Ever cigarette smoke	Khuzestan	Karun	Female	0.57	0.00	3.56
**1912**	Ever cigarette smoke	Isfahan	Kashan	Female	0.65	0.00	3.78
**1913**	Ever cigarette smoke	Khorasan_razavi	Kashmar	Female	0.30	0.00	3.96
**1914**	Ever cigarette smoke	Fars	Kavar	Female	1.07	0.00	10.00
**1915**	Ever cigarette smoke	Fars	Kazerun	Female	0.67	0.00	6.78
**1916**	Ever cigarette smoke	Mazandaran	Kelardasht	Female	0.84	0.00	7.93
**1917**	Ever cigarette smoke	Kerman	Kerman	Female	0.79	0.00	3.80
**1918**	Ever cigarette smoke	Kermanshah	Kermanshah	Female	1.76	0.00	5.80
**1919**	Ever cigarette smoke	Khorasan_razavi	Khaf	Female	0.36	0.00	3.89
**1920**	Ever cigarette smoke	Khorasan_razavi	Khalilabad	Female	0.58	0.00	5.81
**1921**	Ever cigarette smoke	Ardebil	Khalkhal	Female	0.90	0.00	3.58
**1922**	Ever cigarette smoke	Hormozgan	Khamir	Female	0.89	0.00	7.06
**1923**	Ever cigarette smoke	Isfahan	Khansar	Female	1.17	0.00	5.75
**1924**	Ever cigarette smoke	Sistan and Balouchestan	Khash	Female	2.21	0.00	15.65
**1925**	Ever cigarette smoke	Yazd	Khatam	Female	2.22	0.00	10.35
**1926**	Ever cigarette smoke	Fars	Kherameh	Female	1.33	0.00	9.99
**1927**	Ever cigarette smoke	Azararbayjan_East	Khodaafarin	Female	3.04	0.00	6.57
**1928**	Ever cigarette smoke	Zanjan	Khodabandeh	Female	1.09	0.00	3.97
**1929**	Ever cigarette smoke	Markazi	Khomeyn	Female	0.93	0.00	2.86
**1930**	Ever cigarette smoke	Isfahan	Khomeynishahr	Female	1.37	0.00	4.68
**1931**	Ever cigarette smoke	Markazi	Khondab	Female	1.04	0.00	2.98
**1932**	Ever cigarette smoke	Fars	Khonj	Female	1.12	0.00	9.46
**1933**	Ever cigarette smoke	Isfahan	Khoor va Biabanak	Female	0.77	0.00	5.23
**1934**	Ever cigarette smoke	Lorestan	Khorramabad	Female	0.49	0.00	2.78
**1935**	Ever cigarette smoke	Fars	Khorrambid	Female	1.58	0.00	10.30
**1936**	Ever cigarette smoke	Zanjan	Khorramdarreh	Female	0.44	0.00	3.07
**1937**	Ever cigarette smoke	Khuzestan	Khorramshahr	Female	0.27	0.00	2.24
**1938**	Ever cigarette smoke	Khorasan_razavi	Khoshab	Female	0.45	0.00	5.49
**1939**	Ever cigarette smoke	Azarbayjan_West	Khoy	Female	1.28	0.00	4.70
**1940**	Ever cigarette smoke	Khorasan_South	Khusef	Female	2.21	0.00	11.27
**1941**	Ever cigarette smoke	Chaharmahal	Kiaar	Female	0.14	0.00	1.35
**1942**	Ever cigarette smoke	Kohkiluye and Bouyer Ahmad	Kohgiluyeh	Female	2.37	0.00	8.58
**1943**	Ever cigarette smoke	Markazi	Komeijan	Female	1.84	0.00	4.54
**1944**	Ever cigarette smoke	Sistan and Balouchestan	Konarak	Female	3.22	0.00	22.28
**1945**	Ever cigarette smoke	Golestan	Kordkuy	Female	1.35	0.00	8.96
**1946**	Ever cigarette smoke	Ardebil	Kowsar	Female	1.00	0.00	4.43
**1947**	Ever cigarette smoke	Kerman	Kuhbonan	Female	0.78	0.00	5.09
**1948**	Ever cigarette smoke	Lorestan	Kuhdasht	Female	0.29	0.00	2.73
**1949**	Ever cigarette smoke	Chaharmahal	Kuhrang	Female	0.29	0.00	1.87
**1950**	Ever cigarette smoke	Gilan	Lahijan	Female	0.31	0.00	2.98
**1951**	Ever cigarette smoke	Khuzestan	Lali	Female	0.74	0.00	3.45
**1952**	Ever cigarette smoke	Fars	Lamard	Female	0.74	0.00	7.09
**1953**	Ever cigarette smoke	Kohkiluye and Bouyer Ahmad	Landeh	Female	1.71	0.00	10.18
**1954**	Ever cigarette smoke	Gilan	Langrud	Female	0.29	0.00	3.01
**1955**	Ever cigarette smoke	Isfahan	Lanjan	Female	0.56	0.00	3.61

**1956**	Ever cigarette smoke	Fars	Lar (Larestan)	Female	0.65	0.00	6.70
**1957**	Ever cigarette smoke	Chaharmahal	Lordakan	Female	0.35	0.00	1.67
**1958**	Ever cigarette smoke	Azarbayjan_West	Mahabad	Female	2.59	0.00	6.84
**1959**	Ever cigarette smoke	Markazi	Mahalat	Female	1.38	0.00	3.57
**1960**	Ever cigarette smoke	Mazandaran	Mahmudabad	Female	0.44	0.00	5.45
**1961**	Ever cigarette smoke	Zanjan	Mahneshan	Female	1.05	0.00	4.82
**1962**	Ever cigarette smoke	Khorasan_razavi	Mahvelat	Female	0.63	0.00	5.61
**1963**	Ever cigarette smoke	Azarbayjan_West	Maku	Female	3.00	0.00	8.03
**1964**	Ever cigarette smoke	Tehran	Malard	Female	1.36	0.00	5.38
**1965**	Ever cigarette smoke	Hamedan	Malayer	Female	0.91	0.00	2.69
**1966**	Ever cigarette smoke	Azararbayjan_East	Malekan	Female	0.86	0.00	3.29
**1967**	Ever cigarette smoke	Ilam	Malekshahi	Female	1.92	0.00	10.53
**1968**	Ever cigarette smoke	Fars	Mamasany	Female	0.77	0.00	6.83
**1969**	Ever cigarette smoke	Khorasan_North	Maneh and Samalqan	Female	0.49	0.00	4.89
**1970**	Ever cigarette smoke	Kerman	Manujan	Female	0.65	0.00	5.00
**1971**	Ever cigarette smoke	Azararbayjan_East	Maragheh	Female	0.95	0.00	3.16
**1972**	Ever cigarette smoke	Azararbayjan_East	Marand	Female	1.21	0.00	3.56
**1973**	Ever cigarette smoke	Golestan	Maravehtapeh	Female	1.21	0.00	9.05
**1974**	Ever cigarette smoke	Kordestan	Marivan	Female	2.48	0.00	6.39
**1975**	Ever cigarette smoke	Fars	Marvdasht	Female	1.65	0.00	8.18
**1976**	Ever cigarette smoke	Gilan	Masal	Female	0.68	0.00	4.46
**1977**	Ever cigarette smoke	Khorasan_razavi	Mashhad	Female	0.92	0.00	4.50
**1978**	Ever cigarette smoke	Khuzestan	Masjed Soleyman	Female	0.68	0.00	3.19
**1979**	Ever cigarette smoke	Semnan	Mayamey	Female	0.33	0.00	4.22
**1980**	Ever cigarette smoke	Semnan	Mehdishahr	Female	0.57	0.00	4.31
**1981**	Ever cigarette smoke	Ilam	Mehran	Female	1.04	0.00	7.21
**1982**	Ever cigarette smoke	Yazd	Mehriz	Female	1.58	0.00	9.67
**1983**	Ever cigarette smoke	Ardebil	Meshkinshahr	Female	1.58	0.00	4.30
**1984**	Ever cigarette smoke	Yazd	Meybod	Female	0.85	0.00	6.92
**1985**	Ever cigarette smoke	Hormozgan	Minab	Female	0.40	0.00	4.68
**1986**	Ever cigarette smoke	Golestan	Minudasht	Female	0.66	0.00	5.87
**1987**	Ever cigarette smoke	Sistan and Balouchestan	Mirjaveh	Female	3.61	0.00	24.60
**1988**	Ever cigarette smoke	Azarbayjan_West	Miyandoab	Female	1.31	0.00	4.56
**1989**	Ever cigarette smoke	Mazandaran	Miyandorud	Female	0.63	0.00	8.45
**1990**	Ever cigarette smoke	Azararbayjan_East	Miyaneh	Female	1.04	0.00	3.33
**1991**	Ever cigarette smoke	Isfahan	Mobarakeh	Female	1.34	0.00	5.00
**1992**	Ever cigarette smoke	Fars	Mohr	Female	1.21	0.00	10.10
**1993**	Ever cigarette smoke	Hamedan	Nahavand	Female	0.82	0.00	2.59
**1994**	Ever cigarette smoke	Isfahan	Najafabad	Female	1.49	0.00	5.39
**1995**	Ever cigarette smoke	Ardebil	Namin	Female	1.09	0.00	4.27
**1996**	Ever cigarette smoke	Azarbayjan_West	Naqadeh	Female	2.55	0.00	6.85
**1997**	Ever cigarette smoke	Kerman	Narmashir	Female	0.69	0.00	5.01
**1998**	Ever cigarette smoke	Isfahan	Natanz	Female	1.21	0.00	4.87
**1999**	Ever cigarette smoke	Isfahan	Nayin	Female	0.85	0.00	5.06
**2000**	Ever cigarette smoke	Alborz	Nazarabad	Female	1.22	0.00	4.54
**2001**	Ever cigarette smoke	Ardebil	Neer	Female	1.10	0.00	4.54
**2002**	Ever cigarette smoke	Khorasan_South	Nehbandan	Female	1.26	0.00	7.75
**2003**	Ever cigarette smoke	Mazandaran	Neka	Female	0.35	0.00	5.32
**2004**	Ever cigarette smoke	Fars	Neyriz	Female	2.26	0.00	9.45
**2005**	Ever cigarette smoke	Khorasan_razavi	Neyshabur	Female	0.29	0.00	3.77
**2006**	Ever cigarette smoke	Sistan and Balouchestan	Nikshahr	Female	1.85	0.00	15.03
**2007**	Ever cigarette smoke	Sistan and Balouchestan	Nimruz	Female	3.74	0.00	23.60
**2008**	Ever cigarette smoke	Mazandaran	Noshahr	Female	0.38	0.00	5.46
**2009**	Ever cigarette smoke	Mazandaran	Nur	Female	0.49	0.00	5.22
**2010**	Ever cigarette smoke	Khuzestan	Omidiyeh	Female	0.57	0.00	3.14
**2011**	Ever cigarette smoke	Azarbayjan_West	Orumiyeh	Female	2.63	0.00	6.73
**2012**	Ever cigarette smoke	Azarbayjan_West	Oshnaviyeh	Female	2.42	0.00	7.64
**2013**	Ever cigarette smoke	Azararbayjan_East	Osku	Female	1.53	0.00	4.84
**2014**	Ever cigarette smoke	Tehran	Pakdasht	Female	0.77	0.00	4.42
**2015**	Ever cigarette smoke	Tehran	Pardis	Female	1.18	0.00	6.58
**2016**	Ever cigarette smoke	Ardebil	Parsabad	Female	1.47	0.00	4.09
**2017**	Ever cigarette smoke	Hormozgan	Parsian (Gavbandi)	Female	0.94	0.00	7.39
**2018**	Ever cigarette smoke	Fars	Pasargad	Female	1.97	0.00	10.21
**2019**	Ever cigarette smoke	Kermanshah	Paveh	Female	2.01	0.00	7.84
**2020**	Ever cigarette smoke	Azarbayjan_West	Piranshahr	Female	2.32	0.00	7.25
**2021**	Ever cigarette smoke	Tehran	Pishva	Female	1.83	0.00	6.58
**2022**	Ever cigarette smoke	Azarbayjan_West	Poldasht	Female	2.93	0.00	7.85
**2023**	Ever cigarette smoke	Lorestan	Poldokhtar	Female	0.30	0.00	2.75
**2024**	Ever cigarette smoke	Mazandaran	Qaemshahr	Female	0.42	0.00	5.31
**2025**	Ever cigarette smoke	Tehran	Qarchak	Female	1.21	0.00	6.53
**2026**	Ever cigarette smoke	Sistan and Balouchestan	Qasr qand	Female	3.21	0.00	23.04
**2027**	Ever cigarette smoke	Kermanshah	Qasr-e-Shirin	Female	1.48	0.00	8.02
**2028**	Ever cigarette smoke	Khorasan_South	Qayenat	Female	2.75	0.00	9.93
**2029**	Ever cigarette smoke	Qazvin	Qazvin	Female	1.44	0.00	5.64
**2030**	Ever cigarette smoke	Hormozgan	Qeshm	Female	0.50	0.00	5.02
**2031**	Ever cigarette smoke	Fars	Qirokarzin	Female	0.66	0.00	6.86
**2032**	Ever cigarette smoke	Qom	Qom	Female	1.51	0.00	6.99
**2033**	Ever cigarette smoke	Kordestan	Qorveh	Female	3.04	0.00	7.71
**2034**	Ever cigarette smoke	Khorasan_razavi	Quchan	Female	0.73	0.00	4.40
**2035**	Ever cigarette smoke	Kerman	Rabar	Female	1.32	0.00	5.13
**2036**	Ever cigarette smoke	Kerman	Rafsanjan	Female	0.61	0.00	3.58
**2037**	Ever cigarette smoke	Khuzestan	Ramhormoz	Female	0.83	0.00	3.34
**2038**	Ever cigarette smoke	Mazandaran	Ramsar	Female	1.13	0.00	8.32
**2039**	Ever cigarette smoke	Khuzestan	Ramshir	Female	0.67	0.00	3.27
**2040**	Ever cigarette smoke	Golestan	Ramyan	Female	1.30	0.00	8.78
**2041**	Ever cigarette smoke	Gilan	Rasht	Female	0.34	0.00	2.89
**2042**	Ever cigarette smoke	Khorasan_razavi	Rashtkhar	Female	0.60	0.00	5.54
**2043**	Ever cigarette smoke	Kermanshah	Ravansar	Female	1.96	0.00	7.44
**2044**	Ever cigarette smoke	Kerman	Ravar	Female	0.48	0.00	3.47
**2045**	Ever cigarette smoke	Khorasan_North	Raz va Jergolan	Female	0.81	0.00	7.79
**2046**	Ever cigarette smoke	Hamedan	Razan	Female	1.06	0.00	2.80
**2047**	Ever cigarette smoke	Tehran	Rey	Female	1.46	0.00	5.41
**2048**	Ever cigarette smoke	Kerman	Reygan	Female	0.67	0.00	4.67
**2049**	Ever cigarette smoke	Gilan	Rezvanshahr	Female	0.70	0.00	4.41
**2050**	Ever cigarette smoke	Tehran	Robatkarim	Female	0.77	0.00	4.60
**2051**	Ever cigarette smoke	Fars	Rostam	Female	0.62	0.00	6.83
**2052**	Ever cigarette smoke	Kerman	Roudbar-e-Jonub	Female	0.40	0.00	3.35
**2053**	Ever cigarette smoke	Hormozgan	Rudan	Female	0.43	0.00	4.48

**2054**	Ever cigarette smoke	Gilan	Rudbar	Female	0.36	0.00	2.95
**2055**	Ever cigarette smoke	Gilan	Rudsar	Female	0.87	0.00	3.82
**2056**	Ever cigarette smoke	Lorestan	Rumshekan	Female	0.44	0.00	3.92
**2057**	Ever cigarette smoke	Khorasan_razavi	Sabzevar	Female	0.31	0.00	3.82
**2058**	Ever cigarette smoke	Yazd	Sadugh	Female	1.60	0.00	10.09
**2059**	Ever cigarette smoke	Kermanshah	Sahneh	Female	1.59	0.00	6.98
**2060**	Ever cigarette smoke	Kermanshah	Salas-e-Babajani	Female	1.74	0.00	7.39
**2061**	Ever cigarette smoke	Azarbayjan_West	Salmas	Female	1.18	0.00	4.69
**2062**	Ever cigarette smoke	Chaharmahal	Saman	Female	0.26	0.00	1.94
**2063**	Ever cigarette smoke	Kordestan	Sanandaj	Female	2.67	0.00	6.62
**2064**	Ever cigarette smoke	Kordestan	Saqqez	Female	3.53	0.00	8.07
**2065**	Ever cigarette smoke	Kermanshah	Sar-e-Pol-e-Zohab	Female	0.93	0.00	4.94
**2066**	Ever cigarette smoke	Azararbayjan_East	Sarab	Female	2.29	0.00	4.86
**2067**	Ever cigarette smoke	Khorasan_razavi	Sarakhs	Female	0.59	0.00	5.97
**2068**	Ever cigarette smoke	Sistan and Balouchestan	Saravan	Female	6.03	0.00	24.46
**2069**	Ever cigarette smoke	Khorasan_South	Sarayan	Female	2.20	0.00	11.44
**2070**	Ever cigarette smoke	Sistan and Balouchestan	Sarbaz	Female	1.85	0.00	14.98
**2071**	Ever cigarette smoke	Khorasan_South	Sarbisheh	Female	3.29	0.00	12.48
**2072**	Ever cigarette smoke	Azarbayjan_West	Sardasht	Female	1.48	0.00	4.76
**2073**	Ever cigarette smoke	Ardebil	Sarein	Female	1.32	0.00	4.81
**2074**	Ever cigarette smoke	Mazandaran	Sari	Female	1.12	0.00	6.17
**2075**	Ever cigarette smoke	Kordestan	Sarvabad	Female	2.76	0.00	7.97
**2076**	Ever cigarette smoke	Fars	Sarvestan	Female	1.24	0.00	9.91
**2077**	Ever cigarette smoke	Mazandaran	Savadkuh	Female	0.82	0.00	7.41
**2078**	Ever cigarette smoke	Mazandaran	Savadkuh_North	Female	0.78	0.00	7.80
**2079**	Ever cigarette smoke	Markazi	Saveh	Female	1.06	0.00	2.98
**2080**	Ever cigarette smoke	Alborz	Savojbolagh	Female	1.10	0.00	4.24
**2081**	Ever cigarette smoke	Lorestan	Selseleh	Female	0.92	0.00	3.77
**2082**	Ever cigarette smoke	Isfahan	Semirom	Female	0.52	0.00	3.57
**2083**	Ever cigarette smoke	Isfahan	Semirom-e-Sofla	Female	0.86	0.00	5.28
**2084**	Ever cigarette smoke	Semnan	Semnan	Female	0.90	0.00	4.11
**2085**	Ever cigarette smoke	Fars	Sepidan	Female	0.74	0.00	6.98
**2086**	Ever cigarette smoke	Azararbayjan_East	Shabestar	Female	1.09	0.00	3.38
**2087**	Ever cigarette smoke	Khuzestan	Shadegan	Female	0.31	0.00	2.19
**2088**	Ever cigarette smoke	Gilan	Shaft	Female	0.34	0.00	2.96
**2089**	Ever cigarette smoke	Azarbayjan_West	Shahindezh	Female	6.26	2.62	10.11
**2090**	Ever cigarette smoke	Tehran	Shahr-e Qods	Female	1.76	0.00	5.93
**2091**	Ever cigarette smoke	Kerman	Shahr-e-Babak	Female	1.27	0.00	5.27
**2092**	Ever cigarette smoke	Chaharmahal	Shahr-e-Kord	Female	0.24	0.00	1.41
**2093**	Ever cigarette smoke	Isfahan	Shahreza	Female	0.89	0.00	4.17
**2094**	Ever cigarette smoke	Tehran	Shahriyar	Female	1.20	0.00	4.95
**2095**	Ever cigarette smoke	Semnan	Shahrud	Female	0.25	0.00	2.78
**2096**	Ever cigarette smoke	Markazi	Shazand	Female	4.89	2.50	7.40
**2097**	Ever cigarette smoke	Tehran	Shemiranat	Female	1.49	0.00	6.42
**2098**	Ever cigarette smoke	Fars	Shiraz	Female	2.43	0.00	9.42
**2099**	Ever cigarette smoke	Khorasan_North	Shirvan	Female	0.75	0.00	5.29
**2100**	Ever cigarette smoke	Ilam	Shirvan and Chard-e-Aval	Female	1.27	0.00	7.26
**2101**	Ever cigarette smoke	Azarbayjan_West	Showt	Female	2.41	0.00	6.39
**2102**	Ever cigarette smoke	Khuzestan	Shush	Female	1.91	0.02	3.84
**2103**	Ever cigarette smoke	Khuzestan	Shushtar	Female	0.95	0.00	3.14
**2104**	Ever cigarette smoke	Gilan	Siahkal	Female	0.53	0.00	4.10
**2105**	Ever cigarette smoke	Sistan and Balouchestan	Sib o Soran	Female	3.93	0.00	24.00
**2106**	Ever cigarette smoke	Mazandaran	Simorgh	Female	0.77	0.00	7.78
**2107**	Ever cigarette smoke	Hormozgan	Sirik	Female	0.80	0.00	7.41
**2108**	Ever cigarette smoke	Kerman	Sirjan	Female	0.86	0.00	3.83
**2109**	Ever cigarette smoke	Ilam	Sirvan	Female	1.82	0.00	10.25
**2110**	Ever cigarette smoke	Zanjan	Soltaniyeh	Female	0.89	0.00	4.41
**2111**	Ever cigarette smoke	Kermanshah	Sonqor	Female	2.09	0.00	7.33
**2112**	Ever cigarette smoke	Semnan	Sorkheh	Female	0.64	0.00	4.56
**2113**	Ever cigarette smoke	Gilan	Sume'eh Sara	Female	0.38	0.00	2.97
**2114**	Ever cigarette smoke	Khorasan_South	Tabas	Female	1.20	0.00	7.47
**2115**	Ever cigarette smoke	Azararbayjan_East	Tabriz	Female	1.81	0.00	4.27
**2116**	Ever cigarette smoke	Markazi	Tafresh	Female	1.62	0.00	4.42
**2117**	Ever cigarette smoke	Yazd	Taft	Female	2.41	0.00	10.12
**2118**	Ever cigarette smoke	Azarbayjan_West	Takab	Female	1.40	0.00	4.81
**2119**	Ever cigarette smoke	Qazvin	Takestan	Female	0.72	0.00	4.69
**2120**	Ever cigarette smoke	Khorasan_razavi	Takht-e-Jolgeh (Firuzeh)	Female	0.54	0.00	5.87
**2121**	Ever cigarette smoke	Alborz	Taleghan	Female	0.54	0.00	3.32
**2122**	Ever cigarette smoke	Boushehr	Tangestan	Female	3.96	0.00	14.34
**2123**	Ever cigarette smoke	Zanjan	Tarom	Female	0.51	0.00	3.08
**2124**	Ever cigarette smoke	Gilan	Tavalesh	Female	0.57	0.00	3.23
**2125**	Ever cigarette smoke	Khorasan_razavi	Taybad	Female	0.32	0.00	4.17
**2126**	Ever cigarette smoke	Tehran	Tehran	Female	1.68	0.00	5.27
**2127**	Ever cigarette smoke	Isfahan	Tiran and Karvan	Female	0.98	0.00	5.47
**2128**	Ever cigarette smoke	Mazandaran	Tonekabon	Female	0.60	0.00	5.37
**2129**	Ever cigarette smoke	Khorasan_razavi	Torbat-e-Heydariyeh	Female	1.00	0.00	4.76
**2130**	Ever cigarette smoke	Khorasan_razavi	Torbat-e-Jam	Female	0.91	0.00	4.78
**2131**	Ever cigarette smoke	Hamedan	Tuyserkan	Female	0.86	0.00	2.65
**2132**	Ever cigarette smoke	Tehran	Varamin	Female	1.21	0.00	5.07
**2133**	Ever cigarette smoke	Azararbayjan_East	Varzaqan	Female	2.51	0.00	5.56
**2134**	Ever cigarette smoke	Yazd	Yazd	Female	1.12	0.00	7.01
**2135**	Ever cigarette smoke	Sistan and Balouchestan	Zabol	Female	2.13	0.00	16.58
**2136**	Ever cigarette smoke	Sistan and Balouchestan	Zaboli (Mehrestan)	Female	3.12	0.00	22.69
**2137**	Ever cigarette smoke	Sistan and Balouchestan	Zahedan	Female	5.91	0.00	22.96
**2138**	Ever cigarette smoke	Zanjan	Zanjan	Female	1.34	0.00	4.42
**2139**	Ever cigarette smoke	Kerman	Zarand	Female	1.28	0.00	4.98
**2140**	Ever cigarette smoke	Markazi	Zarandiyeh	Female	1.42	0.00	4.04
**2141**	Ever cigarette smoke	Fars	Zarrindasht	Female	1.15	0.00	10.25
**2142**	Ever cigarette smoke	Khorasan_razavi	Zave	Female	0.57	0.00	5.41
**2143**	Ever cigarette smoke	Sistan and Balouchestan	Zehak	Female	3.73	0.00	25.48
**2144**	Ever cigarette smoke	Khorasan_South	Zir kuh	Female	2.40	0.00	11.93
**2145**	Ever cigarette smoke	Khuzestan	Abadan	Male	26.37	9.68	43.46
**2146**	Ever cigarette smoke	Fars	Abadeh	Male	34.66	22.52	48.29
**2147**	Ever cigarette smoke	Yazd	Abarkuh	Male	28.30	13.93	43.15
**2148**	Ever cigarette smoke	Mazandaran	Abbas abad	Male	43.21	29.22	55.47
**2149**	Ever cigarette smoke	Ilam	Abdanan	Male	14.75	3.87	25.64
**2150**	Ever cigarette smoke	Zanjan	Abhar	Male	25.25	12.60	37.59
**2151**	Ever cigarette smoke	Hormozgan	Abumusa	Male	21.16	2.46	40.75

**2152**	Ever cigarette smoke	Qazvin	Abyek	Male	29.61	13.35	45.26
**2153**	Ever cigarette smoke	Azararbayjan_East	Ahar	Male	27.50	14.38	40.70
**2154**	Ever cigarette smoke	Khuzestan	Ahvaz	Male	25.65	14.44	37.61
**2155**	Ever cigarette smoke	Azararbayjan_East	Ajabshir	Male	27.51	13.19	42.37
**2156**	Ever cigarette smoke	Qazvin	Alborz	Male	32.36	15.52	49.62
**2157**	Ever cigarette smoke	Golestan	Aliabad	Male	18.01	6.90	29.17
**2158**	Ever cigarette smoke	Lorestan	Aligudarz	Male	26.36	11.46	40.59
**2159**	Ever cigarette smoke	Gilan	Amlash	Male	28.70	12.13	45.56
**2160**	Ever cigarette smoke	Mazandaran	Amol	Male	29.32	15.57	42.98
**2161**	Ever cigarette smoke	Kerman	Anar	Male	28.53	10.83	47.33
**2162**	Ever cigarette smoke	Kerman	Anbarabad	Male	20.83	6.18	34.61
**2163**	Ever cigarette smoke	Khuzestan	Andika	Male	11.39	0.00	24.47
**2164**	Ever cigarette smoke	Khuzestan	Andimeshk	Male	17.43	4.83	29.27
**2165**	Ever cigarette smoke	Golestan	Aq Qala	Male	16.85	3.14	29.95
**2166**	Ever cigarette smoke	Khuzestan	Aqajari	Male	23.40	5.77	41.32
**2167**	Ever cigarette smoke	Semnan	Aradan	Male	25.55	9.00	41.27
**2168**	Ever cigarette smoke	Markazi	Arak	Male	37.03	21.08	54.54
**2169**	Ever cigarette smoke	Isfahan	Aran and Bidgol	Male	27.58	13.41	42.53
**2170**	Ever cigarette smoke	Ardebil	Ardabil	Male	28.49	16.08	41.54
**2171**	Ever cigarette smoke	Yazd	Ardakan	Male	21.74	8.92	33.24
**2172**	Ever cigarette smoke	Chaharmahal	Ardal	Male	29.30	11.83	47.01
**2173**	Ever cigarette smoke	Isfahan	Ardestan	Male	27.29	11.55	42.10
**2174**	Ever cigarette smoke	Fars	Arsanjan	Male	34.26	19.93	49.38
**2175**	Ever cigarette smoke	Kerman	Arzouyeh	Male	26.21	10.38	42.06
**2176**	Ever cigarette smoke	Hamedan	Asadabad	Male	30.11	13.41	46.26
**2177**	Ever cigarette smoke	Boushehr	Asaluyeh	Male	15.16	1.45	29.10
**2178**	Ever cigarette smoke	Markazi	Ashtiyan	Male	33.68	14.16	53.21
**2179**	Ever cigarette smoke	Gilan	Astaneh-ye-Ashrafiyeh	Male	29.54	14.72	45.70
**2180**	Ever cigarette smoke	Gilan	Astara	Male	26.35	10.16	42.37
**2181**	Ever cigarette smoke	Qazvin	Avaj	Male	32.74	15.16	50.86
**2182**	Ever cigarette smoke	Golestan	Azadshahr	Male	15.06	2.30	27.54
**2183**	Ever cigarette smoke	Azararbayjan_East	Azarshahr	Male	27.33	11.17	43.89
**2184**	Ever cigarette smoke	Lorestan	Azna	Male	30.08	13.71	47.03
**2185**	Ever cigarette smoke	Mazandaran	Babol	Male	25.56	11.81	38.68
**2186**	Ever cigarette smoke	Mazandaran	Babolsar	Male	27.64	10.61	45.16
**2187**	Ever cigarette smoke	Ilam	Badreh	Male	18.90	4.84	33.62
**2188**	Ever cigarette smoke	Yazd	Bafq	Male	18.42	8.52	29.27
**2189**	Ever cigarette smoke	Kerman	Baft	Male	26.62	13.02	40.39
**2190**	Ever cigarette smoke	Khuzestan	Baghemalek	Male	21.89	7.96	34.98
**2191**	Ever cigarette smoke	Yazd	Bahabad	Male	22.42	7.11	37.58
**2192**	Ever cigarette smoke	Hamedan	Bahar	Male	35.15	19.38	51.85
**2193**	Ever cigarette smoke	Tehran	Baharestan (Golestan)	Male	26.27	13.86	39.29
**2194**	Ever cigarette smoke	Kohkiluye and Bouyer Ahmad	Bahmani	Male	18.19	5.08	30.20
**2195**	Ever cigarette smoke	Khorasan_razavi	Bajestan	Male	18.65	4.12	33.22
**2196**	Ever cigarette smoke	Khorasan_razavi	Bakhraz	Male	20.21	4.41	36.07
**2197**	Ever cigarette smoke	Kerman	Bam	Male	24.62	10.30	39.40
**2198**	Ever cigarette smoke	Hormozgan	Bandar-e-Abbas	Male	20.53	9.02	32.27
**2199**	Ever cigarette smoke	Gilan	Bandar-e-Anzali	Male	27.99	13.11	43.06
**2200**	Ever cigarette smoke	Golestan	Bandar-e-Gaz	Male	15.02	0.00	30.17
**2201**	Ever cigarette smoke	Hormozgan	Bandar-e-Jask	Male	21.69	6.52	37.48
**2202**	Ever cigarette smoke	Hormozgan	Bandar-e-Lengeh	Male	19.74	5.70	33.97
**2203**	Ever cigarette smoke	Khuzestan	Bandar-e-Mahshahr	Male	25.65	11.34	40.73
**2204**	Ever cigarette smoke	Golestan	Bandar-e-Torkaman	Male	12.64	0.81	23.76
**2205**	Ever cigarette smoke	Kordestan	Baneh	Male	40.43	21.62	59.66
**2206**	Ever cigarette smoke	Khorasan_razavi	Bardeskan	Male	19.88	5.01	35.89
**2207**	Ever cigarette smoke	Kerman	Bardsir	Male	27.55	12.32	43.44
**2208**	Ever cigarette smoke	Hormozgan	Bashagerd	Male	20.13	5.30	35.00
**2209**	Ever cigarette smoke	Kohkiluye and Bouyer Ahmad	Basht	Male	15.62	4.73	26.69
**2210**	Ever cigarette smoke	Hormozgan	Bastak	Male	20.24	4.88	35.62
**2211**	Ever cigarette smoke	Khuzestan	Bavi	Male	24.68	11.74	37.61
**2212**	Ever cigarette smoke	Khuzestan	Behbahan	Male	24.09	9.34	39.33
**2213**	Ever cigarette smoke	Mazandaran	Behshahr	Male	19.80	3.45	35.13
**2214**	Ever cigarette smoke	Kordestan	Bijar	Male	29.40	15.43	42.23
**2215**	Ever cigarette smoke	Ardebil	Bilehsavar	Male	29.19	11.48	46.76
**2216**	Ever cigarette smoke	Khorasan_razavi	Binaloud	Male	18.64	1.73	35.37
**2217**	Ever cigarette smoke	Khorasan_South	Birjand	Male	13.09	2.22	23.41
**2218**	Ever cigarette smoke	Khorasan_North	Bojnurd	Male	12.59	3.18	21.80
**2219**	Ever cigarette smoke	Chaharmahal	Bon	Male	30.83	10.05	52.39
**2220**	Ever cigarette smoke	Azararbayjan_East	Bonab	Male	29.13	15.87	42.93
**2221**	Ever cigarette smoke	Isfahan	Borkhar	Male	27.77	12.14	43.78
**2222**	Ever cigarette smoke	Isfahan	Borkhar and Meymeh	Male	28.90	15.49	42.56
**2223**	Ever cigarette smoke	Chaharmahal	Borujen	Male	31.61	15.94	47.28
**2224**	Ever cigarette smoke	Lorestan	Borujerd	Male	29.79	14.06	45.54
**2225**	Ever cigarette smoke	Khorasan_South	Boshruyeh	Male	12.22	0.00	24.34
**2226**	Ever cigarette smoke	Azararbayjan_East	Bostanabad	Male	28.25	15.23	42.31
**2227**	Ever cigarette smoke	Fars	Bovanat	Male	23.70	9.59	36.56
**2228**	Ever cigarette smoke	Kohkiluye and Bouyer Ahmad	Boyer Ahmad	Male	23.45	10.65	36.02
**2229**	Ever cigarette smoke	Qazvin	Boyinzahra	Male	36.08	20.67	52.97
**2230**	Ever cigarette smoke	Isfahan	Buein va Miandasht	Male	27.56	11.23	44.01
**2231**	Ever cigarette smoke	Azarbayjan_West	Bukan	Male	37.29	21.18	54.12
**2232**	Ever cigarette smoke	Boushehr	Bushehr	Male	16.94	4.04	29.86
**2233**	Ever cigarette smoke	Isfahan	Chadegan	Male	28.22	12.26	44.03
**2234**	Ever cigarette smoke	Sistan and Balouchestan	Chahbahar	Male	19.25	5.33	33.33
**2235**	Ever cigarette smoke	Azarbayjan_West	Chaipareh	Male	43.83	25.86	62.48
**2236**	Ever cigarette smoke	Azarbayjan_West	Chaldoran	Male	45.81	26.26	66.19
**2237**	Ever cigarette smoke	Mazandaran	Chalus	Male	33.61	17.76	50.03
**2238**	Ever cigarette smoke	Azararbayjan_East	Charoimaq	Male	26.63	13.19	40.38
**2239**	Ever cigarette smoke	Khorasan_razavi	Chenaran	Male	20.04	4.98	36.43
**2240**	Ever cigarette smoke	Kohkiluye and Bouyer Ahmad	Cheram	Male	22.99	13.05	32.68
**2241**	Ever cigarette smoke	Kermanshah	Dalaho	Male	21.67	4.91	38.38
**2242**	Ever cigarette smoke	Lorestan	Dalfan	Male	27.27	12.59	42.80
**2243**	Ever cigarette smoke	Sistan and Balouchestan	Dalgan	Male	17.37	8.21	26.85
**2244**	Ever cigarette smoke	Tehran	Damavand	Male	23.49	10.24	36.81
**2245**	Ever cigarette smoke	Semnan	Damghan	Male	23.22	9.59	37.82
**2246**	Ever cigarette smoke	Fars	Darab	Male	29.16	15.15	43.96
**2247**	Ever cigarette smoke	Khorasan_South	Darmian	Male	12.29	0.00	24.38
**2248**	Ever cigarette smoke	Khorasan_razavi	Darrehgaz	Male	18.88	4.27	33.46
**2249**	Ever cigarette smoke	Ilam	Darrehshahr	Male	18.94	7.10	30.58

**2250**	Ever cigarette smoke	Khuzestan	Dasht-e-Azadegan	Male	23.42	6.09	40.19
**2251**	Ever cigarette smoke	Boushehr	Dashtestan	Male	17.49	5.85	28.75
**2252**	Ever cigarette smoke	Boushehr	Dashti	Male	14.58	2.68	25.64
**2253**	Ever cigarette smoke	Khorasan_razavi	Davarzan	Male	19.13	2.84	35.53
**2254**	Ever cigarette smoke	Boushehr	Dayyer	Male	14.70	6.22	23.63
**2255**	Ever cigarette smoke	Kordestan	Dehgolan	Male	31.53	15.71	48.03
**2256**	Ever cigarette smoke	Ilam	Dehloran	Male	18.91	4.50	32.64
**2257**	Ever cigarette smoke	Markazi	Delijan	Male	34.91	21.62	48.27
**2258**	Ever cigarette smoke	Kohkiluye and Bouyer Ahmad	Dena	Male	24.17	8.68	39.99
**2259**	Ever cigarette smoke	Boushehr	Deylam	Male	18.85	5.26	32.23
**2260**	Ever cigarette smoke	Khuzestan	Dezful	Male	20.18	8.06	31.71
**2261**	Ever cigarette smoke	Kordestan	Divandarreh	Male	41.84	26.03	60.60
**2262**	Ever cigarette smoke	Lorestan	Dorud	Male	29.19	11.78	46.53
**2263**	Ever cigarette smoke	Lorestan	Doureh	Male	26.19	9.27	42.12
**2264**	Ever cigarette smoke	Fars	Eqlid	Male	30.94	16.27	46.01
**2265**	Ever cigarette smoke	Khorasan_North	Esfarayen	Male	13.42	3.68	23.03
**2266**	Ever cigarette smoke	Alborz	Eshtehard	Male	30.28	14.78	45.95
**2267**	Ever cigarette smoke	Kermanshah	Eslamabad-e-Gharb	Male	19.30	5.42	32.83
**2268**	Ever cigarette smoke	Tehran	Eslamshahr	Male	26.31	14.10	38.86
**2269**	Ever cigarette smoke	Fars	Estahban	Male	28.14	12.18	44.35
**2270**	Ever cigarette smoke	Ilam	Eyvan	Male	17.50	3.55	31.28
**2271**	Ever cigarette smoke	Kerman	Fahraj	Male	27.85	13.05	43.82
**2272**	Ever cigarette smoke	Isfahan	Falavarjan	Male	30.28	15.91	45.42
**2273**	Ever cigarette smoke	Hamedan	Famenin	Male	32.08	14.56	49.84
**2274**	Ever cigarette smoke	Markazi	Farahan	Male	33.71	13.90	53.55
**2275**	Ever cigarette smoke	Fars	Farashband	Male	23.47	8.32	38.86
**2276**	Ever cigarette smoke	Alborz	Fardis	Male	29.36	12.37	46.54
**2277**	Ever cigarette smoke	Isfahan	Faridan	Male	29.26	14.46	44.66
**2278**	Ever cigarette smoke	Khorasan_razavi	Fariman	Male	11.36	0.00	23.39
**2279**	Ever cigarette smoke	Khorasan_North	Faroj	Male	11.21	2.51	19.78
**2280**	Ever cigarette smoke	Chaharmahal	Farsan	Male	30.85	12.94	48.61
**2281**	Ever cigarette smoke	Kerman	Faryab	Male	23.75	7.46	39.44
**2282**	Ever cigarette smoke	Fars	Fasa	Male	29.15	14.72	44.46
**2283**	Ever cigarette smoke	Khorasan_South	Ferdows	Male	10.20	0.11	20.02
**2284**	Ever cigarette smoke	Mazandaran	Fereydunkenar	Male	26.77	10.73	43.33
**2285**	Ever cigarette smoke	Isfahan	Fereydunshahr	Male	24.38	9.56	37.90
**2286**	Ever cigarette smoke	Fars	Firozabad	Male	26.93	16.33	37.84
**2287**	Ever cigarette smoke	Tehran	Firuzkuh	Male	20.23	7.49	32.03
**2288**	Ever cigarette smoke	Sistan and Balouchestan	Fonuj	Male	19.01	4.25	33.88
**2289**	Ever cigarette smoke	Gilan	Fuman	Male	25.99	12.63	39.15
**2290**	Ever cigarette smoke	Kohkiluye and Bouyer Ahmad	Gachsaran	Male	20.68	6.93	33.84
**2291**	Ever cigarette smoke	Golestan	Galikesh	Male	16.30	2.67	30.04
**2292**	Ever cigarette smoke	Mazandaran	Galugah	Male	21.11	1.08	41.13
**2293**	Ever cigarette smoke	Semnan	Garmsar	Male	24.82	10.29	38.58
**2294**	Ever cigarette smoke	Boushehr	Genaveh	Male	17.42	4.79	29.90
**2295**	Ever cigarette smoke	Fars	Gerash	Male	22.94	4.19	40.52
**2296**	Ever cigarette smoke	Khorasan_North	Germeh	Male	14.59	1.32	28.12
**2297**	Ever cigarette smoke	Ardebil	Germi	Male	32.25	17.28	47.98
**2298**	Ever cigarette smoke	Kerman	Ghaleye-Ganj	Male	22.26	7.25	36.73
**2299**	Ever cigarette smoke	Kermanshah	Gilan-e-Gharb	Male	21.37	9.82	33.21
**2300**	Ever cigarette smoke	Isfahan	Golpayegan	Male	30.45	16.55	45.16
**2301**	Ever cigarette smoke	Golestan	Gomishan	Male	15.01	0.00	30.59
**2302**	Ever cigarette smoke	Khorasan_razavi	Gonabad	Male	17.09	2.88	31.39
**2303**	Ever cigarette smoke	Golestan	Gonbad-e-Kavus	Male	17.48	4.95	30.00
**2304**	Ever cigarette smoke	Golestan	Gorgan	Male	17.05	5.17	28.57
**2305**	Ever cigarette smoke	Khuzestan	Guotvand	Male	21.69	6.03	36.55
**2306**	Ever cigarette smoke	Khuzestan	Haftgol	Male	39.02	23.96	53.78
**2307**	Ever cigarette smoke	Hormozgan	Hajiabad	Male	23.67	10.40	38.03
**2308**	Ever cigarette smoke	Hamedan	Hamadan	Male	33.07	18.57	48.00
**2309**	Ever cigarette smoke	Khuzestan	Hamidiyeh	Male	23.81	6.47	40.95
**2310**	Ever cigarette smoke	Sistan and Balouchestan	Hamoon	Male	16.66	0.05	33.71
**2311**	Ever cigarette smoke	Azararbayjan_East	Haris	Male	27.67	14.21	41.58
**2312**	Ever cigarette smoke	Kermanshah	Harsin	Male	16.95	1.81	30.45
**2313**	Ever cigarette smoke	Azararbayjan_East	Hashtrud	Male	27.91	13.91	42.20
**2314**	Ever cigarette smoke	Khuzestan	Hendijan	Male	23.71	6.73	40.72
**2315**	Ever cigarette smoke	Sistan and Balouchestan	Hirmand	Male	15.32	0.00	33.28
**2316**	Ever cigarette smoke	Khuzestan	Hoveizeh	Male	24.20	6.06	42.92
**2317**	Ever cigarette smoke	Zanjan	Ijerod	Male	28.90	16.75	41.45
**2318**	Ever cigarette smoke	Ilam	Ilam	Male	19.29	6.64	32.56
**2319**	Ever cigarette smoke	Sistan and Balouchestan	Iranshahr	Male	20.88	8.44	33.56
**2320**	Ever cigarette smoke	Isfahan	Isfahan	Male	27.81	17.28	38.18
**2321**	Ever cigarette smoke	Khuzestan	Izeh	Male	22.83	9.71	36.19
**2322**	Ever cigarette smoke	Fars	Jahrom	Male	26.66	12.46	41.25
**2323**	Ever cigarette smoke	Khorasan_North	Jajarm	Male	16.47	5.14	28.82
**2324**	Ever cigarette smoke	Boushehr	Jam	Male	15.36	3.00	27.83
**2325**	Ever cigarette smoke	Kermanshah	Javanrud	Male	24.59	6.70	42.24
**2326**	Ever cigarette smoke	Kerman	Jiroft	Male	26.09	12.96	39.53
**2327**	Ever cigarette smoke	Khorasan_razavi	Joghatai	Male	18.77	1.49	35.51
**2328**	Ever cigarette smoke	Azararbayjan_East	Jolfa	Male	29.13	14.95	43.57
**2329**	Ever cigarette smoke	Khorasan_razavi	Jowayin	Male	18.92	2.26	34.65
**2330**	Ever cigarette smoke	Mazandaran	Juybar	Male	26.58	9.69	43.67
**2331**	Ever cigarette smoke	Hamedan	Kabudarahang	Male	32.43	16.95	48.33
**2332**	Ever cigarette smoke	Kerman	Kahnuj	Male	20.62	6.34	33.48
**2333**	Ever cigarette smoke	Golestan	Kalaleh	Male	15.31	1.33	28.65
**2334**	Ever cigarette smoke	Khorasan_razavi	Kalat	Male	18.54	0.25	37.40
**2335**	Ever cigarette smoke	Azararbayjan_East	Kaleibar	Male	27.69	12.13	43.42
**2336**	Ever cigarette smoke	Kordestan	Kamyaran	Male	31.07	13.16	49.14
**2337**	Ever cigarette smoke	Boushehr	Kangan	Male	14.86	2.36	27.30
**2338**	Ever cigarette smoke	Kermanshah	Kangavar	Male	33.21	20.01	48.53
**2339**	Ever cigarette smoke	Alborz	Karaj	Male	29.34	18.28	40.61
**2340**	Ever cigarette smoke	Khuzestan	Karun	Male	24.31	5.19	44.23
**2341**	Ever cigarette smoke	Isfahan	Kashan	Male	28.02	14.30	42.10
**2342**	Ever cigarette smoke	Khorasan_razavi	Kashmar	Male	14.68	3.11	26.15
**2343**	Ever cigarette smoke	Fars	Kavar	Male	23.54	8.65	37.47
**2344**	Ever cigarette smoke	Fars	Kazerun	Male	24.20	11.07	37.02
**2345**	Ever cigarette smoke	Mazandaran	Kelardasht	Male	35.05	17.22	53.46
**2346**	Ever cigarette smoke	Kerman	Kerman	Male	24.06	12.27	35.78
**2347**	Ever cigarette smoke	Kermanshah	Kermanshah	Male	22.89	11.41	34.92

**2348**	Ever cigarette smoke	Khorasan_razavi	Khaf	Male	10.44	0.00	22.60
**2349**	Ever cigarette smoke	Khorasan_razavi	Khalilabad	Male	19.77	4.42	35.57
**2350**	Ever cigarette smoke	Ardebil	Khalkhal	Male	29.36	15.37	43.32
**2351**	Ever cigarette smoke	Hormozgan	Khamir	Male	28.01	15.28	40.74
**2352**	Ever cigarette smoke	Isfahan	Khansar	Male	27.31	11.83	42.74
**2353**	Ever cigarette smoke	Sistan and Balouchestan	Khash	Male	32.83	20.09	45.06
**2354**	Ever cigarette smoke	Yazd	Khatam	Male	27.51	13.07	42.18
**2355**	Ever cigarette smoke	Fars	Kherameh	Male	28.99	16.02	42.37
**2356**	Ever cigarette smoke	Azararbayjan_East	Khodaafarin	Male	28.34	12.48	44.36
**2357**	Ever cigarette smoke	Zanjan	Khodabandeh	Male	26.56	14.54	38.77
**2358**	Ever cigarette smoke	Markazi	Khomeyn	Male	38.30	22.19	55.63
**2359**	Ever cigarette smoke	Isfahan	Khomeynishahr	Male	29.77	15.36	44.50
**2360**	Ever cigarette smoke	Markazi	Khondab	Male	29.32	11.52	46.15
**2361**	Ever cigarette smoke	Fars	Khonj	Male	23.56	7.70	39.10
**2362**	Ever cigarette smoke	Isfahan	Khoor va Biabanak	Male	23.89	7.75	40.42
**2363**	Ever cigarette smoke	Lorestan	Khorramabad	Male	24.84	11.19	38.32
**2364**	Ever cigarette smoke	Fars	Khorrambid	Male	32.79	16.96	49.87
**2365**	Ever cigarette smoke	Zanjan	Khorramdarreh	Male	23.42	9.71	36.50
**2366**	Ever cigarette smoke	Khuzestan	Khorramshahr	Male	27.07	11.07	44.18
**2367**	Ever cigarette smoke	Khorasan_razavi	Khoshab	Male	18.06	2.26	33.68
**2368**	Ever cigarette smoke	Azarbayjan_West	Khoy	Male	42.93	27.46	58.89
**2369**	Ever cigarette smoke	Khorasan_South	Khusef	Male	14.42	1.24	27.39
**2370**	Ever cigarette smoke	Chaharmahal	Kiaar	Male	31.31	14.63	49.13
**2371**	Ever cigarette smoke	Kohkiluye and Bouyer Ahmad	Kohgiluyeh	Male	23.21	9.99	37.08
**2372**	Ever cigarette smoke	Markazi	Komeijan	Male	33.78	14.36	52.63
**2373**	Ever cigarette smoke	Sistan and Balouchestan	Konarak	Male	19.53	5.17	34.08
**2374**	Ever cigarette smoke	Golestan	Kordkuy	Male	17.40	4.09	30.88
**2375**	Ever cigarette smoke	Ardebil	Kowsar	Male	29.22	13.80	44.38
**2376**	Ever cigarette smoke	Kerman	Kuhbonan	Male	23.26	6.74	39.71
**2377**	Ever cigarette smoke	Lorestan	Kuhdasht	Male	25.86	10.39	41.46
**2378**	Ever cigarette smoke	Chaharmahal	Kuhrang	Male	23.94	8.25	38.58
**2379**	Ever cigarette smoke	Gilan	Lahijan	Male	31.14	18.44	44.27
**2380**	Ever cigarette smoke	Khuzestan	Lali	Male	22.06	5.06	38.42
**2381**	Ever cigarette smoke	Fars	Lamard	Male	13.94	1.42	26.88
**2382**	Ever cigarette smoke	Kohkiluye and Bouyer Ahmad	Landeh	Male	21.74	2.17	41.64
**2383**	Ever cigarette smoke	Gilan	Langrud	Male	28.50	13.84	43.10
**2384**	Ever cigarette smoke	Isfahan	Lanjan	Male	30.13	16.33	44.33
**2385**	Ever cigarette smoke	Fars	Lar (Larestan)	Male	25.38	11.39	39.62
**2386**	Ever cigarette smoke	Chaharmahal	Lordakan	Male	31.13	16.63	45.90
**2387**	Ever cigarette smoke	Azarbayjan_West	Mahabad	Male	37.84	20.43	54.43
**2388**	Ever cigarette smoke	Markazi	Mahalat	Male	35.91	16.14	55.32
**2389**	Ever cigarette smoke	Mazandaran	Mahmudabad	Male	26.24	10.89	40.69
**2390**	Ever cigarette smoke	Zanjan	Mahneshan	Male	28.90	14.88	43.53
**2391**	Ever cigarette smoke	Khorasan_razavi	Mahvelat	Male	17.81	2.05	33.93
**2392**	Ever cigarette smoke	Azarbayjan_West	Maku	Male	46.79	28.16	66.54
**2393**	Ever cigarette smoke	Tehran	Malard	Male	26.54	14.22	39.62
**2394**	Ever cigarette smoke	Hamedan	Malayer	Male	31.18	16.26	46.50
**2395**	Ever cigarette smoke	Azararbayjan_East	Malekan	Male	27.41	12.80	42.10
**2396**	Ever cigarette smoke	Ilam	Malekshahi	Male	19.25	4.44	35.00
**2397**	Ever cigarette smoke	Fars	Mamasany	Male	26.98	12.58	42.32
**2398**	Ever cigarette smoke	Khorasan_North	Maneh and Samalqan	Male	15.07	3.54	26.02
**2399**	Ever cigarette smoke	Kerman	Manujan	Male	22.65	5.88	39.16
**2400**	Ever cigarette smoke	Azararbayjan_East	Maragheh	Male	29.61	18.29	41.94
**2401**	Ever cigarette smoke	Azararbayjan_East	Marand	Male	27.63	13.96	40.87
**2402**	Ever cigarette smoke	Golestan	Maravehtapeh	Male	16.95	1.20	32.77
**2403**	Ever cigarette smoke	Kordestan	Marivan	Male	34.46	16.87	52.20
**2404**	Ever cigarette smoke	Fars	Marvdasht	Male	27.72	14.01	40.91
**2405**	Ever cigarette smoke	Gilan	Masal	Male	28.57	13.77	43.07
**2406**	Ever cigarette smoke	Khorasan_razavi	Mashhad	Male	17.82	7.35	28.45
**2407**	Ever cigarette smoke	Khuzestan	Masjed Soleyman	Male	22.63	8.47	36.60
**2408**	Ever cigarette smoke	Semnan	Mayamey	Male	19.61	3.56	35.32
**2409**	Ever cigarette smoke	Semnan	Mehdishahr	Male	24.03	8.96	39.35
**2410**	Ever cigarette smoke	Ilam	Mehran	Male	18.52	8.56	28.93
**2411**	Ever cigarette smoke	Yazd	Mehriz	Male	24.73	11.37	37.68
**2412**	Ever cigarette smoke	Ardebil	Meshkinshahr	Male	30.53	15.83	44.75
**2413**	Ever cigarette smoke	Yazd	Meybod	Male	31.35	16.80	47.20
**2414**	Ever cigarette smoke	Hormozgan	Minab	Male	18.06	4.73	31.09
**2415**	Ever cigarette smoke	Golestan	Minudasht	Male	13.97	1.73	25.85
**2416**	Ever cigarette smoke	Sistan and Balouchestan	Mirjaveh	Male	22.77	5.70	40.60
**2417**	Ever cigarette smoke	Azarbayjan_West	Miyandoab	Male	36.68	21.96	51.42
**2418**	Ever cigarette smoke	Mazandaran	Miyandorud	Male	24.02	2.74	44.49
**2419**	Ever cigarette smoke	Azararbayjan_East	Miyaneh	Male	25.33	13.15	37.41
**2420**	Ever cigarette smoke	Isfahan	Mobarakeh	Male	30.23	16.09	45.14
**2421**	Ever cigarette smoke	Fars	Mohr	Male	18.50	7.75	29.36
**2422**	Ever cigarette smoke	Hamedan	Nahavand	Male	30.75	14.55	47.23
**2423**	Ever cigarette smoke	Isfahan	Najafabad	Male	29.18	15.55	43.41
**2424**	Ever cigarette smoke	Ardebil	Namin	Male	29.94	13.32	46.65
**2425**	Ever cigarette smoke	Azarbayjan_West	Naqadeh	Male	38.75	21.57	55.83
**2426**	Ever cigarette smoke	Kerman	Narmashir	Male	24.87	7.95	40.79
**2427**	Ever cigarette smoke	Isfahan	Natanz	Male	27.26	11.68	42.93
**2428**	Ever cigarette smoke	Isfahan	Nayin	Male	28.59	15.38	43.06
**2429**	Ever cigarette smoke	Alborz	Nazarabad	Male	33.31	18.04	49.46
**2430**	Ever cigarette smoke	Ardebil	Neer	Male	29.96	19.18	40.84
**2431**	Ever cigarette smoke	Khorasan_South	Nehbandan	Male	9.85	0.03	19.77
**2432**	Ever cigarette smoke	Mazandaran	Neka	Male	21.54	5.75	36.67
**2433**	Ever cigarette smoke	Fars	Neyriz	Male	28.31	14.99	42.03
**2434**	Ever cigarette smoke	Khorasan_razavi	Neyshabur	Male	14.61	3.34	25.33
**2435**	Ever cigarette smoke	Sistan and Balouchestan	Nikshahr	Male	16.05	4.99	27.00
**2436**	Ever cigarette smoke	Sistan and Balouchestan	Nimruz	Male	16.61	0.82	32.08
**2437**	Ever cigarette smoke	Mazandaran	Noshahr	Male	31.98	13.13	51.40
**2438**	Ever cigarette smoke	Mazandaran	Nur	Male	28.18	13.20	42.80
**2439**	Ever cigarette smoke	Khuzestan	Omidiyeh	Male	24.68	9.96	40.48
**2440**	Ever cigarette smoke	Azarbayjan_West	Orumiyeh	Male	39.16	25.04	53.96
**2441**	Ever cigarette smoke	Azarbayjan_West	Oshnaviyeh	Male	40.33	21.11	60.44
**2442**	Ever cigarette smoke	Azararbayjan_East	Osku	Male	26.55	13.14	39.49
**2443**	Ever cigarette smoke	Tehran	Pakdasht	Male	23.28	11.31	34.95
**2444**	Ever cigarette smoke	Tehran	Pardis	Male	23.80	9.12	38.54
**2445**	Ever cigarette smoke	Ardebil	Parsabad	Male	30.72	14.06	47.99

**2446**	Ever cigarette smoke	Hormozgan	Parsian (Gavbandi)	Male	18.09	6.37	29.87
**2447**	Ever cigarette smoke	Fars	Pasargad	Male	42.95	26.99	61.68
**2448**	Ever cigarette smoke	Kermanshah	Paveh	Male	25.82	8.44	42.70
**2449**	Ever cigarette smoke	Azarbayjan_West	Piranshahr	Male	39.28	21.19	57.66
**2450**	Ever cigarette smoke	Tehran	Pishva	Male	21.45	8.58	33.87
**2451**	Ever cigarette smoke	Azarbayjan_West	Poldasht	Male	44.79	29.22	60.43
**2452**	Ever cigarette smoke	Lorestan	Poldokhtar	Male	24.16	7.85	39.97
**2453**	Ever cigarette smoke	Mazandaran	Qaemshahr	Male	27.23	11.37	43.05
**2454**	Ever cigarette smoke	Tehran	Qarchak	Male	24.21	7.69	40.57
**2455**	Ever cigarette smoke	Sistan and Balouchestan	Qasr qand	Male	19.19	3.41	34.56
**2456**	Ever cigarette smoke	Kermanshah	Qasr-e-Shirin	Male	20.55	0.08	40.89
**2457**	Ever cigarette smoke	Khorasan_South	Qayenat	Male	14.48	2.63	26.48
**2458**	Ever cigarette smoke	Qazvin	Qazvin	Male	35.92	21.34	51.83
**2459**	Ever cigarette smoke	Hormozgan	Qeshm	Male	21.64	6.74	36.40
**2460**	Ever cigarette smoke	Fars	Qirokarzin	Male	26.49	16.28	36.81
**2461**	Ever cigarette smoke	Qom	Qom	Male	26.05	10.91	41.19
**2462**	Ever cigarette smoke	Kordestan	Qorveh	Male	28.99	12.70	43.87
**2463**	Ever cigarette smoke	Khorasan_razavi	Quchan	Male	20.15	4.69	35.95
**2464**	Ever cigarette smoke	Kerman	Rabar	Male	30.96	15.30	48.90
**2465**	Ever cigarette smoke	Kerman	Rafsanjan	Male	26.79	13.03	40.80
**2466**	Ever cigarette smoke	Khuzestan	Ramhormoz	Male	23.84	10.04	37.72
**2467**	Ever cigarette smoke	Mazandaran	Ramsar	Male	33.84	16.76	52.04
**2468**	Ever cigarette smoke	Khuzestan	Ramshir	Male	25.24	9.48	41.75
**2469**	Ever cigarette smoke	Golestan	Ramyan	Male	16.64	3.37	30.07
**2470**	Ever cigarette smoke	Gilan	Rasht	Male	27.00	16.24	37.79
**2471**	Ever cigarette smoke	Khorasan_razavi	Rashtkhar	Male	15.91	1.86	29.08
**2472**	Ever cigarette smoke	Kermanshah	Ravansar	Male	24.29	7.72	40.65
**2473**	Ever cigarette smoke	Kerman	Ravar	Male	26.38	13.75	39.26
**2474**	Ever cigarette smoke	Khorasan_North	Raz va Jergolan	Male	14.16	0.00	29.29
**2475**	Ever cigarette smoke	Hamedan	Razan	Male	34.14	17.70	51.61
**2476**	Ever cigarette smoke	Tehran	Rey	Male	25.08	12.56	37.88
**2477**	Ever cigarette smoke	Kerman	Reygan	Male	24.76	9.21	39.85
**2478**	Ever cigarette smoke	Gilan	Rezvanshahr	Male	29.73	16.20	43.93
**2479**	Ever cigarette smoke	Tehran	Robatkarim	Male	23.11	10.52	35.60
**2480**	Ever cigarette smoke	Fars	Rostam	Male	24.36	7.96	39.33
**2481**	Ever cigarette smoke	Kerman	Roudbar-e-Jonub	Male	22.23	8.08	36.47
**2482**	Ever cigarette smoke	Hormozgan	Rudan	Male	12.40	1.03	24.01
**2483**	Ever cigarette smoke	Gilan	Rudbar	Male	28.35	14.56	41.61
**2484**	Ever cigarette smoke	Gilan	Rudsar	Male	28.84	16.31	42.10
**2485**	Ever cigarette smoke	Lorestan	Rumshekan	Male	24.02	4.47	42.60
**2486**	Ever cigarette smoke	Khorasan_razavi	Sabzevar	Male	19.66	7.41	32.74
**2487**	Ever cigarette smoke	Yazd	Sadugh	Male	42.56	26.11	59.12
**2488**	Ever cigarette smoke	Kermanshah	Sahneh	Male	20.29	8.78	31.41
**2489**	Ever cigarette smoke	Kermanshah	Salas-e-Babajani	Male	21.33	7.01	35.33
**2490**	Ever cigarette smoke	Azarbayjan_West	Salmas	Male	43.51	25.96	63.12
**2491**	Ever cigarette smoke	Chaharmahal	Saman	Male	31.33	12.22	51.21
**2492**	Ever cigarette smoke	Kordestan	Sanandaj	Male	31.87	16.47	47.48
**2493**	Ever cigarette smoke	Kordestan	Saqqez	Male	37.83	21.47	54.64
**2494**	Ever cigarette smoke	Kermanshah	Sar-e-Pol-e-Zohab	Male	17.77	3.27	31.69
**2495**	Ever cigarette smoke	Azararbayjan_East	Sarab	Male	26.20	13.37	38.36
**2496**	Ever cigarette smoke	Khorasan_razavi	Sarakhs	Male	19.10	1.92	36.75
**2497**	Ever cigarette smoke	Sistan and Balouchestan	Saravan	Male	27.98	13.83	42.39
**2498**	Ever cigarette smoke	Khorasan_South	Sarayan	Male	13.55	0.96	25.83
**2499**	Ever cigarette smoke	Sistan and Balouchestan	Sarbaz	Male	19.01	5.80	31.80
**2500**	Ever cigarette smoke	Khorasan_South	Sarbisheh	Male	18.34	6.77	31.00
**2501**	Ever cigarette smoke	Azarbayjan_West	Sardasht	Male	39.51	21.99	57.70
**2502**	Ever cigarette smoke	Ardebil	Sarein	Male	29.68	13.78	45.85
**2503**	Ever cigarette smoke	Mazandaran	Sari	Male	25.91	11.93	40.09
**2504**	Ever cigarette smoke	Kordestan	Sarvabad	Male	31.86	12.22	52.13
**2505**	Ever cigarette smoke	Fars	Sarvestan	Male	27.11	10.54	43.38
**2506**	Ever cigarette smoke	Mazandaran	Savadkuh	Male	28.84	13.02	45.96
**2507**	Ever cigarette smoke	Mazandaran	Savadkuh_North	Male	26.69	8.68	44.47
**2508**	Ever cigarette smoke	Markazi	Saveh	Male	32.41	15.52	48.21
**2509**	Ever cigarette smoke	Alborz	Savojbolagh	Male	31.87	16.84	47.40
**2510**	Ever cigarette smoke	Lorestan	Selseleh	Male	27.65	10.91	44.64
**2511**	Ever cigarette smoke	Isfahan	Semirom	Male	29.94	18.23	42.65
**2512**	Ever cigarette smoke	Isfahan	Semirom-e-Sofla	Male	28.91	11.86	45.59
**2513**	Ever cigarette smoke	Semnan	Semnan	Male	23.12	8.27	37.66
**2514**	Ever cigarette smoke	Fars	Sepidan	Male	30.10	15.42	45.68
**2515**	Ever cigarette smoke	Azararbayjan_East	Shabestar	Male	21.40	8.84	32.94
**2516**	Ever cigarette smoke	Khuzestan	Shadegan	Male	21.71	7.46	35.08
**2517**	Ever cigarette smoke	Gilan	Shaft	Male	28.26	16.25	39.61
**2518**	Ever cigarette smoke	Azarbayjan_West	Shahindezh	Male	32.69	17.65	47.13
**2519**	Ever cigarette smoke	Tehran	Shahr-e Qods	Male	24.33	11.18	37.24
**2520**	Ever cigarette smoke	Kerman	Shahr-e-Babak	Male	27.20	12.14	42.83
**2521**	Ever cigarette smoke	Chaharmahal	Shahr-e-Kord	Male	33.35	17.64	49.56
**2522**	Ever cigarette smoke	Isfahan	Shahreza	Male	29.70	14.68	45.03
**2523**	Ever cigarette smoke	Tehran	Shahriyar	Male	26.67	14.93	38.97
**2524**	Ever cigarette smoke	Semnan	Shahrud	Male	21.62	8.86	35.13
**2525**	Ever cigarette smoke	Markazi	Shazand	Male	36.99	20.96	54.80
**2526**	Ever cigarette smoke	Tehran	Shemiranat	Male	23.34	10.25	36.14
**2527**	Ever cigarette smoke	Fars	Shiraz	Male	26.76	15.11	38.20
**2528**	Ever cigarette smoke	Khorasan_North	Shirvan	Male	13.37	2.06	24.45
**2529**	Ever cigarette smoke	Ilam	Shirvan and Chard-e-Aval	Male	18.88	6.02	31.38
**2530**	Ever cigarette smoke	Azarbayjan_West	Showt	Male	58.55	39.50	77.32
**2531**	Ever cigarette smoke	Khuzestan	Shush	Male	22.49	9.25	35.45
**2532**	Ever cigarette smoke	Khuzestan	Shushtar	Male	23.69	10.89	36.60
**2533**	Ever cigarette smoke	Gilan	Siahkal	Male	29.24	14.99	44.01
**2534**	Ever cigarette smoke	Sistan and Balouchestan	Sib o Soran	Male	18.64	5.93	31.26
**2535**	Ever cigarette smoke	Mazandaran	Simorgh	Male	26.62	8.24	45.72
**2536**	Ever cigarette smoke	Hormozgan	Sirik	Male	21.80	11.51	31.85
**2537**	Ever cigarette smoke	Kerman	Sirjan	Male	27.05	13.63	41.05
**2538**	Ever cigarette smoke	Ilam	Sirvan	Male	19.08	3.56	34.03
**2539**	Ever cigarette smoke	Zanjan	Soltaniyeh	Male	27.77	13.21	43.12
**2540**	Ever cigarette smoke	Kermanshah	Sonqor	Male	26.85	11.95	41.95
**2541**	Ever cigarette smoke	Semnan	Sorkheh	Male	24.87	8.81	41.14
**2542**	Ever cigarette smoke	Gilan	Sume'eh Sara	Male	30.17	18.09	42.64
**2543**	Ever cigarette smoke	Khorasan_South	Tabas	Male	12.44	2.76	21.46

**2544**	Ever cigarette smoke	Azararbayjan_East	Tabriz	Male	26.86	16.51	37.42
**2545**	Ever cigarette smoke	Markazi	Tafresh	Male	29.53	11.32	46.41
**2546**	Ever cigarette smoke	Yazd	Taft	Male	28.47	15.58	41.97
**2547**	Ever cigarette smoke	Azarbayjan_West	Takab	Male	36.21	20.51	52.78
**2548**	Ever cigarette smoke	Qazvin	Takestan	Male	34.34	16.76	53.16
**2549**	Ever cigarette smoke	Khorasan_razavi	Takht-e-Jolgeh (Firuzeh)	Male	19.37	7.08	31.35
**2550**	Ever cigarette smoke	Alborz	Taleghan	Male	34.18	19.90	50.39
**2551**	Ever cigarette smoke	Boushehr	Tangestan	Male	16.69	1.10	31.30
**2552**	Ever cigarette smoke	Zanjan	Tarom	Male	28.76	15.13	42.64
**2553**	Ever cigarette smoke	Gilan	Tavalesh	Male	25.89	12.59	38.42
**2554**	Ever cigarette smoke	Khorasan_razavi	Taybad	Male	20.20	4.05	36.79
**2555**	Ever cigarette smoke	Tehran	Tehran	Male	23.47	14.26	32.49
**2556**	Ever cigarette smoke	Isfahan	Tiran and Karvan	Male	28.66	13.31	44.25
**2557**	Ever cigarette smoke	Mazandaran	Tonekabon	Male	34.45	18.94	51.51
**2558**	Ever cigarette smoke	Khorasan_razavi	Torbat-e-Heydariyeh	Male	19.29	5.92	33.63
**2559**	Ever cigarette smoke	Khorasan_razavi	Torbat-e-Jam	Male	17.83	5.57	30.18
**2560**	Ever cigarette smoke	Hamedan	Tuyserkan	Male	28.49	13.59	42.41
**2561**	Ever cigarette smoke	Tehran	Varamin	Male	24.85	13.51	36.86
**2562**	Ever cigarette smoke	Azararbayjan_East	Varzaqan	Male	27.85	13.56	41.90
**2563**	Ever cigarette smoke	Yazd	Yazd	Male	23.23	11.96	34.29
**2564**	Ever cigarette smoke	Sistan and Balouchestan	Zabol	Male	10.89	0.00	23.61
**2565**	Ever cigarette smoke	Sistan and Balouchestan	Zaboli (Mehrestan)	Male	19.32	10.14	28.69
**2566**	Ever cigarette smoke	Sistan and Balouchestan	Zahedan	Male	21.56	10.79	33.26
**2567**	Ever cigarette smoke	Zanjan	Zanjan	Male	29.27	17.89	40.74
**2568**	Ever cigarette smoke	Kerman	Zarand	Male	22.28	7.40	36.39
**2569**	Ever cigarette smoke	Markazi	Zarandiyeh	Male	37.08	20.40	55.44
**2570**	Ever cigarette smoke	Fars	Zarrindasht	Male	27.46	11.30	44.28
**2571**	Ever cigarette smoke	Khorasan_razavi	Zave	Male	27.82	14.42	42.99
**2572**	Ever cigarette smoke	Sistan and Balouchestan	Zehak	Male	15.38	0.00	33.24
**2573**	Ever cigarette smoke	Khorasan_South	Zir kuh	Male	13.33	0.00	27.49
**2574**	Ever tobacco smoke	Khuzestan	Abadan	Female	3.69	0.00	10.19
**2575**	Ever tobacco smoke	Fars	Abadeh	Female	10.72	0.95	20.36
**2576**	Ever tobacco smoke	Yazd	Abarkuh	Female	9.05	0.00	20.13
**2577**	Ever tobacco smoke	Mazandaran	Abbas abad	Female	3.30	0.00	13.31
**2578**	Ever tobacco smoke	Ilam	Abdanan	Female	2.85	0.00	9.00
**2579**	Ever tobacco smoke	Zanjan	Abhar	Female	2.55	0.00	8.55
**2580**	Ever tobacco smoke	Hormozgan	Abumusa	Female	16.51	0.12	33.24
**2581**	Ever tobacco smoke	Qazvin	Abyek	Female	7.05	0.00	25.85
**2582**	Ever tobacco smoke	Azararbayjan_East	Ahar	Female	4.01	0.00	8.33
**2583**	Ever tobacco smoke	Khuzestan	Ahvaz	Female	2.38	0.00	6.89
**2584**	Ever tobacco smoke	Azararbayjan_East	Ajabshir	Female	1.56	0.00	7.53
**2585**	Ever tobacco smoke	Qazvin	Alborz	Female	10.35	0.00	30.74
**2586**	Ever tobacco smoke	Golestan	Aliabad	Female	6.32	0.00	16.80
**2587**	Ever tobacco smoke	Lorestan	Aligudarz	Female	4.66	0.00	14.77
**2588**	Ever tobacco smoke	Gilan	Amlash	Female	1.56	0.00	9.71
**2589**	Ever tobacco smoke	Mazandaran	Amol	Female	3.26	0.00	11.00
**2590**	Ever tobacco smoke	Kerman	Anar	Female	3.78	0.00	14.59
**2591**	Ever tobacco smoke	Kerman	Anbarabad	Female	5.98	0.00	14.89
**2592**	Ever tobacco smoke	Khuzestan	Andika	Female	2.70	0.00	9.88
**2593**	Ever tobacco smoke	Khuzestan	Andimeshk	Female	1.40	0.00	5.99
**2594**	Ever tobacco smoke	Golestan	Aq Qala	Female	2.94	0.00	10.44
**2595**	Ever tobacco smoke	Khuzestan	Aqajari	Female	3.88	0.00	11.79
**2596**	Ever tobacco smoke	Semnan	Aradan	Female	2.91	0.00	11.04
**2597**	Ever tobacco smoke	Markazi	Arak	Female	6.31	0.00	14.44
**2598**	Ever tobacco smoke	Isfahan	Aran and Bidgol	Female	2.16	0.00	8.07
**2599**	Ever tobacco smoke	Ardebil	Ardabil	Female	1.76	0.00	6.53
**2600**	Ever tobacco smoke	Yazd	Ardakan	Female	2.97	0.00	10.56
**2601**	Ever tobacco smoke	Chaharmahal	Ardal	Female	2.11	0.00	6.44
**2602**	Ever tobacco smoke	Isfahan	Ardestan	Female	4.51	0.00	12.78
**2603**	Ever tobacco smoke	Fars	Arsanjan	Female	11.15	0.87	21.55
**2604**	Ever tobacco smoke	Kerman	Arzouyeh	Female	5.20	0.00	15.22
**2605**	Ever tobacco smoke	Hamedan	Asadabad	Female	2.50	0.00	7.67
**2606**	Ever tobacco smoke	Boushehr	Asaluyeh	Female	22.80	8.14	38.12
**2607**	Ever tobacco smoke	Markazi	Ashtiyan	Female	5.80	0.00	16.86
**2608**	Ever tobacco smoke	Gilan	Astaneh-ye-Ashrafiyeh	Female	1.92	0.00	8.03
**2609**	Ever tobacco smoke	Gilan	Astara	Female	1.71	0.00	10.95
**2610**	Ever tobacco smoke	Qazvin	Avaj	Female	6.94	0.00	25.55
**2611**	Ever tobacco smoke	Golestan	Azadshahr	Female	6.24	0.00	16.99
**2612**	Ever tobacco smoke	Azararbayjan_East	Azarshahr	Female	0.69	0.00	4.88
**2613**	Ever tobacco smoke	Lorestan	Azna	Female	8.94	0.00	22.06
**2614**	Ever tobacco smoke	Mazandaran	Babol	Female	3.89	0.00	12.15
**2615**	Ever tobacco smoke	Mazandaran	Babolsar	Female	4.11	0.00	15.25
**2616**	Ever tobacco smoke	Ilam	Badreh	Female	2.88	0.00	10.58
**2617**	Ever tobacco smoke	Yazd	Bafq	Female	3.31	0.00	10.97
**2618**	Ever tobacco smoke	Kerman	Baft	Female	2.78	0.00	9.28
**2619**	Ever tobacco smoke	Khuzestan	Baghemalek	Female	1.66	0.00	6.47
**2620**	Ever tobacco smoke	Yazd	Bahabad	Female	5.31	0.00	17.04
**2621**	Ever tobacco smoke	Hamedan	Bahar	Female	5.24	0.00	11.43
**2622**	Ever tobacco smoke	Tehran	Baharestan (Golestan)	Female	3.19	0.00	10.76
**2623**	Ever tobacco smoke	Kohkiluye and Bouyer Ahmad	Bahmani	Female	18.43	1.56	35.92
**2624**	Ever tobacco smoke	Khorasan_razavi	Bajestan	Female	11.32	1.50	21.37
**2625**	Ever tobacco smoke	Khorasan_razavi	Bakhraz	Female	16.57	5.16	28.66
**2626**	Ever tobacco smoke	Kerman	Bam	Female	6.22	0.00	15.38
**2627**	Ever tobacco smoke	Hormozgan	Bandar-e-Abbas	Female	16.61	4.96	28.54
**2628**	Ever tobacco smoke	Gilan	Bandar-e-Anzali	Female	2.52	0.00	9.97
**2629**	Ever tobacco smoke	Golestan	Bandar-e-Gaz	Female	5.88	0.00	18.71
**2630**	Ever tobacco smoke	Hormozgan	Bandar-e-Jask	Female	17.65	3.23	32.74
**2631**	Ever tobacco smoke	Hormozgan	Bandar-e-Lengeh	Female	18.82	5.82	32.56
**2632**	Ever tobacco smoke	Khuzestan	Bandar-e-Mahshahr	Female	3.69	0.00	9.91
**2633**	Ever tobacco smoke	Golestan	Bandar-e-Torkaman	Female	5.57	0.00	17.05
**2634**	Ever tobacco smoke	Kordestan	Baneh	Female	8.61	0.00	18.89
**2635**	Ever tobacco smoke	Khorasan_razavi	Bardeskan	Female	9.08	0.65	17.30
**2636**	Ever tobacco smoke	Kerman	Bardsir	Female	5.69	0.00	12.08
**2637**	Ever tobacco smoke	Hormozgan	Bashagerd	Female	18.44	5.29	32.57
**2638**	Ever tobacco smoke	Kohkiluye and Bouyer Ahmad	Basht	Female	23.20	10.41	35.83
**2639**	Ever tobacco smoke	Hormozgan	Bastak	Female	19.23	5.59	33.71
**2640**	Ever tobacco smoke	Khuzestan	Bavi	Female	2.68	0.00	10.29
**2641**	Ever tobacco smoke	Khuzestan	Behbahan	Female	4.13	0.00	9.73

**2642**	Ever tobacco smoke	Mazandaran	Behshahr	Female	6.44	0.00	17.99
**2643**	Ever tobacco smoke	Kordestan	Bijar	Female	5.56	0.00	16.13
**2644**	Ever tobacco smoke	Ardebil	Bilehsavar	Female	3.08	0.00	11.59
**2645**	Ever tobacco smoke	Khorasan_razavi	Binaloud	Female	19.44	11.53	26.98
**2646**	Ever tobacco smoke	Khorasan_South	Birjand	Female	5.20	0.00	11.20
**2647**	Ever tobacco smoke	Khorasan_North	Bojnurd	Female	5.65	0.00	13.51
**2648**	Ever tobacco smoke	Chaharmahal	Bon	Female	1.73	0.00	7.91
**2649**	Ever tobacco smoke	Azararbayjan_East	Bonab	Female	0.91	0.00	4.83
**2650**	Ever tobacco smoke	Isfahan	Borkhar	Female	5.31	0.00	12.65
**2651**	Ever tobacco smoke	Isfahan	Borkhar and Meymeh	Female	9.67	2.80	17.03
**2652**	Ever tobacco smoke	Chaharmahal	Borujen	Female	1.98	0.00	5.88
**2653**	Ever tobacco smoke	Lorestan	Borujerd	Female	12.54	0.00	27.60
**2654**	Ever tobacco smoke	Khorasan_South	Boshruyeh	Female	4.50	0.00	12.85
**2655**	Ever tobacco smoke	Azararbayjan_East	Bostanabad	Female	1.21	0.00	4.73
**2656**	Ever tobacco smoke	Fars	Bovanat	Female	10.86	0.83	21.01
**2657**	Ever tobacco smoke	Kohkiluye and Bouyer Ahmad	Boyer Ahmad	Female	21.65	5.37	38.99
**2658**	Ever tobacco smoke	Qazvin	Boyinzahra	Female	4.06	0.00	16.99
**2659**	Ever tobacco smoke	Isfahan	Buein va Miandasht	Female	4.81	0.00	13.77
**2660**	Ever tobacco smoke	Azarbayjan_West	Bukan	Female	4.14	0.00	10.39
**2661**	Ever tobacco smoke	Boushehr	Bushehr	Female	18.43	4.58	33.15
**2662**	Ever tobacco smoke	Isfahan	Chadegan	Female	4.63	0.00	12.73
**2663**	Ever tobacco smoke	Sistan and Balouchestan	Chahbahar	Female	25.99	5.54	47.21
**2664**	Ever tobacco smoke	Azarbayjan_West	Chaipareh	Female	4.13	0.00	13.08
**2665**	Ever tobacco smoke	Azarbayjan_West	Chaldoran	Female	3.99	0.00	14.12
**2666**	Ever tobacco smoke	Mazandaran	Chalus	Female	4.08	0.00	13.15
**2667**	Ever tobacco smoke	Azararbayjan_East	Charoimaq	Female	2.63	0.00	7.82
**2668**	Ever tobacco smoke	Khorasan_razavi	Chenaran	Female	15.03	5.27	25.34
**2669**	Ever tobacco smoke	Kohkiluye and Bouyer Ahmad	Cheram	Female	15.86	1.23	29.99
**2670**	Ever tobacco smoke	Kermanshah	Dalaho	Female	3.34	0.00	10.63
**2671**	Ever tobacco smoke	Lorestan	Dalfan	Female	3.76	0.00	13.60
**2672**	Ever tobacco smoke	Sistan and Balouchestan	Dalgan	Female	19.62	1.61	38.41
**2673**	Ever tobacco smoke	Tehran	Damavand	Female	1.43	0.00	8.36
**2674**	Ever tobacco smoke	Semnan	Damghan	Female	4.36	0.00	10.58
**2675**	Ever tobacco smoke	Fars	Darab	Female	13.09	3.64	22.84
**2676**	Ever tobacco smoke	Khorasan_South	Darmian	Female	5.86	0.00	13.64
**2677**	Ever tobacco smoke	Khorasan_razavi	Darrehgaz	Female	6.51	0.00	14.40
**2678**	Ever tobacco smoke	Ilam	Darrehshahr	Female	1.55	0.00	7.30
**2679**	Ever tobacco smoke	Khuzestan	Dasht-e-Azadegan	Female	1.23	0.00	6.51
**2680**	Ever tobacco smoke	Boushehr	Dashtestan	Female	20.67	8.57	33.67
**2681**	Ever tobacco smoke	Boushehr	Dashti	Female	20.77	6.83	34.95
**2682**	Ever tobacco smoke	Khorasan_razavi	Davarzan	Female	9.48	0.00	20.20
**2683**	Ever tobacco smoke	Boushehr	Dayyer	Female	22.16	6.64	38.01
**2684**	Ever tobacco smoke	Kordestan	Dehgolan	Female	5.12	0.00	16.31
**2685**	Ever tobacco smoke	Ilam	Dehloran	Female	5.56	0.00	11.74
**2686**	Ever tobacco smoke	Markazi	Delijan	Female	6.63	0.00	17.44
**2687**	Ever tobacco smoke	Kohkiluye and Bouyer Ahmad	Dena	Female	20.54	1.19	40.29
**2688**	Ever tobacco smoke	Boushehr	Deylam	Female	15.24	1.45	29.06
**2689**	Ever tobacco smoke	Khuzestan	Dezful	Female	2.24	0.00	6.95
**2690**	Ever tobacco smoke	Kordestan	Divandarreh	Female	5.87	0.00	16.63
**2691**	Ever tobacco smoke	Lorestan	Dorud	Female	9.72	0.00	22.53
**2692**	Ever tobacco smoke	Lorestan	Doureh	Female	6.87	0.00	21.52
**2693**	Ever tobacco smoke	Fars	Eqlid	Female	9.79	1.19	18.17
**2694**	Ever tobacco smoke	Khorasan_North	Esfarayen	Female	6.81	0.00	16.26
**2695**	Ever tobacco smoke	Alborz	Eshtehard	Female	2.44	0.00	9.61
**2696**	Ever tobacco smoke	Kermanshah	Eslamabad-e-Gharb	Female	3.89	0.00	10.26
**2697**	Ever tobacco smoke	Tehran	Eslamshahr	Female	3.55	0.00	12.06
**2698**	Ever tobacco smoke	Fars	Estahban	Female	12.28	1.61	22.79
**2699**	Ever tobacco smoke	Ilam	Eyvan	Female	2.84	0.00	10.03
**2700**	Ever tobacco smoke	Kerman	Fahraj	Female	5.37	0.00	15.75
**2701**	Ever tobacco smoke	Isfahan	Falavarjan	Female	5.11	0.00	11.94
**2702**	Ever tobacco smoke	Hamedan	Famenin	Female	14.34	6.94	21.76
**2703**	Ever tobacco smoke	Markazi	Farahan	Female	5.79	0.00	16.73
**2704**	Ever tobacco smoke	Fars	Farashband	Female	13.70	4.84	22.11
**2705**	Ever tobacco smoke	Alborz	Fardis	Female	2.63	0.00	10.88
**2706**	Ever tobacco smoke	Isfahan	Faridan	Female	2.59	0.00	8.38
**2707**	Ever tobacco smoke	Khorasan_razavi	Fariman	Female	12.23	3.52	21.08
**2708**	Ever tobacco smoke	Khorasan_North	Faroj	Female	3.26	0.00	10.44
**2709**	Ever tobacco smoke	Chaharmahal	Farsan	Female	0.97	0.00	4.82
**2710**	Ever tobacco smoke	Kerman	Faryab	Female	5.68	0.00	15.53
**2711**	Ever tobacco smoke	Fars	Fasa	Female	10.61	2.23	18.46
**2712**	Ever tobacco smoke	Khorasan_South	Ferdows	Female	4.91	0.00	13.57
**2713**	Ever tobacco smoke	Mazandaran	Fereydunkenar	Female	1.64	0.00	8.80
**2714**	Ever tobacco smoke	Isfahan	Fereydunshahr	Female	4.53	0.00	13.48
**2715**	Ever tobacco smoke	Fars	Firozabad	Female	17.71	8.59	27.56
**2716**	Ever tobacco smoke	Tehran	Firuzkuh	Female	1.81	0.00	8.84
**2717**	Ever tobacco smoke	Sistan and Balouchestan	Fonuj	Female	20.59	1.47	40.54
**2718**	Ever tobacco smoke	Gilan	Fuman	Female	1.01	0.00	5.99
**2719**	Ever tobacco smoke	Kohkiluye and Bouyer Ahmad	Gachsaran	Female	21.80	4.15	40.67
**2720**	Ever tobacco smoke	Golestan	Galikesh	Female	6.28	0.00	17.09
**2721**	Ever tobacco smoke	Mazandaran	Galugah	Female	6.20	0.00	20.09
**2722**	Ever tobacco smoke	Semnan	Garmsar	Female	1.43	0.00	6.72
**2723**	Ever tobacco smoke	Boushehr	Genaveh	Female	18.38	4.63	32.69
**2724**	Ever tobacco smoke	Fars	Gerash	Female	17.47	5.79	29.03
**2725**	Ever tobacco smoke	Khorasan_North	Germeh	Female	5.08	0.00	15.81
**2726**	Ever tobacco smoke	Ardebil	Germi	Female	2.87	0.00	8.37
**2727**	Ever tobacco smoke	Kerman	Ghaleye-Ganj	Female	3.57	0.00	10.08
**2728**	Ever tobacco smoke	Kermanshah	Gilan-e-Gharb	Female	3.30	0.00	10.55
**2729**	Ever tobacco smoke	Isfahan	Golpayegan	Female	6.31	0.00	14.63
**2730**	Ever tobacco smoke	Golestan	Gomishan	Female	5.10	0.00	18.84
**2731**	Ever tobacco smoke	Khorasan_razavi	Gonabad	Female	12.60	2.47	22.95
**2732**	Ever tobacco smoke	Golestan	Gonbad-e-Kavus	Female	5.22	0.00	12.67
**2733**	Ever tobacco smoke	Golestan	Gorgan	Female	7.03	0.00	16.66
**2734**	Ever tobacco smoke	Khuzestan	Guotvand	Female	4.21	0.00	9.07
**2735**	Ever tobacco smoke	Khuzestan	Haftgol	Female	2.68	0.00	9.44
**2736**	Ever tobacco smoke	Hormozgan	Hajiabad	Female	13.81	1.14	26.46
**2737**	Ever tobacco smoke	Hamedan	Hamadan	Female	5.70	0.00	12.02
**2738**	Ever tobacco smoke	Khuzestan	Hamidiyeh	Female	2.58	0.00	10.03
**2739**	Ever tobacco smoke	Sistan and Balouchestan	Hamoon	Female	16.93	0.00	37.42

**2740**	Ever tobacco smoke	Azararbayjan_East	Haris	Female	3.28	0.00	7.91
**2741**	Ever tobacco smoke	Kermanshah	Harsin	Female	3.95	0.00	10.44
**2742**	Ever tobacco smoke	Azararbayjan_East	Hashtrud	Female	2.12	0.00	7.68
**2743**	Ever tobacco smoke	Khuzestan	Hendijan	Female	4.14	0.00	11.95
**2744**	Ever tobacco smoke	Sistan and Balouchestan	Hirmand	Female	18.14	0.00	39.25
**2745**	Ever tobacco smoke	Khuzestan	Hoveizeh	Female	2.34	0.00	9.95
**2746**	Ever tobacco smoke	Zanjan	Ijerod	Female	5.88	0.00	14.88
**2747**	Ever tobacco smoke	Ilam	Ilam	Female	1.64	0.00	7.20
**2748**	Ever tobacco smoke	Sistan and Balouchestan	Iranshahr	Female	20.97	4.32	38.46
**2749**	Ever tobacco smoke	Isfahan	Isfahan	Female	5.75	0.00	11.89
**2750**	Ever tobacco smoke	Khuzestan	Izeh	Female	2.65	0.00	7.71
**2751**	Ever tobacco smoke	Fars	Jahrom	Female	13.06	3.91	21.68
**2752**	Ever tobacco smoke	Khorasan_North	Jajarm	Female	5.77	0.00	15.66
**2753**	Ever tobacco smoke	Boushehr	Jam	Female	24.84	12.38	38.25
**2754**	Ever tobacco smoke	Kermanshah	Javanrud	Female	4.07	0.00	12.03
**2755**	Ever tobacco smoke	Kerman	Jiroft	Female	4.78	0.00	11.79
**2756**	Ever tobacco smoke	Khorasan_razavi	Joghatai	Female	9.55	0.00	20.43
**2757**	Ever tobacco smoke	Azararbayjan_East	Jolfa	Female	2.79	0.00	8.66
**2758**	Ever tobacco smoke	Khorasan_razavi	Jowayin	Female	9.76	0.00	20.38
**2759**	Ever tobacco smoke	Mazandaran	Juybar	Female	3.92	0.00	15.35
**2760**	Ever tobacco smoke	Hamedan	Kabudarahang	Female	2.85	0.00	8.09
**2761**	Ever tobacco smoke	Kerman	Kahnuj	Female	5.86	0.00	14.94
**2762**	Ever tobacco smoke	Golestan	Kalaleh	Female	6.78	0.00	17.90
**2763**	Ever tobacco smoke	Khorasan_razavi	Kalat	Female	12.74	1.21	24.57
**2764**	Ever tobacco smoke	Azararbayjan_East	Kaleibar	Female	10.48	5.00	15.87
**2765**	Ever tobacco smoke	Kordestan	Kamyaran	Female	2.70	0.00	10.19
**2766**	Ever tobacco smoke	Boushehr	Kangan	Female	21.62	8.14	36.12
**2767**	Ever tobacco smoke	Kermanshah	Kangavar	Female	6.76	1.81	11.77
**2768**	Ever tobacco smoke	Alborz	Karaj	Female	2.46	0.00	7.75
**2769**	Ever tobacco smoke	Khuzestan	Karun	Female	2.65	0.00	10.98
**2770**	Ever tobacco smoke	Isfahan	Kashan	Female	4.25	0.00	11.02
**2771**	Ever tobacco smoke	Khorasan_razavi	Kashmar	Female	12.12	2.41	22.00
**2772**	Ever tobacco smoke	Fars	Kavar	Female	15.33	5.14	26.25
**2773**	Ever tobacco smoke	Fars	Kazerun	Female	15.38	5.60	25.27
**2774**	Ever tobacco smoke	Mazandaran	Kelardasht	Female	3.49	0.00	15.01
**2775**	Ever tobacco smoke	Kerman	Kerman	Female	5.15	0.00	12.42
**2776**	Ever tobacco smoke	Kermanshah	Kermanshah	Female	3.26	0.00	8.16
**2777**	Ever tobacco smoke	Khorasan_razavi	Khaf	Female	15.50	5.26	26.31
**2778**	Ever tobacco smoke	Khorasan_razavi	Khalilabad	Female	11.23	0.00	22.43
**2779**	Ever tobacco smoke	Ardebil	Khalkhal	Female	1.25	0.00	6.11
**2780**	Ever tobacco smoke	Hormozgan	Khamir	Female	13.44	4.49	22.70
**2781**	Ever tobacco smoke	Isfahan	Khansar	Female	5.00	0.00	14.30
**2782**	Ever tobacco smoke	Sistan and Balouchestan	Khash	Female	21.83	3.47	41.24
**2783**	Ever tobacco smoke	Yazd	Khatam	Female	7.43	0.00	18.92
**2784**	Ever tobacco smoke	Fars	Kherameh	Female	12.20	2.38	22.07
**2785**	Ever tobacco smoke	Azararbayjan_East	Khodaafarin	Female	3.88	0.00	10.09
**2786**	Ever tobacco smoke	Zanjan	Khodabandeh	Female	5.33	0.00	12.58
**2787**	Ever tobacco smoke	Markazi	Khomeyn	Female	3.82	0.00	11.16
**2788**	Ever tobacco smoke	Isfahan	Khomeynishahr	Female	6.84	0.00	15.54
**2789**	Ever tobacco smoke	Markazi	Khondab	Female	3.71	0.00	11.32
**2790**	Ever tobacco smoke	Fars	Khonj	Female	14.19	7.16	21.38
**2791**	Ever tobacco smoke	Isfahan	Khoor va Biabanak	Female	5.34	0.00	14.03
**2792**	Ever tobacco smoke	Lorestan	Khorramabad	Female	7.82	0.00	19.06
**2793**	Ever tobacco smoke	Fars	Khorrambid	Female	10.91	0.83	21.35
**2794**	Ever tobacco smoke	Zanjan	Khorramdarreh	Female	4.67	0.00	12.33
**2795**	Ever tobacco smoke	Khuzestan	Khorramshahr	Female	2.30	0.00	7.47
**2796**	Ever tobacco smoke	Khorasan_razavi	Khoshab	Female	10.12	0.00	20.93
**2797**	Ever tobacco smoke	Azarbayjan_West	Khoy	Female	2.16	0.00	8.79
**2798**	Ever tobacco smoke	Khorasan_South	Khusef	Female	4.78	0.00	12.43
**2799**	Ever tobacco smoke	Chaharmahal	Kiaar	Female	1.09	0.00	4.86
**2800**	Ever tobacco smoke	Kohkiluye and Bouyer Ahmad	Kohgiluyeh	Female	16.21	1.61	30.97
**2801**	Ever tobacco smoke	Markazi	Komeijan	Female	6.32	0.00	16.88
**2802**	Ever tobacco smoke	Sistan and Balouchestan	Konarak	Female	24.77	4.68	45.90
**2803**	Ever tobacco smoke	Golestan	Kordkuy	Female	6.50	0.00	17.17
**2804**	Ever tobacco smoke	Ardebil	Kowsar	Female	1.27	0.00	8.13
**2805**	Ever tobacco smoke	Kerman	Kuhbonan	Female	2.95	0.00	12.64
**2806**	Ever tobacco smoke	Lorestan	Kuhdasht	Female	7.87	0.00	20.43
**2807**	Ever tobacco smoke	Chaharmahal	Kuhrang	Female	1.90	0.00	7.03
**2808**	Ever tobacco smoke	Gilan	Lahijan	Female	2.27	0.00	8.30
**2809**	Ever tobacco smoke	Khuzestan	Lali	Female	2.81	0.00	10.22
**2810**	Ever tobacco smoke	Fars	Lamard	Female	31.75	20.02	44.02
**2811**	Ever tobacco smoke	Kohkiluye and Bouyer Ahmad	Landeh	Female	17.09	0.00	38.72
**2812**	Ever tobacco smoke	Gilan	Langrud	Female	0.83	0.00	5.89
**2813**	Ever tobacco smoke	Isfahan	Lanjan	Female	2.78	0.00	8.72
**2814**	Ever tobacco smoke	Fars	Lar (Larestan)	Female	14.86	6.10	23.24
**2815**	Ever tobacco smoke	Chaharmahal	Lordakan	Female	2.77	0.00	7.14
**2816**	Ever tobacco smoke	Azarbayjan_West	Mahabad	Female	5.05	0.00	12.80
**2817**	Ever tobacco smoke	Markazi	Mahalat	Female	9.24	0.00	20.13
**2818**	Ever tobacco smoke	Mazandaran	Mahmudabad	Female	1.38	0.00	8.88
**2819**	Ever tobacco smoke	Zanjan	Mahneshan	Female	10.54	3.56	17.74
**2820**	Ever tobacco smoke	Khorasan_razavi	Mahvelat	Female	12.20	2.03	23.16
**2821**	Ever tobacco smoke	Azarbayjan_West	Maku	Female	4.86	0.00	14.16
**2822**	Ever tobacco smoke	Tehran	Malard	Female	3.53	0.00	11.45
**2823**	Ever tobacco smoke	Hamedan	Malayer	Female	5.95	0.00	12.98
**2824**	Ever tobacco smoke	Azararbayjan_East	Malekan	Female	0.92	0.00	4.81
**2825**	Ever tobacco smoke	Ilam	Malekshahi	Female	2.71	0.00	11.16
**2826**	Ever tobacco smoke	Fars	Mamasany	Female	14.39	5.13	23.86
**2827**	Ever tobacco smoke	Khorasan_North	Maneh and Samalqan	Female	4.83	0.00	12.48
**2828**	Ever tobacco smoke	Kerman	Manujan	Female	6.70	0.00	16.74
**2829**	Ever tobacco smoke	Azararbayjan_East	Maragheh	Female	1.14	0.00	4.78
**2830**	Ever tobacco smoke	Azararbayjan_East	Marand	Female	1.29	0.00	5.04
**2831**	Ever tobacco smoke	Golestan	Maravehtapeh	Female	5.87	0.00	18.31
**2832**	Ever tobacco smoke	Kordestan	Marivan	Female	3.79	0.00	11.78
**2833**	Ever tobacco smoke	Fars	Marvdasht	Female	9.22	1.18	16.97
**2834**	Ever tobacco smoke	Gilan	Masal	Female	1.64	0.00	9.35
**2835**	Ever tobacco smoke	Khorasan_razavi	Mashhad	Female	13.75	6.03	21.48
**2836**	Ever tobacco smoke	Khuzestan	Masjed Soleyman	Female	2.87	0.00	9.41
**2837**	Ever tobacco smoke	Semnan	Mayamey	Female	4.44	0.00	12.40

**2838**	Ever tobacco smoke	Semnan	Mehdishahr	Female	3.38	0.00	11.22
**2839**	Ever tobacco smoke	Ilam	Mehran	Female	1.40	0.00	7.15
**2840**	Ever tobacco smoke	Yazd	Mehriz	Female	6.41	0.00	17.51
**2841**	Ever tobacco smoke	Ardebil	Meshkinshahr	Female	1.19	0.00	5.74
**2842**	Ever tobacco smoke	Yazd	Meybod	Female	2.89	0.00	11.04
**2843**	Ever tobacco smoke	Hormozgan	Minab	Female	16.52	3.22	29.79
**2844**	Ever tobacco smoke	Golestan	Minudasht	Female	2.93	0.00	10.48
**2845**	Ever tobacco smoke	Sistan and Balouchestan	Mirjaveh	Female	20.12	0.00	43.07
**2846**	Ever tobacco smoke	Azarbayjan_West	Miyandoab	Female	3.85	0.00	10.24
**2847**	Ever tobacco smoke	Mazandaran	Miyandorud	Female	5.23	0.00	18.29
**2848**	Ever tobacco smoke	Azararbayjan_East	Miyaneh	Female	1.29	0.00	4.92
**2849**	Ever tobacco smoke	Isfahan	Mobarakeh	Female	5.93	0.00	14.23
**2850**	Ever tobacco smoke	Fars	Mohr	Female	18.28	8.17	29.10
**2851**	Ever tobacco smoke	Hamedan	Nahavand	Female	5.63	0.00	12.92
**2852**	Ever tobacco smoke	Isfahan	Najafabad	Female	5.30	0.00	12.30
**2853**	Ever tobacco smoke	Ardebil	Namin	Female	1.18	0.00	9.00
**2854**	Ever tobacco smoke	Azarbayjan_West	Naqadeh	Female	5.59	0.00	14.67
**2855**	Ever tobacco smoke	Kerman	Narmashir	Female	5.28	0.00	15.71
**2856**	Ever tobacco smoke	Isfahan	Natanz	Female	3.76	0.00	10.28
**2857**	Ever tobacco smoke	Isfahan	Nayin	Female	4.89	0.00	13.17
**2858**	Ever tobacco smoke	Alborz	Nazarabad	Female	2.16	0.00	8.08
**2859**	Ever tobacco smoke	Ardebil	Neer	Female	1.43	0.00	8.55
**2860**	Ever tobacco smoke	Khorasan_South	Nehbandan	Female	2.62	0.00	7.97
**2861**	Ever tobacco smoke	Mazandaran	Neka	Female	5.99	0.00	15.58
**2862**	Ever tobacco smoke	Fars	Neyriz	Female	9.17	1.22	16.75
**2863**	Ever tobacco smoke	Khorasan_razavi	Neyshabur	Female	10.67	2.49	18.81
**2864**	Ever tobacco smoke	Sistan and Balouchestan	Nikshahr	Female	23.59	6.06	42.42
**2865**	Ever tobacco smoke	Sistan and Balouchestan	Nimruz	Female	17.15	0.00	36.90
**2866**	Ever tobacco smoke	Mazandaran	Noshahr	Female	4.27	0.00	16.40
**2867**	Ever tobacco smoke	Mazandaran	Nur	Female	1.85	0.00	9.01
**2868**	Ever tobacco smoke	Khuzestan	Omidiyeh	Female	3.90	0.00	9.55
**2869**	Ever tobacco smoke	Azarbayjan_West	Orumiyeh	Female	5.84	0.00	14.30
**2870**	Ever tobacco smoke	Azarbayjan_West	Oshnaviyeh	Female	5.26	0.00	16.26
**2871**	Ever tobacco smoke	Azararbayjan_East	Osku	Female	1.77	0.00	7.16
**2872**	Ever tobacco smoke	Tehran	Pakdasht	Female	1.53	0.00	8.37
**2873**	Ever tobacco smoke	Tehran	Pardis	Female	2.63	0.00	13.70
**2874**	Ever tobacco smoke	Ardebil	Parsabad	Female	1.92	0.00	6.83
**2875**	Ever tobacco smoke	Hormozgan	Parsian (Gavbandi)	Female	20.57	6.35	35.25
**2876**	Ever tobacco smoke	Fars	Pasargad	Female	9.24	0.29	18.06
**2877**	Ever tobacco smoke	Kermanshah	Paveh	Female	3.58	0.00	11.33
**2878**	Ever tobacco smoke	Azarbayjan_West	Piranshahr	Female	5.26	0.00	15.77
**2879**	Ever tobacco smoke	Tehran	Pishva	Female	1.31	0.00	8.73
**2880**	Ever tobacco smoke	Azarbayjan_West	Poldasht	Female	4.77	0.00	13.47
**2881**	Ever tobacco smoke	Lorestan	Poldokhtar	Female	7.06	0.00	17.10
**2882**	Ever tobacco smoke	Mazandaran	Qaemshahr	Female	2.13	0.00	9.59
**2883**	Ever tobacco smoke	Tehran	Qarchak	Female	3.05	0.00	14.37
**2884**	Ever tobacco smoke	Sistan and Balouchestan	Qasr qand	Female	23.23	2.57	44.11
**2885**	Ever tobacco smoke	Kermanshah	Qasr-e-Shirin	Female	3.05	0.00	11.95
**2886**	Ever tobacco smoke	Khorasan_South	Qayenat	Female	6.28	0.00	13.69
**2887**	Ever tobacco smoke	Qazvin	Qazvin	Female	9.48	0.00	26.18
**2888**	Ever tobacco smoke	Hormozgan	Qeshm	Female	12.85	1.82	23.88
**2889**	Ever tobacco smoke	Fars	Qirokarzin	Female	15.34	5.92	24.81
**2890**	Ever tobacco smoke	Qom	Qom	Female	6.59	0.00	22.91
**2891**	Ever tobacco smoke	Kordestan	Qorveh	Female	5.93	0.00	15.56
**2892**	Ever tobacco smoke	Khorasan_razavi	Quchan	Female	11.87	1.05	22.40
**2893**	Ever tobacco smoke	Kerman	Rabar	Female	5.12	0.00	13.52
**2894**	Ever tobacco smoke	Kerman	Rafsanjan	Female	2.28	0.00	8.54
**2895**	Ever tobacco smoke	Khuzestan	Ramhormoz	Female	1.65	0.00	6.25
**2896**	Ever tobacco smoke	Mazandaran	Ramsar	Female	3.68	0.00	16.04
**2897**	Ever tobacco smoke	Khuzestan	Ramshir	Female	2.93	0.00	9.05
**2898**	Ever tobacco smoke	Golestan	Ramyan	Female	5.82	0.00	17.23
**2899**	Ever tobacco smoke	Gilan	Rasht	Female	1.02	0.00	5.77
**2900**	Ever tobacco smoke	Khorasan_razavi	Rashtkhar	Female	13.29	2.71	23.80
**2901**	Ever tobacco smoke	Kermanshah	Ravansar	Female	3.54	0.00	10.95
**2902**	Ever tobacco smoke	Kerman	Ravar	Female	1.71	0.00	7.86
**2903**	Ever tobacco smoke	Khorasan_North	Raz va Jergolan	Female	5.30	0.00	17.72
**2904**	Ever tobacco smoke	Hamedan	Razan	Female	2.64	0.00	7.99
**2905**	Ever tobacco smoke	Tehran	Rey	Female	4.63	0.00	13.89
**2906**	Ever tobacco smoke	Kerman	Reygan	Female	5.50	0.00	14.67
**2907**	Ever tobacco smoke	Gilan	Rezvanshahr	Female	1.82	0.00	9.31
**2908**	Ever tobacco smoke	Tehran	Robatkarim	Female	1.69	0.00	9.18
**2909**	Ever tobacco smoke	Fars	Rostam	Female	13.35	2.70	24.29
**2910**	Ever tobacco smoke	Kerman	Roudbar-e-Jonub	Female	3.11	0.00	9.63
**2911**	Ever tobacco smoke	Hormozgan	Rudan	Female	14.74	2.09	27.42
**2912**	Ever tobacco smoke	Gilan	Rudbar	Female	1.84	0.00	7.13
**2913**	Ever tobacco smoke	Gilan	Rudsar	Female	1.92	0.00	7.30
**2914**	Ever tobacco smoke	Lorestan	Rumshekan	Female	6.29	0.00	22.14
**2915**	Ever tobacco smoke	Khorasan_razavi	Sabzevar	Female	6.37	0.00	13.75
**2916**	Ever tobacco smoke	Yazd	Sadugh	Female	5.53	0.00	17.23
**2917**	Ever tobacco smoke	Kermanshah	Sahneh	Female	4.04	0.00	10.99
**2918**	Ever tobacco smoke	Kermanshah	Salas-e-Babajani	Female	3.38	0.00	10.72
**2919**	Ever tobacco smoke	Azarbayjan_West	Salmas	Female	6.14	0.00	15.82
**2920**	Ever tobacco smoke	Chaharmahal	Saman	Female	1.70	0.00	7.64
**2921**	Ever tobacco smoke	Kordestan	Sanandaj	Female	6.00	0.00	15.93
**2922**	Ever tobacco smoke	Kordestan	Saqqez	Female	6.79	0.00	17.00
**2923**	Ever tobacco smoke	Kermanshah	Sar-e-Pol-e-Zohab	Female	1.64	0.00	6.86
**2924**	Ever tobacco smoke	Azararbayjan_East	Sarab	Female	2.60	0.00	6.65
**2925**	Ever tobacco smoke	Khorasan_razavi	Sarakhs	Female	14.22	3.50	25.33
**2926**	Ever tobacco smoke	Sistan and Balouchestan	Saravan	Female	22.47	0.00	46.53
**2927**	Ever tobacco smoke	Khorasan_South	Sarayan	Female	4.94	0.00	12.65
**2928**	Ever tobacco smoke	Sistan and Balouchestan	Sarbaz	Female	21.47	4.24	39.23
**2929**	Ever tobacco smoke	Khorasan_South	Sarbisheh	Female	6.25	0.00	14.44
**2930**	Ever tobacco smoke	Azarbayjan_West	Sardasht	Female	6.04	0.00	15.85
**2931**	Ever tobacco smoke	Ardebil	Sarein	Female	1.57	0.00	8.57
**2932**	Ever tobacco smoke	Mazandaran	Sari	Female	5.61	0.00	15.01
**2933**	Ever tobacco smoke	Kordestan	Sarvabad	Female	4.71	0.00	16.38
**2934**	Ever tobacco smoke	Fars	Sarvestan	Female	13.46	3.21	23.55
**2935**	Ever tobacco smoke	Mazandaran	Savadkuh	Female	4.07	0.00	14.80

**2936**	Ever tobacco smoke	Mazandaran	Savadkuh_North	Female	4.12	0.00	15.35
**2937**	Ever tobacco smoke	Markazi	Saveh	Female	3.26	0.00	10.65
**2938**	Ever tobacco smoke	Alborz	Savojbolagh	Female	2.84	0.00	9.36
**2939**	Ever tobacco smoke	Lorestan	Selseleh	Female	7.23	0.00	18.63
**2940**	Ever tobacco smoke	Isfahan	Semirom	Female	6.43	0.00	13.88
**2941**	Ever tobacco smoke	Isfahan	Semirom-e-Sofla	Female	5.79	0.00	14.96
**2942**	Ever tobacco smoke	Semnan	Semnan	Female	3.72	0.00	10.09
**2943**	Ever tobacco smoke	Fars	Sepidan	Female	12.70	2.38	22.90
**2944**	Ever tobacco smoke	Azararbayjan_East	Shabestar	Female	1.26	0.00	5.08
**2945**	Ever tobacco smoke	Khuzestan	Shadegan	Female	1.54	0.00	6.50
**2946**	Ever tobacco smoke	Gilan	Shaft	Female	2.14	0.00	8.30
**2947**	Ever tobacco smoke	Azarbayjan_West	Shahindezh	Female	8.54	2.26	14.79
**2948**	Ever tobacco smoke	Tehran	Shahr-e Qods	Female	3.05	0.00	10.76
**2949**	Ever tobacco smoke	Kerman	Shahr-e-Babak	Female	4.75	0.00	13.74
**2950**	Ever tobacco smoke	Chaharmahal	Shahr-e-Kord	Female	1.50	0.00	5.32
**2951**	Ever tobacco smoke	Isfahan	Shahreza	Female	6.67	0.00	14.50
**2952**	Ever tobacco smoke	Tehran	Shahriyar	Female	4.28	0.00	12.64
**2953**	Ever tobacco smoke	Semnan	Shahrud	Female	4.96	0.00	11.08
**2954**	Ever tobacco smoke	Markazi	Shazand	Female	13.43	5.54	21.62
**2955**	Ever tobacco smoke	Tehran	Shemiranat	Female	3.07	0.00	12.11
**2956**	Ever tobacco smoke	Fars	Shiraz	Female	15.33	7.12	23.77
**2957**	Ever tobacco smoke	Khorasan_North	Shirvan	Female	4.13	0.00	11.67
**2958**	Ever tobacco smoke	Ilam	Shirvan and Chard-e-Aval	Female	1.38	0.00	6.72
**2959**	Ever tobacco smoke	Azarbayjan_West	Showt	Female	3.34	0.00	10.21
**2960**	Ever tobacco smoke	Khuzestan	Shush	Female	4.34	0.00	9.43
**2961**	Ever tobacco smoke	Khuzestan	Shushtar	Female	2.68	0.00	7.63
**2962**	Ever tobacco smoke	Gilan	Siahkal	Female	1.64	0.00	8.66
**2963**	Ever tobacco smoke	Sistan and Balouchestan	Sib o Soran	Female	21.51	0.00	43.95
**2964**	Ever tobacco smoke	Mazandaran	Simorgh	Female	3.60	0.00	14.92
**2965**	Ever tobacco smoke	Hormozgan	Sirik	Female	18.89	4.14	34.92
**2966**	Ever tobacco smoke	Kerman	Sirjan	Female	5.38	0.00	13.04
**2967**	Ever tobacco smoke	Ilam	Sirvan	Female	2.53	0.00	10.66
**2968**	Ever tobacco smoke	Zanjan	Soltaniyeh	Female	5.26	0.00	13.94
**2969**	Ever tobacco smoke	Kermanshah	Sonqor	Female	4.16	0.00	10.21
**2970**	Ever tobacco smoke	Semnan	Sorkheh	Female	3.35	0.00	11.41
**2971**	Ever tobacco smoke	Gilan	Sume'eh Sara	Female	1.57	0.00	6.93
**2972**	Ever tobacco smoke	Khorasan_South	Tabas	Female	2.28	0.00	7.34
**2973**	Ever tobacco smoke	Azararbayjan_East	Tabriz	Female	2.13	0.00	6.12
**2974**	Ever tobacco smoke	Markazi	Tafresh	Female	5.23	0.00	14.84
**2975**	Ever tobacco smoke	Yazd	Taft	Female	6.79	0.00	16.73
**2976**	Ever tobacco smoke	Azarbayjan_West	Takab	Female	2.87	0.00	9.20
**2977**	Ever tobacco smoke	Qazvin	Takestan	Female	3.36	0.00	16.77
**2978**	Ever tobacco smoke	Khorasan_razavi	Takht-e-Jolgeh (Firuzeh)	Female	10.50	0.00	21.51
**2979**	Ever tobacco smoke	Alborz	Taleghan	Female	1.38	0.00	6.65
**2980**	Ever tobacco smoke	Boushehr	Tangestan	Female	19.72	4.56	35.26
**2981**	Ever tobacco smoke	Zanjan	Tarom	Female	5.41	0.00	13.16
**2982**	Ever tobacco smoke	Gilan	Tavalesh	Female	0.99	0.00	5.96
**2983**	Ever tobacco smoke	Khorasan_razavi	Taybad	Female	20.86	9.35	34.17
**2984**	Ever tobacco smoke	Tehran	Tehran	Female	4.34	0.00	11.51
**2985**	Ever tobacco smoke	Isfahan	Tiran and Karvan	Female	4.63	0.00	13.43
**2986**	Ever tobacco smoke	Mazandaran	Tonekabon	Female	3.76	0.00	11.92
**2987**	Ever tobacco smoke	Khorasan_razavi	Torbat-e-Heydariyeh	Female	13.50	3.90	23.36
**2988**	Ever tobacco smoke	Khorasan_razavi	Torbat-e-Jam	Female	20.50	10.34	31.72
**2989**	Ever tobacco smoke	Hamedan	Tuyserkan	Female	2.72	0.00	8.10
**2990**	Ever tobacco smoke	Tehran	Varamin	Female	2.73	0.00	9.91
**2991**	Ever tobacco smoke	Azararbayjan_East	Varzaqan	Female	3.20	0.00	8.42
**2992**	Ever tobacco smoke	Yazd	Yazd	Female	6.21	0.00	15.00
**2993**	Ever tobacco smoke	Sistan and Balouchestan	Zabol	Female	13.03	0.00	28.05
**2994**	Ever tobacco smoke	Sistan and Balouchestan	Zaboli (Mehrestan)	Female	21.12	3.07	39.95
**2995**	Ever tobacco smoke	Sistan and Balouchestan	Zahedan	Female	21.06	4.71	37.76
**2996**	Ever tobacco smoke	Zanjan	Zanjan	Female	6.50	0.00	14.15
**2997**	Ever tobacco smoke	Kerman	Zarand	Female	3.51	0.00	10.87
**2998**	Ever tobacco smoke	Markazi	Zarandiyeh	Female	4.42	0.00	14.64
**2999**	Ever tobacco smoke	Fars	Zarrindasht	Female	13.73	2.98	24.57
**3000**	Ever tobacco smoke	Khorasan_razavi	Zave	Female	14.65	4.11	25.78
**3001**	Ever tobacco smoke	Sistan and Balouchestan	Zehak	Female	16.83	0.00	37.82
**3002**	Ever tobacco smoke	Khorasan_South	Zir kuh	Female	6.09	0.00	14.56
**3003**	Ever tobacco smoke	Khuzestan	Abadan	Male	32.36	16.26	48.79
**3004**	Ever tobacco smoke	Fars	Abadeh	Male	40.99	26.65	55.55
**3005**	Ever tobacco smoke	Yazd	Abarkuh	Male	42.32	24.27	61.66
**3006**	Ever tobacco smoke	Mazandaran	Abbas abad	Male	47.37	31.92	61.18
**3007**	Ever tobacco smoke	Ilam	Abdanan	Male	26.71	9.04	42.50
**3008**	Ever tobacco smoke	Zanjan	Abhar	Male	33.36	16.21	48.70
**3009**	Ever tobacco smoke	Hormozgan	Abumusa	Male	33.95	13.29	54.60
**3010**	Ever tobacco smoke	Qazvin	Abyek	Male	52.78	28.45	77.51
**3011**	Ever tobacco smoke	Azararbayjan_East	Ahar	Male	31.65	16.62	47.04
**3012**	Ever tobacco smoke	Khuzestan	Ahvaz	Male	31.98	18.54	45.87
**3013**	Ever tobacco smoke	Azararbayjan_East	Ajabshir	Male	31.15	13.77	47.62
**3014**	Ever tobacco smoke	Qazvin	Alborz	Male	48.60	25.01	71.27
**3015**	Ever tobacco smoke	Golestan	Aliabad	Male	35.72	20.29	52.06
**3016**	Ever tobacco smoke	Lorestan	Aligudarz	Male	35.13	16.25	53.00
**3017**	Ever tobacco smoke	Gilan	Amlash	Male	34.81	14.81	55.24
**3018**	Ever tobacco smoke	Mazandaran	Amol	Male	39.83	23.56	58.12
**3019**	Ever tobacco smoke	Kerman	Anar	Male	60.04	38.38	80.36
**3020**	Ever tobacco smoke	Kerman	Anbarabad	Male	33.30	15.66	49.55
**3021**	Ever tobacco smoke	Khuzestan	Andika	Male	15.92	0.00	33.41
**3022**	Ever tobacco smoke	Khuzestan	Andimeshk	Male	24.16	7.22	38.70
**3023**	Ever tobacco smoke	Golestan	Aq Qala	Male	36.33	17.56	54.87
**3024**	Ever tobacco smoke	Khuzestan	Aqajari	Male	30.92	10.99	51.17
**3025**	Ever tobacco smoke	Semnan	Aradan	Male	30.86	12.41	49.33
**3026**	Ever tobacco smoke	Markazi	Arak	Male	42.05	24.76	61.10
**3027**	Ever tobacco smoke	Isfahan	Aran and Bidgol	Male	37.34	18.37	55.35
**3028**	Ever tobacco smoke	Ardebil	Ardabil	Male	35.05	19.08	51.49
**3029**	Ever tobacco smoke	Yazd	Ardakan	Male	39.80	23.02	56.95
**3030**	Ever tobacco smoke	Chaharmahal	Ardal	Male	41.94	24.23	60.79
**3031**	Ever tobacco smoke	Isfahan	Ardestan	Male	38.71	19.15	57.82
**3032**	Ever tobacco smoke	Fars	Arsanjan	Male	42.48	26.62	59.70
**3033**	Ever tobacco smoke	Kerman	Arzouyeh	Male	43.44	29.86	57.73

**3034**	Ever tobacco smoke	Hamedan	Asadabad	Male	37.77	16.46	57.24
**3035**	Ever tobacco smoke	Boushehr	Asaluyeh	Male	32.80	14.81	50.75
**3036**	Ever tobacco smoke	Markazi	Ashtiyan	Male	39.34	16.58	61.46
**3037**	Ever tobacco smoke	Gilan	Astaneh-ye-Ashrafiyeh	Male	35.12	17.29	54.32
**3038**	Ever tobacco smoke	Gilan	Astara	Male	33.65	16.13	51.20
**3039**	Ever tobacco smoke	Qazvin	Avaj	Male	51.50	25.88	77.86
**3040**	Ever tobacco smoke	Golestan	Azadshahr	Male	35.30	17.05	54.37
**3041**	Ever tobacco smoke	Azararbayjan_East	Azarshahr	Male	31.86	14.55	50.08
**3042**	Ever tobacco smoke	Lorestan	Azna	Male	38.10	18.94	58.34
**3043**	Ever tobacco smoke	Mazandaran	Babol	Male	38.78	21.92	56.18
**3044**	Ever tobacco smoke	Mazandaran	Babolsar	Male	42.26	22.62	63.67
**3045**	Ever tobacco smoke	Ilam	Badreh	Male	28.03	8.43	46.98
**3046**	Ever tobacco smoke	Yazd	Bafq	Male	27.04	10.63	44.37
**3047**	Ever tobacco smoke	Kerman	Baft	Male	36.90	20.86	52.07
**3048**	Ever tobacco smoke	Khuzestan	Baghemalek	Male	29.05	11.80	45.29
**3049**	Ever tobacco smoke	Yazd	Bahabad	Male	37.73	17.35	57.40
**3050**	Ever tobacco smoke	Hamedan	Bahar	Male	44.04	25.19	64.88
**3051**	Ever tobacco smoke	Tehran	Baharestan (Golestan)	Male	33.69	19.22	49.40
**3052**	Ever tobacco smoke	Kohkiluye and Bouyer Ahmad	Bahmani	Male	31.16	11.09	49.84
**3053**	Ever tobacco smoke	Khorasan_razavi	Bajestan	Male	30.75	13.26	48.10
**3054**	Ever tobacco smoke	Khorasan_razavi	Bakhraz	Male	29.74	12.10	46.56
**3055**	Ever tobacco smoke	Kerman	Bam	Male	38.53	22.61	55.36
**3056**	Ever tobacco smoke	Hormozgan	Bandar-e-Abbas	Male	32.03	17.39	45.56
**3057**	Ever tobacco smoke	Gilan	Bandar-e-Anzali	Male	32.66	14.65	49.64
**3058**	Ever tobacco smoke	Golestan	Bandar-e-Gaz	Male	30.27	10.53	48.50
**3059**	Ever tobacco smoke	Hormozgan	Bandar-e-Jask	Male	35.71	18.99	53.67
**3060**	Ever tobacco smoke	Hormozgan	Bandar-e-Lengeh	Male	34.22	17.23	51.86
**3061**	Ever tobacco smoke	Khuzestan	Bandar-e-Mahshahr	Male	32.61	16.99	49.93
**3062**	Ever tobacco smoke	Golestan	Bandar-e-Torkaman	Male	34.78	16.26	53.28
**3063**	Ever tobacco smoke	Kordestan	Baneh	Male	45.80	26.02	68.17
**3064**	Ever tobacco smoke	Khorasan_razavi	Bardeskan	Male	32.39	16.25	50.13
**3065**	Ever tobacco smoke	Kerman	Bardsir	Male	38.91	21.76	56.34
**3066**	Ever tobacco smoke	Hormozgan	Bashagerd	Male	44.56	29.33	59.69
**3067**	Ever tobacco smoke	Kohkiluye and Bouyer Ahmad	Basht	Male	27.61	8.54	48.43
**3068**	Ever tobacco smoke	Hormozgan	Bastak	Male	34.01	16.64	52.32
**3069**	Ever tobacco smoke	Khuzestan	Bavi	Male	30.02	13.63	46.22
**3070**	Ever tobacco smoke	Khuzestan	Behbahan	Male	31.95	14.79	49.96
**3071**	Ever tobacco smoke	Mazandaran	Behshahr	Male	31.97	10.35	50.13
**3072**	Ever tobacco smoke	Kordestan	Bijar	Male	31.73	13.92	46.98
**3073**	Ever tobacco smoke	Ardebil	Bilehsavar	Male	32.57	12.07	51.59
**3074**	Ever tobacco smoke	Khorasan_razavi	Binaloud	Male	35.08	17.46	54.11
**3075**	Ever tobacco smoke	Khorasan_South	Birjand	Male	17.29	5.50	28.23
**3076**	Ever tobacco smoke	Khorasan_North	Bojnurd	Male	24.22	11.90	35.36
**3077**	Ever tobacco smoke	Chaharmahal	Bon	Male	41.44	19.15	65.80
**3078**	Ever tobacco smoke	Azararbayjan_East	Bonab	Male	32.84	18.20	48.03
**3079**	Ever tobacco smoke	Isfahan	Borkhar	Male	41.33	21.18	61.49
**3080**	Ever tobacco smoke	Isfahan	Borkhar and Meymeh	Male	40.46	22.75	58.03
**3081**	Ever tobacco smoke	Chaharmahal	Borujen	Male	39.93	21.61	57.80
**3082**	Ever tobacco smoke	Lorestan	Borujerd	Male	39.38	21.84	58.85
**3083**	Ever tobacco smoke	Khorasan_South	Boshruyeh	Male	17.41	3.70	29.74
**3084**	Ever tobacco smoke	Azararbayjan_East	Bostanabad	Male	33.27	16.83	50.63
**3085**	Ever tobacco smoke	Fars	Bovanat	Male	28.41	9.31	44.60
**3086**	Ever tobacco smoke	Kohkiluye and Bouyer Ahmad	Boyer Ahmad	Male	46.25	26.46	67.61
**3087**	Ever tobacco smoke	Qazvin	Boyinzahra	Male	60.11	39.20	86.12
**3088**	Ever tobacco smoke	Isfahan	Buein va Miandasht	Male	39.48	18.54	60.17
**3089**	Ever tobacco smoke	Azarbayjan_West	Bukan	Male	47.53	28.25	68.30
**3090**	Ever tobacco smoke	Boushehr	Bushehr	Male	31.27	15.54	46.67
**3091**	Ever tobacco smoke	Isfahan	Chadegan	Male	40.58	21.04	59.78
**3092**	Ever tobacco smoke	Sistan and Balouchestan	Chahbahar	Male	25.12	8.91	39.66
**3093**	Ever tobacco smoke	Azarbayjan_West	Chaipareh	Male	46.34	23.75	69.52
**3094**	Ever tobacco smoke	Azarbayjan_West	Chaldoran	Male	46.93	23.40	72.65
**3095**	Ever tobacco smoke	Mazandaran	Chalus	Male	41.42	20.92	61.06
**3096**	Ever tobacco smoke	Azararbayjan_East	Charoimaq	Male	31.63	15.02	48.37
**3097**	Ever tobacco smoke	Khorasan_razavi	Chenaran	Male	34.44	17.76	52.72
**3098**	Ever tobacco smoke	Kohkiluye and Bouyer Ahmad	Cheram	Male	36.67	21.35	52.99
**3099**	Ever tobacco smoke	Kermanshah	Dalaho	Male	27.31	7.44	46.97
**3100**	Ever tobacco smoke	Lorestan	Dalfan	Male	34.71	17.02	52.34
**3101**	Ever tobacco smoke	Sistan and Balouchestan	Dalgan	Male	29.44	14.75	44.98
**3102**	Ever tobacco smoke	Tehran	Damavand	Male	31.29	15.05	48.22
**3103**	Ever tobacco smoke	Semnan	Damghan	Male	33.07	17.03	50.45
**3104**	Ever tobacco smoke	Fars	Darab	Male	41.20	25.30	58.91
**3105**	Ever tobacco smoke	Khorasan_South	Darmian	Male	16.35	2.96	28.59
**3106**	Ever tobacco smoke	Khorasan_razavi	Darrehgaz	Male	28.35	12.52	42.83
**3107**	Ever tobacco smoke	Ilam	Darrehshahr	Male	25.07	10.07	39.38
**3108**	Ever tobacco smoke	Khuzestan	Dasht-e-Azadegan	Male	29.60	10.12	48.26
**3109**	Ever tobacco smoke	Boushehr	Dashtestan	Male	32.97	18.28	48.10
**3110**	Ever tobacco smoke	Boushehr	Dashti	Male	27.63	10.79	41.86
**3111**	Ever tobacco smoke	Khorasan_razavi	Davarzan	Male	32.07	13.51	50.85
**3112**	Ever tobacco smoke	Boushehr	Dayyer	Male	49.44	31.39	66.25
**3113**	Ever tobacco smoke	Kordestan	Dehgolan	Male	36.95	19.27	54.62
**3114**	Ever tobacco smoke	Ilam	Dehloran	Male	28.17	10.67	45.62
**3115**	Ever tobacco smoke	Markazi	Delijan	Male	40.83	20.28	62.01
**3116**	Ever tobacco smoke	Kohkiluye and Bouyer Ahmad	Dena	Male	45.91	22.39	70.55
**3117**	Ever tobacco smoke	Boushehr	Deylam	Male	32.13	14.42	50.17
**3118**	Ever tobacco smoke	Khuzestan	Dezful	Male	25.66	10.17	39.45
**3119**	Ever tobacco smoke	Kordestan	Divandarreh	Male	47.53	29.14	68.85
**3120**	Ever tobacco smoke	Lorestan	Dorud	Male	37.34	17.89	57.94
**3121**	Ever tobacco smoke	Lorestan	Doureh	Male	33.60	13.88	51.66
**3122**	Ever tobacco smoke	Fars	Eqlid	Male	39.77	21.52	58.43
**3123**	Ever tobacco smoke	Khorasan_North	Esfarayen	Male	26.88	15.00	38.82
**3124**	Ever tobacco smoke	Alborz	Eshtehard	Male	37.01	17.72	56.15
**3125**	Ever tobacco smoke	Kermanshah	Eslamabad-e-Gharb	Male	25.32	9.84	39.94
**3126**	Ever tobacco smoke	Tehran	Eslamshahr	Male	34.23	19.60	49.99
**3127**	Ever tobacco smoke	Fars	Estahban	Male	37.28	17.91	55.32
**3128**	Ever tobacco smoke	Ilam	Eyvan	Male	23.64	6.56	38.96
**3129**	Ever tobacco smoke	Kerman	Fahraj	Male	41.06	24.04	60.06
**3130**	Ever tobacco smoke	Isfahan	Falavarjan	Male	42.44	24.67	60.51
**3131**	Ever tobacco smoke	Hamedan	Famenin	Male	41.64	20.86	64.65

**3132**	Ever tobacco smoke	Markazi	Farahan	Male	39.15	17.18	62.32
**3133**	Ever tobacco smoke	Fars	Farashband	Male	38.26	19.04	57.64
**3134**	Ever tobacco smoke	Alborz	Fardis	Male	35.74	16.50	55.32
**3135**	Ever tobacco smoke	Isfahan	Faridan	Male	41.97	23.60	61.69
**3136**	Ever tobacco smoke	Khorasan_razavi	Fariman	Male	25.35	9.03	39.42
**3137**	Ever tobacco smoke	Khorasan_North	Faroj	Male	30.12	17.16	44.26
**3138**	Ever tobacco smoke	Chaharmahal	Farsan	Male	41.63	22.42	61.92
**3139**	Ever tobacco smoke	Kerman	Faryab	Male	37.10	18.04	56.56
**3140**	Ever tobacco smoke	Fars	Fasa	Male	41.65	25.05	60.21
**3141**	Ever tobacco smoke	Khorasan_South	Ferdows	Male	14.06	1.94	25.54
**3142**	Ever tobacco smoke	Mazandaran	Fereydunkenar	Male	36.14	14.91	55.24
**3143**	Ever tobacco smoke	Isfahan	Fereydunshahr	Male	41.08	21.79	61.24
**3144**	Ever tobacco smoke	Fars	Firozabad	Male	44.08	27.16	64.09
**3145**	Ever tobacco smoke	Tehran	Firuzkuh	Male	25.91	9.38	40.29
**3146**	Ever tobacco smoke	Sistan and Balouchestan	Fonuj	Male	28.90	10.14	46.66
**3147**	Ever tobacco smoke	Gilan	Fuman	Male	30.24	13.70	44.98
**3148**	Ever tobacco smoke	Kohkiluye and Bouyer Ahmad	Gachsaran	Male	44.08	21.96	67.99
**3149**	Ever tobacco smoke	Golestan	Galikesh	Male	35.46	15.80	54.11
**3150**	Ever tobacco smoke	Mazandaran	Galugah	Male	36.62	12.86	60.81
**3151**	Ever tobacco smoke	Semnan	Garmsar	Male	28.44	12.31	43.32
**3152**	Ever tobacco smoke	Boushehr	Genaveh	Male	31.27	14.67	47.49
**3153**	Ever tobacco smoke	Fars	Gerash	Male	38.09	17.59	59.19
**3154**	Ever tobacco smoke	Khorasan_North	Germeh	Male	27.15	10.57	43.76
**3155**	Ever tobacco smoke	Ardebil	Germi	Male	37.30	19.46	56.66
**3156**	Ever tobacco smoke	Kerman	Ghaleye-Ganj	Male	34.91	16.87	51.57
**3157**	Ever tobacco smoke	Kermanshah	Gilan-e-Gharb	Male	24.52	11.19	37.06
**3158**	Ever tobacco smoke	Isfahan	Golpayegan	Male	41.96	23.60	61.81
**3159**	Ever tobacco smoke	Golestan	Gomishan	Male	35.59	14.36	56.44
**3160**	Ever tobacco smoke	Khorasan_razavi	Gonabad	Male	27.27	10.42	42.87
**3161**	Ever tobacco smoke	Golestan	Gonbad-e-Kavus	Male	34.91	18.02	51.61
**3162**	Ever tobacco smoke	Golestan	Gorgan	Male	36.33	21.25	52.36
**3163**	Ever tobacco smoke	Khuzestan	Guotvand	Male	28.01	9.23	44.75
**3164**	Ever tobacco smoke	Khuzestan	Haftgol	Male	41.34	25.94	56.30
**3165**	Ever tobacco smoke	Hormozgan	Hajiabad	Male	35.42	19.15	52.17
**3166**	Ever tobacco smoke	Hamedan	Hamadan	Male	45.54	27.87	65.41
**3167**	Ever tobacco smoke	Khuzestan	Hamidiyeh	Male	30.01	10.19	49.53
**3168**	Ever tobacco smoke	Sistan and Balouchestan	Hamoon	Male	25.71	6.70	44.86
**3169**	Ever tobacco smoke	Azararbayjan_East	Haris	Male	34.59	19.71	51.58
**3170**	Ever tobacco smoke	Kermanshah	Harsin	Male	27.52	8.95	44.20
**3171**	Ever tobacco smoke	Azararbayjan_East	Hashtrud	Male	32.43	16.05	50.04
**3172**	Ever tobacco smoke	Khuzestan	Hendijan	Male	31.22	11.06	50.77
**3173**	Ever tobacco smoke	Sistan and Balouchestan	Hirmand	Male	22.45	3.22	38.56
**3174**	Ever tobacco smoke	Khuzestan	Hoveizeh	Male	30.34	10.33	50.65
**3175**	Ever tobacco smoke	Zanjan	Ijerod	Male	37.18	21.52	52.82
**3176**	Ever tobacco smoke	Ilam	Ilam	Male	27.82	11.55	44.11
**3177**	Ever tobacco smoke	Sistan and Balouchestan	Iranshahr	Male	30.99	17.94	46.13
**3178**	Ever tobacco smoke	Isfahan	Isfahan	Male	40.39	26.71	53.80
**3179**	Ever tobacco smoke	Khuzestan	Izeh	Male	29.36	12.69	44.64
**3180**	Ever tobacco smoke	Fars	Jahrom	Male	39.75	22.17	57.75
**3181**	Ever tobacco smoke	Khorasan_North	Jajarm	Male	28.64	14.75	43.77
**3182**	Ever tobacco smoke	Boushehr	Jam	Male	33.45	16.57	49.65
**3183**	Ever tobacco smoke	Kermanshah	Javanrud	Male	28.56	9.66	46.19
**3184**	Ever tobacco smoke	Kerman	Jiroft	Male	38.09	22.47	53.68
**3185**	Ever tobacco smoke	Khorasan_razavi	Joghatai	Male	31.94	12.52	50.54
**3186**	Ever tobacco smoke	Azararbayjan_East	Jolfa	Male	29.98	12.60	46.38
**3187**	Ever tobacco smoke	Khorasan_razavi	Jowayin	Male	31.94	12.96	49.99
**3188**	Ever tobacco smoke	Mazandaran	Juybar	Male	37.63	16.64	57.93
**3189**	Ever tobacco smoke	Hamedan	Kabudarahang	Male	41.49	20.66	63.01
**3190**	Ever tobacco smoke	Kerman	Kahnuj	Male	30.00	12.94	45.06
**3191**	Ever tobacco smoke	Golestan	Kalaleh	Male	35.42	18.38	53.00
**3192**	Ever tobacco smoke	Khorasan_razavi	Kalat	Male	31.55	11.74	51.09
**3193**	Ever tobacco smoke	Azararbayjan_East	Kaleibar	Male	31.49	12.95	49.66
**3194**	Ever tobacco smoke	Kordestan	Kamyaran	Male	37.77	16.84	59.24
**3195**	Ever tobacco smoke	Boushehr	Kangan	Male	34.19	18.11	50.89
**3196**	Ever tobacco smoke	Kermanshah	Kangavar	Male	33.70	18.29	50.83
**3197**	Ever tobacco smoke	Alborz	Karaj	Male	35.20	22.02	48.21
**3198**	Ever tobacco smoke	Khuzestan	Karun	Male	30.63	9.42	51.27
**3199**	Ever tobacco smoke	Isfahan	Kashan	Male	37.77	20.83	54.23
**3200**	Ever tobacco smoke	Khorasan_razavi	Kashmar	Male	31.20	14.55	48.23
**3201**	Ever tobacco smoke	Fars	Kavar	Male	37.82	19.35	56.27
**3202**	Ever tobacco smoke	Fars	Kazerun	Male	34.53	18.03	49.78
**3203**	Ever tobacco smoke	Mazandaran	Kelardasht	Male	42.83	20.24	66.11
**3204**	Ever tobacco smoke	Kerman	Kerman	Male	37.04	22.72	51.66
**3205**	Ever tobacco smoke	Kermanshah	Kermanshah	Male	27.48	14.08	40.08
**3206**	Ever tobacco smoke	Khorasan_razavi	Khaf	Male	31.45	14.85	48.14
**3207**	Ever tobacco smoke	Khorasan_razavi	Khalilabad	Male	31.86	15.04	48.54
**3208**	Ever tobacco smoke	Ardebil	Khalkhal	Male	35.28	17.10	53.72
**3209**	Ever tobacco smoke	Hormozgan	Khamir	Male	36.23	23.53	49.21
**3210**	Ever tobacco smoke	Isfahan	Khansar	Male	36.61	17.48	54.31
**3211**	Ever tobacco smoke	Sistan and Balouchestan	Khash	Male	35.11	20.81	51.61
**3212**	Ever tobacco smoke	Yazd	Khatam	Male	42.15	23.66	62.62
**3213**	Ever tobacco smoke	Fars	Kherameh	Male	36.83	21.05	51.59
**3214**	Ever tobacco smoke	Azararbayjan_East	Khodaafarin	Male	31.39	12.71	49.74
**3215**	Ever tobacco smoke	Zanjan	Khodabandeh	Male	36.75	20.47	52.78
**3216**	Ever tobacco smoke	Markazi	Khomeyn	Male	42.13	25.48	60.07
**3217**	Ever tobacco smoke	Isfahan	Khomeynishahr	Male	43.09	26.25	61.59
**3218**	Ever tobacco smoke	Markazi	Khondab	Male	31.70	11.67	49.84
**3219**	Ever tobacco smoke	Fars	Khonj	Male	37.60	18.10	56.16
**3220**	Ever tobacco smoke	Isfahan	Khoor va Biabanak	Male	38.90	18.38	59.67
**3221**	Ever tobacco smoke	Lorestan	Khorramabad	Male	34.39	17.45	51.39
**3222**	Ever tobacco smoke	Fars	Khorrambid	Male	41.64	22.64	61.45
**3223**	Ever tobacco smoke	Zanjan	Khorramdarreh	Male	41.35	23.40	61.86
**3224**	Ever tobacco smoke	Khuzestan	Khorramshahr	Male	35.25	18.24	54.00
**3225**	Ever tobacco smoke	Khorasan_razavi	Khoshab	Male	31.33	13.12	49.38
**3226**	Ever tobacco smoke	Azarbayjan_West	Khoy	Male	48.98	30.18	68.66
**3227**	Ever tobacco smoke	Khorasan_South	Khusef	Male	20.90	6.43	35.22
**3228**	Ever tobacco smoke	Chaharmahal	Kiaar	Male	44.96	26.23	65.90
**3229**	Ever tobacco smoke	Kohkiluye and Bouyer Ahmad	Kohgiluyeh	Male	42.41	21.31	64.05

**3230**	Ever tobacco smoke	Markazi	Komeijan	Male	39.27	16.47	61.44
**3231**	Ever tobacco smoke	Sistan and Balouchestan	Konarak	Male	30.30	14.96	46.50
**3232**	Ever tobacco smoke	Golestan	Kordkuy	Male	33.34	15.79	50.19
**3233**	Ever tobacco smoke	Ardebil	Kowsar	Male	35.87	15.60	56.10
**3234**	Ever tobacco smoke	Kerman	Kuhbonan	Male	40.71	23.70	60.04
**3235**	Ever tobacco smoke	Lorestan	Kuhdasht	Male	32.96	15.15	51.02
**3236**	Ever tobacco smoke	Chaharmahal	Kuhrang	Male	40.02	19.06	60.47
**3237**	Ever tobacco smoke	Gilan	Lahijan	Male	36.14	18.34	54.25
**3238**	Ever tobacco smoke	Khuzestan	Lali	Male	30.01	11.19	48.98
**3239**	Ever tobacco smoke	Fars	Lamard	Male	40.42	25.42	57.55
**3240**	Ever tobacco smoke	Kohkiluye and Bouyer Ahmad	Landeh	Male	40.13	11.96	68.14
**3241**	Ever tobacco smoke	Gilan	Langrud	Male	33.31	15.55	50.56
**3242**	Ever tobacco smoke	Isfahan	Lanjan	Male	42.33	25.46	61.45
**3243**	Ever tobacco smoke	Fars	Lar (Larestan)	Male	38.72	20.73	56.68
**3244**	Ever tobacco smoke	Chaharmahal	Lordakan	Male	43.11	26.67	60.77
**3245**	Ever tobacco smoke	Azarbayjan_West	Mahabad	Male	44.87	23.56	64.57
**3246**	Ever tobacco smoke	Markazi	Mahalat	Male	41.71	20.63	63.77
**3247**	Ever tobacco smoke	Mazandaran	Mahmudabad	Male	33.36	15.08	50.68
**3248**	Ever tobacco smoke	Zanjan	Mahneshan	Male	38.77	18.76	59.41
**3249**	Ever tobacco smoke	Khorasan_razavi	Mahvelat	Male	31.14	13.49	49.79
**3250**	Ever tobacco smoke	Azarbayjan_West	Maku	Male	47.16	25.15	70.35
**3251**	Ever tobacco smoke	Tehran	Malard	Male	34.28	19.08	50.42
**3252**	Ever tobacco smoke	Hamedan	Malayer	Male	45.24	25.84	67.06
**3253**	Ever tobacco smoke	Azararbayjan_East	Malekan	Male	31.26	15.71	46.93
**3254**	Ever tobacco smoke	Ilam	Malekshahi	Male	27.34	7.48	47.54
**3255**	Ever tobacco smoke	Fars	Mamasany	Male	40.76	24.11	59.23
**3256**	Ever tobacco smoke	Khorasan_North	Maneh and Samalqan	Male	25.91	11.77	39.02
**3257**	Ever tobacco smoke	Kerman	Manujan	Male	37.35	18.43	56.49
**3258**	Ever tobacco smoke	Azararbayjan_East	Maragheh	Male	35.28	21.02	51.88
**3259**	Ever tobacco smoke	Azararbayjan_East	Marand	Male	29.44	13.26	44.23
**3260**	Ever tobacco smoke	Golestan	Maravehtapeh	Male	45.37	28.55	63.91
**3261**	Ever tobacco smoke	Kordestan	Marivan	Male	39.30	19.18	60.04
**3262**	Ever tobacco smoke	Fars	Marvdasht	Male	34.54	17.13	49.46
**3263**	Ever tobacco smoke	Gilan	Masal	Male	34.00	15.88	52.26
**3264**	Ever tobacco smoke	Khorasan_razavi	Mashhad	Male	28.08	15.25	40.05
**3265**	Ever tobacco smoke	Khuzestan	Masjed Soleyman	Male	33.75	18.63	51.83
**3266**	Ever tobacco smoke	Semnan	Mayamey	Male	31.11	13.04	49.28
**3267**	Ever tobacco smoke	Semnan	Mehdishahr	Male	30.11	13.85	46.23
**3268**	Ever tobacco smoke	Ilam	Mehran	Male	23.16	10.76	36.89
**3269**	Ever tobacco smoke	Yazd	Mehriz	Male	44.23	27.22	63.33
**3270**	Ever tobacco smoke	Ardebil	Meshkinshahr	Male	35.87	17.18	54.08
**3271**	Ever tobacco smoke	Yazd	Meybod	Male	44.38	26.33	65.09
**3272**	Ever tobacco smoke	Hormozgan	Minab	Male	30.01	14.01	44.57
**3273**	Ever tobacco smoke	Golestan	Minudasht	Male	34.23	15.56	52.04
**3274**	Ever tobacco smoke	Sistan and Balouchestan	Mirjaveh	Male	28.82	9.61	48.52
**3275**	Ever tobacco smoke	Azarbayjan_West	Miyandoab	Male	46.48	26.93	66.11
**3276**	Ever tobacco smoke	Mazandaran	Miyandorud	Male	37.91	13.40	61.79
**3277**	Ever tobacco smoke	Azararbayjan_East	Miyaneh	Male	28.81	12.54	43.12
**3278**	Ever tobacco smoke	Isfahan	Mobarakeh	Male	45.40	27.41	66.03
**3279**	Ever tobacco smoke	Fars	Mohr	Male	26.72	10.69	43.11
**3280**	Ever tobacco smoke	Hamedan	Nahavand	Male	40.43	20.86	60.65
**3281**	Ever tobacco smoke	Isfahan	Najafabad	Male	39.57	21.63	56.74
**3282**	Ever tobacco smoke	Ardebil	Namin	Male	37.62	17.59	59.04
**3283**	Ever tobacco smoke	Azarbayjan_West	Naqadeh	Male	47.26	26.46	68.39
**3284**	Ever tobacco smoke	Kerman	Narmashir	Male	35.56	16.92	52.83
**3285**	Ever tobacco smoke	Isfahan	Natanz	Male	38.95	19.71	57.37
**3286**	Ever tobacco smoke	Isfahan	Nayin	Male	41.06	24.41	59.20
**3287**	Ever tobacco smoke	Alborz	Nazarabad	Male	39.04	22.42	56.44
**3288**	Ever tobacco smoke	Ardebil	Neer	Male	36.64	17.98	56.00
**3289**	Ever tobacco smoke	Khorasan_South	Nehbandan	Male	20.54	6.98	33.50
**3290**	Ever tobacco smoke	Mazandaran	Neka	Male	37.30	17.02	56.86
**3291**	Ever tobacco smoke	Fars	Neyriz	Male	38.79	22.91	54.52
**3292**	Ever tobacco smoke	Khorasan_razavi	Neyshabur	Male	24.54	8.52	37.73
**3293**	Ever tobacco smoke	Sistan and Balouchestan	Nikshahr	Male	25.37	9.83	38.98
**3294**	Ever tobacco smoke	Sistan and Balouchestan	Nimruz	Male	26.05	7.14	43.81
**3295**	Ever tobacco smoke	Mazandaran	Noshahr	Male	42.01	19.34	64.63
**3296**	Ever tobacco smoke	Mazandaran	Nur	Male	36.74	17.39	55.20
**3297**	Ever tobacco smoke	Khuzestan	Omidiyeh	Male	32.48	15.35	51.16
**3298**	Ever tobacco smoke	Azarbayjan_West	Orumiyeh	Male	46.42	28.69	64.17
**3299**	Ever tobacco smoke	Azarbayjan_West	Oshnaviyeh	Male	48.38	26.85	72.18
**3300**	Ever tobacco smoke	Azararbayjan_East	Osku	Male	30.01	13.92	44.74
**3301**	Ever tobacco smoke	Tehran	Pakdasht	Male	29.46	14.41	43.70
**3302**	Ever tobacco smoke	Tehran	Pardis	Male	31.02	12.33	49.72
**3303**	Ever tobacco smoke	Ardebil	Parsabad	Male	34.71	14.72	54.89
**3304**	Ever tobacco smoke	Hormozgan	Parsian (Gavbandi)	Male	30.70	19.27	42.52
**3305**	Ever tobacco smoke	Fars	Pasargad	Male	50.98	34.11	71.52
**3306**	Ever tobacco smoke	Kermanshah	Paveh	Male	29.81	11.81	48.46
**3307**	Ever tobacco smoke	Azarbayjan_West	Piranshahr	Male	46.68	24.76	69.87
**3308**	Ever tobacco smoke	Tehran	Pishva	Male	27.03	11.77	41.12
**3309**	Ever tobacco smoke	Azarbayjan_West	Poldasht	Male	46.14	28.31	64.69
**3310**	Ever tobacco smoke	Lorestan	Poldokhtar	Male	32.51	12.32	52.33
**3311**	Ever tobacco smoke	Mazandaran	Qaemshahr	Male	39.11	19.85	57.78
**3312**	Ever tobacco smoke	Tehran	Qarchak	Male	31.19	11.46	50.29
**3313**	Ever tobacco smoke	Sistan and Balouchestan	Qasr qand	Male	28.17	9.57	46.55
**3314**	Ever tobacco smoke	Kermanshah	Qasr-e-Shirin	Male	26.67	4.80	47.58
**3315**	Ever tobacco smoke	Khorasan_South	Qayenat	Male	21.88	9.57	34.58
**3316**	Ever tobacco smoke	Qazvin	Qazvin	Male	59.35	39.76	83.33
**3317**	Ever tobacco smoke	Hormozgan	Qeshm	Male	35.11	18.58	52.78
**3318**	Ever tobacco smoke	Fars	Qirokarzin	Male	39.87	21.63	59.18
**3319**	Ever tobacco smoke	Qom	Qom	Male	34.82	15.15	54.29
**3320**	Ever tobacco smoke	Kordestan	Qorveh	Male	32.86	13.38	50.32
**3321**	Ever tobacco smoke	Khorasan_razavi	Quchan	Male	41.05	25.28	60.49
**3322**	Ever tobacco smoke	Kerman	Rabar	Male	43.20	26.28	63.30
**3323**	Ever tobacco smoke	Kerman	Rafsanjan	Male	41.02	24.72	58.59
**3324**	Ever tobacco smoke	Khuzestan	Ramhormoz	Male	28.37	12.07	43.26
**3325**	Ever tobacco smoke	Mazandaran	Ramsar	Male	43.68	23.04	66.12
**3326**	Ever tobacco smoke	Khuzestan	Ramshir	Male	31.87	14.13	51.12
**3327**	Ever tobacco smoke	Golestan	Ramyan	Male	36.00	16.85	55.37

**3328**	Ever tobacco smoke	Gilan	Rasht	Male	30.83	17.04	44.22
**3329**	Ever tobacco smoke	Khorasan_razavi	Rashtkhar	Male	37.12	22.74	53.85
**3330**	Ever tobacco smoke	Kermanshah	Ravansar	Male	28.77	9.64	47.55
**3331**	Ever tobacco smoke	Kerman	Ravar	Male	33.92	19.83	47.53
**3332**	Ever tobacco smoke	Khorasan_North	Raz va Jergolan	Male	26.50	8.63	43.78
**3333**	Ever tobacco smoke	Hamedan	Razan	Male	43.32	23.63	63.51
**3334**	Ever tobacco smoke	Tehran	Rey	Male	33.14	17.92	50.46
**3335**	Ever tobacco smoke	Kerman	Reygan	Male	36.63	18.79	54.35
**3336**	Ever tobacco smoke	Gilan	Rezvanshahr	Male	36.50	19.75	54.34
**3337**	Ever tobacco smoke	Tehran	Robatkarim	Male	28.19	12.59	42.43
**3338**	Ever tobacco smoke	Fars	Rostam	Male	41.43	24.62	59.86
**3339**	Ever tobacco smoke	Kerman	Roudbar-e-Jonub	Male	36.56	18.30	54.58
**3340**	Ever tobacco smoke	Hormozgan	Rudan	Male	20.64	3.80	36.46
**3341**	Ever tobacco smoke	Gilan	Rudbar	Male	34.56	16.99	51.80
**3342**	Ever tobacco smoke	Gilan	Rudsar	Male	34.70	19.00	50.47
**3343**	Ever tobacco smoke	Lorestan	Rumshekan	Male	32.71	10.83	54.37
**3344**	Ever tobacco smoke	Khorasan_razavi	Sabzevar	Male	30.23	15.40	44.03
**3345**	Ever tobacco smoke	Yazd	Sadugh	Male	52.98	35.43	71.90
**3346**	Ever tobacco smoke	Kermanshah	Sahneh	Male	28.44	16.70	40.65
**3347**	Ever tobacco smoke	Kermanshah	Salas-e-Babajani	Male	24.57	9.06	38.92
**3348**	Ever tobacco smoke	Azarbayjan_West	Salmas	Male	51.59	31.41	74.86
**3349**	Ever tobacco smoke	Chaharmahal	Saman	Male	41.86	19.28	65.21
**3350**	Ever tobacco smoke	Kordestan	Sanandaj	Male	35.77	18.05	52.78
**3351**	Ever tobacco smoke	Kordestan	Saqqez	Male	45.11	25.63	66.90
**3352**	Ever tobacco smoke	Kermanshah	Sar-e-Pol-e-Zohab	Male	24.49	6.60	40.70
**3353**	Ever tobacco smoke	Azararbayjan_East	Sarab	Male	29.92	14.57	44.36
**3354**	Ever tobacco smoke	Khorasan_razavi	Sarakhs	Male	33.55	15.62	52.26
**3355**	Ever tobacco smoke	Sistan and Balouchestan	Saravan	Male	31.06	13.29	50.15
**3356**	Ever tobacco smoke	Khorasan_South	Sarayan	Male	20.26	5.75	34.65
**3357**	Ever tobacco smoke	Sistan and Balouchestan	Sarbaz	Male	26.36	10.77	41.58
**3358**	Ever tobacco smoke	Khorasan_South	Sarbisheh	Male	22.65	11.45	34.70
**3359**	Ever tobacco smoke	Azarbayjan_West	Sardasht	Male	49.66	29.07	71.52
**3360**	Ever tobacco smoke	Ardebil	Sarein	Male	35.92	15.60	56.03
**3361**	Ever tobacco smoke	Mazandaran	Sari	Male	39.07	22.03	57.28
**3362**	Ever tobacco smoke	Kordestan	Sarvabad	Male	37.86	14.19	61.15
**3363**	Ever tobacco smoke	Fars	Sarvestan	Male	38.89	18.69	58.92
**3364**	Ever tobacco smoke	Mazandaran	Savadkuh	Male	41.05	21.64	61.38
**3365**	Ever tobacco smoke	Mazandaran	Savadkuh_North	Male	38.61	17.05	60.65
**3366**	Ever tobacco smoke	Markazi	Saveh	Male	38.94	20.61	57.70
**3367**	Ever tobacco smoke	Alborz	Savojbolagh	Male	37.80	20.56	55.78
**3368**	Ever tobacco smoke	Lorestan	Selseleh	Male	34.97	15.40	54.48
**3369**	Ever tobacco smoke	Isfahan	Semirom	Male	45.34	29.78	62.01
**3370**	Ever tobacco smoke	Isfahan	Semirom-e-Sofla	Male	42.35	21.65	63.67
**3371**	Ever tobacco smoke	Semnan	Semnan	Male	30.06	14.42	45.37
**3372**	Ever tobacco smoke	Fars	Sepidan	Male	41.50	23.57	61.12
**3373**	Ever tobacco smoke	Azararbayjan_East	Shabestar	Male	27.85	11.79	42.49
**3374**	Ever tobacco smoke	Khuzestan	Shadegan	Male	25.13	8.41	39.84
**3375**	Ever tobacco smoke	Gilan	Shaft	Male	34.49	20.19	49.08
**3376**	Ever tobacco smoke	Azarbayjan_West	Shahindezh	Male	38.47	17.84	56.47
**3377**	Ever tobacco smoke	Tehran	Shahr-e Qods	Male	29.95	14.30	45.18
**3378**	Ever tobacco smoke	Kerman	Shahr-e-Babak	Male	38.82	20.19	56.38
**3379**	Ever tobacco smoke	Chaharmahal	Shahr-e-Kord	Male	45.02	27.80	64.09
**3380**	Ever tobacco smoke	Isfahan	Shahreza	Male	43.93	25.09	64.03
**3381**	Ever tobacco smoke	Tehran	Shahriyar	Male	34.16	20.31	49.99
**3382**	Ever tobacco smoke	Semnan	Shahrud	Male	31.16	16.30	45.72
**3383**	Ever tobacco smoke	Markazi	Shazand	Male	42.54	24.45	62.16
**3384**	Ever tobacco smoke	Tehran	Shemiranat	Male	30.24	12.84	47.13
**3385**	Ever tobacco smoke	Fars	Shiraz	Male	38.82	24.57	52.52
**3386**	Ever tobacco smoke	Khorasan_North	Shirvan	Male	22.30	8.49	35.00
**3387**	Ever tobacco smoke	Ilam	Shirvan and Chard-e-Aval	Male	28.81	12.82	44.66
**3388**	Ever tobacco smoke	Azarbayjan_West	Showt	Male	53.13	31.51	77.71
**3389**	Ever tobacco smoke	Khuzestan	Shush	Male	29.73	13.34	46.60
**3390**	Ever tobacco smoke	Khuzestan	Shushtar	Male	29.52	13.45	44.92
**3391**	Ever tobacco smoke	Gilan	Siahkal	Male	35.54	17.89	54.32
**3392**	Ever tobacco smoke	Sistan and Balouchestan	Sib o Soran	Male	27.32	9.48	44.53
**3393**	Ever tobacco smoke	Mazandaran	Simorgh	Male	38.95	16.37	62.06
**3394**	Ever tobacco smoke	Hormozgan	Sirik	Male	41.41	27.68	54.25
**3395**	Ever tobacco smoke	Kerman	Sirjan	Male	39.27	23.97	54.88
**3396**	Ever tobacco smoke	Ilam	Sirvan	Male	27.74	7.88	47.97
**3397**	Ever tobacco smoke	Zanjan	Soltaniyeh	Male	38.24	18.68	58.73
**3398**	Ever tobacco smoke	Kermanshah	Sonqor	Male	32.41	16.55	50.41
**3399**	Ever tobacco smoke	Semnan	Sorkheh	Male	31.09	12.97	49.44
**3400**	Ever tobacco smoke	Gilan	Sume'eh Sara	Male	35.77	19.01	53.29
**3401**	Ever tobacco smoke	Khorasan_South	Tabas	Male	18.70	6.12	29.48
**3402**	Ever tobacco smoke	Azararbayjan_East	Tabriz	Male	31.26	18.67	43.77
**3403**	Ever tobacco smoke	Markazi	Tafresh	Male	32.53	11.08	51.00
**3404**	Ever tobacco smoke	Yazd	Taft	Male	41.23	22.80	59.78
**3405**	Ever tobacco smoke	Azarbayjan_West	Takab	Male	44.87	22.64	66.12
**3406**	Ever tobacco smoke	Qazvin	Takestan	Male	54.96	31.09	80.56
**3407**	Ever tobacco smoke	Khorasan_razavi	Takht-e-Jolgeh (Firuzeh)	Male	27.31	13.73	40.71
**3408**	Ever tobacco smoke	Alborz	Taleghan	Male	40.86	24.04	60.18
**3409**	Ever tobacco smoke	Boushehr	Tangestan	Male	32.45	13.52	50.61
**3410**	Ever tobacco smoke	Zanjan	Tarom	Male	38.15	20.27	56.24
**3411**	Ever tobacco smoke	Gilan	Tavalesh	Male	35.26	19.06	52.53
**3412**	Ever tobacco smoke	Khorasan_razavi	Taybad	Male	31.58	13.68	50.23
**3413**	Ever tobacco smoke	Tehran	Tehran	Male	29.25	17.79	40.69
**3414**	Ever tobacco smoke	Isfahan	Tiran and Karvan	Male	41.73	22.74	62.06
**3415**	Ever tobacco smoke	Mazandaran	Tonekabon	Male	45.57	25.79	67.12
**3416**	Ever tobacco smoke	Khorasan_razavi	Torbat-e-Heydariyeh	Male	33.25	18.11	50.12
**3417**	Ever tobacco smoke	Khorasan_razavi	Torbat-e-Jam	Male	32.58	16.64	49.51
**3418**	Ever tobacco smoke	Hamedan	Tuyserkan	Male	33.22	13.37	50.44
**3419**	Ever tobacco smoke	Tehran	Varamin	Male	32.18	18.55	46.68
**3420**	Ever tobacco smoke	Azararbayjan_East	Varzaqan	Male	31.61	13.63	48.90
**3421**	Ever tobacco smoke	Yazd	Yazd	Male	38.64	23.40	53.49
**3422**	Ever tobacco smoke	Sistan and Balouchestan	Zabol	Male	17.62	1.76	32.67
**3423**	Ever tobacco smoke	Sistan and Balouchestan	Zaboli (Mehrestan)	Male	23.22	11.70	35.52
**3424**	Ever tobacco smoke	Sistan and Balouchestan	Zahedan	Male	30.14	16.16	44.80
**3425**	Ever tobacco smoke	Zanjan	Zanjan	Male	40.46	25.70	55.90

**3426**	Ever tobacco smoke	Kerman	Zarand	Male	31.45	12.90	46.63
**3427**	Ever tobacco smoke	Markazi	Zarandiyeh	Male	42.80	22.62	64.98
**3428**	Ever tobacco smoke	Fars	Zarrindasht	Male	39.80	20.77	59.36
**3429**	Ever tobacco smoke	Khorasan_razavi	Zave	Male	35.99	23.14	50.22
**3430**	Ever tobacco smoke	Sistan and Balouchestan	Zehak	Male	24.57	2.17	44.40
**3431**	Ever tobacco smoke	Khorasan_South	Zir kuh	Male	20.06	5.41	34.94
**3432**	Exposure to secondhand smoke	Khuzestan	Abadan	Female	29.05	7.33	49.83
**3433**	Exposure to secondhand smoke	Fars	Abadeh	Female	48.56	27.84	69.69
**3434**	Exposure to secondhand smoke	Yazd	Abarkuh	Female	41.44	20.85	62.55
**3435**	Exposure to secondhand smoke	Mazandaran	Abbas abad	Female	41.41	19.91	60.66
**3436**	Exposure to secondhand smoke	Ilam	Abdanan	Female	28.56	14.42	43.16
**3437**	Exposure to secondhand smoke	Zanjan	Abhar	Female	33.73	13.04	54.06
**3438**	Exposure to secondhand smoke	Hormozgan	Abumusa	Female	32.16	0.00	67.89
**3439**	Exposure to secondhand smoke	Qazvin	Abyek	Female	49.36	21.21	79.11
**3440**	Exposure to secondhand smoke	Azararbayjan_East	Ahar	Female	20.90	0.00	42.55
**3441**	Exposure to secondhand smoke	Khuzestan	Ahvaz	Female	31.97	16.89	46.91
**3442**	Exposure to secondhand smoke	Azararbayjan_East	Ajabshir	Female	26.19	0.70	51.23
**3443**	Exposure to secondhand smoke	Qazvin	Alborz	Female	54.45	24.58	84.89
**3444**	Exposure to secondhand smoke	Golestan	Aliabad	Female	20.54	1.54	39.55
**3445**	Exposure to secondhand smoke	Lorestan	Aligudarz	Female	29.87	11.00	47.97
**3446**	Exposure to secondhand smoke	Gilan	Amlash	Female	13.27	0.00	40.10
**3447**	Exposure to secondhand smoke	Mazandaran	Amol	Female	28.55	4.13	53.86
**3448**	Exposure to secondhand smoke	Kerman	Anar	Female	34.36	4.00	63.22
**3449**	Exposure to secondhand smoke	Kerman	Anbarabad	Female	34.40	13.51	55.45
**3450**	Exposure to secondhand smoke	Khuzestan	Andika	Female	35.50	13.15	59.80
**3451**	Exposure to secondhand smoke	Khuzestan	Andimeshk	Female	29.64	9.18	49.36
**3452**	Exposure to secondhand smoke	Golestan	Aq Qala	Female	12.24	0.00	29.51
**3453**	Exposure to secondhand smoke	Khuzestan	Aqajari	Female	32.37	3.77	61.41
**3454**	Exposure to secondhand smoke	Semnan	Aradan	Female	19.66	0.00	44.41
**3455**	Exposure to secondhand smoke	Markazi	Arak	Female	32.58	15.41	49.46
**3456**	Exposure to secondhand smoke	Isfahan	Aran and Bidgol	Female	19.30	0.62	37.78
**3457**	Exposure to secondhand smoke	Ardebil	Ardabil	Female	39.42	17.94	60.46
**3458**	Exposure to secondhand smoke	Yazd	Ardakan	Female	28.61	10.97	46.58
**3459**	Exposure to secondhand smoke	Chaharmahal	Ardal	Female	39.68	15.64	65.13
**3460**	Exposure to secondhand smoke	Isfahan	Ardestan	Female	25.71	2.20	48.48
**3461**	Exposure to secondhand smoke	Fars	Arsanjan	Female	45.93	23.08	69.03
**3462**	Exposure to secondhand smoke	Kerman	Arzouyeh	Female	38.19	13.40	62.79
**3463**	Exposure to secondhand smoke	Hamedan	Asadabad	Female	27.51	5.41	50.21
**3464**	Exposure to secondhand smoke	Boushehr	Asaluyeh	Female	34.72	10.00	59.67
**3465**	Exposure to secondhand smoke	Markazi	Ashtiyan	Female	28.98	3.66	54.99
**3466**	Exposure to secondhand smoke	Gilan	Astaneh-ye-Ashrafiyeh	Female	17.68	0.00	40.85
**3467**	Exposure to secondhand smoke	Gilan	Astara	Female	30.56	6.50	54.96
**3468**	Exposure to secondhand smoke	Qazvin	Avaj	Female	45.99	15.37	75.99
**3469**	Exposure to secondhand smoke	Golestan	Azadshahr	Female	16.27	0.00	32.85
**3470**	Exposure to secondhand smoke	Azararbayjan_East	Azarshahr	Female	26.91	0.00	56.68
**3471**	Exposure to secondhand smoke	Lorestan	Azna	Female	38.77	11.12	67.53
**3472**	Exposure to secondhand smoke	Mazandaran	Babol	Female	30.94	7.49	55.63
**3473**	Exposure to secondhand smoke	Mazandaran	Babolsar	Female	28.20	0.00	61.08
**3474**	Exposure to secondhand smoke	Ilam	Badreh	Female	26.49	4.13	49.38
**3475**	Exposure to secondhand smoke	Yazd	Bafq	Female	20.38	7.50	35.68
**3476**	Exposure to secondhand smoke	Kerman	Baft	Female	49.22	28.85	70.51
**3477**	Exposure to secondhand smoke	Khuzestan	Baghemalek	Female	19.20	3.74	36.42
**3478**	Exposure to secondhand smoke	Yazd	Bahabad	Female	24.21	0.00	48.56
**3479**	Exposure to secondhand smoke	Hamedan	Bahar	Female	43.19	16.31	69.98
**3480**	Exposure to secondhand smoke	Tehran	Baharestan (Golestan)	Female	25.97	5.05	47.76
**3481**	Exposure to secondhand smoke	Kohkiluye and Bouyer Ahmad	Bahmani	Female	38.95	14.03	63.86
**3482**	Exposure to secondhand smoke	Khorasan_razavi	Bajestan	Female	24.34	2.88	45.52
**3483**	Exposure to secondhand smoke	Khorasan_razavi	Bakhraz	Female	33.64	14.43	52.98
**3484**	Exposure to secondhand smoke	Kerman	Bam	Female	33.00	13.37	53.48
**3485**	Exposure to secondhand smoke	Hormozgan	Bandar-e-Abbas	Female	30.32	12.39	48.26
**3486**	Exposure to secondhand smoke	Gilan	Bandar-e-Anzali	Female	18.01	0.00	38.40
**3487**	Exposure to secondhand smoke	Golestan	Bandar-e-Gaz	Female	17.29	0.00	43.71
**3488**	Exposure to secondhand smoke	Hormozgan	Bandar-e-Jask	Female	39.34	13.62	66.44
**3489**	Exposure to secondhand smoke	Hormozgan	Bandar-e-Lengeh	Female	30.66	8.31	52.47
**3490**	Exposure to secondhand smoke	Khuzestan	Bandar-e-Mahshahr	Female	32.71	13.09	51.93
**3491**	Exposure to secondhand smoke	Golestan	Bandar-e-Torkaman	Female	15.60	0.00	40.26
**3492**	Exposure to secondhand smoke	Kordestan	Baneh	Female	51.33	22.02	81.56
**3493**	Exposure to secondhand smoke	Khorasan_razavi	Bardeskan	Female	15.15	0.00	32.42
**3494**	Exposure to secondhand smoke	Kerman	Bardsir	Female	39.71	21.21	58.10
**3495**	Exposure to secondhand smoke	Hormozgan	Bashagerd	Female	41.11	18.54	65.05
**3496**	Exposure to secondhand smoke	Kohkiluye and Bouyer Ahmad	Basht	Female	57.13	34.71	79.91
**3497**	Exposure to secondhand smoke	Hormozgan	Bastak	Female	35.54	9.89	61.92
**3498**	Exposure to secondhand smoke	Khuzestan	Bavi	Female	20.82	1.67	40.63
**3499**	Exposure to secondhand smoke	Khuzestan	Behbahan	Female	28.08	9.55	46.20
**3500**	Exposure to secondhand smoke	Mazandaran	Behshahr	Female	8.61	0.00	32.68
**3501**	Exposure to secondhand smoke	Kordestan	Bijar	Female	44.85	22.43	66.68
**3502**	Exposure to secondhand smoke	Ardebil	Bilehsavar	Female	32.98	0.00	75.77
**3503**	Exposure to secondhand smoke	Khorasan_razavi	Binaloud	Female	42.51	24.15	57.67
**3504**	Exposure to secondhand smoke	Khorasan_South	Birjand	Female	17.09	1.12	32.92
**3505**	Exposure to secondhand smoke	Khorasan_North	Bojnurd	Female	22.60	5.03	39.63
**3506**	Exposure to secondhand smoke	Chaharmahal	Bon	Female	31.44	0.00	63.80
**3507**	Exposure to secondhand smoke	Azararbayjan_East	Bonab	Female	36.47	10.35	63.37
**3508**	Exposure to secondhand smoke	Isfahan	Borkhar	Female	19.57	1.14	38.08
**3509**	Exposure to secondhand smoke	Isfahan	Borkhar and Meymeh	Female	32.58	14.38	51.96
**3510**	Exposure to secondhand smoke	Chaharmahal	Borujen	Female	26.86	8.25	45.24
**3511**	Exposure to secondhand smoke	Lorestan	Borujerd	Female	42.34	21.65	62.76
**3512**	Exposure to secondhand smoke	Khorasan_South	Boshruyeh	Female	12.81	0.00	30.76
**3513**	Exposure to secondhand smoke	Azararbayjan_East	Bostanabad	Female	27.03	6.38	48.26
**3514**	Exposure to secondhand smoke	Fars	Bovanat	Female	48.79	27.27	70.13
**3515**	Exposure to secondhand smoke	Kohkiluye and Bouyer Ahmad	Boyer Ahmad	Female	55.68	34.98	77.80
**3516**	Exposure to secondhand smoke	Qazvin	Boyinzahra	Female	53.15	28.18	79.23
**3517**	Exposure to secondhand smoke	Isfahan	Buein va Miandasht	Female	30.58	3.89	56.34
**3518**	Exposure to secondhand smoke	Azarbayjan_West	Bukan	Female	49.54	27.03	72.55
**3519**	Exposure to secondhand smoke	Boushehr	Bushehr	Female	31.54	11.54	51.42
**3520**	Exposure to secondhand smoke	Isfahan	Chadegan	Female	34.72	12.71	57.78
**3521**	Exposure to secondhand smoke	Sistan and Balouchestan	Chahbahar	Female	40.03	15.49	64.91
**3522**	Exposure to secondhand smoke	Azarbayjan_West	Chaipareh	Female	25.91	0.00	53.17
**3523**	Exposure to secondhand smoke	Azarbayjan_West	Chaldoran	Female	26.77	0.00	57.01

**3524**	Exposure to secondhand smoke	Mazandaran	Chalus	Female	32.43	7.40	57.67
**3525**	Exposure to secondhand smoke	Azararbayjan_East	Charoimaq	Female	37.94	12.45	63.37
**3526**	Exposure to secondhand smoke	Khorasan_razavi	Chenaran	Female	30.78	8.20	53.29
**3527**	Exposure to secondhand smoke	Kohkiluye and Bouyer Ahmad	Cheram	Female	50.23	22.95	77.60
**3528**	Exposure to secondhand smoke	Kermanshah	Dalaho	Female	23.18	4.90	41.33
**3529**	Exposure to secondhand smoke	Lorestan	Dalfan	Female	42.04	20.80	64.32
**3530**	Exposure to secondhand smoke	Sistan and Balouchestan	Dalgan	Female	29.72	4.65	53.87
**3531**	Exposure to secondhand smoke	Tehran	Damavand	Female	13.48	0.00	31.82
**3532**	Exposure to secondhand smoke	Semnan	Damghan	Female	21.93	2.50	42.85
**3533**	Exposure to secondhand smoke	Fars	Darab	Female	49.15	29.67	69.27
**3534**	Exposure to secondhand smoke	Khorasan_South	Darmian	Female	16.53	0.00	34.93
**3535**	Exposure to secondhand smoke	Khorasan_razavi	Darrehgaz	Female	15.77	0.00	35.03
**3536**	Exposure to secondhand smoke	Ilam	Darrehshahr	Female	21.64	1.63	41.54
**3537**	Exposure to secondhand smoke	Khuzestan	Dasht-e-Azadegan	Female	45.61	20.72	71.15
**3538**	Exposure to secondhand smoke	Boushehr	Dashtestan	Female	35.40	16.90	55.09
**3539**	Exposure to secondhand smoke	Boushehr	Dashti	Female	34.86	13.64	56.43
**3540**	Exposure to secondhand smoke	Khorasan_razavi	Davarzan	Female	23.61	0.00	50.13
**3541**	Exposure to secondhand smoke	Boushehr	Dayyer	Female	33.76	4.42	63.24
**3542**	Exposure to secondhand smoke	Kordestan	Dehgolan	Female	45.65	21.63	72.13
**3543**	Exposure to secondhand smoke	Ilam	Dehloran	Female	28.16	8.52	47.79
**3544**	Exposure to secondhand smoke	Markazi	Delijan	Female	33.77	12.23	56.35
**3545**	Exposure to secondhand smoke	Kohkiluye and Bouyer Ahmad	Dena	Female	55.23	29.09	83.45
**3546**	Exposure to secondhand smoke	Boushehr	Deylam	Female	33.26	9.06	56.89
**3547**	Exposure to secondhand smoke	Khuzestan	Dezful	Female	33.55	17.05	50.23
**3548**	Exposure to secondhand smoke	Kordestan	Divandarreh	Female	49.11	25.72	72.99
**3549**	Exposure to secondhand smoke	Lorestan	Dorud	Female	39.25	15.01	64.20
**3550**	Exposure to secondhand smoke	Lorestan	Doureh	Female	27.24	12.05	44.07
**3551**	Exposure to secondhand smoke	Fars	Eqlid	Female	48.42	27.50	68.89
**3552**	Exposure to secondhand smoke	Khorasan_North	Esfarayen	Female	25.21	6.26	45.10
**3553**	Exposure to secondhand smoke	Alborz	Eshtehard	Female	24.35	5.17	44.44
**3554**	Exposure to secondhand smoke	Kermanshah	Eslamabad-e-Gharb	Female	9.50	0.00	28.24
**3555**	Exposure to secondhand smoke	Tehran	Eslamshahr	Female	14.24	0.00	31.69
**3556**	Exposure to secondhand smoke	Fars	Estahban	Female	44.41	20.68	68.75
**3557**	Exposure to secondhand smoke	Ilam	Eyvan	Female	28.49	6.47	51.05
**3558**	Exposure to secondhand smoke	Kerman	Fahraj	Female	36.37	12.32	62.44
**3559**	Exposure to secondhand smoke	Isfahan	Falavarjan	Female	39.01	18.18	62.00
**3560**	Exposure to secondhand smoke	Hamedan	Famenin	Female	37.91	10.69	65.50
**3561**	Exposure to secondhand smoke	Markazi	Farahan	Female	29.62	4.10	54.75
**3562**	Exposure to secondhand smoke	Fars	Farashband	Female	46.07	24.43	67.86
**3563**	Exposure to secondhand smoke	Alborz	Fardis	Female	19.94	0.00	42.75
**3564**	Exposure to secondhand smoke	Isfahan	Faridan	Female	34.93	13.95	56.81
**3565**	Exposure to secondhand smoke	Khorasan_razavi	Fariman	Female	21.70	2.53	40.75
**3566**	Exposure to secondhand smoke	Khorasan_North	Faroj	Female	29.34	7.31	52.72
**3567**	Exposure to secondhand smoke	Chaharmahal	Farsan	Female	33.56	8.00	58.77
**3568**	Exposure to secondhand smoke	Kerman	Faryab	Female	34.38	10.22	59.70
**3569**	Exposure to secondhand smoke	Fars	Fasa	Female	50.93	32.10	70.94
**3570**	Exposure to secondhand smoke	Khorasan_South	Ferdows	Female	13.34	0.00	30.47
**3571**	Exposure to secondhand smoke	Mazandaran	Fereydunkenar	Female	28.20	0.00	58.82
**3572**	Exposure to secondhand smoke	Isfahan	Fereydunshahr	Female	29.51	6.01	52.52
**3573**	Exposure to secondhand smoke	Fars	Firozabad	Female	57.68	38.69	77.04
**3574**	Exposure to secondhand smoke	Tehran	Firuzkuh	Female	8.56	0.00	25.26
**3575**	Exposure to secondhand smoke	Sistan and Balouchestan	Fonuj	Female	33.19	4.41	61.71
**3576**	Exposure to secondhand smoke	Gilan	Fuman	Female	15.80	0.00	32.47
**3577**	Exposure to secondhand smoke	Kohkiluye and Bouyer Ahmad	Gachsaran	Female	53.17	31.50	76.10
**3578**	Exposure to secondhand smoke	Golestan	Galikesh	Female	21.63	1.07	43.05
**3579**	Exposure to secondhand smoke	Mazandaran	Galugah	Female	19.19	0.00	59.56
**3580**	Exposure to secondhand smoke	Semnan	Garmsar	Female	19.03	0.00	37.22
**3581**	Exposure to secondhand smoke	Boushehr	Genaveh	Female	30.92	9.93	51.13
**3582**	Exposure to secondhand smoke	Fars	Gerash	Female	46.96	17.67	76.57
**3583**	Exposure to secondhand smoke	Khorasan_North	Germeh	Female	30.18	14.06	45.01
**3584**	Exposure to secondhand smoke	Ardebil	Germi	Female	33.90	5.16	63.77
**3585**	Exposure to secondhand smoke	Kerman	Ghaleye-Ganj	Female	25.61	6.48	43.84
**3586**	Exposure to secondhand smoke	Kermanshah	Gilan-e-Gharb	Female	27.78	8.81	48.01
**3587**	Exposure to secondhand smoke	Isfahan	Golpayegan	Female	31.83	10.92	52.54
**3588**	Exposure to secondhand smoke	Golestan	Gomishan	Female	14.71	0.00	46.99
**3589**	Exposure to secondhand smoke	Khorasan_razavi	Gonabad	Female	31.60	17.22	45.37
**3590**	Exposure to secondhand smoke	Golestan	Gonbad-e-Kavus	Female	17.25	1.33	33.47
**3591**	Exposure to secondhand smoke	Golestan	Gorgan	Female	14.35	0.00	29.21
**3592**	Exposure to secondhand smoke	Khuzestan	Guotvand	Female	31.90	13.09	50.88
**3593**	Exposure to secondhand smoke	Khuzestan	Haftgol	Female	30.36	6.72	53.79
**3594**	Exposure to secondhand smoke	Hormozgan	Hajiabad	Female	39.83	20.19	60.31
**3595**	Exposure to secondhand smoke	Hamedan	Hamadan	Female	40.49	20.45	60.61
**3596**	Exposure to secondhand smoke	Khuzestan	Hamidiyeh	Female	44.74	18.24	72.14
**3597**	Exposure to secondhand smoke	Sistan and Balouchestan	Hamoon	Female	21.78	0.00	51.67
**3598**	Exposure to secondhand smoke	Azararbayjan_East	Haris	Female	25.00	3.42	46.56
**3599**	Exposure to secondhand smoke	Kermanshah	Harsin	Female	60.33	36.40	79.94
**3600**	Exposure to secondhand smoke	Azararbayjan_East	Hashtrud	Female	34.55	9.71	59.63
**3601**	Exposure to secondhand smoke	Khuzestan	Hendijan	Female	34.19	6.70	61.04
**3602**	Exposure to secondhand smoke	Sistan and Balouchestan	Hirmand	Female	19.92	0.00	48.57
**3603**	Exposure to secondhand smoke	Khuzestan	Hoveizeh	Female	61.75	39.74	77.59
**3604**	Exposure to secondhand smoke	Zanjan	Ijerod	Female	54.15	31.81	77.11
**3605**	Exposure to secondhand smoke	Ilam	Ilam	Female	26.71	8.32	45.60
**3606**	Exposure to secondhand smoke	Sistan and Balouchestan	Iranshahr	Female	35.68	14.47	57.66
**3607**	Exposure to secondhand smoke	Isfahan	Isfahan	Female	27.62	13.45	41.60
**3608**	Exposure to secondhand smoke	Khuzestan	Izeh	Female	32.85	14.42	51.67
**3609**	Exposure to secondhand smoke	Fars	Jahrom	Female	43.42	23.66	62.31
**3610**	Exposure to secondhand smoke	Khorasan_North	Jajarm	Female	23.03	5.24	40.14
**3611**	Exposure to secondhand smoke	Boushehr	Jam	Female	30.46	12.79	48.11
**3612**	Exposure to secondhand smoke	Kermanshah	Javanrud	Female	31.77	8.13	55.46
**3613**	Exposure to secondhand smoke	Kerman	Jiroft	Female	27.25	9.54	44.57
**3614**	Exposure to secondhand smoke	Khorasan_razavi	Joghatai	Female	24.14	0.00	51.04
**3615**	Exposure to secondhand smoke	Azararbayjan_East	Jolfa	Female	19.31	4.37	34.82
**3616**	Exposure to secondhand smoke	Khorasan_razavi	Jowayin	Female	25.45	0.00	49.96
**3617**	Exposure to secondhand smoke	Mazandaran	Juybar	Female	12.43	0.00	34.47
**3618**	Exposure to secondhand smoke	Hamedan	Kabudarahang	Female	42.71	18.97	66.38
**3619**	Exposure to secondhand smoke	Kerman	Kahnuj	Female	27.43	7.38	45.75
**3620**	Exposure to secondhand smoke	Golestan	Kalaleh	Female	19.15	0.25	38.05
**3621**	Exposure to secondhand smoke	Khorasan_razavi	Kalat	Female	25.12	0.00	54.57

**3622**	Exposure to secondhand smoke	Azararbayjan_East	Kaleibar	Female	22.65	0.00	50.79
**3623**	Exposure to secondhand smoke	Kordestan	Kamyaran	Female	37.43	12.33	62.23
**3624**	Exposure to secondhand smoke	Boushehr	Kangan	Female	34.22	10.05	58.84
**3625**	Exposure to secondhand smoke	Kermanshah	Kangavar	Female	31.39	12.62	50.91
**3626**	Exposure to secondhand smoke	Alborz	Karaj	Female	16.72	4.96	29.64
**3627**	Exposure to secondhand smoke	Khuzestan	Karun	Female	33.96	1.17	67.46
**3628**	Exposure to secondhand smoke	Isfahan	Kashan	Female	29.77	10.88	49.26
**3629**	Exposure to secondhand smoke	Khorasan_razavi	Kashmar	Female	21.91	3.63	39.61
**3630**	Exposure to secondhand smoke	Fars	Kavar	Female	42.42	18.30	65.19
**3631**	Exposure to secondhand smoke	Fars	Kazerun	Female	38.68	19.01	57.65
**3632**	Exposure to secondhand smoke	Mazandaran	Kelardasht	Female	36.43	2.59	71.11
**3633**	Exposure to secondhand smoke	Kerman	Kerman	Female	27.15	11.93	42.47
**3634**	Exposure to secondhand smoke	Kermanshah	Kermanshah	Female	23.12	9.14	37.07
**3635**	Exposure to secondhand smoke	Khorasan_razavi	Khaf	Female	39.26	18.91	60.99
**3636**	Exposure to secondhand smoke	Khorasan_razavi	Khalilabad	Female	26.07	0.83	51.38
**3637**	Exposure to secondhand smoke	Ardebil	Khalkhal	Female	38.15	11.54	66.41
**3638**	Exposure to secondhand smoke	Hormozgan	Khamir	Female	30.25	7.03	52.19
**3639**	Exposure to secondhand smoke	Isfahan	Khansar	Female	23.90	3.04	44.40
**3640**	Exposure to secondhand smoke	Sistan and Balouchestan	Khash	Female	30.40	8.73	51.60
**3641**	Exposure to secondhand smoke	Yazd	Khatam	Female	55.13	33.92	76.67
**3642**	Exposure to secondhand smoke	Fars	Kherameh	Female	43.06	21.55	63.92
**3643**	Exposure to secondhand smoke	Azararbayjan_East	Khodaafarin	Female	20.87	0.00	49.86
**3644**	Exposure to secondhand smoke	Zanjan	Khodabandeh	Female	52.41	32.57	74.16
**3645**	Exposure to secondhand smoke	Markazi	Khomeyn	Female	36.39	15.27	58.35
**3646**	Exposure to secondhand smoke	Isfahan	Khomeynishahr	Female	45.10	24.40	66.34
**3647**	Exposure to secondhand smoke	Markazi	Khondab	Female	62.18	38.57	82.62
**3648**	Exposure to secondhand smoke	Fars	Khonj	Female	47.99	26.51	69.47
**3649**	Exposure to secondhand smoke	Isfahan	Khoor va Biabanak	Female	22.41	0.00	48.26
**3650**	Exposure to secondhand smoke	Lorestan	Khorramabad	Female	29.29	9.98	47.01
**3651**	Exposure to secondhand smoke	Fars	Khorrambid	Female	48.48	24.59	71.77
**3652**	Exposure to secondhand smoke	Zanjan	Khorramdarreh	Female	31.73	8.46	54.78
**3653**	Exposure to secondhand smoke	Khuzestan	Khorramshahr	Female	33.76	8.87	59.32
**3654**	Exposure to secondhand smoke	Khorasan_razavi	Khoshab	Female	25.06	0.00	50.53
**3655**	Exposure to secondhand smoke	Azarbayjan_West	Khoy	Female	22.47	3.10	43.08
**3656**	Exposure to secondhand smoke	Khorasan_South	Khusef	Female	18.79	0.00	41.96
**3657**	Exposure to secondhand smoke	Chaharmahal	Kiaar	Female	37.75	13.99	61.47
**3658**	Exposure to secondhand smoke	Kohkiluye and Bouyer Ahmad	Kohgiluyeh	Female	42.98	21.72	63.79
**3659**	Exposure to secondhand smoke	Markazi	Komeijan	Female	34.12	10.94	57.52
**3660**	Exposure to secondhand smoke	Sistan and Balouchestan	Konarak	Female	41.01	14.83	67.31
**3661**	Exposure to secondhand smoke	Golestan	Kordkuy	Female	16.95	0.00	36.80
**3662**	Exposure to secondhand smoke	Ardebil	Kowsar	Female	37.43	7.97	67.95
**3663**	Exposure to secondhand smoke	Kerman	Kuhbonan	Female	23.93	0.00	51.06
**3664**	Exposure to secondhand smoke	Lorestan	Kuhdasht	Female	19.71	1.86	38.90
**3665**	Exposure to secondhand smoke	Chaharmahal	Kuhrang	Female	35.77	13.12	58.94
**3666**	Exposure to secondhand smoke	Gilan	Lahijan	Female	16.86	0.00	36.20
**3667**	Exposure to secondhand smoke	Khuzestan	Lali	Female	39.09	16.21	63.89
**3668**	Exposure to secondhand smoke	Fars	Lamard	Female	57.08	37.52	77.62
**3669**	Exposure to secondhand smoke	Kohkiluye and Bouyer Ahmad	Landeh	Female	41.89	0.57	81.08
**3670**	Exposure to secondhand smoke	Gilan	Langrud	Female	8.68	0.00	24.30
**3671**	Exposure to secondhand smoke	Isfahan	Lanjan	Female	26.72	8.12	44.84
**3672**	Exposure to secondhand smoke	Fars	Lar (Larestan)	Female	41.89	23.23	59.49
**3673**	Exposure to secondhand smoke	Chaharmahal	Lordakan	Female	41.78	21.71	62.49
**3674**	Exposure to secondhand smoke	Azarbayjan_West	Mahabad	Female	49.64	25.89	73.81
**3675**	Exposure to secondhand smoke	Markazi	Mahalat	Female	37.05	17.54	57.15
**3676**	Exposure to secondhand smoke	Mazandaran	Mahmudabad	Female	31.90	3.44	60.65
**3677**	Exposure to secondhand smoke	Zanjan	Mahneshan	Female	60.99	38.11	84.78
**3678**	Exposure to secondhand smoke	Khorasan_razavi	Mahvelat	Female	24.99	1.00	48.55
**3679**	Exposure to secondhand smoke	Azarbayjan_West	Maku	Female	31.77	1.88	60.73
**3680**	Exposure to secondhand smoke	Tehran	Malard	Female	25.20	5.45	45.78
**3681**	Exposure to secondhand smoke	Hamedan	Malayer	Female	54.02	30.07	78.39
**3682**	Exposure to secondhand smoke	Azararbayjan_East	Malekan	Female	41.00	13.28	70.76
**3683**	Exposure to secondhand smoke	Ilam	Malekshahi	Female	28.62	13.82	43.27
**3684**	Exposure to secondhand smoke	Fars	Mamasany	Female	48.63	29.75	67.86
**3685**	Exposure to secondhand smoke	Khorasan_North	Maneh and Samalqan	Female	23.70	5.47	41.75
**3686**	Exposure to secondhand smoke	Kerman	Manujan	Female	33.91	8.73	60.01
**3687**	Exposure to secondhand smoke	Azararbayjan_East	Maragheh	Female	31.23	12.28	50.00
**3688**	Exposure to secondhand smoke	Azararbayjan_East	Marand	Female	13.80	0.00	31.34
**3689**	Exposure to secondhand smoke	Golestan	Maravehtapeh	Female	31.73	12.91	50.33
**3690**	Exposure to secondhand smoke	Kordestan	Marivan	Female	37.15	14.25	59.69
**3691**	Exposure to secondhand smoke	Fars	Marvdasht	Female	41.21	22.49	58.92
**3692**	Exposure to secondhand smoke	Gilan	Masal	Female	22.47	0.72	44.84
**3693**	Exposure to secondhand smoke	Khorasan_razavi	Mashhad	Female	22.34	8.56	37.60
**3694**	Exposure to secondhand smoke	Khuzestan	Masjed Soleyman	Female	43.07	24.74	63.14
**3695**	Exposure to secondhand smoke	Semnan	Mayamey	Female	20.50	0.00	43.82
**3696**	Exposure to secondhand smoke	Semnan	Mehdishahr	Female	18.28	0.00	43.45
**3697**	Exposure to secondhand smoke	Ilam	Mehran	Female	13.77	0.00	32.97
**3698**	Exposure to secondhand smoke	Yazd	Mehriz	Female	33.70	15.54	52.28
**3699**	Exposure to secondhand smoke	Ardebil	Meshkinshahr	Female	32.21	7.32	56.27
**3700**	Exposure to secondhand smoke	Yazd	Meybod	Female	24.44	7.68	41.87
**3701**	Exposure to secondhand smoke	Hormozgan	Minab	Female	34.71	13.14	57.02
**3702**	Exposure to secondhand smoke	Golestan	Minudasht	Female	11.38	0.00	27.83
**3703**	Exposure to secondhand smoke	Sistan and Balouchestan	Mirjaveh	Female	29.90	0.00	65.26
**3704**	Exposure to secondhand smoke	Azarbayjan_West	Miyandoab	Female	44.82	24.50	65.39
**3705**	Exposure to secondhand smoke	Mazandaran	Miyandorud	Female	24.86	0.00	69.29
**3706**	Exposure to secondhand smoke	Azararbayjan_East	Miyaneh	Female	31.58	9.29	55.10
**3707**	Exposure to secondhand smoke	Isfahan	Mobarakeh	Female	31.10	9.84	51.84
**3708**	Exposure to secondhand smoke	Fars	Mohr	Female	46.35	24.36	69.85
**3709**	Exposure to secondhand smoke	Hamedan	Nahavand	Female	56.17	32.35	81.24
**3710**	Exposure to secondhand smoke	Isfahan	Najafabad	Female	31.48	13.09	49.70
**3711**	Exposure to secondhand smoke	Ardebil	Namin	Female	40.80	8.05	74.84
**3712**	Exposure to secondhand smoke	Azarbayjan_West	Naqadeh	Female	54.01	30.06	78.72
**3713**	Exposure to secondhand smoke	Kerman	Narmashir	Female	28.55	4.99	51.25
**3714**	Exposure to secondhand smoke	Isfahan	Natanz	Female	18.03	0.00	36.41
**3715**	Exposure to secondhand smoke	Isfahan	Nayin	Female	22.83	0.00	46.24
**3716**	Exposure to secondhand smoke	Alborz	Nazarabad	Female	22.79	6.15	39.17
**3717**	Exposure to secondhand smoke	Ardebil	Neer	Female	31.02	7.62	54.36
**3718**	Exposure to secondhand smoke	Khorasan_South	Nehbandan	Female	12.56	0.00	30.01
**3719**	Exposure to secondhand smoke	Mazandaran	Neka	Female	29.93	0.76	60.41

**3720**	Exposure to secondhand smoke	Fars	Neyriz	Female	45.68	25.84	65.82
**3721**	Exposure to secondhand smoke	Khorasan_razavi	Neyshabur	Female	24.23	6.85	40.82
**3722**	Exposure to secondhand smoke	Sistan and Balouchestan	Nikshahr	Female	37.46	17.34	58.45
**3723**	Exposure to secondhand smoke	Sistan and Balouchestan	Nimruz	Female	21.58	0.00	49.76
**3724**	Exposure to secondhand smoke	Mazandaran	Noshahr	Female	24.09	0.00	51.78
**3725**	Exposure to secondhand smoke	Mazandaran	Nur	Female	24.49	0.00	48.75
**3726**	Exposure to secondhand smoke	Khuzestan	Omidiyeh	Female	36.50	17.18	56.60
**3727**	Exposure to secondhand smoke	Azarbayjan_West	Orumiyeh	Female	42.66	23.62	62.07
**3728**	Exposure to secondhand smoke	Azarbayjan_West	Oshnaviyeh	Female	66.34	41.33	92.00
**3729**	Exposure to secondhand smoke	Azararbayjan_East	Osku	Female	24.76	1.05	48.18
**3730**	Exposure to secondhand smoke	Tehran	Pakdasht	Female	16.85	0.00	34.18
**3731**	Exposure to secondhand smoke	Tehran	Pardis	Female	15.60	0.00	42.96
**3732**	Exposure to secondhand smoke	Ardebil	Parsabad	Female	32.05	1.87	61.98
**3733**	Exposure to secondhand smoke	Hormozgan	Parsian (Gavbandi)	Female	29.19	9.52	48.01
**3734**	Exposure to secondhand smoke	Fars	Pasargad	Female	71.45	48.20	91.30
**3735**	Exposure to secondhand smoke	Kermanshah	Paveh	Female	31.42	6.96	55.79
**3736**	Exposure to secondhand smoke	Azarbayjan_West	Piranshahr	Female	55.72	29.00	84.57
**3737**	Exposure to secondhand smoke	Tehran	Pishva	Female	20.73	0.00	46.95
**3738**	Exposure to secondhand smoke	Azarbayjan_West	Poldasht	Female	30.91	6.80	54.63
**3739**	Exposure to secondhand smoke	Lorestan	Poldokhtar	Female	30.38	5.85	54.85
**3740**	Exposure to secondhand smoke	Mazandaran	Qaemshahr	Female	20.31	0.00	42.81
**3741**	Exposure to secondhand smoke	Tehran	Qarchak	Female	21.10	0.00	51.42
**3742**	Exposure to secondhand smoke	Sistan and Balouchestan	Qasr qand	Female	38.51	9.52	68.48
**3743**	Exposure to secondhand smoke	Kermanshah	Qasr-e-Shirin	Female	23.73	0.00	53.48
**3744**	Exposure to secondhand smoke	Khorasan_South	Qayenat	Female	21.89	3.56	40.49
**3745**	Exposure to secondhand smoke	Qazvin	Qazvin	Female	44.99	23.82	66.40
**3746**	Exposure to secondhand smoke	Hormozgan	Qeshm	Female	32.65	6.89	58.25
**3747**	Exposure to secondhand smoke	Fars	Qirokarzin	Female	45.57	25.63	66.23
**3748**	Exposure to secondhand smoke	Qom	Qom	Female	28.78	2.94	55.86
**3749**	Exposure to secondhand smoke	Kordestan	Qorveh	Female	21.84	2.32	43.92
**3750**	Exposure to secondhand smoke	Khorasan_razavi	Quchan	Female	40.26	18.27	64.24
**3751**	Exposure to secondhand smoke	Kerman	Rabar	Female	35.52	11.13	60.38
**3752**	Exposure to secondhand smoke	Kerman	Rafsanjan	Female	36.43	17.55	56.59
**3753**	Exposure to secondhand smoke	Khuzestan	Ramhormoz	Female	21.47	4.76	38.88
**3754**	Exposure to secondhand smoke	Mazandaran	Ramsar	Female	24.35	0.00	49.69
**3755**	Exposure to secondhand smoke	Khuzestan	Ramshir	Female	50.81	30.87	68.46
**3756**	Exposure to secondhand smoke	Golestan	Ramyan	Female	19.29	0.00	41.92
**3757**	Exposure to secondhand smoke	Gilan	Rasht	Female	10.95	0.00	25.15
**3758**	Exposure to secondhand smoke	Khorasan_razavi	Rashtkhar	Female	22.95	0.82	44.01
**3759**	Exposure to secondhand smoke	Kermanshah	Ravansar	Female	28.91	5.54	52.62
**3760**	Exposure to secondhand smoke	Kerman	Ravar	Female	20.21	6.11	36.10
**3761**	Exposure to secondhand smoke	Khorasan_North	Raz va Jergolan	Female	23.90	0.00	59.87
**3762**	Exposure to secondhand smoke	Hamedan	Razan	Female	48.88	24.79	74.41
**3763**	Exposure to secondhand smoke	Tehran	Rey	Female	26.72	7.73	48.02
**3764**	Exposure to secondhand smoke	Kerman	Reygan	Female	35.77	14.92	58.32
**3765**	Exposure to secondhand smoke	Gilan	Rezvanshahr	Female	21.01	0.00	43.81
**3766**	Exposure to secondhand smoke	Tehran	Robatkarim	Female	8.35	0.00	27.30
**3767**	Exposure to secondhand smoke	Fars	Rostam	Female	50.75	28.21	74.73
**3768**	Exposure to secondhand smoke	Kerman	Roudbar-e-Jonub	Female	33.29	12.13	55.46
**3769**	Exposure to secondhand smoke	Hormozgan	Rudan	Female	33.51	13.48	54.13
**3770**	Exposure to secondhand smoke	Gilan	Rudbar	Female	23.75	7.74	40.72
**3771**	Exposure to secondhand smoke	Gilan	Rudsar	Female	8.77	0.00	23.54
**3772**	Exposure to secondhand smoke	Lorestan	Rumshekan	Female	26.54	0.00	57.50
**3773**	Exposure to secondhand smoke	Khorasan_razavi	Sabzevar	Female	20.83	3.10	37.65
**3774**	Exposure to secondhand smoke	Yazd	Sadugh	Female	33.86	13.85	54.24
**3775**	Exposure to secondhand smoke	Kermanshah	Sahneh	Female	35.52	15.76	56.07
**3776**	Exposure to secondhand smoke	Kermanshah	Salas-e-Babajani	Female	24.06	4.33	43.40
**3777**	Exposure to secondhand smoke	Azarbayjan_West	Salmas	Female	34.94	12.61	57.68
**3778**	Exposure to secondhand smoke	Chaharmahal	Saman	Female	30.34	1.18	58.98
**3779**	Exposure to secondhand smoke	Kordestan	Sanandaj	Female	38.80	18.02	58.98
**3780**	Exposure to secondhand smoke	Kordestan	Saqqez	Female	56.36	33.85	80.21
**3781**	Exposure to secondhand smoke	Kermanshah	Sar-e-Pol-e-Zohab	Female	19.37	5.64	34.09
**3782**	Exposure to secondhand smoke	Azararbayjan_East	Sarab	Female	27.74	5.28	49.93
**3783**	Exposure to secondhand smoke	Khorasan_razavi	Sarakhs	Female	29.64	2.31	56.83
**3784**	Exposure to secondhand smoke	Sistan and Balouchestan	Saravan	Female	39.14	7.73	72.38
**3785**	Exposure to secondhand smoke	Khorasan_South	Sarayan	Female	18.41	0.00	40.41
**3786**	Exposure to secondhand smoke	Sistan and Balouchestan	Sarbaz	Female	39.14	16.75	61.91
**3787**	Exposure to secondhand smoke	Khorasan_South	Sarbisheh	Female	21.06	0.00	44.02
**3788**	Exposure to secondhand smoke	Azarbayjan_West	Sardasht	Female	52.75	26.86	80.05
**3789**	Exposure to secondhand smoke	Ardebil	Sarein	Female	39.24	11.73	67.59
**3790**	Exposure to secondhand smoke	Mazandaran	Sari	Female	22.22	1.48	43.03
**3791**	Exposure to secondhand smoke	Kordestan	Sarvabad	Female	41.95	12.67	72.56
**3792**	Exposure to secondhand smoke	Fars	Sarvestan	Female	44.39	20.26	68.93
**3793**	Exposure to secondhand smoke	Mazandaran	Savadkuh	Female	26.41	0.00	56.99
**3794**	Exposure to secondhand smoke	Mazandaran	Savadkuh_North	Female	24.31	0.00	60.14
**3795**	Exposure to secondhand smoke	Markazi	Saveh	Female	27.90	8.60	46.38
**3796**	Exposure to secondhand smoke	Alborz	Savojbolagh	Female	24.84	6.83	42.64
**3797**	Exposure to secondhand smoke	Lorestan	Selseleh	Female	37.10	11.28	63.64
**3798**	Exposure to secondhand smoke	Isfahan	Semirom	Female	39.78	18.68	61.08
**3799**	Exposure to secondhand smoke	Isfahan	Semirom-e-Sofla	Female	34.53	7.55	61.95
**3800**	Exposure to secondhand smoke	Semnan	Semnan	Female	14.59	0.00	32.01
**3801**	Exposure to secondhand smoke	Fars	Sepidan	Female	52.42	33.45	73.08
**3802**	Exposure to secondhand smoke	Azararbayjan_East	Shabestar	Female	9.17	0.00	28.34
**3803**	Exposure to secondhand smoke	Khuzestan	Shadegan	Female	33.23	11.38	55.47
**3804**	Exposure to secondhand smoke	Gilan	Shaft	Female	22.35	4.99	39.87
**3805**	Exposure to secondhand smoke	Azarbayjan_West	Shahindezh	Female	49.75	25.83	74.04
**3806**	Exposure to secondhand smoke	Tehran	Shahr-e Qods	Female	17.49	0.00	36.22
**3807**	Exposure to secondhand smoke	Kerman	Shahr-e-Babak	Female	34.27	12.65	55.35
**3808**	Exposure to secondhand smoke	Chaharmahal	Shahr-e-Kord	Female	27.76	9.79	45.09
**3809**	Exposure to secondhand smoke	Isfahan	Shahreza	Female	36.90	14.34	59.77
**3810**	Exposure to secondhand smoke	Tehran	Shahriyar	Female	20.64	4.42	36.74
**3811**	Exposure to secondhand smoke	Semnan	Shahrud	Female	19.85	4.63	35.17
**3812**	Exposure to secondhand smoke	Markazi	Shazand	Female	39.58	17.43	61.69
**3813**	Exposure to secondhand smoke	Tehran	Shemiranat	Female	7.42	0.00	24.90
**3814**	Exposure to secondhand smoke	Fars	Shiraz	Female	40.64	25.63	55.40
**3815**	Exposure to secondhand smoke	Khorasan_North	Shirvan	Female	18.01	0.00	36.44
**3816**	Exposure to secondhand smoke	Ilam	Shirvan and Chard-e-Aval	Female	29.90	8.86	51.79
**3817**	Exposure to secondhand smoke	Azarbayjan_West	Showt	Female	25.15	0.00	49.34

**3818**	Exposure to secondhand smoke	Khuzestan	Shush	Female	48.79	29.70	69.18
**3819**	Exposure to secondhand smoke	Khuzestan	Shushtar	Female	31.77	13.69	49.86
**3820**	Exposure to secondhand smoke	Gilan	Siahkal	Female	17.23	0.00	38.32
**3821**	Exposure to secondhand smoke	Sistan and Balouchestan	Sib o Soran	Female	39.20	10.96	69.10
**3822**	Exposure to secondhand smoke	Mazandaran	Simorgh	Female	23.15	0.00	57.94
**3823**	Exposure to secondhand smoke	Hormozgan	Sirik	Female	46.52	20.56	74.71
**3824**	Exposure to secondhand smoke	Kerman	Sirjan	Female	34.37	16.61	51.88
**3825**	Exposure to secondhand smoke	Ilam	Sirvan	Female	26.52	1.41	51.73
**3826**	Exposure to secondhand smoke	Zanjan	Soltaniyeh	Female	41.47	16.24	66.25
**3827**	Exposure to secondhand smoke	Kermanshah	Sonqor	Female	26.66	9.13	43.75
**3828**	Exposure to secondhand smoke	Semnan	Sorkheh	Female	19.42	0.00	43.69
**3829**	Exposure to secondhand smoke	Gilan	Sume'eh Sara	Female	13.76	0.00	29.92
**3830**	Exposure to secondhand smoke	Khorasan_South	Tabas	Female	16.61	0.95	31.99
**3831**	Exposure to secondhand smoke	Azararbayjan_East	Tabriz	Female	25.56	9.14	42.04
**3832**	Exposure to secondhand smoke	Markazi	Tafresh	Female	15.87	0.00	35.98
**3833**	Exposure to secondhand smoke	Yazd	Taft	Female	31.78	12.27	50.98
**3834**	Exposure to secondhand smoke	Azarbayjan_West	Takab	Female	50.41	26.68	74.88
**3835**	Exposure to secondhand smoke	Qazvin	Takestan	Female	43.93	12.33	74.73
**3836**	Exposure to secondhand smoke	Khorasan_razavi	Takht-e-Jolgeh (Firuzeh)	Female	20.75	0.00	42.11
**3837**	Exposure to secondhand smoke	Alborz	Taleghan	Female	33.85	18.22	49.96
**3838**	Exposure to secondhand smoke	Boushehr	Tangestan	Female	33.42	12.55	54.33
**3839**	Exposure to secondhand smoke	Zanjan	Tarom	Female	36.11	14.74	57.69
**3840**	Exposure to secondhand smoke	Gilan	Tavalesh	Female	25.60	5.50	46.39
**3841**	Exposure to secondhand smoke	Khorasan_razavi	Taybad	Female	53.36	29.19	77.41
**3842**	Exposure to secondhand smoke	Tehran	Tehran	Female	16.59	2.60	30.83
**3843**	Exposure to secondhand smoke	Isfahan	Tiran and Karvan	Female	22.71	3.52	42.10
**3844**	Exposure to secondhand smoke	Mazandaran	Tonekabon	Female	27.49	3.18	50.74
**3845**	Exposure to secondhand smoke	Khorasan_razavi	Torbat-e-Heydariyeh	Female	25.48	6.95	44.14
**3846**	Exposure to secondhand smoke	Khorasan_razavi	Torbat-e-Jam	Female	36.09	16.68	56.66
**3847**	Exposure to secondhand smoke	Hamedan	Tuyserkan	Female	44.00	18.54	69.34
**3848**	Exposure to secondhand smoke	Tehran	Varamin	Female	23.88	5.67	42.76
**3849**	Exposure to secondhand smoke	Azararbayjan_East	Varzaqan	Female	15.69	0.00	33.80
**3850**	Exposure to secondhand smoke	Yazd	Yazd	Female	26.89	11.31	42.22
**3851**	Exposure to secondhand smoke	Sistan and Balouchestan	Zabol	Female	16.78	0.00	38.48
**3852**	Exposure to secondhand smoke	Sistan and Balouchestan	Zaboli (Mehrestan)	Female	35.53	6.56	64.54
**3853**	Exposure to secondhand smoke	Sistan and Balouchestan	Zahedan	Female	24.76	7.79	41.41
**3854**	Exposure to secondhand smoke	Zanjan	Zanjan	Female	40.34	22.89	58.22
**3855**	Exposure to secondhand smoke	Kerman	Zarand	Female	25.83	3.45	48.11
**3856**	Exposure to secondhand smoke	Markazi	Zarandiyeh	Female	27.66	12.59	42.80
**3857**	Exposure to secondhand smoke	Fars	Zarrindasht	Female	35.09	15.41	54.44
**3858**	Exposure to secondhand smoke	Khorasan_razavi	Zave	Female	23.25	5.89	41.00
**3859**	Exposure to secondhand smoke	Sistan and Balouchestan	Zehak	Female	20.52	0.00	53.80
**3860**	Exposure to secondhand smoke	Khorasan_South	Zir kuh	Female	21.44	0.00	47.07
**3861**	Exposure to secondhand smoke	Khuzestan	Abadan	Male	31.83	7.32	56.47
**3862**	Exposure to secondhand smoke	Fars	Abadeh	Male	58.12	36.87	81.82
**3863**	Exposure to secondhand smoke	Yazd	Abarkuh	Male	39.47	18.37	60.65
**3864**	Exposure to secondhand smoke	Mazandaran	Abbas abad	Male	53.44	32.49	72.00
**3865**	Exposure to secondhand smoke	Ilam	Abdanan	Male	27.08	5.50	51.32
**3866**	Exposure to secondhand smoke	Zanjan	Abhar	Male	45.41	21.29	68.06
**3867**	Exposure to secondhand smoke	Hormozgan	Abumusa	Male	28.75	0.00	61.83
**3868**	Exposure to secondhand smoke	Qazvin	Abyek	Male	61.85	37.42	86.50
**3869**	Exposure to secondhand smoke	Azararbayjan_East	Ahar	Male	23.58	0.00	46.20
**3870**	Exposure to secondhand smoke	Khuzestan	Ahvaz	Male	42.60	24.86	59.83
**3871**	Exposure to secondhand smoke	Azararbayjan_East	Ajabshir	Male	28.71	11.07	49.10
**3872**	Exposure to secondhand smoke	Qazvin	Alborz	Male	46.14	18.10	74.34
**3873**	Exposure to secondhand smoke	Golestan	Aliabad	Male	33.93	8.19	60.88
**3874**	Exposure to secondhand smoke	Lorestan	Aligudarz	Male	38.18	14.90	59.82
**3875**	Exposure to secondhand smoke	Gilan	Amlash	Male	23.78	0.00	50.15
**3876**	Exposure to secondhand smoke	Mazandaran	Amol	Male	35.68	14.73	57.86
**3877**	Exposure to secondhand smoke	Kerman	Anar	Male	45.02	10.73	80.60
**3878**	Exposure to secondhand smoke	Kerman	Anbarabad	Male	42.89	16.01	69.07
**3879**	Exposure to secondhand smoke	Khuzestan	Andika	Male	56.00	32.41	81.28
**3880**	Exposure to secondhand smoke	Khuzestan	Andimeshk	Male	39.11	13.54	62.64
**3881**	Exposure to secondhand smoke	Golestan	Aq Qala	Male	26.82	3.53	49.48
**3882**	Exposure to secondhand smoke	Khuzestan	Aqajari	Male	49.30	17.48	81.92
**3883**	Exposure to secondhand smoke	Semnan	Aradan	Male	29.20	0.97	57.13
**3884**	Exposure to secondhand smoke	Markazi	Arak	Male	56.93	32.55	81.95
**3885**	Exposure to secondhand smoke	Isfahan	Aran and Bidgol	Male	34.41	8.66	58.92
**3886**	Exposure to secondhand smoke	Ardebil	Ardabil	Male	66.31	41.39	91.78
**3887**	Exposure to secondhand smoke	Yazd	Ardakan	Male	40.54	21.79	61.75
**3888**	Exposure to secondhand smoke	Chaharmahal	Ardal	Male	49.16	24.67	74.78
**3889**	Exposure to secondhand smoke	Isfahan	Ardestan	Male	42.89	17.75	69.37
**3890**	Exposure to secondhand smoke	Fars	Arsanjan	Male	47.79	22.20	73.10
**3891**	Exposure to secondhand smoke	Kerman	Arzouyeh	Male	48.14	30.25	65.06
**3892**	Exposure to secondhand smoke	Hamedan	Asadabad	Male	46.39	17.90	73.35
**3893**	Exposure to secondhand smoke	Boushehr	Asaluyeh	Male	39.61	9.67	69.13
**3894**	Exposure to secondhand smoke	Markazi	Ashtiyan	Male	47.36	12.38	83.29
**3895**	Exposure to secondhand smoke	Gilan	Astaneh-ye-Ashrafiyeh	Male	28.08	2.05	54.57
**3896**	Exposure to secondhand smoke	Gilan	Astara	Male	42.05	12.26	74.01
**3897**	Exposure to secondhand smoke	Qazvin	Avaj	Male	52.03	19.17	83.93
**3898**	Exposure to secondhand smoke	Golestan	Azadshahr	Male	29.74	2.42	56.65
**3899**	Exposure to secondhand smoke	Azararbayjan_East	Azarshahr	Male	31.86	0.00	63.12
**3900**	Exposure to secondhand smoke	Lorestan	Azna	Male	26.29	5.53	48.47
**3901**	Exposure to secondhand smoke	Mazandaran	Babol	Male	32.63	11.84	53.70
**3902**	Exposure to secondhand smoke	Mazandaran	Babolsar	Male	36.10	10.85	62.62
**3903**	Exposure to secondhand smoke	Ilam	Badreh	Male	45.49	15.36	75.29
**3904**	Exposure to secondhand smoke	Yazd	Bafq	Male	31.37	8.62	52.69
**3905**	Exposure to secondhand smoke	Kerman	Baft	Male	58.48	34.42	84.77
**3906**	Exposure to secondhand smoke	Khuzestan	Baghemalek	Male	42.31	18.62	64.00
**3907**	Exposure to secondhand smoke	Yazd	Bahabad	Male	32.51	5.67	58.12
**3908**	Exposure to secondhand smoke	Hamedan	Bahar	Male	59.78	36.25	84.01
**3909**	Exposure to secondhand smoke	Tehran	Baharestan (Golestan)	Male	31.97	11.10	53.33
**3910**	Exposure to secondhand smoke	Kohkiluye and Bouyer Ahmad	Bahmani	Male	57.99	38.08	78.00
**3911**	Exposure to secondhand smoke	Khorasan_razavi	Bajestan	Male	29.86	2.12	57.11
**3912**	Exposure to secondhand smoke	Khorasan_razavi	Bakhraz	Male	38.47	13.75	62.50
**3913**	Exposure to secondhand smoke	Kerman	Bam	Male	42.18	20.15	64.09
**3914**	Exposure to secondhand smoke	Hormozgan	Bandar-e-Abbas	Male	25.74	8.31	43.38
**3915**	Exposure to secondhand smoke	Gilan	Bandar-e-Anzali	Male	24.95	2.18	47.05

**3916**	Exposure to secondhand smoke	Golestan	Bandar-e-Gaz	Male	34.68	15.83	52.52
**3917**	Exposure to secondhand smoke	Hormozgan	Bandar-e-Jask	Male	36.14	10.20	62.00
**3918**	Exposure to secondhand smoke	Hormozgan	Bandar-e-Lengeh	Male	19.51	0.53	39.47
**3919**	Exposure to secondhand smoke	Khuzestan	Bandar-e-Mahshahr	Male	40.24	20.37	59.35
**3920**	Exposure to secondhand smoke	Golestan	Bandar-e-Torkaman	Male	31.39	4.26	57.86
**3921**	Exposure to secondhand smoke	Kordestan	Baneh	Male	57.87	30.49	86.51
**3922**	Exposure to secondhand smoke	Khorasan_razavi	Bardeskan	Male	29.40	5.35	52.78
**3923**	Exposure to secondhand smoke	Kerman	Bardsir	Male	52.63	28.29	79.01
**3924**	Exposure to secondhand smoke	Hormozgan	Bashagerd	Male	44.29	25.38	62.96
**3925**	Exposure to secondhand smoke	Kohkiluye and Bouyer Ahmad	Basht	Male	75.62	49.21	100.00
**3926**	Exposure to secondhand smoke	Hormozgan	Bastak	Male	31.80	5.76	58.74
**3927**	Exposure to secondhand smoke	Khuzestan	Bavi	Male	42.57	17.90	66.66
**3928**	Exposure to secondhand smoke	Khuzestan	Behbahan	Male	50.12	25.64	73.36
**3929**	Exposure to secondhand smoke	Mazandaran	Behshahr	Male	31.31	0.00	64.08
**3930**	Exposure to secondhand smoke	Kordestan	Bijar	Male	57.19	38.02	77.98
**3931**	Exposure to secondhand smoke	Ardebil	Bilehsavar	Male	40.56	0.00	87.01
**3932**	Exposure to secondhand smoke	Khorasan_razavi	Binaloud	Male	41.99	20.10	63.51
**3933**	Exposure to secondhand smoke	Khorasan_South	Birjand	Male	30.58	10.06	51.02
**3934**	Exposure to secondhand smoke	Khorasan_North	Bojnurd	Male	28.69	9.41	48.10
**3935**	Exposure to secondhand smoke	Chaharmahal	Bon	Male	48.04	15.22	80.54
**3936**	Exposure to secondhand smoke	Azararbayjan_East	Bonab	Male	38.94	12.48	63.91
**3937**	Exposure to secondhand smoke	Isfahan	Borkhar	Male	50.39	28.42	73.65
**3938**	Exposure to secondhand smoke	Isfahan	Borkhar and Meymeh	Male	45.76	23.71	69.25
**3939**	Exposure to secondhand smoke	Chaharmahal	Borujen	Male	39.94	19.78	59.30
**3940**	Exposure to secondhand smoke	Lorestan	Borujerd	Male	43.06	20.87	64.94
**3941**	Exposure to secondhand smoke	Khorasan_South	Boshruyeh	Male	26.65	0.00	55.82
**3942**	Exposure to secondhand smoke	Azararbayjan_East	Bostanabad	Male	39.70	13.68	66.50
**3943**	Exposure to secondhand smoke	Fars	Bovanat	Male	45.12	20.96	67.44
**3944**	Exposure to secondhand smoke	Kohkiluye and Bouyer Ahmad	Boyer Ahmad	Male	58.28	36.49	80.17
**3945**	Exposure to secondhand smoke	Qazvin	Boyinzahra	Male	59.29	36.34	83.45
**3946**	Exposure to secondhand smoke	Isfahan	Buein va Miandasht	Male	47.80	19.84	76.46
**3947**	Exposure to secondhand smoke	Azarbayjan_West	Bukan	Male	55.47	31.02	79.94
**3948**	Exposure to secondhand smoke	Boushehr	Bushehr	Male	38.05	14.18	62.23
**3949**	Exposure to secondhand smoke	Isfahan	Chadegan	Male	60.57	39.59	82.97
**3950**	Exposure to secondhand smoke	Sistan and Balouchestan	Chahbahar	Male	35.33	12.80	57.35
**3951**	Exposure to secondhand smoke	Azarbayjan_West	Chaipareh	Male	26.74	0.00	57.85
**3952**	Exposure to secondhand smoke	Azarbayjan_West	Chaldoran	Male	25.13	0.00	57.49
**3953**	Exposure to secondhand smoke	Mazandaran	Chalus	Male	38.60	12.93	63.43
**3954**	Exposure to secondhand smoke	Azararbayjan_East	Charoimaq	Male	44.61	15.88	74.33
**3955**	Exposure to secondhand smoke	Khorasan_razavi	Chenaran	Male	52.96	29.66	77.43
**3956**	Exposure to secondhand smoke	Kohkiluye and Bouyer Ahmad	Cheram	Male	56.84	26.05	86.28
**3957**	Exposure to secondhand smoke	Kermanshah	Dalaho	Male	37.47	12.10	62.35
**3958**	Exposure to secondhand smoke	Lorestan	Dalfan	Male	50.64	29.36	73.96
**3959**	Exposure to secondhand smoke	Sistan and Balouchestan	Dalgan	Male	42.33	24.61	59.62
**3960**	Exposure to secondhand smoke	Tehran	Damavand	Male	30.41	7.78	53.19
**3961**	Exposure to secondhand smoke	Semnan	Damghan	Male	28.28	6.43	49.30
**3962**	Exposure to secondhand smoke	Fars	Darab	Male	59.60	39.09	82.14
**3963**	Exposure to secondhand smoke	Khorasan_South	Darmian	Male	30.67	7.30	54.47
**3964**	Exposure to secondhand smoke	Khorasan_razavi	Darrehgaz	Male	23.06	1.29	45.40
**3965**	Exposure to secondhand smoke	Ilam	Darrehshahr	Male	37.25	11.13	62.85
**3966**	Exposure to secondhand smoke	Khuzestan	Dasht-e-Azadegan	Male	46.71	19.64	74.83
**3967**	Exposure to secondhand smoke	Boushehr	Dashtestan	Male	45.24	22.15	68.34
**3968**	Exposure to secondhand smoke	Boushehr	Dashti	Male	43.80	20.66	68.42
**3969**	Exposure to secondhand smoke	Khorasan_razavi	Davarzan	Male	30.85	1.85	59.60
**3970**	Exposure to secondhand smoke	Boushehr	Dayyer	Male	59.36	35.69	78.52
**3971**	Exposure to secondhand smoke	Kordestan	Dehgolan	Male	60.62	38.65	85.61
**3972**	Exposure to secondhand smoke	Ilam	Dehloran	Male	47.02	22.77	71.50
**3973**	Exposure to secondhand smoke	Markazi	Delijan	Male	50.42	21.77	79.82
**3974**	Exposure to secondhand smoke	Kohkiluye and Bouyer Ahmad	Dena	Male	56.84	27.71	87.24
**3975**	Exposure to secondhand smoke	Boushehr	Deylam	Male	48.22	18.22	78.83
**3976**	Exposure to secondhand smoke	Khuzestan	Dezful	Male	45.92	25.80	65.64
**3977**	Exposure to secondhand smoke	Kordestan	Divandarreh	Male	62.57	38.67	89.18
**3978**	Exposure to secondhand smoke	Lorestan	Dorud	Male	49.29	26.58	73.74
**3979**	Exposure to secondhand smoke	Lorestan	Doureh	Male	40.08	19.06	60.42
**3980**	Exposure to secondhand smoke	Fars	Eqlid	Male	50.77	26.74	75.52
**3981**	Exposure to secondhand smoke	Khorasan_North	Esfarayen	Male	35.97	15.16	57.27
**3982**	Exposure to secondhand smoke	Alborz	Eshtehard	Male	37.15	10.60	63.25
**3983**	Exposure to secondhand smoke	Kermanshah	Eslamabad-e-Gharb	Male	35.18	11.91	57.30
**3984**	Exposure to secondhand smoke	Tehran	Eslamshahr	Male	33.86	12.56	55.83
**3985**	Exposure to secondhand smoke	Fars	Estahban	Male	43.53	18.68	67.00
**3986**	Exposure to secondhand smoke	Ilam	Eyvan	Male	70.96	43.59	97.26
**3987**	Exposure to secondhand smoke	Kerman	Fahraj	Male	36.84	10.07	62.49
**3988**	Exposure to secondhand smoke	Isfahan	Falavarjan	Male	62.21	40.69	84.42
**3989**	Exposure to secondhand smoke	Hamedan	Famenin	Male	24.48	1.02	51.10
**3990**	Exposure to secondhand smoke	Markazi	Farahan	Male	47.88	10.41	83.36
**3991**	Exposure to secondhand smoke	Fars	Farashband	Male	47.30	21.33	73.80
**3992**	Exposure to secondhand smoke	Alborz	Fardis	Male	33.09	3.88	61.27
**3993**	Exposure to secondhand smoke	Isfahan	Faridan	Male	55.11	33.44	78.48
**3994**	Exposure to secondhand smoke	Khorasan_razavi	Fariman	Male	37.94	14.45	61.79
**3995**	Exposure to secondhand smoke	Khorasan_North	Faroj	Male	41.00	19.53	63.59
**3996**	Exposure to secondhand smoke	Chaharmahal	Farsan	Male	52.33	30.83	74.28
**3997**	Exposure to secondhand smoke	Kerman	Faryab	Male	43.06	12.55	73.29
**3998**	Exposure to secondhand smoke	Fars	Fasa	Male	61.97	40.95	83.30
**3999**	Exposure to secondhand smoke	Khorasan_South	Ferdows	Male	15.52	0.00	36.39
**4000**	Exposure to secondhand smoke	Mazandaran	Fereydunkenar	Male	38.84	17.27	60.15
**4001**	Exposure to secondhand smoke	Isfahan	Fereydunshahr	Male	50.30	23.01	77.92
**4002**	Exposure to secondhand smoke	Fars	Firozabad	Male	63.19	41.36	86.38
**4003**	Exposure to secondhand smoke	Tehran	Firuzkuh	Male	20.75	0.00	40.48
**4004**	Exposure to secondhand smoke	Sistan and Balouchestan	Fonuj	Male	38.55	11.43	67.74
**4005**	Exposure to secondhand smoke	Gilan	Fuman	Male	25.76	4.89	45.35
**4006**	Exposure to secondhand smoke	Kohkiluye and Bouyer Ahmad	Gachsaran	Male	71.01	47.85	95.93
**4007**	Exposure to secondhand smoke	Golestan	Galikesh	Male	30.84	3.66	58.44
**4008**	Exposure to secondhand smoke	Mazandaran	Galugah	Male	32.40	0.00	66.67
**4009**	Exposure to secondhand smoke	Semnan	Garmsar	Male	26.47	6.77	45.91
**4010**	Exposure to secondhand smoke	Boushehr	Genaveh	Male	46.98	21.30	72.87
**4011**	Exposure to secondhand smoke	Fars	Gerash	Male	50.97	18.30	84.36
**4012**	Exposure to secondhand smoke	Khorasan_North	Germeh	Male	29.82	0.00	60.66
**4013**	Exposure to secondhand smoke	Ardebil	Germi	Male	43.64	10.54	76.37

**4014**	Exposure to secondhand smoke	Kerman	Ghaleye-Ganj	Male	43.85	18.82	67.97
**4015**	Exposure to secondhand smoke	Kermanshah	Gilan-e-Gharb	Male	36.67	14.95	57.53
**4016**	Exposure to secondhand smoke	Isfahan	Golpayegan	Male	49.18	25.06	73.57
**4017**	Exposure to secondhand smoke	Golestan	Gomishan	Male	27.33	0.00	57.88
**4018**	Exposure to secondhand smoke	Khorasan_razavi	Gonabad	Male	22.40	5.96	40.96
**4019**	Exposure to secondhand smoke	Golestan	Gonbad-e-Kavus	Male	30.54	9.26	51.80
**4020**	Exposure to secondhand smoke	Golestan	Gorgan	Male	29.99	9.19	50.35
**4021**	Exposure to secondhand smoke	Khuzestan	Guotvand	Male	46.71	23.50	68.93
**4022**	Exposure to secondhand smoke	Khuzestan	Haftgol	Male	45.21	18.57	71.59
**4023**	Exposure to secondhand smoke	Hormozgan	Hajiabad	Male	37.66	16.65	60.26
**4024**	Exposure to secondhand smoke	Hamedan	Hamadan	Male	47.92	27.06	69.12
**4025**	Exposure to secondhand smoke	Khuzestan	Hamidiyeh	Male	46.81	15.36	77.54
**4026**	Exposure to secondhand smoke	Sistan and Balouchestan	Hamoon	Male	21.24	0.00	49.63
**4027**	Exposure to secondhand smoke	Azararbayjan_East	Haris	Male	30.62	6.36	54.37
**4028**	Exposure to secondhand smoke	Kermanshah	Harsin	Male	51.34	30.54	73.09
**4029**	Exposure to secondhand smoke	Azararbayjan_East	Hashtrud	Male	23.30	1.22	47.07
**4030**	Exposure to secondhand smoke	Khuzestan	Hendijan	Male	48.33	18.62	78.42
**4031**	Exposure to secondhand smoke	Sistan and Balouchestan	Hirmand	Male	8.96	0.00	29.42
**4032**	Exposure to secondhand smoke	Khuzestan	Hoveizeh	Male	46.52	13.10	80.20
**4033**	Exposure to secondhand smoke	Zanjan	Ijerod	Male	57.14	31.40	83.27
**4034**	Exposure to secondhand smoke	Ilam	Ilam	Male	54.03	28.55	80.19
**4035**	Exposure to secondhand smoke	Sistan and Balouchestan	Iranshahr	Male	43.77	24.63	64.96
**4036**	Exposure to secondhand smoke	Isfahan	Isfahan	Male	39.51	22.29	56.59
**4037**	Exposure to secondhand smoke	Khuzestan	Izeh	Male	50.81	28.26	74.23
**4038**	Exposure to secondhand smoke	Fars	Jahrom	Male	56.17	35.42	78.17
**4039**	Exposure to secondhand smoke	Khorasan_North	Jajarm	Male	28.62	4.87	51.31
**4040**	Exposure to secondhand smoke	Boushehr	Jam	Male	22.30	0.73	47.59
**4041**	Exposure to secondhand smoke	Kermanshah	Javanrud	Male	47.29	25.11	70.66
**4042**	Exposure to secondhand smoke	Kerman	Jiroft	Male	38.96	15.82	60.92
**4043**	Exposure to secondhand smoke	Khorasan_razavi	Joghatai	Male	31.49	1.72	61.18
**4044**	Exposure to secondhand smoke	Azararbayjan_East	Jolfa	Male	15.81	0.00	38.68
**4045**	Exposure to secondhand smoke	Khorasan_razavi	Jowayin	Male	32.53	6.08	59.90
**4046**	Exposure to secondhand smoke	Mazandaran	Juybar	Male	20.76	0.00	43.30
**4047**	Exposure to secondhand smoke	Hamedan	Kabudarahang	Male	55.22	30.70	82.46
**4048**	Exposure to secondhand smoke	Kerman	Kahnuj	Male	40.08	14.04	65.20
**4049**	Exposure to secondhand smoke	Golestan	Kalaleh	Male	34.44	10.16	57.79
**4050**	Exposure to secondhand smoke	Khorasan_razavi	Kalat	Male	36.59	5.07	69.05
**4051**	Exposure to secondhand smoke	Azararbayjan_East	Kaleibar	Male	26.69	0.00	59.93
**4052**	Exposure to secondhand smoke	Kordestan	Kamyaran	Male	49.67	30.76	68.24
**4053**	Exposure to secondhand smoke	Boushehr	Kangan	Male	39.03	9.94	66.81
**4054**	Exposure to secondhand smoke	Kermanshah	Kangavar	Male	36.43	17.80	54.65
**4055**	Exposure to secondhand smoke	Alborz	Karaj	Male	30.45	14.98	46.60
**4056**	Exposure to secondhand smoke	Khuzestan	Karun	Male	43.20	7.92	79.13
**4057**	Exposure to secondhand smoke	Isfahan	Kashan	Male	30.28	7.91	52.01
**4058**	Exposure to secondhand smoke	Khorasan_razavi	Kashmar	Male	30.81	7.41	54.62
**4059**	Exposure to secondhand smoke	Fars	Kavar	Male	58.49	34.34	84.49
**4060**	Exposure to secondhand smoke	Fars	Kazerun	Male	32.79	9.39	55.73
**4061**	Exposure to secondhand smoke	Mazandaran	Kelardasht	Male	42.66	11.73	74.91
**4062**	Exposure to secondhand smoke	Kerman	Kerman	Male	38.48	19.20	58.10
**4063**	Exposure to secondhand smoke	Kermanshah	Kermanshah	Male	31.92	15.69	47.73
**4064**	Exposure to secondhand smoke	Khorasan_razavi	Khaf	Male	43.91	22.90	68.28
**4065**	Exposure to secondhand smoke	Khorasan_razavi	Khalilabad	Male	32.39	5.47	59.81
**4066**	Exposure to secondhand smoke	Ardebil	Khalkhal	Male	61.86	40.40	82.33
**4067**	Exposure to secondhand smoke	Hormozgan	Khamir	Male	30.98	8.49	53.32
**4068**	Exposure to secondhand smoke	Isfahan	Khansar	Male	36.84	12.18	60.20
**4069**	Exposure to secondhand smoke	Sistan and Balouchestan	Khash	Male	37.94	13.91	61.55
**4070**	Exposure to secondhand smoke	Yazd	Khatam	Male	51.11	33.70	66.15
**4071**	Exposure to secondhand smoke	Fars	Kherameh	Male	36.04	14.97	56.48
**4072**	Exposure to secondhand smoke	Azararbayjan_East	Khodaafarin	Male	24.03	0.00	57.42
**4073**	Exposure to secondhand smoke	Zanjan	Khodabandeh	Male	63.65	42.05	86.75
**4074**	Exposure to secondhand smoke	Markazi	Khomeyn	Male	59.42	29.41	92.19
**4075**	Exposure to secondhand smoke	Isfahan	Khomeynishahr	Male	53.71	31.01	77.10
**4076**	Exposure to secondhand smoke	Markazi	Khondab	Male	62.41	32.76	94.45
**4077**	Exposure to secondhand smoke	Fars	Khonj	Male	56.43	33.81	80.69
**4078**	Exposure to secondhand smoke	Isfahan	Khoor va Biabanak	Male	36.93	8.03	66.52
**4079**	Exposure to secondhand smoke	Lorestan	Khorramabad	Male	37.51	17.06	56.73
**4080**	Exposure to secondhand smoke	Fars	Khorrambid	Male	50.92	25.64	76.99
**4081**	Exposure to secondhand smoke	Zanjan	Khorramdarreh	Male	58.37	33.99	83.53
**4082**	Exposure to secondhand smoke	Khuzestan	Khorramshahr	Male	55.13	31.52	78.94
**4083**	Exposure to secondhand smoke	Khorasan_razavi	Khoshab	Male	32.34	4.91	58.40
**4084**	Exposure to secondhand smoke	Azarbayjan_West	Khoy	Male	26.91	3.97	48.64
**4085**	Exposure to secondhand smoke	Khorasan_South	Khusef	Male	30.63	3.91	58.67
**4086**	Exposure to secondhand smoke	Chaharmahal	Kiaar	Male	48.42	25.09	72.21
**4087**	Exposure to secondhand smoke	Kohkiluye and Bouyer Ahmad	Kohgiluyeh	Male	49.83	24.62	72.32
**4088**	Exposure to secondhand smoke	Markazi	Komeijan	Male	49.51	14.72	83.12
**4089**	Exposure to secondhand smoke	Sistan and Balouchestan	Konarak	Male	39.54	16.80	62.67
**4090**	Exposure to secondhand smoke	Golestan	Kordkuy	Male	32.13	7.33	57.08
**4091**	Exposure to secondhand smoke	Ardebil	Kowsar	Male	58.27	24.49	93.22
**4092**	Exposure to secondhand smoke	Kerman	Kuhbonan	Male	36.56	5.81	67.17
**4093**	Exposure to secondhand smoke	Lorestan	Kuhdasht	Male	35.74	13.37	56.82
**4094**	Exposure to secondhand smoke	Chaharmahal	Kuhrang	Male	52.91	30.59	77.55
**4095**	Exposure to secondhand smoke	Gilan	Lahijan	Male	29.87	5.94	54.73
**4096**	Exposure to secondhand smoke	Khuzestan	Lali	Male	53.64	27.27	80.90
**4097**	Exposure to secondhand smoke	Fars	Lamard	Male	62.34	39.36	87.17
**4098**	Exposure to secondhand smoke	Kohkiluye and Bouyer Ahmad	Landeh	Male	56.11	16.59	95.76
**4099**	Exposure to secondhand smoke	Gilan	Langrud	Male	14.75	0.00	34.19
**4100**	Exposure to secondhand smoke	Isfahan	Lanjan	Male	42.42	19.93	62.99
**4101**	Exposure to secondhand smoke	Fars	Lar (Larestan)	Male	51.06	30.45	72.77
**4102**	Exposure to secondhand smoke	Chaharmahal	Lordakan	Male	51.15	31.95	71.54
**4103**	Exposure to secondhand smoke	Azarbayjan_West	Mahabad	Male	50.50	27.62	72.97
**4104**	Exposure to secondhand smoke	Markazi	Mahalat	Male	51.34	18.28	82.93
**4105**	Exposure to secondhand smoke	Mazandaran	Mahmudabad	Male	32.44	6.23	58.33
**4106**	Exposure to secondhand smoke	Zanjan	Mahneshan	Male	64.36	40.26	89.68
**4107**	Exposure to secondhand smoke	Khorasan_razavi	Mahvelat	Male	31.57	2.79	59.47
**4108**	Exposure to secondhand smoke	Azarbayjan_West	Maku	Male	11.36	0.00	35.85
**4109**	Exposure to secondhand smoke	Tehran	Malard	Male	34.59	13.54	55.79
**4110**	Exposure to secondhand smoke	Hamedan	Malayer	Male	55.04	31.29	80.35
**4111**	Exposure to secondhand smoke	Azararbayjan_East	Malekan	Male	57.50	30.25	86.90

**4112**	Exposure to secondhand smoke	Ilam	Malekshahi	Male	47.23	13.05	82.50
**4113**	Exposure to secondhand smoke	Fars	Mamasany	Male	50.35	28.60	71.83
**4114**	Exposure to secondhand smoke	Khorasan_North	Maneh and Samalqan	Male	27.54	5.79	49.34
**4115**	Exposure to secondhand smoke	Kerman	Manujan	Male	44.27	14.73	74.59
**4116**	Exposure to secondhand smoke	Azararbayjan_East	Maragheh	Male	46.93	24.36	71.86
**4117**	Exposure to secondhand smoke	Azararbayjan_East	Marand	Male	14.65	0.00	35.16
**4118**	Exposure to secondhand smoke	Golestan	Maravehtapeh	Male	31.05	1.69	61.05
**4119**	Exposure to secondhand smoke	Kordestan	Marivan	Male	56.94	31.08	83.75
**4120**	Exposure to secondhand smoke	Fars	Marvdasht	Male	34.01	10.53	56.08
**4121**	Exposure to secondhand smoke	Gilan	Masal	Male	32.00	7.51	56.40
**4122**	Exposure to secondhand smoke	Khorasan_razavi	Mashhad	Male	32.80	16.60	49.57
**4123**	Exposure to secondhand smoke	Khuzestan	Masjed Soleyman	Male	60.41	38.99	83.24
**4124**	Exposure to secondhand smoke	Semnan	Mayamey	Male	26.72	1.95	53.09
**4125**	Exposure to secondhand smoke	Semnan	Mehdishahr	Male	25.94	5.31	46.14
**4126**	Exposure to secondhand smoke	Ilam	Mehran	Male	43.81	12.17	74.74
**4127**	Exposure to secondhand smoke	Yazd	Mehriz	Male	40.33	23.89	57.43
**4128**	Exposure to secondhand smoke	Ardebil	Meshkinshahr	Male	48.08	19.57	75.90
**4129**	Exposure to secondhand smoke	Yazd	Meybod	Male	40.92	18.58	63.16
**4130**	Exposure to secondhand smoke	Hormozgan	Minab	Male	30.07	9.48	49.51
**4131**	Exposure to secondhand smoke	Golestan	Minudasht	Male	26.43	1.33	49.36
**4132**	Exposure to secondhand smoke	Sistan and Balouchestan	Mirjaveh	Male	34.74	1.36	67.10
**4133**	Exposure to secondhand smoke	Azarbayjan_West	Miyandoab	Male	59.43	38.61	81.13
**4134**	Exposure to secondhand smoke	Mazandaran	Miyandorud	Male	29.74	0.00	67.89
**4135**	Exposure to secondhand smoke	Azararbayjan_East	Miyaneh	Male	38.91	15.84	61.81
**4136**	Exposure to secondhand smoke	Isfahan	Mobarakeh	Male	49.85	27.71	74.00
**4137**	Exposure to secondhand smoke	Fars	Mohr	Male	45.36	18.74	71.97
**4138**	Exposure to secondhand smoke	Hamedan	Nahavand	Male	44.12	18.67	68.64
**4139**	Exposure to secondhand smoke	Isfahan	Najafabad	Male	49.05	31.51	67.20
**4140**	Exposure to secondhand smoke	Ardebil	Namin	Male	66.49	37.38	96.56
**4141**	Exposure to secondhand smoke	Azarbayjan_West	Naqadeh	Male	58.71	35.39	82.46
**4142**	Exposure to secondhand smoke	Kerman	Narmashir	Male	42.62	13.84	71.65
**4143**	Exposure to secondhand smoke	Isfahan	Natanz	Male	40.96	11.87	70.40
**4144**	Exposure to secondhand smoke	Isfahan	Nayin	Male	36.61	12.79	60.24
**4145**	Exposure to secondhand smoke	Alborz	Nazarabad	Male	36.33	12.26	60.45
**4146**	Exposure to secondhand smoke	Ardebil	Neer	Male	59.88	28.39	93.58
**4147**	Exposure to secondhand smoke	Khorasan_South	Nehbandan	Male	30.54	5.49	54.65
**4148**	Exposure to secondhand smoke	Mazandaran	Neka	Male	31.33	0.41	62.95
**4149**	Exposure to secondhand smoke	Fars	Neyriz	Male	48.22	24.34	71.51
**4150**	Exposure to secondhand smoke	Khorasan_razavi	Neyshabur	Male	29.01	10.06	47.09
**4151**	Exposure to secondhand smoke	Sistan and Balouchestan	Nikshahr	Male	33.23	11.39	52.79
**4152**	Exposure to secondhand smoke	Sistan and Balouchestan	Nimruz	Male	22.04	0.00	50.03
**4153**	Exposure to secondhand smoke	Mazandaran	Noshahr	Male	37.26	5.22	69.53
**4154**	Exposure to secondhand smoke	Mazandaran	Nur	Male	31.66	9.42	54.20
**4155**	Exposure to secondhand smoke	Khuzestan	Omidiyeh	Male	50.13	26.40	73.91
**4156**	Exposure to secondhand smoke	Azarbayjan_West	Orumiyeh	Male	54.31	33.53	75.77
**4157**	Exposure to secondhand smoke	Azarbayjan_West	Oshnaviyeh	Male	58.90	33.37	84.84
**4158**	Exposure to secondhand smoke	Azararbayjan_East	Osku	Male	34.34	7.67	61.66
**4159**	Exposure to secondhand smoke	Tehran	Pakdasht	Male	17.46	0.00	36.90
**4160**	Exposure to secondhand smoke	Tehran	Pardis	Male	25.96	0.00	55.53
**4161**	Exposure to secondhand smoke	Ardebil	Parsabad	Male	35.76	1.85	68.79
**4162**	Exposure to secondhand smoke	Hormozgan	Parsian (Gavbandi)	Male	18.23	0.00	39.14
**4163**	Exposure to secondhand smoke	Fars	Pasargad	Male	49.19	22.17	76.11
**4164**	Exposure to secondhand smoke	Kermanshah	Paveh	Male	45.16	24.65	67.06
**4165**	Exposure to secondhand smoke	Azarbayjan_West	Piranshahr	Male	62.29	36.26	90.77
**4166**	Exposure to secondhand smoke	Tehran	Pishva	Male	26.80	3.90	49.07
**4167**	Exposure to secondhand smoke	Azarbayjan_West	Poldasht	Male	23.21	0.00	49.74
**4168**	Exposure to secondhand smoke	Lorestan	Poldokhtar	Male	33.88	10.19	56.57
**4169**	Exposure to secondhand smoke	Mazandaran	Qaemshahr	Male	22.48	0.00	44.19
**4170**	Exposure to secondhand smoke	Tehran	Qarchak	Male	26.46	0.00	57.06
**4171**	Exposure to secondhand smoke	Sistan and Balouchestan	Qasr qand	Male	37.66	8.70	66.39
**4172**	Exposure to secondhand smoke	Kermanshah	Qasr-e-Shirin	Male	36.89	5.37	67.31
**4173**	Exposure to secondhand smoke	Khorasan_South	Qayenat	Male	30.73	9.84	51.64
**4174**	Exposure to secondhand smoke	Qazvin	Qazvin	Male	55.98	34.78	78.07
**4175**	Exposure to secondhand smoke	Hormozgan	Qeshm	Male	34.53	11.25	58.52
**4176**	Exposure to secondhand smoke	Fars	Qirokarzin	Male	41.14	21.06	60.51
**4177**	Exposure to secondhand smoke	Qom	Qom	Male	37.75	6.67	68.50
**4178**	Exposure to secondhand smoke	Kordestan	Qorveh	Male	21.71	0.00	49.70
**4179**	Exposure to secondhand smoke	Khorasan_razavi	Quchan	Male	45.68	21.82	71.18
**4180**	Exposure to secondhand smoke	Kerman	Rabar	Male	55.65	30.43	83.94
**4181**	Exposure to secondhand smoke	Kerman	Rafsanjan	Male	51.91	30.09	77.38
**4182**	Exposure to secondhand smoke	Khuzestan	Ramhormoz	Male	45.59	22.81	67.08
**4183**	Exposure to secondhand smoke	Mazandaran	Ramsar	Male	24.19	6.30	45.58
**4184**	Exposure to secondhand smoke	Khuzestan	Ramshir	Male	63.07	42.15	80.44
**4185**	Exposure to secondhand smoke	Golestan	Ramyan	Male	32.32	4.54	60.34
**4186**	Exposure to secondhand smoke	Gilan	Rasht	Male	20.42	4.59	37.27
**4187**	Exposure to secondhand smoke	Khorasan_razavi	Rashtkhar	Male	30.02	5.61	53.07
**4188**	Exposure to secondhand smoke	Kermanshah	Ravansar	Male	41.01	16.93	66.28
**4189**	Exposure to secondhand smoke	Kerman	Ravar	Male	21.88	1.29	44.95
**4190**	Exposure to secondhand smoke	Khorasan_North	Raz va Jergolan	Male	29.49	0.00	64.50
**4191**	Exposure to secondhand smoke	Hamedan	Razan	Male	49.34	21.12	78.62
**4192**	Exposure to secondhand smoke	Tehran	Rey	Male	28.47	7.79	49.16
**4193**	Exposure to secondhand smoke	Kerman	Reygan	Male	61.51	36.65	87.66
**4194**	Exposure to secondhand smoke	Gilan	Rezvanshahr	Male	33.22	5.58	60.06
**4195**	Exposure to secondhand smoke	Tehran	Robatkarim	Male	18.07	0.00	38.50
**4196**	Exposure to secondhand smoke	Fars	Rostam	Male	49.39	22.06	76.16
**4197**	Exposure to secondhand smoke	Kerman	Roudbar-e-Jonub	Male	45.90	19.39	71.68
**4198**	Exposure to secondhand smoke	Hormozgan	Rudan	Male	15.70	0.00	36.22
**4199**	Exposure to secondhand smoke	Gilan	Rudbar	Male	36.12	18.29	55.61
**4200**	Exposure to secondhand smoke	Gilan	Rudsar	Male	19.48	2.73	37.09
**4201**	Exposure to secondhand smoke	Lorestan	Rumshekan	Male	36.44	6.21	66.80
**4202**	Exposure to secondhand smoke	Khorasan_razavi	Sabzevar	Male	26.13	7.14	44.82
**4203**	Exposure to secondhand smoke	Yazd	Sadugh	Male	49.57	31.95	65.01
**4204**	Exposure to secondhand smoke	Kermanshah	Sahneh	Male	36.95	14.81	58.04
**4205**	Exposure to secondhand smoke	Kermanshah	Salas-e-Babajani	Male	38.23	14.04	61.75
**4206**	Exposure to secondhand smoke	Azarbayjan_West	Salmas	Male	61.61	36.54	86.75
**4207**	Exposure to secondhand smoke	Chaharmahal	Saman	Male	47.63	18.95	76.47
**4208**	Exposure to secondhand smoke	Kordestan	Sanandaj	Male	51.57	28.35	74.53
**4209**	Exposure to secondhand smoke	Kordestan	Saqqez	Male	61.64	38.99	85.57

**4210**	Exposure to secondhand smoke	Kermanshah	Sar-e-Pol-e-Zohab	Male	35.26	10.68	59.19
**4211**	Exposure to secondhand smoke	Azararbayjan_East	Sarab	Male	40.12	14.41	67.12
**4212**	Exposure to secondhand smoke	Khorasan_razavi	Sarakhs	Male	42.91	15.17	71.60
**4213**	Exposure to secondhand smoke	Sistan and Balouchestan	Saravan	Male	48.84	24.03	74.21
**4214**	Exposure to secondhand smoke	Khorasan_South	Sarayan	Male	27.76	0.98	54.80
**4215**	Exposure to secondhand smoke	Sistan and Balouchestan	Sarbaz	Male	39.20	18.94	59.60
**4216**	Exposure to secondhand smoke	Khorasan_South	Sarbisheh	Male	31.58	4.22	59.25
**4217**	Exposure to secondhand smoke	Azarbayjan_West	Sardasht	Male	61.83	37.04	89.15
**4218**	Exposure to secondhand smoke	Ardebil	Sarein	Male	55.04	16.87	91.69
**4219**	Exposure to secondhand smoke	Mazandaran	Sari	Male	25.30	3.70	45.74
**4220**	Exposure to secondhand smoke	Kordestan	Sarvabad	Male	53.45	21.20	85.95
**4221**	Exposure to secondhand smoke	Fars	Sarvestan	Male	50.35	21.14	78.12
**4222**	Exposure to secondhand smoke	Mazandaran	Savadkuh	Male	32.36	4.00	59.73
**4223**	Exposure to secondhand smoke	Mazandaran	Savadkuh_North	Male	29.24	0.00	60.18
**4224**	Exposure to secondhand smoke	Markazi	Saveh	Male	42.46	16.55	67.76
**4225**	Exposure to secondhand smoke	Alborz	Savojbolagh	Male	40.00	18.10	62.95
**4226**	Exposure to secondhand smoke	Lorestan	Selseleh	Male	36.99	11.08	61.29
**4227**	Exposure to secondhand smoke	Isfahan	Semirom	Male	49.07	26.54	71.04
**4228**	Exposure to secondhand smoke	Isfahan	Semirom-e-Sofla	Male	49.57	22.37	78.65
**4229**	Exposure to secondhand smoke	Semnan	Semnan	Male	24.77	5.10	43.45
**4230**	Exposure to secondhand smoke	Fars	Sepidan	Male	50.73	26.77	75.28
**4231**	Exposure to secondhand smoke	Azararbayjan_East	Shabestar	Male	23.65	1.89	45.31
**4232**	Exposure to secondhand smoke	Khuzestan	Shadegan	Male	36.63	13.19	58.96
**4233**	Exposure to secondhand smoke	Gilan	Shaft	Male	19.67	0.85	38.35
**4234**	Exposure to secondhand smoke	Azarbayjan_West	Shahindezh	Male	57.65	32.60	82.82
**4235**	Exposure to secondhand smoke	Tehran	Shahr-e Qods	Male	28.77	7.99	49.53
**4236**	Exposure to secondhand smoke	Kerman	Shahr-e-Babak	Male	39.16	12.01	63.67
**4237**	Exposure to secondhand smoke	Chaharmahal	Shahr-e-Kord	Male	40.77	20.58	60.10
**4238**	Exposure to secondhand smoke	Isfahan	Shahreza	Male	51.35	28.95	73.84
**4239**	Exposure to secondhand smoke	Tehran	Shahriyar	Male	31.36	12.39	50.97
**4240**	Exposure to secondhand smoke	Semnan	Shahrud	Male	27.49	7.08	48.01
**4241**	Exposure to secondhand smoke	Markazi	Shazand	Male	61.16	32.08	91.30
**4242**	Exposure to secondhand smoke	Tehran	Shemiranat	Male	18.83	0.00	38.49
**4243**	Exposure to secondhand smoke	Fars	Shiraz	Male	44.09	26.96	61.30
**4244**	Exposure to secondhand smoke	Khorasan_North	Shirvan	Male	29.26	6.79	50.88
**4245**	Exposure to secondhand smoke	Ilam	Shirvan and Chard-e-Aval	Male	52.62	27.04	79.11
**4246**	Exposure to secondhand smoke	Azarbayjan_West	Showt	Male	26.83	0.00	54.67
**4247**	Exposure to secondhand smoke	Khuzestan	Shush	Male	53.79	32.64	76.85
**4248**	Exposure to secondhand smoke	Khuzestan	Shushtar	Male	40.68	19.29	60.78
**4249**	Exposure to secondhand smoke	Gilan	Siahkal	Male	31.85	15.61	47.63
**4250**	Exposure to secondhand smoke	Sistan and Balouchestan	Sib o Soran	Male	43.92	18.83	70.03
**4251**	Exposure to secondhand smoke	Mazandaran	Simorgh	Male	29.08	0.00	61.03
**4252**	Exposure to secondhand smoke	Hormozgan	Sirik	Male	54.29	32.25	70.91
**4253**	Exposure to secondhand smoke	Kerman	Sirjan	Male	47.40	25.93	68.91
**4254**	Exposure to secondhand smoke	Ilam	Sirvan	Male	50.55	18.44	82.82
**4255**	Exposure to secondhand smoke	Zanjan	Soltaniyeh	Male	52.27	22.45	81.07
**4256**	Exposure to secondhand smoke	Kermanshah	Sonqor	Male	35.52	14.61	56.10
**4257**	Exposure to secondhand smoke	Semnan	Sorkheh	Male	28.97	1.66	55.51
**4258**	Exposure to secondhand smoke	Gilan	Sume'eh Sara	Male	31.42	8.87	54.64
**4259**	Exposure to secondhand smoke	Khorasan_South	Tabas	Male	30.49	9.26	51.42
**4260**	Exposure to secondhand smoke	Azararbayjan_East	Tabriz	Male	35.46	17.33	53.47
**4261**	Exposure to secondhand smoke	Markazi	Tafresh	Male	33.04	3.10	61.53
**4262**	Exposure to secondhand smoke	Yazd	Taft	Male	40.15	17.86	61.49
**4263**	Exposure to secondhand smoke	Azarbayjan_West	Takab	Male	65.42	46.18	81.70
**4264**	Exposure to secondhand smoke	Qazvin	Takestan	Male	51.45	26.82	75.96
**4265**	Exposure to secondhand smoke	Khorasan_razavi	Takht-e-Jolgeh (Firuzeh)	Male	26.15	8.54	46.03
**4266**	Exposure to secondhand smoke	Alborz	Taleghan	Male	40.51	18.49	63.22
**4267**	Exposure to secondhand smoke	Boushehr	Tangestan	Male	48.07	20.19	77.06
**4268**	Exposure to secondhand smoke	Zanjan	Tarom	Male	56.45	33.54	81.54
**4269**	Exposure to secondhand smoke	Gilan	Tavalesh	Male	44.06	22.92	65.79
**4270**	Exposure to secondhand smoke	Khorasan_razavi	Taybad	Male	46.54	21.89	72.78
**4271**	Exposure to secondhand smoke	Tehran	Tehran	Male	24.60	9.71	40.65
**4272**	Exposure to secondhand smoke	Isfahan	Tiran and Karvan	Male	56.25	34.45	78.99
**4273**	Exposure to secondhand smoke	Mazandaran	Tonekabon	Male	41.25	16.40	66.47
**4274**	Exposure to secondhand smoke	Khorasan_razavi	Torbat-e-Heydariyeh	Male	36.62	15.07	58.40
**4275**	Exposure to secondhand smoke	Khorasan_razavi	Torbat-e-Jam	Male	44.83	22.40	68.61
**4276**	Exposure to secondhand smoke	Hamedan	Tuyserkan	Male	49.86	23.86	76.26
**4277**	Exposure to secondhand smoke	Tehran	Varamin	Male	28.03	8.72	47.41
**4278**	Exposure to secondhand smoke	Azararbayjan_East	Varzaqan	Male	26.44	0.00	55.83
**4279**	Exposure to secondhand smoke	Yazd	Yazd	Male	31.19	13.80	48.43
**4280**	Exposure to secondhand smoke	Sistan and Balouchestan	Zabol	Male	12.61	0.00	31.98
**4281**	Exposure to secondhand smoke	Sistan and Balouchestan	Zaboli (Mehrestan)	Male	39.55	11.24	68.53
**4282**	Exposure to secondhand smoke	Sistan and Balouchestan	Zahedan	Male	26.38	9.34	43.28
**4283**	Exposure to secondhand smoke	Zanjan	Zanjan	Male	49.43	29.74	69.28
**4284**	Exposure to secondhand smoke	Kerman	Zarand	Male	31.11	5.98	55.42
**4285**	Exposure to secondhand smoke	Markazi	Zarandiyeh	Male	53.90	26.42	84.24
**4286**	Exposure to secondhand smoke	Fars	Zarrindasht	Male	62.43	41.39	83.27
**4287**	Exposure to secondhand smoke	Khorasan_razavi	Zave	Male	41.70	18.47	65.54
**4288**	Exposure to secondhand smoke	Sistan and Balouchestan	Zehak	Male	16.98	0.00	48.72
**4289**	Exposure to secondhand smoke	Khorasan_South	Zir kuh	Male	31.81	2.73	61.85
**4290**	Exposure to secondhand smoke at home	Khuzestan	Abadan	Female	28.10	7.64	48.89
**4291**	Exposure to secondhand smoke at home	Fars	Abadeh	Female	46.70	26.47	67.97
**4292**	Exposure to secondhand smoke at home	Yazd	Abarkuh	Female	40.18	19.00	61.76
**4293**	Exposure to secondhand smoke at home	Mazandaran	Abbas abad	Female	40.51	20.10	58.26
**4294**	Exposure to secondhand smoke at home	Ilam	Abdanan	Female	28.33	14.37	42.66
**4295**	Exposure to secondhand smoke at home	Zanjan	Abhar	Female	31.48	11.45	50.98
**4296**	Exposure to secondhand smoke at home	Hormozgan	Abumusa	Female	31.13	0.00	66.14
**4297**	Exposure to secondhand smoke at home	Qazvin	Abyek	Female	47.27	19.19	77.32
**4298**	Exposure to secondhand smoke at home	Azararbayjan_East	Ahar	Female	20.24	0.00	41.30
**4299**	Exposure to secondhand smoke at home	Khuzestan	Ahvaz	Female	30.15	15.88	44.15
**4300**	Exposure to secondhand smoke at home	Azararbayjan_East	Ajabshir	Female	25.91	0.59	51.06
**4301**	Exposure to secondhand smoke at home	Qazvin	Alborz	Female	47.26	16.92	78.22
**4302**	Exposure to secondhand smoke at home	Golestan	Aliabad	Female	18.88	0.41	38.05
**4303**	Exposure to secondhand smoke at home	Lorestan	Aligudarz	Female	26.80	8.12	44.99
**4304**	Exposure to secondhand smoke at home	Gilan	Amlash	Female	13.14	0.00	39.53
**4305**	Exposure to secondhand smoke at home	Mazandaran	Amol	Female	22.00	1.23	43.07
**4306**	Exposure to secondhand smoke at home	Kerman	Anar	Female	31.14	0.74	61.35
**4307**	Exposure to secondhand smoke at home	Kerman	Anbarabad	Female	32.62	11.56	54.78

**4308**	Exposure to secondhand smoke at home	Khuzestan	Andika	Female	32.12	11.76	53.34
**4309**	Exposure to secondhand smoke at home	Khuzestan	Andimeshk	Female	28.17	8.88	47.27
**4310**	Exposure to secondhand smoke at home	Golestan	Aq Qala	Female	11.16	0.00	27.28
**4311**	Exposure to secondhand smoke at home	Khuzestan	Aqajari	Female	30.79	2.68	58.94
**4312**	Exposure to secondhand smoke at home	Semnan	Aradan	Female	15.48	0.00	38.62
**4313**	Exposure to secondhand smoke at home	Markazi	Arak	Female	31.93	14.83	48.23
**4314**	Exposure to secondhand smoke at home	Isfahan	Aran and Bidgol	Female	9.01	0.00	26.69
**4315**	Exposure to secondhand smoke at home	Ardebil	Ardabil	Female	36.68	14.83	57.45
**4316**	Exposure to secondhand smoke at home	Yazd	Ardakan	Female	28.18	11.24	46.67
**4317**	Exposure to secondhand smoke at home	Chaharmahal	Ardal	Female	36.42	12.46	62.62
**4318**	Exposure to secondhand smoke at home	Isfahan	Ardestan	Female	21.91	0.00	45.76
**4319**	Exposure to secondhand smoke at home	Fars	Arsanjan	Female	42.07	18.64	65.30
**4320**	Exposure to secondhand smoke at home	Kerman	Arzouyeh	Female	33.55	8.61	59.00
**4321**	Exposure to secondhand smoke at home	Hamedan	Asadabad	Female	27.17	5.22	48.75
**4322**	Exposure to secondhand smoke at home	Boushehr	Asaluyeh	Female	33.46	10.15	57.20
**4323**	Exposure to secondhand smoke at home	Markazi	Ashtiyan	Female	28.10	3.61	51.84
**4324**	Exposure to secondhand smoke at home	Gilan	Astaneh-ye-Ashrafiyeh	Female	17.94	0.00	41.44
**4325**	Exposure to secondhand smoke at home	Gilan	Astara	Female	25.66	0.00	54.90
**4326**	Exposure to secondhand smoke at home	Qazvin	Avaj	Female	44.62	14.80	74.99
**4327**	Exposure to secondhand smoke at home	Golestan	Azadshahr	Female	15.17	0.00	32.00
**4328**	Exposure to secondhand smoke at home	Azararbayjan_East	Azarshahr	Female	26.44	0.00	54.46
**4329**	Exposure to secondhand smoke at home	Lorestan	Azna	Female	35.86	10.47	62.71
**4330**	Exposure to secondhand smoke at home	Mazandaran	Babol	Female	27.17	5.52	50.42
**4331**	Exposure to secondhand smoke at home	Mazandaran	Babolsar	Female	23.78	0.00	53.67
**4332**	Exposure to secondhand smoke at home	Ilam	Badreh	Female	25.01	2.59	48.80
**4333**	Exposure to secondhand smoke at home	Yazd	Bafq	Female	19.61	7.06	34.28
**4334**	Exposure to secondhand smoke at home	Kerman	Baft	Female	31.52	13.04	50.89
**4335**	Exposure to secondhand smoke at home	Khuzestan	Baghemalek	Female	18.17	4.10	34.71
**4336**	Exposure to secondhand smoke at home	Yazd	Bahabad	Female	23.78	0.38	48.18
**4337**	Exposure to secondhand smoke at home	Hamedan	Bahar	Female	41.95	17.28	67.90
**4338**	Exposure to secondhand smoke at home	Tehran	Baharestan (Golestan)	Female	23.64	4.38	43.44
**4339**	Exposure to secondhand smoke at home	Kohkiluye and Bouyer Ahmad	Bahmani	Female	37.91	13.13	62.25
**4340**	Exposure to secondhand smoke at home	Khorasan_razavi	Bajestan	Female	22.52	2.14	43.17
**4341**	Exposure to secondhand smoke at home	Khorasan_razavi	Bakhraz	Female	29.86	11.67	48.15
**4342**	Exposure to secondhand smoke at home	Kerman	Bam	Female	29.68	9.04	50.50
**4343**	Exposure to secondhand smoke at home	Hormozgan	Bandar-e-Abbas	Female	27.48	11.17	44.01
**4344**	Exposure to secondhand smoke at home	Gilan	Bandar-e-Anzali	Female	17.84	0.00	39.46
**4345**	Exposure to secondhand smoke at home	Golestan	Bandar-e-Gaz	Female	15.46	0.00	41.21
**4346**	Exposure to secondhand smoke at home	Hormozgan	Bandar-e-Jask	Female	33.97	6.89	61.28
**4347**	Exposure to secondhand smoke at home	Hormozgan	Bandar-e-Lengeh	Female	30.23	7.77	52.28
**4348**	Exposure to secondhand smoke at home	Khuzestan	Bandar-e-Mahshahr	Female	30.84	12.06	49.70
**4349**	Exposure to secondhand smoke at home	Golestan	Bandar-e-Torkaman	Female	13.81	0.00	38.86
**4350**	Exposure to secondhand smoke at home	Kordestan	Baneh	Female	50.07	20.99	80.04
**4351**	Exposure to secondhand smoke at home	Khorasan_razavi	Bardeskan	Female	11.24	0.00	26.93
**4352**	Exposure to secondhand smoke at home	Kerman	Bardsir	Female	27.99	10.91	45.67
**4353**	Exposure to secondhand smoke at home	Hormozgan	Bashagerd	Female	32.50	9.52	55.13
**4354**	Exposure to secondhand smoke at home	Kohkiluye and Bouyer Ahmad	Basht	Female	56.85	35.14	78.21
**4355**	Exposure to secondhand smoke at home	Hormozgan	Bastak	Female	34.82	9.00	60.80
**4356**	Exposure to secondhand smoke at home	Khuzestan	Bavi	Female	19.65	0.32	38.41
**4357**	Exposure to secondhand smoke at home	Khuzestan	Behbahan	Female	27.14	9.80	44.25
**4358**	Exposure to secondhand smoke at home	Mazandaran	Behshahr	Female	7.17	0.00	28.39
**4359**	Exposure to secondhand smoke at home	Kordestan	Bijar	Female	44.06	22.43	66.29
**4360**	Exposure to secondhand smoke at home	Ardebil	Bilehsavar	Female	30.76	0.00	72.44
**4361**	Exposure to secondhand smoke at home	Khorasan_razavi	Binaloud	Female	41.59	23.65	55.83
**4362**	Exposure to secondhand smoke at home	Khorasan_South	Birjand	Female	16.47	0.55	32.30
**4363**	Exposure to secondhand smoke at home	Khorasan_North	Bojnurd	Female	22.77	5.47	39.70
**4364**	Exposure to secondhand smoke at home	Chaharmahal	Bon	Female	29.49	0.00	61.83
**4365**	Exposure to secondhand smoke at home	Azararbayjan_East	Bonab	Female	35.70	9.84	62.54
**4366**	Exposure to secondhand smoke at home	Isfahan	Borkhar	Female	17.92	0.32	36.05
**4367**	Exposure to secondhand smoke at home	Isfahan	Borkhar and Meymeh	Female	30.55	12.72	49.93
**4368**	Exposure to secondhand smoke at home	Chaharmahal	Borujen	Female	25.82	6.88	44.22
**4369**	Exposure to secondhand smoke at home	Lorestan	Borujerd	Female	38.70	20.46	56.69
**4370**	Exposure to secondhand smoke at home	Khorasan_South	Boshruyeh	Female	12.60	0.00	30.05
**4371**	Exposure to secondhand smoke at home	Azararbayjan_East	Bostanabad	Female	26.49	5.84	47.36
**4372**	Exposure to secondhand smoke at home	Fars	Bovanat	Female	45.03	23.35	65.87
**4373**	Exposure to secondhand smoke at home	Kohkiluye and Bouyer Ahmad	Boyer Ahmad	Female	53.03	32.50	73.57
**4374**	Exposure to secondhand smoke at home	Qazvin	Boyinzahra	Female	51.34	26.30	77.96
**4375**	Exposure to secondhand smoke at home	Isfahan	Buein va Miandasht	Female	29.69	4.22	55.74
**4376**	Exposure to secondhand smoke at home	Azarbayjan_West	Bukan	Female	48.30	26.86	70.09
**4377**	Exposure to secondhand smoke at home	Boushehr	Bushehr	Female	29.23	10.30	47.72
**4378**	Exposure to secondhand smoke at home	Isfahan	Chadegan	Female	33.01	10.96	56.11
**4379**	Exposure to secondhand smoke at home	Sistan and Balouchestan	Chahbahar	Female	37.01	12.68	62.03
**4380**	Exposure to secondhand smoke at home	Azarbayjan_West	Chaipareh	Female	24.51	0.00	51.13
**4381**	Exposure to secondhand smoke at home	Azarbayjan_West	Chaldoran	Female	25.38	0.00	54.70
**4382**	Exposure to secondhand smoke at home	Mazandaran	Chalus	Female	28.43	5.40	52.23
**4383**	Exposure to secondhand smoke at home	Azararbayjan_East	Charoimaq	Female	37.08	13.16	61.58
**4384**	Exposure to secondhand smoke at home	Khorasan_razavi	Chenaran	Female	28.65	6.56	50.58
**4385**	Exposure to secondhand smoke at home	Kohkiluye and Bouyer Ahmad	Cheram	Female	49.48	22.91	76.13
**4386**	Exposure to secondhand smoke at home	Kermanshah	Dalaho	Female	22.29	4.90	39.60
**4387**	Exposure to secondhand smoke at home	Lorestan	Dalfan	Female	39.35	19.34	60.25
**4388**	Exposure to secondhand smoke at home	Sistan and Balouchestan	Dalgan	Female	28.59	3.97	52.84
**4389**	Exposure to secondhand smoke at home	Tehran	Damavand	Female	12.64	0.00	30.03
**4390**	Exposure to secondhand smoke at home	Semnan	Damghan	Female	19.68	3.92	36.98
**4391**	Exposure to secondhand smoke at home	Fars	Darab	Female	47.49	28.20	67.68
**4392**	Exposure to secondhand smoke at home	Khorasan_South	Darmian	Female	16.05	0.00	34.37
**4393**	Exposure to secondhand smoke at home	Khorasan_razavi	Darrehgaz	Female	14.36	0.00	32.23
**4394**	Exposure to secondhand smoke at home	Ilam	Darrehshahr	Female	21.32	1.80	40.86
**4395**	Exposure to secondhand smoke at home	Khuzestan	Dasht-e-Azadegan	Female	44.80	20.27	68.60
**4396**	Exposure to secondhand smoke at home	Boushehr	Dashtestan	Female	33.69	16.20	51.40
**4397**	Exposure to secondhand smoke at home	Boushehr	Dashti	Female	34.28	14.58	54.40
**4398**	Exposure to secondhand smoke at home	Khorasan_razavi	Davarzan	Female	20.81	0.00	45.94
**4399**	Exposure to secondhand smoke at home	Boushehr	Dayyer	Female	38.15	14.39	62.92
**4400**	Exposure to secondhand smoke at home	Kordestan	Dehgolan	Female	44.80	19.63	71.85
**4401**	Exposure to secondhand smoke at home	Ilam	Dehloran	Female	27.06	7.59	47.14
**4402**	Exposure to secondhand smoke at home	Markazi	Delijan	Female	32.37	11.98	53.60
**4403**	Exposure to secondhand smoke at home	Kohkiluye and Bouyer Ahmad	Dena	Female	52.34	26.81	78.45
**4404**	Exposure to secondhand smoke at home	Boushehr	Deylam	Female	30.97	8.21	53.64
**4405**	Exposure to secondhand smoke at home	Khuzestan	Dezful	Female	32.25	15.70	48.64

**4406**	Exposure to secondhand smoke at home	Kordestan	Divandarreh	Female	48.96	25.68	72.73
**4407**	Exposure to secondhand smoke at home	Lorestan	Dorud	Female	36.75	13.63	60.96
**4408**	Exposure to secondhand smoke at home	Lorestan	Doureh	Female	25.91	11.64	41.69
**4409**	Exposure to secondhand smoke at home	Fars	Eqlid	Female	45.03	24.88	65.30
**4410**	Exposure to secondhand smoke at home	Khorasan_North	Esfarayen	Female	24.75	5.33	44.20
**4411**	Exposure to secondhand smoke at home	Alborz	Eshtehard	Female	23.20	4.41	41.94
**4412**	Exposure to secondhand smoke at home	Kermanshah	Eslamabad-e-Gharb	Female	8.47	0.00	25.27
**4413**	Exposure to secondhand smoke at home	Tehran	Eslamshahr	Female	11.39	0.00	28.39
**4414**	Exposure to secondhand smoke at home	Fars	Estahban	Female	43.01	19.32	66.39
**4415**	Exposure to secondhand smoke at home	Ilam	Eyvan	Female	27.85	6.26	50.01
**4416**	Exposure to secondhand smoke at home	Kerman	Fahraj	Female	37.25	19.51	55.13
**4417**	Exposure to secondhand smoke at home	Isfahan	Falavarjan	Female	38.04	16.69	61.52
**4418**	Exposure to secondhand smoke at home	Hamedan	Famenin	Female	36.77	10.22	63.11
**4419**	Exposure to secondhand smoke at home	Markazi	Farahan	Female	29.03	4.37	53.15
**4420**	Exposure to secondhand smoke at home	Fars	Farashband	Female	46.62	25.78	68.91
**4421**	Exposure to secondhand smoke at home	Alborz	Fardis	Female	18.17	0.00	40.73
**4422**	Exposure to secondhand smoke at home	Isfahan	Faridan	Female	34.49	13.23	57.05
**4423**	Exposure to secondhand smoke at home	Khorasan_razavi	Fariman	Female	20.26	1.61	38.54
**4424**	Exposure to secondhand smoke at home	Khorasan_North	Faroj	Female	28.93	7.18	52.01
**4425**	Exposure to secondhand smoke at home	Chaharmahal	Farsan	Female	31.73	6.02	56.98
**4426**	Exposure to secondhand smoke at home	Kerman	Faryab	Female	30.82	5.09	56.17
**4427**	Exposure to secondhand smoke at home	Fars	Fasa	Female	49.86	30.35	69.82
**4428**	Exposure to secondhand smoke at home	Khorasan_South	Ferdows	Female	12.95	0.00	30.01
**4429**	Exposure to secondhand smoke at home	Mazandaran	Fereydunkenar	Female	17.13	0.00	39.29
**4430**	Exposure to secondhand smoke at home	Isfahan	Fereydunshahr	Female	28.65	5.11	52.78
**4431**	Exposure to secondhand smoke at home	Fars	Firozabad	Female	58.25	39.07	77.90
**4432**	Exposure to secondhand smoke at home	Tehran	Firuzkuh	Female	7.46	0.00	23.34
**4433**	Exposure to secondhand smoke at home	Sistan and Balouchestan	Fonuj	Female	31.23	3.01	59.40
**4434**	Exposure to secondhand smoke at home	Gilan	Fuman	Female	15.59	0.00	32.41
**4435**	Exposure to secondhand smoke at home	Kohkiluye and Bouyer Ahmad	Gachsaran	Female	52.69	30.88	75.50
**4436**	Exposure to secondhand smoke at home	Golestan	Galikesh	Female	20.40	1.11	41.15
**4437**	Exposure to secondhand smoke at home	Mazandaran	Galugah	Female	16.47	0.00	54.99
**4438**	Exposure to secondhand smoke at home	Semnan	Garmsar	Female	15.68	0.00	32.07
**4439**	Exposure to secondhand smoke at home	Boushehr	Genaveh	Female	29.41	10.56	48.03
**4440**	Exposure to secondhand smoke at home	Fars	Gerash	Female	46.62	17.95	76.45
**4441**	Exposure to secondhand smoke at home	Khorasan_North	Germeh	Female	30.43	14.58	44.22
**4442**	Exposure to secondhand smoke at home	Ardebil	Germi	Female	32.17	2.77	62.20
**4443**	Exposure to secondhand smoke at home	Kerman	Ghaleye-Ganj	Female	23.62	3.90	42.12
**4444**	Exposure to secondhand smoke at home	Kermanshah	Gilan-e-Gharb	Female	26.28	8.46	45.51
**4445**	Exposure to secondhand smoke at home	Isfahan	Golpayegan	Female	31.27	9.19	53.11
**4446**	Exposure to secondhand smoke at home	Golestan	Gomishan	Female	13.37	0.00	45.16
**4447**	Exposure to secondhand smoke at home	Khorasan_razavi	Gonabad	Female	30.58	16.46	43.12
**4448**	Exposure to secondhand smoke at home	Golestan	Gonbad-e-Kavus	Female	16.62	0.74	31.74
**4449**	Exposure to secondhand smoke at home	Golestan	Gorgan	Female	12.31	0.00	27.20
**4450**	Exposure to secondhand smoke at home	Khuzestan	Guotvand	Female	30.66	12.18	48.78
**4451**	Exposure to secondhand smoke at home	Khuzestan	Haftgol	Female	28.56	6.03	51.00
**4452**	Exposure to secondhand smoke at home	Hormozgan	Hajiabad	Female	35.73	17.57	54.51
**4453**	Exposure to secondhand smoke at home	Hamedan	Hamadan	Female	39.84	19.93	59.77
**4454**	Exposure to secondhand smoke at home	Khuzestan	Hamidiyeh	Female	43.26	18.89	69.66
**4455**	Exposure to secondhand smoke at home	Sistan and Balouchestan	Hamoon	Female	20.75	0.00	50.49
**4456**	Exposure to secondhand smoke at home	Azararbayjan_East	Haris	Female	24.19	2.90	45.08
**4457**	Exposure to secondhand smoke at home	Kermanshah	Harsin	Female	60.05	37.63	78.47
**4458**	Exposure to secondhand smoke at home	Azararbayjan_East	Hashtrud	Female	33.43	10.10	57.40
**4459**	Exposure to secondhand smoke at home	Khuzestan	Hendijan	Female	32.57	6.86	57.56
**4460**	Exposure to secondhand smoke at home	Sistan and Balouchestan	Hirmand	Female	18.79	0.00	47.24
**4461**	Exposure to secondhand smoke at home	Khuzestan	Hoveizeh	Female	61.36	41.06	76.25
**4462**	Exposure to secondhand smoke at home	Zanjan	Ijerod	Female	48.84	26.42	71.94
**4463**	Exposure to secondhand smoke at home	Ilam	Ilam	Female	24.79	6.86	42.57
**4464**	Exposure to secondhand smoke at home	Sistan and Balouchestan	Iranshahr	Female	34.14	13.34	55.52
**4465**	Exposure to secondhand smoke at home	Isfahan	Isfahan	Female	26.38	12.66	40.17
**4466**	Exposure to secondhand smoke at home	Khuzestan	Izeh	Female	30.65	12.77	48.31
**4467**	Exposure to secondhand smoke at home	Fars	Jahrom	Female	43.57	24.61	62.39
**4468**	Exposure to secondhand smoke at home	Khorasan_North	Jajarm	Female	23.01	6.07	40.09
**4469**	Exposure to secondhand smoke at home	Boushehr	Jam	Female	29.89	14.02	46.27
**4470**	Exposure to secondhand smoke at home	Kermanshah	Javanrud	Female	31.09	11.33	50.66
**4471**	Exposure to secondhand smoke at home	Kerman	Jiroft	Female	24.73	7.35	41.37
**4472**	Exposure to secondhand smoke at home	Khorasan_razavi	Joghatai	Female	21.85	0.00	47.90
**4473**	Exposure to secondhand smoke at home	Azararbayjan_East	Jolfa	Female	19.11	4.43	33.81
**4474**	Exposure to secondhand smoke at home	Khorasan_razavi	Jowayin	Female	22.77	0.00	46.75
**4475**	Exposure to secondhand smoke at home	Mazandaran	Juybar	Female	11.00	0.00	31.80
**4476**	Exposure to secondhand smoke at home	Hamedan	Kabudarahang	Female	41.42	18.80	64.28
**4477**	Exposure to secondhand smoke at home	Kerman	Kahnuj	Female	25.55	6.18	44.90
**4478**	Exposure to secondhand smoke at home	Golestan	Kalaleh	Female	18.54	0.00	37.30
**4479**	Exposure to secondhand smoke at home	Khorasan_razavi	Kalat	Female	23.22	0.00	51.10
**4480**	Exposure to secondhand smoke at home	Azararbayjan_East	Kaleibar	Female	22.36	0.00	49.49
**4481**	Exposure to secondhand smoke at home	Kordestan	Kamyaran	Female	37.03	12.59	61.27
**4482**	Exposure to secondhand smoke at home	Boushehr	Kangan	Female	34.00	12.25	55.69
**4483**	Exposure to secondhand smoke at home	Kermanshah	Kangavar	Female	30.57	12.27	48.97
**4484**	Exposure to secondhand smoke at home	Alborz	Karaj	Female	15.55	4.01	27.97
**4485**	Exposure to secondhand smoke at home	Khuzestan	Karun	Female	32.24	0.00	63.76
**4486**	Exposure to secondhand smoke at home	Isfahan	Kashan	Female	27.36	8.55	47.13
**4487**	Exposure to secondhand smoke at home	Khorasan_razavi	Kashmar	Female	19.35	2.15	35.83
**4488**	Exposure to secondhand smoke at home	Fars	Kavar	Female	42.72	19.35	65.21
**4489**	Exposure to secondhand smoke at home	Fars	Kazerun	Female	39.00	19.23	57.72
**4490**	Exposure to secondhand smoke at home	Mazandaran	Kelardasht	Female	34.09	0.91	68.00
**4491**	Exposure to secondhand smoke at home	Kerman	Kerman	Female	25.37	10.26	39.94
**4492**	Exposure to secondhand smoke at home	Kermanshah	Kermanshah	Female	21.49	8.34	34.94
**4493**	Exposure to secondhand smoke at home	Khorasan_razavi	Khaf	Female	34.97	15.48	55.51
**4494**	Exposure to secondhand smoke at home	Khorasan_razavi	Khalilabad	Female	24.77	2.73	47.31
**4495**	Exposure to secondhand smoke at home	Ardebil	Khalkhal	Female	33.56	9.58	58.25
**4496**	Exposure to secondhand smoke at home	Hormozgan	Khamir	Female	29.55	6.96	51.95
**4497**	Exposure to secondhand smoke at home	Isfahan	Khansar	Female	23.62	2.89	44.47
**4498**	Exposure to secondhand smoke at home	Sistan and Balouchestan	Khash	Female	29.83	8.25	51.19
**4499**	Exposure to secondhand smoke at home	Yazd	Khatam	Female	54.67	33.51	75.76
**4500**	Exposure to secondhand smoke at home	Fars	Kherameh	Female	41.15	20.11	61.77
**4501**	Exposure to secondhand smoke at home	Azararbayjan_East	Khodaafarin	Female	20.79	0.00	49.52
**4502**	Exposure to secondhand smoke at home	Zanjan	Khodabandeh	Female	49.37	29.85	70.78
**4503**	Exposure to secondhand smoke at home	Markazi	Khomeyn	Female	33.72	14.56	51.65

**4504**	Exposure to secondhand smoke at home	Isfahan	Khomeynishahr	Female	41.91	21.17	62.92
**4505**	Exposure to secondhand smoke at home	Markazi	Khondab	Female	62.84	40.04	82.59
**4506**	Exposure to secondhand smoke at home	Fars	Khonj	Female	48.17	26.52	69.87
**4507**	Exposure to secondhand smoke at home	Isfahan	Khoor va Biabanak	Female	20.36	0.00	45.82
**4508**	Exposure to secondhand smoke at home	Lorestan	Khorramabad	Female	27.96	9.55	44.48
**4509**	Exposure to secondhand smoke at home	Fars	Khorrambid	Female	44.19	20.64	67.75
**4510**	Exposure to secondhand smoke at home	Zanjan	Khorramdarreh	Female	30.19	7.37	53.46
**4511**	Exposure to secondhand smoke at home	Khuzestan	Khorramshahr	Female	32.03	7.99	56.89
**4512**	Exposure to secondhand smoke at home	Khorasan_razavi	Khoshab	Female	22.54	0.00	45.95
**4513**	Exposure to secondhand smoke at home	Azarbayjan_West	Khoy	Female	21.28	2.69	40.39
**4514**	Exposure to secondhand smoke at home	Khorasan_South	Khusef	Female	17.73	0.00	40.00
**4515**	Exposure to secondhand smoke at home	Chaharmahal	Kiaar	Female	36.19	13.63	59.25
**4516**	Exposure to secondhand smoke at home	Kohkiluye and Bouyer Ahmad	Kohgiluyeh	Female	42.23	21.12	63.68
**4517**	Exposure to secondhand smoke at home	Markazi	Komeijan	Female	33.37	10.95	56.69
**4518**	Exposure to secondhand smoke at home	Sistan and Balouchestan	Konarak	Female	37.97	11.90	64.61
**4519**	Exposure to secondhand smoke at home	Golestan	Kordkuy	Female	13.85	0.00	32.39
**4520**	Exposure to secondhand smoke at home	Ardebil	Kowsar	Female	34.59	5.71	62.55
**4521**	Exposure to secondhand smoke at home	Kerman	Kuhbonan	Female	22.71	0.00	49.43
**4522**	Exposure to secondhand smoke at home	Lorestan	Kuhdasht	Female	18.53	1.92	36.75
**4523**	Exposure to secondhand smoke at home	Chaharmahal	Kuhrang	Female	32.68	10.26	54.90
**4524**	Exposure to secondhand smoke at home	Gilan	Lahijan	Female	17.32	0.00	37.00
**4525**	Exposure to secondhand smoke at home	Khuzestan	Lali	Female	35.85	13.12	59.49
**4526**	Exposure to secondhand smoke at home	Fars	Lamard	Female	57.65	38.39	77.58
**4527**	Exposure to secondhand smoke at home	Kohkiluye and Bouyer Ahmad	Landeh	Female	40.76	1.49	80.23
**4528**	Exposure to secondhand smoke at home	Gilan	Langrud	Female	8.59	0.00	23.69
**4529**	Exposure to secondhand smoke at home	Isfahan	Lanjan	Female	26.11	7.56	44.60
**4530**	Exposure to secondhand smoke at home	Fars	Lar (Larestan)	Female	41.37	22.81	59.50
**4531**	Exposure to secondhand smoke at home	Chaharmahal	Lordakan	Female	40.44	20.79	60.82
**4532**	Exposure to secondhand smoke at home	Azarbayjan_West	Mahabad	Female	47.92	25.29	71.35
**4533**	Exposure to secondhand smoke at home	Markazi	Mahalat	Female	36.28	16.81	56.05
**4534**	Exposure to secondhand smoke at home	Mazandaran	Mahmudabad	Female	27.80	0.12	56.06
**4535**	Exposure to secondhand smoke at home	Zanjan	Mahneshan	Female	58.63	36.59	82.00
**4536**	Exposure to secondhand smoke at home	Khorasan_razavi	Mahvelat	Female	23.06	0.05	46.07
**4537**	Exposure to secondhand smoke at home	Azarbayjan_West	Maku	Female	30.50	1.34	60.35
**4538**	Exposure to secondhand smoke at home	Tehran	Malard	Female	23.09	3.26	43.66
**4539**	Exposure to secondhand smoke at home	Hamedan	Malayer	Female	53.09	30.37	76.39
**4540**	Exposure to secondhand smoke at home	Azararbayjan_East	Malekan	Female	37.65	10.78	65.45
**4541**	Exposure to secondhand smoke at home	Ilam	Malekshahi	Female	23.18	0.00	50.79
**4542**	Exposure to secondhand smoke at home	Fars	Mamasany	Female	48.33	29.92	67.70
**4543**	Exposure to secondhand smoke at home	Khorasan_North	Maneh and Samalqan	Female	23.85	5.82	41.66
**4544**	Exposure to secondhand smoke at home	Kerman	Manujan	Female	30.15	3.95	55.82
**4545**	Exposure to secondhand smoke at home	Azararbayjan_East	Maragheh	Female	30.91	12.43	49.10
**4546**	Exposure to secondhand smoke at home	Azararbayjan_East	Marand	Female	13.34	0.00	30.45
**4547**	Exposure to secondhand smoke at home	Golestan	Maravehtapeh	Female	31.56	12.99	49.73
**4548**	Exposure to secondhand smoke at home	Kordestan	Marivan	Female	35.62	14.32	57.14
**4549**	Exposure to secondhand smoke at home	Fars	Marvdasht	Female	38.61	20.30	56.65
**4550**	Exposure to secondhand smoke at home	Gilan	Masal	Female	20.43	0.00	43.11
**4551**	Exposure to secondhand smoke at home	Khorasan_razavi	Mashhad	Female	20.62	7.84	34.63
**4552**	Exposure to secondhand smoke at home	Khuzestan	Masjed Soleyman	Female	41.11	23.04	61.18
**4553**	Exposure to secondhand smoke at home	Semnan	Mayamey	Female	17.57	0.00	38.05
**4554**	Exposure to secondhand smoke at home	Semnan	Mehdishahr	Female	15.41	0.00	37.05
**4555**	Exposure to secondhand smoke at home	Ilam	Mehran	Female	12.89	0.00	32.39
**4556**	Exposure to secondhand smoke at home	Yazd	Mehriz	Female	29.62	10.55	48.36
**4557**	Exposure to secondhand smoke at home	Ardebil	Meshkinshahr	Female	30.68	7.91	53.83
**4558**	Exposure to secondhand smoke at home	Yazd	Meybod	Female	24.51	7.28	41.28
**4559**	Exposure to secondhand smoke at home	Hormozgan	Minab	Female	30.64	8.52	52.68
**4560**	Exposure to secondhand smoke at home	Golestan	Minudasht	Female	6.30	0.00	22.11
**4561**	Exposure to secondhand smoke at home	Sistan and Balouchestan	Mirjaveh	Female	28.80	0.00	63.28
**4562**	Exposure to secondhand smoke at home	Azarbayjan_West	Miyandoab	Female	43.12	23.60	62.72
**4563**	Exposure to secondhand smoke at home	Mazandaran	Miyandorud	Female	22.96	0.00	66.37
**4564**	Exposure to secondhand smoke at home	Azararbayjan_East	Miyaneh	Female	30.42	9.22	52.42
**4565**	Exposure to secondhand smoke at home	Isfahan	Mobarakeh	Female	30.03	8.11	51.77
**4566**	Exposure to secondhand smoke at home	Fars	Mohr	Female	46.19	24.37	68.34
**4567**	Exposure to secondhand smoke at home	Hamedan	Nahavand	Female	54.65	31.80	79.77
**4568**	Exposure to secondhand smoke at home	Isfahan	Najafabad	Female	30.96	12.87	49.66
**4569**	Exposure to secondhand smoke at home	Ardebil	Namin	Female	36.66	4.27	70.00
**4570**	Exposure to secondhand smoke at home	Azarbayjan_West	Naqadeh	Female	52.80	30.25	76.62
**4571**	Exposure to secondhand smoke at home	Kerman	Narmashir	Female	22.54	1.99	43.32
**4572**	Exposure to secondhand smoke at home	Isfahan	Natanz	Female	8.45	0.00	26.47
**4573**	Exposure to secondhand smoke at home	Isfahan	Nayin	Female	20.58	0.00	43.91
**4574**	Exposure to secondhand smoke at home	Alborz	Nazarabad	Female	22.43	5.60	39.74
**4575**	Exposure to secondhand smoke at home	Ardebil	Neer	Female	29.38	6.38	52.84
**4576**	Exposure to secondhand smoke at home	Khorasan_South	Nehbandan	Female	11.73	0.00	27.65
**4577**	Exposure to secondhand smoke at home	Mazandaran	Neka	Female	26.90	0.32	53.96
**4578**	Exposure to secondhand smoke at home	Fars	Neyriz	Female	42.95	22.97	63.16
**4579**	Exposure to secondhand smoke at home	Khorasan_razavi	Neyshabur	Female	22.00	6.14	37.13
**4580**	Exposure to secondhand smoke at home	Sistan and Balouchestan	Nikshahr	Female	35.54	15.82	56.03
**4581**	Exposure to secondhand smoke at home	Sistan and Balouchestan	Nimruz	Female	20.88	0.00	47.88
**4582**	Exposure to secondhand smoke at home	Mazandaran	Noshahr	Female	22.62	0.00	48.10
**4583**	Exposure to secondhand smoke at home	Mazandaran	Nur	Female	21.92	0.00	46.00
**4584**	Exposure to secondhand smoke at home	Khuzestan	Omidiyeh	Female	35.00	16.10	54.07
**4585**	Exposure to secondhand smoke at home	Azarbayjan_West	Orumiyeh	Female	41.75	22.95	61.42
**4586**	Exposure to secondhand smoke at home	Azarbayjan_West	Oshnaviyeh	Female	65.53	42.20	89.27
**4587**	Exposure to secondhand smoke at home	Azararbayjan_East	Osku	Female	24.14	0.95	46.40
**4588**	Exposure to secondhand smoke at home	Tehran	Pakdasht	Female	15.97	0.00	33.59
**4589**	Exposure to secondhand smoke at home	Tehran	Pardis	Female	14.52	0.00	41.47
**4590**	Exposure to secondhand smoke at home	Ardebil	Parsabad	Female	31.10	2.51	60.03
**4591**	Exposure to secondhand smoke at home	Hormozgan	Parsian (Gavbandi)	Female	29.07	9.98	47.79
**4592**	Exposure to secondhand smoke at home	Fars	Pasargad	Female	53.53	32.26	76.44
**4593**	Exposure to secondhand smoke at home	Kermanshah	Paveh	Female	30.53	8.49	54.00
**4594**	Exposure to secondhand smoke at home	Azarbayjan_West	Piranshahr	Female	54.61	28.21	83.21
**4595**	Exposure to secondhand smoke at home	Tehran	Pishva	Female	19.49	0.00	45.68
**4596**	Exposure to secondhand smoke at home	Azarbayjan_West	Poldasht	Female	30.08	6.37	54.02
**4597**	Exposure to secondhand smoke at home	Lorestan	Poldokhtar	Female	28.42	5.07	51.65
**4598**	Exposure to secondhand smoke at home	Mazandaran	Qaemshahr	Female	17.62	0.00	39.58
**4599**	Exposure to secondhand smoke at home	Tehran	Qarchak	Female	19.71	0.00	49.78
**4600**	Exposure to secondhand smoke at home	Sistan and Balouchestan	Qasr qand	Female	36.05	6.95	65.41
**4601**	Exposure to secondhand smoke at home	Kermanshah	Qasr-e-Shirin	Female	22.37	0.00	51.59

**4602**	Exposure to secondhand smoke at home	Khorasan_South	Qayenat	Female	21.52	3.61	39.16
**4603**	Exposure to secondhand smoke at home	Qazvin	Qazvin	Female	43.13	21.99	64.47
**4604**	Exposure to secondhand smoke at home	Hormozgan	Qeshm	Female	31.66	5.97	56.66
**4605**	Exposure to secondhand smoke at home	Fars	Qirokarzin	Female	45.73	25.08	66.67
**4606**	Exposure to secondhand smoke at home	Qom	Qom	Female	27.13	0.21	53.82
**4607**	Exposure to secondhand smoke at home	Kordestan	Qorveh	Female	21.51	2.66	41.91
**4608**	Exposure to secondhand smoke at home	Khorasan_razavi	Quchan	Female	36.70	15.66	58.89
**4609**	Exposure to secondhand smoke at home	Kerman	Rabar	Female	29.96	5.19	54.52
**4610**	Exposure to secondhand smoke at home	Kerman	Rafsanjan	Female	33.10	13.96	53.90
**4611**	Exposure to secondhand smoke at home	Khuzestan	Ramhormoz	Female	17.90	2.12	35.04
**4612**	Exposure to secondhand smoke at home	Mazandaran	Ramsar	Female	23.33	0.00	47.40
**4613**	Exposure to secondhand smoke at home	Khuzestan	Ramshir	Female	49.47	30.94	66.16
**4614**	Exposure to secondhand smoke at home	Golestan	Ramyan	Female	17.20	0.00	37.97
**4615**	Exposure to secondhand smoke at home	Gilan	Rasht	Female	10.75	0.00	25.04
**4616**	Exposure to secondhand smoke at home	Khorasan_razavi	Rashtkhar	Female	21.49	1.01	41.54
**4617**	Exposure to secondhand smoke at home	Kermanshah	Ravansar	Female	27.95	5.23	50.55
**4618**	Exposure to secondhand smoke at home	Kerman	Ravar	Female	19.46	5.67	33.70
**4619**	Exposure to secondhand smoke at home	Khorasan_North	Raz va Jergolan	Female	23.58	0.00	56.34
**4620**	Exposure to secondhand smoke at home	Hamedan	Razan	Female	47.57	23.60	72.85
**4621**	Exposure to secondhand smoke at home	Tehran	Rey	Female	25.23	6.41	45.73
**4622**	Exposure to secondhand smoke at home	Kerman	Reygan	Female	34.24	13.88	56.97
**4623**	Exposure to secondhand smoke at home	Gilan	Rezvanshahr	Female	20.22	0.00	43.89
**4624**	Exposure to secondhand smoke at home	Tehran	Robatkarim	Female	7.39	0.00	24.51
**4625**	Exposure to secondhand smoke at home	Fars	Rostam	Female	50.32	28.05	74.18
**4626**	Exposure to secondhand smoke at home	Kerman	Roudbar-e-Jonub	Female	31.51	10.27	53.66
**4627**	Exposure to secondhand smoke at home	Hormozgan	Rudan	Female	30.50	10.34	51.13
**4628**	Exposure to secondhand smoke at home	Gilan	Rudbar	Female	23.82	7.00	41.69
**4629**	Exposure to secondhand smoke at home	Gilan	Rudsar	Female	8.62	0.00	23.39
**4630**	Exposure to secondhand smoke at home	Lorestan	Rumshekan	Female	24.37	0.00	53.47
**4631**	Exposure to secondhand smoke at home	Khorasan_razavi	Sabzevar	Female	16.77	0.72	33.18
**4632**	Exposure to secondhand smoke at home	Yazd	Sadugh	Female	33.93	13.34	55.02
**4633**	Exposure to secondhand smoke at home	Kermanshah	Sahneh	Female	34.33	15.90	53.69
**4634**	Exposure to secondhand smoke at home	Kermanshah	Salas-e-Babajani	Female	23.24	3.89	42.76
**4635**	Exposure to secondhand smoke at home	Azarbayjan_West	Salmas	Female	32.84	10.97	54.16
**4636**	Exposure to secondhand smoke at home	Chaharmahal	Saman	Female	28.77	0.52	56.09
**4637**	Exposure to secondhand smoke at home	Kordestan	Sanandaj	Female	37.91	17.39	58.53
**4638**	Exposure to secondhand smoke at home	Kordestan	Saqqez	Female	56.19	34.03	79.23
**4639**	Exposure to secondhand smoke at home	Kermanshah	Sar-e-Pol-e-Zohab	Female	18.77	5.71	33.26
**4640**	Exposure to secondhand smoke at home	Azararbayjan_East	Sarab	Female	26.97	4.62	49.74
**4641**	Exposure to secondhand smoke at home	Khorasan_razavi	Sarakhs	Female	28.19	3.15	54.02
**4642**	Exposure to secondhand smoke at home	Sistan and Balouchestan	Saravan	Female	38.88	7.13	72.93
**4643**	Exposure to secondhand smoke at home	Khorasan_South	Sarayan	Female	17.56	0.00	38.65
**4644**	Exposure to secondhand smoke at home	Sistan and Balouchestan	Sarbaz	Female	36.36	14.38	58.48
**4645**	Exposure to secondhand smoke at home	Khorasan_South	Sarbisheh	Female	20.03	0.00	42.74
**4646**	Exposure to secondhand smoke at home	Azarbayjan_West	Sardasht	Female	50.99	25.01	78.12
**4647**	Exposure to secondhand smoke at home	Ardebil	Sarein	Female	37.21	9.93	65.23
**4648**	Exposure to secondhand smoke at home	Mazandaran	Sari	Female	19.91	0.00	39.32
**4649**	Exposure to secondhand smoke at home	Kordestan	Sarvabad	Female	41.59	12.50	72.04
**4650**	Exposure to secondhand smoke at home	Fars	Sarvestan	Female	44.09	19.74	68.24
**4651**	Exposure to secondhand smoke at home	Mazandaran	Savadkuh	Female	22.94	0.00	52.33
**4652**	Exposure to secondhand smoke at home	Mazandaran	Savadkuh_North	Female	21.29	0.00	54.83
**4653**	Exposure to secondhand smoke at home	Markazi	Saveh	Female	27.50	9.45	44.86
**4654**	Exposure to secondhand smoke at home	Alborz	Savojbolagh	Female	22.60	6.35	39.19
**4655**	Exposure to secondhand smoke at home	Lorestan	Selseleh	Female	35.08	10.80	59.24
**4656**	Exposure to secondhand smoke at home	Isfahan	Semirom	Female	38.43	18.07	59.28
**4657**	Exposure to secondhand smoke at home	Isfahan	Semirom-e-Sofla	Female	32.78	5.40	60.85
**4658**	Exposure to secondhand smoke at home	Semnan	Semnan	Female	12.48	0.00	28.14
**4659**	Exposure to secondhand smoke at home	Fars	Sepidan	Female	51.65	32.29	71.81
**4660**	Exposure to secondhand smoke at home	Azararbayjan_East	Shabestar	Female	8.31	0.00	26.15
**4661**	Exposure to secondhand smoke at home	Khuzestan	Shadegan	Female	31.92	10.81	54.21
**4662**	Exposure to secondhand smoke at home	Gilan	Shaft	Female	22.64	5.44	40.82
**4663**	Exposure to secondhand smoke at home	Azarbayjan_West	Shahindezh	Female	48.94	26.86	73.03
**4664**	Exposure to secondhand smoke at home	Tehran	Shahr-e Qods	Female	13.21	0.00	30.55
**4665**	Exposure to secondhand smoke at home	Kerman	Shahr-e-Babak	Female	32.27	10.46	53.70
**4666**	Exposure to secondhand smoke at home	Chaharmahal	Shahr-e-Kord	Female	25.05	7.89	42.98
**4667**	Exposure to secondhand smoke at home	Isfahan	Shahreza	Female	35.15	14.35	56.33
**4668**	Exposure to secondhand smoke at home	Tehran	Shahriyar	Female	18.76	2.61	34.87
**4669**	Exposure to secondhand smoke at home	Semnan	Shahrud	Female	15.68	2.01	29.25
**4670**	Exposure to secondhand smoke at home	Markazi	Shazand	Female	38.96	17.75	60.88
**4671**	Exposure to secondhand smoke at home	Tehran	Shemiranat	Female	6.33	0.00	22.50
**4672**	Exposure to secondhand smoke at home	Fars	Shiraz	Female	39.92	25.14	55.02
**4673**	Exposure to secondhand smoke at home	Khorasan_North	Shirvan	Female	18.13	0.00	36.79
**4674**	Exposure to secondhand smoke at home	Ilam	Shirvan and Chard-e-Aval	Female	28.87	7.45	50.67
**4675**	Exposure to secondhand smoke at home	Azarbayjan_West	Showt	Female	24.22	0.03	48.36
**4676**	Exposure to secondhand smoke at home	Khuzestan	Shush	Female	47.28	28.41	66.46
**4677**	Exposure to secondhand smoke at home	Khuzestan	Shushtar	Female	30.09	13.31	47.06
**4678**	Exposure to secondhand smoke at home	Gilan	Siahkal	Female	17.29	0.00	38.57
**4679**	Exposure to secondhand smoke at home	Sistan and Balouchestan	Sib o Soran	Female	38.87	10.24	67.93
**4680**	Exposure to secondhand smoke at home	Mazandaran	Simorgh	Female	19.74	0.00	52.75
**4681**	Exposure to secondhand smoke at home	Hormozgan	Sirik	Female	35.91	10.15	62.47
**4682**	Exposure to secondhand smoke at home	Kerman	Sirjan	Female	30.57	12.72	47.86
**4683**	Exposure to secondhand smoke at home	Ilam	Sirvan	Female	25.74	0.55	50.65
**4684**	Exposure to secondhand smoke at home	Zanjan	Soltaniyeh	Female	37.82	14.38	61.94
**4685**	Exposure to secondhand smoke at home	Kermanshah	Sonqor	Female	26.15	9.48	42.41
**4686**	Exposure to secondhand smoke at home	Semnan	Sorkheh	Female	16.11	0.00	37.97
**4687**	Exposure to secondhand smoke at home	Gilan	Sume'eh Sara	Female	13.78	0.00	30.14
**4688**	Exposure to secondhand smoke at home	Khorasan_South	Tabas	Female	15.83	0.00	31.15
**4689**	Exposure to secondhand smoke at home	Azararbayjan_East	Tabriz	Female	23.88	8.30	39.61
**4690**	Exposure to secondhand smoke at home	Markazi	Tafresh	Female	15.23	0.00	34.33
**4691**	Exposure to secondhand smoke at home	Yazd	Taft	Female	31.19	11.61	50.13
**4692**	Exposure to secondhand smoke at home	Azarbayjan_West	Takab	Female	48.94	25.55	73.62
**4693**	Exposure to secondhand smoke at home	Qazvin	Takestan	Female	42.70	13.52	73.66
**4694**	Exposure to secondhand smoke at home	Khorasan_razavi	Takht-e-Jolgeh (Firuzeh)	Female	19.31	0.00	40.22
**4695**	Exposure to secondhand smoke at home	Alborz	Taleghan	Female	33.56	18.28	49.81
**4696**	Exposure to secondhand smoke at home	Boushehr	Tangestan	Female	31.94	12.34	51.75
**4697**	Exposure to secondhand smoke at home	Zanjan	Tarom	Female	32.62	10.69	54.52
**4698**	Exposure to secondhand smoke at home	Gilan	Tavalesh	Female	24.40	2.94	45.90
**4699**	Exposure to secondhand smoke at home	Khorasan_razavi	Taybad	Female	51.35	29.23	73.48

**4700**	Exposure to secondhand smoke at home	Tehran	Tehran	Female	14.97	1.97	28.73
**4701**	Exposure to secondhand smoke at home	Isfahan	Tiran and Karvan	Female	22.00	1.77	41.30
**4702**	Exposure to secondhand smoke at home	Mazandaran	Tonekabon	Female	26.55	2.80	49.28
**4703**	Exposure to secondhand smoke at home	Khorasan_razavi	Torbat-e-Heydariyeh	Female	22.35	5.48	39.33
**4704**	Exposure to secondhand smoke at home	Khorasan_razavi	Torbat-e-Jam	Female	34.06	14.73	54.00
**4705**	Exposure to secondhand smoke at home	Hamedan	Tuyserkan	Female	42.08	17.58	66.69
**4706**	Exposure to secondhand smoke at home	Tehran	Varamin	Female	23.13	5.04	41.53
**4707**	Exposure to secondhand smoke at home	Azararbayjan_East	Varzaqan	Female	15.40	0.00	33.11
**4708**	Exposure to secondhand smoke at home	Yazd	Yazd	Female	26.72	11.93	42.43
**4709**	Exposure to secondhand smoke at home	Sistan and Balouchestan	Zabol	Female	16.60	0.00	37.81
**4710**	Exposure to secondhand smoke at home	Sistan and Balouchestan	Zaboli (Mehrestan)	Female	34.45	5.81	64.04
**4711**	Exposure to secondhand smoke at home	Sistan and Balouchestan	Zahedan	Female	23.67	7.09	40.09
**4712**	Exposure to secondhand smoke at home	Zanjan	Zanjan	Female	35.45	18.52	52.74
**4713**	Exposure to secondhand smoke at home	Kerman	Zarand	Female	24.16	2.12	46.57
**4714**	Exposure to secondhand smoke at home	Markazi	Zarandiyeh	Female	26.63	12.01	41.42
**4715**	Exposure to secondhand smoke at home	Fars	Zarrindasht	Female	35.02	15.69	54.17
**4716**	Exposure to secondhand smoke at home	Khorasan_razavi	Zave	Female	21.92	5.23	39.06
**4717**	Exposure to secondhand smoke at home	Sistan and Balouchestan	Zehak	Female	19.49	0.00	52.99
**4718**	Exposure to secondhand smoke at home	Khorasan_South	Zir kuh	Female	20.29	0.00	46.29
**4719**	Exposure to secondhand smoke at home	Khuzestan	Abadan	Male	24.33	2.77	45.89
**4720**	Exposure to secondhand smoke at home	Fars	Abadeh	Male	47.25	26.53	71.13
**4721**	Exposure to secondhand smoke at home	Yazd	Abarkuh	Male	19.18	4.10	33.37
**4722**	Exposure to secondhand smoke at home	Mazandaran	Abbas abad	Male	32.94	15.31	49.17
**4723**	Exposure to secondhand smoke at home	Ilam	Abdanan	Male	25.33	5.31	46.65
**4724**	Exposure to secondhand smoke at home	Zanjan	Abhar	Male	33.71	11.92	55.49
**4725**	Exposure to secondhand smoke at home	Hormozgan	Abumusa	Male	20.71	0.00	48.47
**4726**	Exposure to secondhand smoke at home	Qazvin	Abyek	Male	40.82	14.23	69.31
**4727**	Exposure to secondhand smoke at home	Azararbayjan_East	Ahar	Male	18.63	0.00	39.70
**4728**	Exposure to secondhand smoke at home	Khuzestan	Ahvaz	Male	33.99	17.90	49.80
**4729**	Exposure to secondhand smoke at home	Azararbayjan_East	Ajabshir	Male	27.95	0.00	56.73
**4730**	Exposure to secondhand smoke at home	Qazvin	Alborz	Male	35.42	8.95	61.83
**4731**	Exposure to secondhand smoke at home	Golestan	Aliabad	Male	27.25	3.56	52.62
**4732**	Exposure to secondhand smoke at home	Lorestan	Aligudarz	Male	33.00	10.16	55.68
**4733**	Exposure to secondhand smoke at home	Gilan	Amlash	Male	16.66	2.35	30.97
**4734**	Exposure to secondhand smoke at home	Mazandaran	Amol	Male	20.07	0.76	38.99
**4735**	Exposure to secondhand smoke at home	Kerman	Anar	Male	29.69	0.00	61.91
**4736**	Exposure to secondhand smoke at home	Kerman	Anbarabad	Male	37.67	11.41	64.87
**4737**	Exposure to secondhand smoke at home	Khuzestan	Andika	Male	39.20	16.98	62.54
**4738**	Exposure to secondhand smoke at home	Khuzestan	Andimeshk	Male	30.40	8.37	51.53
**4739**	Exposure to secondhand smoke at home	Golestan	Aq Qala	Male	16.16	0.00	36.46
**4740**	Exposure to secondhand smoke at home	Khuzestan	Aqajari	Male	39.39	11.68	66.84
**4741**	Exposure to secondhand smoke at home	Semnan	Aradan	Male	17.60	0.00	40.31
**4742**	Exposure to secondhand smoke at home	Markazi	Arak	Male	36.96	13.69	59.44
**4743**	Exposure to secondhand smoke at home	Isfahan	Aran and Bidgol	Male	14.94	0.00	32.48
**4744**	Exposure to secondhand smoke at home	Ardebil	Ardabil	Male	36.94	16.59	57.08
**4745**	Exposure to secondhand smoke at home	Yazd	Ardakan	Male	22.10	7.31	37.71
**4746**	Exposure to secondhand smoke at home	Chaharmahal	Ardal	Male	39.24	14.48	64.46
**4747**	Exposure to secondhand smoke at home	Isfahan	Ardestan	Male	21.85	0.00	45.26
**4748**	Exposure to secondhand smoke at home	Fars	Arsanjan	Male	38.10	15.72	61.69
**4749**	Exposure to secondhand smoke at home	Kerman	Arzouyeh	Male	46.23	26.42	64.71
**4750**	Exposure to secondhand smoke at home	Hamedan	Asadabad	Male	37.26	12.47	62.55
**4751**	Exposure to secondhand smoke at home	Boushehr	Asaluyeh	Male	34.20	7.86	60.59
**4752**	Exposure to secondhand smoke at home	Markazi	Ashtiyan	Male	34.00	2.42	65.26
**4753**	Exposure to secondhand smoke at home	Gilan	Astaneh-ye-Ashrafiyeh	Male	20.67	0.00	43.48
**4754**	Exposure to secondhand smoke at home	Gilan	Astara	Male	18.74	0.00	39.00
**4755**	Exposure to secondhand smoke at home	Qazvin	Avaj	Male	39.26	10.90	68.53
**4756**	Exposure to secondhand smoke at home	Golestan	Azadshahr	Male	22.74	0.00	49.90
**4757**	Exposure to secondhand smoke at home	Azararbayjan_East	Azarshahr	Male	22.41	0.00	48.41
**4758**	Exposure to secondhand smoke at home	Lorestan	Azna	Male	21.42	0.16	43.28
**4759**	Exposure to secondhand smoke at home	Mazandaran	Babol	Male	16.44	0.00	33.14
**4760**	Exposure to secondhand smoke at home	Mazandaran	Babolsar	Male	17.49	0.00	35.24
**4761**	Exposure to secondhand smoke at home	Ilam	Badreh	Male	36.29	6.10	65.67
**4762**	Exposure to secondhand smoke at home	Yazd	Bafq	Male	16.68	0.46	31.16
**4763**	Exposure to secondhand smoke at home	Kerman	Baft	Male	34.42	10.49	56.04
**4764**	Exposure to secondhand smoke at home	Khuzestan	Baghemalek	Male	32.68	11.27	52.84
**4765**	Exposure to secondhand smoke at home	Yazd	Bahabad	Male	18.76	0.00	38.66
**4766**	Exposure to secondhand smoke at home	Hamedan	Bahar	Male	38.77	13.99	63.87
**4767**	Exposure to secondhand smoke at home	Tehran	Baharestan (Golestan)	Male	22.08	3.60	41.45
**4768**	Exposure to secondhand smoke at home	Kohkiluye and Bouyer Ahmad	Bahmani	Male	57.47	35.83	77.79
**4769**	Exposure to secondhand smoke at home	Khorasan_razavi	Bajestan	Male	21.33	0.00	45.48
**4770**	Exposure to secondhand smoke at home	Khorasan_razavi	Bakhraz	Male	24.12	1.74	46.48
**4771**	Exposure to secondhand smoke at home	Kerman	Bam	Male	36.17	14.31	58.31
**4772**	Exposure to secondhand smoke at home	Hormozgan	Bandar-e-Abbas	Male	17.89	2.68	33.33
**4773**	Exposure to secondhand smoke at home	Gilan	Bandar-e-Anzali	Male	12.57	0.00	29.81
**4774**	Exposure to secondhand smoke at home	Golestan	Bandar-e-Gaz	Male	32.97	11.64	51.27
**4775**	Exposure to secondhand smoke at home	Hormozgan	Bandar-e-Jask	Male	28.42	6.02	50.56
**4776**	Exposure to secondhand smoke at home	Hormozgan	Bandar-e-Lengeh	Male	16.27	0.00	33.00
**4777**	Exposure to secondhand smoke at home	Khuzestan	Bandar-e-Mahshahr	Male	31.77	12.46	50.60
**4778**	Exposure to secondhand smoke at home	Golestan	Bandar-e-Torkaman	Male	17.85	0.00	39.35
**4779**	Exposure to secondhand smoke at home	Kordestan	Baneh	Male	47.79	20.26	76.10
**4780**	Exposure to secondhand smoke at home	Khorasan_razavi	Bardeskan	Male	20.76	0.00	41.61
**4781**	Exposure to secondhand smoke at home	Kerman	Bardsir	Male	35.78	11.27	61.20
**4782**	Exposure to secondhand smoke at home	Hormozgan	Bashagerd	Male	28.49	7.92	49.70
**4783**	Exposure to secondhand smoke at home	Kohkiluye and Bouyer Ahmad	Basht	Male	74.63	47.15	100.00
**4784**	Exposure to secondhand smoke at home	Hormozgan	Bastak	Male	24.69	2.09	47.43
**4785**	Exposure to secondhand smoke at home	Khuzestan	Bavi	Male	35.65	13.22	57.92
**4786**	Exposure to secondhand smoke at home	Khuzestan	Behbahan	Male	38.85	16.49	61.35
**4787**	Exposure to secondhand smoke at home	Mazandaran	Behshahr	Male	18.25	0.00	44.94
**4788**	Exposure to secondhand smoke at home	Kordestan	Bijar	Male	41.70	16.98	65.66
**4789**	Exposure to secondhand smoke at home	Ardebil	Bilehsavar	Male	27.05	0.00	63.64
**4790**	Exposure to secondhand smoke at home	Khorasan_razavi	Binaloud	Male	25.52	1.58	49.97
**4791**	Exposure to secondhand smoke at home	Khorasan_South	Birjand	Male	20.50	1.95	39.12
**4792**	Exposure to secondhand smoke at home	Khorasan_North	Bojnurd	Male	23.12	4.42	42.83
**4793**	Exposure to secondhand smoke at home	Chaharmahal	Bon	Male	33.97	3.56	65.77
**4794**	Exposure to secondhand smoke at home	Azararbayjan_East	Bonab	Male	28.93	5.35	51.58
**4795**	Exposure to secondhand smoke at home	Isfahan	Borkhar	Male	20.96	0.00	40.93
**4796**	Exposure to secondhand smoke at home	Isfahan	Borkhar and Meymeh	Male	28.62	7.96	49.97
**4797**	Exposure to secondhand smoke at home	Chaharmahal	Borujen	Male	22.93	2.28	42.93

**4798**	Exposure to secondhand smoke at home	Lorestan	Borujerd	Male	38.28	16.79	61.03
**4799**	Exposure to secondhand smoke at home	Khorasan_South	Boshruyeh	Male	18.43	0.00	43.40
**4800**	Exposure to secondhand smoke at home	Azararbayjan_East	Bostanabad	Male	30.26	8.03	54.38
**4801**	Exposure to secondhand smoke at home	Fars	Bovanat	Male	35.99	14.82	56.52
**4802**	Exposure to secondhand smoke at home	Kohkiluye and Bouyer Ahmad	Boyer Ahmad	Male	53.24	30.53	75.91
**4803**	Exposure to secondhand smoke at home	Qazvin	Boyinzahra	Male	46.54	23.65	71.78
**4804**	Exposure to secondhand smoke at home	Isfahan	Buein va Miandasht	Male	30.51	5.69	55.70
**4805**	Exposure to secondhand smoke at home	Azarbayjan_West	Bukan	Male	46.62	24.75	69.57
**4806**	Exposure to secondhand smoke at home	Boushehr	Bushehr	Male	31.66	10.08	52.06
**4807**	Exposure to secondhand smoke at home	Isfahan	Chadegan	Male	38.22	17.73	62.17
**4808**	Exposure to secondhand smoke at home	Sistan and Balouchestan	Chahbahar	Male	27.84	7.74	48.23
**4809**	Exposure to secondhand smoke at home	Azarbayjan_West	Chaipareh	Male	18.68	0.00	46.51
**4810**	Exposure to secondhand smoke at home	Azarbayjan_West	Chaldoran	Male	17.45	0.00	47.35
**4811**	Exposure to secondhand smoke at home	Mazandaran	Chalus	Male	24.77	3.28	46.08
**4812**	Exposure to secondhand smoke at home	Azararbayjan_East	Charoimaq	Male	34.50	9.75	59.06
**4813**	Exposure to secondhand smoke at home	Khorasan_razavi	Chenaran	Male	28.56	7.13	51.96
**4814**	Exposure to secondhand smoke at home	Kohkiluye and Bouyer Ahmad	Cheram	Male	53.45	23.21	82.40
**4815**	Exposure to secondhand smoke at home	Kermanshah	Dalaho	Male	29.28	4.50	52.71
**4816**	Exposure to secondhand smoke at home	Lorestan	Dalfan	Male	44.74	22.26	70.79
**4817**	Exposure to secondhand smoke at home	Sistan and Balouchestan	Dalgan	Male	28.16	5.57	50.95
**4818**	Exposure to secondhand smoke at home	Tehran	Damavand	Male	18.16	0.00	39.24
**4819**	Exposure to secondhand smoke at home	Semnan	Damghan	Male	16.88	0.00	34.20
**4820**	Exposure to secondhand smoke at home	Fars	Darab	Male	48.13	27.89	70.90
**4821**	Exposure to secondhand smoke at home	Khorasan_South	Darmian	Male	23.69	1.52	45.99
**4822**	Exposure to secondhand smoke at home	Khorasan_razavi	Darrehgaz	Male	17.84	0.00	36.92
**4823**	Exposure to secondhand smoke at home	Ilam	Darrehshahr	Male	32.95	7.56	57.91
**4824**	Exposure to secondhand smoke at home	Khuzestan	Dasht-e-Azadegan	Male	38.85	13.86	65.03
**4825**	Exposure to secondhand smoke at home	Boushehr	Dashtestan	Male	36.93	17.00	57.95
**4826**	Exposure to secondhand smoke at home	Boushehr	Dashti	Male	30.73	10.17	49.85
**4827**	Exposure to secondhand smoke at home	Khorasan_razavi	Davarzan	Male	21.07	0.00	46.95
**4828**	Exposure to secondhand smoke at home	Boushehr	Dayyer	Male	54.76	32.29	73.87
**4829**	Exposure to secondhand smoke at home	Kordestan	Dehgolan	Male	52.20	29.28	78.69
**4830**	Exposure to secondhand smoke at home	Ilam	Dehloran	Male	36.17	10.13	62.86
**4831**	Exposure to secondhand smoke at home	Markazi	Delijan	Male	31.09	3.38	58.12
**4832**	Exposure to secondhand smoke at home	Kohkiluye and Bouyer Ahmad	Dena	Male	51.21	20.99	81.51
**4833**	Exposure to secondhand smoke at home	Boushehr	Deylam	Male	38.59	11.84	65.64
**4834**	Exposure to secondhand smoke at home	Khuzestan	Dezful	Male	31.50	13.37	49.17
**4835**	Exposure to secondhand smoke at home	Kordestan	Divandarreh	Male	49.08	24.18	76.06
**4836**	Exposure to secondhand smoke at home	Lorestan	Dorud	Male	35.70	9.69	62.52
**4837**	Exposure to secondhand smoke at home	Lorestan	Doureh	Male	34.87	18.05	51.72
**4838**	Exposure to secondhand smoke at home	Fars	Eqlid	Male	37.26	14.92	59.59
**4839**	Exposure to secondhand smoke at home	Khorasan_North	Esfarayen	Male	30.62	10.19	52.40
**4840**	Exposure to secondhand smoke at home	Alborz	Eshtehard	Male	24.32	4.04	44.58
**4841**	Exposure to secondhand smoke at home	Kermanshah	Eslamabad-e-Gharb	Male	24.15	4.25	43.07
**4842**	Exposure to secondhand smoke at home	Tehran	Eslamshahr	Male	19.45	1.11	37.90
**4843**	Exposure to secondhand smoke at home	Fars	Estahban	Male	35.41	11.62	57.76
**4844**	Exposure to secondhand smoke at home	Ilam	Eyvan	Male	45.79	18.54	75.61
**4845**	Exposure to secondhand smoke at home	Kerman	Fahraj	Male	33.05	6.86	59.32
**4846**	Exposure to secondhand smoke at home	Isfahan	Falavarjan	Male	36.00	15.15	58.72
**4847**	Exposure to secondhand smoke at home	Hamedan	Famenin	Male	35.98	7.72	64.85
**4848**	Exposure to secondhand smoke at home	Markazi	Farahan	Male	35.88	3.65	68.14
**4849**	Exposure to secondhand smoke at home	Fars	Farashband	Male	39.47	15.13	63.24
**4850**	Exposure to secondhand smoke at home	Alborz	Fardis	Male	21.23	0.00	45.21
**4851**	Exposure to secondhand smoke at home	Isfahan	Faridan	Male	33.45	12.64	56.17
**4852**	Exposure to secondhand smoke at home	Khorasan_razavi	Fariman	Male	26.02	10.42	41.44
**4853**	Exposure to secondhand smoke at home	Khorasan_North	Faroj	Male	36.72	14.51	61.00
**4854**	Exposure to secondhand smoke at home	Chaharmahal	Farsan	Male	34.19	9.98	58.33
**4855**	Exposure to secondhand smoke at home	Kerman	Faryab	Male	35.76	7.57	65.35
**4856**	Exposure to secondhand smoke at home	Fars	Fasa	Male	48.13	27.97	70.71
**4857**	Exposure to secondhand smoke at home	Khorasan_South	Ferdows	Male	10.91	0.00	28.93
**4858**	Exposure to secondhand smoke at home	Mazandaran	Fereydunkenar	Male	17.98	0.00	44.49
**4859**	Exposure to secondhand smoke at home	Isfahan	Fereydunshahr	Male	33.06	11.66	56.23
**4860**	Exposure to secondhand smoke at home	Fars	Firozabad	Male	45.64	25.76	68.91
**4861**	Exposure to secondhand smoke at home	Tehran	Firuzkuh	Male	8.19	0.00	24.71
**4862**	Exposure to secondhand smoke at home	Sistan and Balouchestan	Fonuj	Male	29.16	5.28	53.56
**4863**	Exposure to secondhand smoke at home	Gilan	Fuman	Male	17.25	0.18	34.25
**4864**	Exposure to secondhand smoke at home	Kohkiluye and Bouyer Ahmad	Gachsaran	Male	67.05	42.20	94.71
**4865**	Exposure to secondhand smoke at home	Golestan	Galikesh	Male	22.51	0.00	47.30
**4866**	Exposure to secondhand smoke at home	Mazandaran	Galugah	Male	19.39	0.00	49.16
**4867**	Exposure to secondhand smoke at home	Semnan	Garmsar	Male	18.57	1.02	36.64
**4868**	Exposure to secondhand smoke at home	Boushehr	Genaveh	Male	36.75	16.80	57.49
**4869**	Exposure to secondhand smoke at home	Fars	Gerash	Male	43.16	15.46	71.39
**4870**	Exposure to secondhand smoke at home	Khorasan_North	Germeh	Male	22.39	0.00	50.77
**4871**	Exposure to secondhand smoke at home	Ardebil	Germi	Male	29.71	4.02	57.56
**4872**	Exposure to secondhand smoke at home	Kerman	Ghaleye-Ganj	Male	38.46	13.48	63.92
**4873**	Exposure to secondhand smoke at home	Kermanshah	Gilan-e-Gharb	Male	29.72	9.24	50.05
**4874**	Exposure to secondhand smoke at home	Isfahan	Golpayegan	Male	33.60	12.82	56.47
**4875**	Exposure to secondhand smoke at home	Golestan	Gomishan	Male	12.14	0.00	34.50
**4876**	Exposure to secondhand smoke at home	Khorasan_razavi	Gonabad	Male	21.85	0.00	45.97
**4877**	Exposure to secondhand smoke at home	Golestan	Gonbad-e-Kavus	Male	23.35	1.63	45.15
**4878**	Exposure to secondhand smoke at home	Golestan	Gorgan	Male	19.09	0.00	38.52
**4879**	Exposure to secondhand smoke at home	Khuzestan	Guotvand	Male	27.05	4.91	47.45
**4880**	Exposure to secondhand smoke at home	Khuzestan	Haftgol	Male	35.36	11.80	57.81
**4881**	Exposure to secondhand smoke at home	Hormozgan	Hajiabad	Male	24.94	7.04	44.87
**4882**	Exposure to secondhand smoke at home	Hamedan	Hamadan	Male	36.63	16.37	55.93
**4883**	Exposure to secondhand smoke at home	Khuzestan	Hamidiyeh	Male	36.78	9.12	63.98
**4884**	Exposure to secondhand smoke at home	Sistan and Balouchestan	Hamoon	Male	16.33	0.00	40.54
**4885**	Exposure to secondhand smoke at home	Azararbayjan_East	Haris	Male	23.87	3.05	45.01
**4886**	Exposure to secondhand smoke at home	Kermanshah	Harsin	Male	48.09	26.31	71.46
**4887**	Exposure to secondhand smoke at home	Azararbayjan_East	Hashtrud	Male	19.38	0.00	39.80
**4888**	Exposure to secondhand smoke at home	Khuzestan	Hendijan	Male	38.40	11.55	64.30
**4889**	Exposure to secondhand smoke at home	Sistan and Balouchestan	Hirmand	Male	7.71	0.00	25.48
**4890**	Exposure to secondhand smoke at home	Khuzestan	Hoveizeh	Male	36.75	8.10	66.06
**4891**	Exposure to secondhand smoke at home	Zanjan	Ijerod	Male	36.77	16.28	56.90
**4892**	Exposure to secondhand smoke at home	Ilam	Ilam	Male	39.86	15.83	65.17
**4893**	Exposure to secondhand smoke at home	Sistan and Balouchestan	Iranshahr	Male	31.59	12.77	51.24
**4894**	Exposure to secondhand smoke at home	Isfahan	Isfahan	Male	22.79	7.37	38.43
**4895**	Exposure to secondhand smoke at home	Khuzestan	Izeh	Male	37.60	17.17	57.87

**4896**	Exposure to secondhand smoke at home	Fars	Jahrom	Male	39.55	19.51	59.76
**4897**	Exposure to secondhand smoke at home	Khorasan_North	Jajarm	Male	23.65	0.50	46.45
**4898**	Exposure to secondhand smoke at home	Boushehr	Jam	Male	20.85	1.30	42.01
**4899**	Exposure to secondhand smoke at home	Kermanshah	Javanrud	Male	31.62	7.74	55.08
**4900**	Exposure to secondhand smoke at home	Kerman	Jiroft	Male	33.29	10.68	54.38
**4901**	Exposure to secondhand smoke at home	Khorasan_razavi	Joghatai	Male	21.82	0.00	47.90
**4902**	Exposure to secondhand smoke at home	Azararbayjan_East	Jolfa	Male	12.43	0.00	32.13
**4903**	Exposure to secondhand smoke at home	Khorasan_razavi	Jowayin	Male	23.40	0.15	46.85
**4904**	Exposure to secondhand smoke at home	Mazandaran	Juybar	Male	11.68	0.00	30.10
**4905**	Exposure to secondhand smoke at home	Hamedan	Kabudarahang	Male	40.28	15.91	65.52
**4906**	Exposure to secondhand smoke at home	Kerman	Kahnuj	Male	31.76	7.96	55.04
**4907**	Exposure to secondhand smoke at home	Golestan	Kalaleh	Male	29.86	6.07	55.34
**4908**	Exposure to secondhand smoke at home	Khorasan_razavi	Kalat	Male	24.75	0.00	52.52
**4909**	Exposure to secondhand smoke at home	Azararbayjan_East	Kaleibar	Male	19.40	0.00	46.95
**4910**	Exposure to secondhand smoke at home	Kordestan	Kamyaran	Male	44.60	25.40	64.03
**4911**	Exposure to secondhand smoke at home	Boushehr	Kangan	Male	33.31	8.96	57.21
**4912**	Exposure to secondhand smoke at home	Kermanshah	Kangavar	Male	28.02	8.42	46.88
**4913**	Exposure to secondhand smoke at home	Alborz	Karaj	Male	18.31	4.91	31.96
**4914**	Exposure to secondhand smoke at home	Khuzestan	Karun	Male	34.14	4.16	64.71
**4915**	Exposure to secondhand smoke at home	Isfahan	Kashan	Male	18.74	1.02	35.87
**4916**	Exposure to secondhand smoke at home	Khorasan_razavi	Kashmar	Male	22.18	0.89	43.60
**4917**	Exposure to secondhand smoke at home	Fars	Kavar	Male	40.33	18.99	62.40
**4918**	Exposure to secondhand smoke at home	Fars	Kazerun	Male	26.63	6.28	46.26
**4919**	Exposure to secondhand smoke at home	Mazandaran	Kelardasht	Male	27.35	0.99	53.79
**4920**	Exposure to secondhand smoke at home	Kerman	Kerman	Male	32.02	12.94	50.49
**4921**	Exposure to secondhand smoke at home	Kermanshah	Kermanshah	Male	28.55	13.24	43.53
**4922**	Exposure to secondhand smoke at home	Khorasan_razavi	Khaf	Male	26.71	5.85	48.87
**4923**	Exposure to secondhand smoke at home	Khorasan_razavi	Khalilabad	Male	21.05	0.00	43.64
**4924**	Exposure to secondhand smoke at home	Ardebil	Khalkhal	Male	47.81	27.19	67.79
**4925**	Exposure to secondhand smoke at home	Hormozgan	Khamir	Male	23.88	4.77	43.85
**4926**	Exposure to secondhand smoke at home	Isfahan	Khansar	Male	25.69	4.25	46.31
**4927**	Exposure to secondhand smoke at home	Sistan and Balouchestan	Khash	Male	30.25	8.86	51.54
**4928**	Exposure to secondhand smoke at home	Yazd	Khatam	Male	37.79	21.09	53.79
**4929**	Exposure to secondhand smoke at home	Fars	Kherameh	Male	29.83	10.64	47.57
**4930**	Exposure to secondhand smoke at home	Azararbayjan_East	Khodaafarin	Male	17.33	0.00	45.20
**4931**	Exposure to secondhand smoke at home	Zanjan	Khodabandeh	Male	48.28	26.84	71.56
**4932**	Exposure to secondhand smoke at home	Markazi	Khomeyn	Male	45.18	16.79	76.11
**4933**	Exposure to secondhand smoke at home	Isfahan	Khomeynishahr	Male	31.63	11.06	53.02
**4934**	Exposure to secondhand smoke at home	Markazi	Khondab	Male	49.69	21.94	81.06
**4935**	Exposure to secondhand smoke at home	Fars	Khonj	Male	54.75	34.76	72.35
**4936**	Exposure to secondhand smoke at home	Isfahan	Khoor va Biabanak	Male	21.68	0.00	46.94
**4937**	Exposure to secondhand smoke at home	Lorestan	Khorramabad	Male	34.08	13.77	54.75
**4938**	Exposure to secondhand smoke at home	Fars	Khorrambid	Male	38.38	16.18	60.62
**4939**	Exposure to secondhand smoke at home	Zanjan	Khorramdarreh	Male	34.88	8.81	60.53
**4940**	Exposure to secondhand smoke at home	Khuzestan	Khorramshahr	Male	37.01	12.20	62.42
**4941**	Exposure to secondhand smoke at home	Khorasan_razavi	Khoshab	Male	23.33	0.00	47.45
**4942**	Exposure to secondhand smoke at home	Azarbayjan_West	Khoy	Male	18.44	0.00	38.53
**4943**	Exposure to secondhand smoke at home	Khorasan_South	Khusef	Male	20.60	0.00	44.53
**4944**	Exposure to secondhand smoke at home	Chaharmahal	Kiaar	Male	32.18	7.71	56.33
**4945**	Exposure to secondhand smoke at home	Kohkiluye and Bouyer Ahmad	Kohgiluyeh	Male	44.07	18.39	67.16
**4946**	Exposure to secondhand smoke at home	Markazi	Komeijan	Male	38.40	6.70	70.94
**4947**	Exposure to secondhand smoke at home	Sistan and Balouchestan	Konarak	Male	30.83	9.94	52.24
**4948**	Exposure to secondhand smoke at home	Golestan	Kordkuy	Male	19.52	1.35	38.63
**4949**	Exposure to secondhand smoke at home	Ardebil	Kowsar	Male	37.09	10.29	64.07
**4950**	Exposure to secondhand smoke at home	Kerman	Kuhbonan	Male	23.39	5.24	42.69
**4951**	Exposure to secondhand smoke at home	Lorestan	Kuhdasht	Male	33.04	10.45	55.20
**4952**	Exposure to secondhand smoke at home	Chaharmahal	Kuhrang	Male	42.40	20.06	67.59
**4953**	Exposure to secondhand smoke at home	Gilan	Lahijan	Male	21.04	0.00	43.85
**4954**	Exposure to secondhand smoke at home	Khuzestan	Lali	Male	40.15	17.09	64.41
**4955**	Exposure to secondhand smoke at home	Fars	Lamard	Male	56.39	34.30	81.11
**4956**	Exposure to secondhand smoke at home	Kohkiluye and Bouyer Ahmad	Landeh	Male	51.56	13.79	90.47
**4957**	Exposure to secondhand smoke at home	Gilan	Langrud	Male	12.57	0.00	28.37
**4958**	Exposure to secondhand smoke at home	Isfahan	Lanjan	Male	25.84	7.75	43.38
**4959**	Exposure to secondhand smoke at home	Fars	Lar (Larestan)	Male	39.89	20.00	60.77
**4960**	Exposure to secondhand smoke at home	Chaharmahal	Lordakan	Male	44.59	23.83	66.17
**4961**	Exposure to secondhand smoke at home	Azarbayjan_West	Mahabad	Male	43.53	22.74	65.45
**4962**	Exposure to secondhand smoke at home	Markazi	Mahalat	Male	37.43	6.90	67.96
**4963**	Exposure to secondhand smoke at home	Mazandaran	Mahmudabad	Male	19.49	0.00	41.57
**4964**	Exposure to secondhand smoke at home	Zanjan	Mahneshan	Male	49.43	26.18	75.34
**4965**	Exposure to secondhand smoke at home	Khorasan_razavi	Mahvelat	Male	21.71	0.00	45.91
**4966**	Exposure to secondhand smoke at home	Azarbayjan_West	Maku	Male	7.60	0.00	29.98
**4967**	Exposure to secondhand smoke at home	Tehran	Malard	Male	23.41	4.64	42.47
**4968**	Exposure to secondhand smoke at home	Hamedan	Malayer	Male	44.51	23.00	67.61
**4969**	Exposure to secondhand smoke at home	Azararbayjan_East	Malekan	Male	41.74	17.18	68.06
**4970**	Exposure to secondhand smoke at home	Ilam	Malekshahi	Male	36.26	3.33	67.57
**4971**	Exposure to secondhand smoke at home	Fars	Mamasany	Male	41.05	20.92	60.49
**4972**	Exposure to secondhand smoke at home	Khorasan_North	Maneh and Samalqan	Male	16.67	0.00	35.60
**4973**	Exposure to secondhand smoke at home	Kerman	Manujan	Male	37.43	7.44	66.65
**4974**	Exposure to secondhand smoke at home	Azararbayjan_East	Maragheh	Male	35.51	15.52	58.14
**4975**	Exposure to secondhand smoke at home	Azararbayjan_East	Marand	Male	11.21	0.00	29.65
**4976**	Exposure to secondhand smoke at home	Golestan	Maravehtapeh	Male	24.91	0.00	54.36
**4977**	Exposure to secondhand smoke at home	Kordestan	Marivan	Male	45.18	20.74	70.59
**4978**	Exposure to secondhand smoke at home	Fars	Marvdasht	Male	24.62	4.64	43.82
**4979**	Exposure to secondhand smoke at home	Gilan	Masal	Male	22.98	1.10	44.89
**4980**	Exposure to secondhand smoke at home	Khorasan_razavi	Mashhad	Male	20.75	5.95	35.76
**4981**	Exposure to secondhand smoke at home	Khuzestan	Masjed Soleyman	Male	39.04	17.64	61.93
**4982**	Exposure to secondhand smoke at home	Semnan	Mayamey	Male	17.75	0.00	40.02
**4983**	Exposure to secondhand smoke at home	Semnan	Mehdishahr	Male	18.08	0.07	36.46
**4984**	Exposure to secondhand smoke at home	Ilam	Mehran	Male	32.09	2.01	60.97
**4985**	Exposure to secondhand smoke at home	Yazd	Mehriz	Male	14.77	0.49	28.61
**4986**	Exposure to secondhand smoke at home	Ardebil	Meshkinshahr	Male	33.07	11.53	55.04
**4987**	Exposure to secondhand smoke at home	Yazd	Meybod	Male	20.67	5.18	35.34
**4988**	Exposure to secondhand smoke at home	Hormozgan	Minab	Male	21.97	4.96	37.98
**4989**	Exposure to secondhand smoke at home	Golestan	Minudasht	Male	14.06	0.00	34.37
**4990**	Exposure to secondhand smoke at home	Sistan and Balouchestan	Mirjaveh	Male	26.96	0.00	54.70
**4991**	Exposure to secondhand smoke at home	Azarbayjan_West	Miyandoab	Male	43.08	22.28	63.90
**4992**	Exposure to secondhand smoke at home	Mazandaran	Miyandorud	Male	17.37	0.00	47.71
**4993**	Exposure to secondhand smoke at home	Azararbayjan_East	Miyaneh	Male	29.78	9.47	50.27

**4994**	Exposure to secondhand smoke at home	Isfahan	Mobarakeh	Male	29.20	10.27	47.51
**4995**	Exposure to secondhand smoke at home	Fars	Mohr	Male	39.93	16.54	63.93
**4996**	Exposure to secondhand smoke at home	Hamedan	Nahavand	Male	33.99	11.68	55.75
**4997**	Exposure to secondhand smoke at home	Isfahan	Najafabad	Male	27.20	8.60	44.81
**4998**	Exposure to secondhand smoke at home	Ardebil	Namin	Male	42.21	18.09	67.08
**4999**	Exposure to secondhand smoke at home	Azarbayjan_West	Naqadeh	Male	50.01	29.16	72.22
**5000**	Exposure to secondhand smoke at home	Kerman	Narmashir	Male	37.19	9.08	65.86
**5001**	Exposure to secondhand smoke at home	Isfahan	Natanz	Male	22.58	0.00	46.92
**5002**	Exposure to secondhand smoke at home	Isfahan	Nayin	Male	21.72	0.00	46.05
**5003**	Exposure to secondhand smoke at home	Alborz	Nazarabad	Male	25.74	5.89	46.80
**5004**	Exposure to secondhand smoke at home	Ardebil	Neer	Male	36.10	8.42	64.63
**5005**	Exposure to secondhand smoke at home	Khorasan_South	Nehbandan	Male	12.51	0.00	30.00
**5006**	Exposure to secondhand smoke at home	Mazandaran	Neka	Male	18.17	0.00	41.39
**5007**	Exposure to secondhand smoke at home	Fars	Neyriz	Male	35.86	15.68	57.02
**5008**	Exposure to secondhand smoke at home	Khorasan_razavi	Neyshabur	Male	19.23	3.46	34.73
**5009**	Exposure to secondhand smoke at home	Sistan and Balouchestan	Nikshahr	Male	27.53	8.60	45.76
**5010**	Exposure to secondhand smoke at home	Sistan and Balouchestan	Nimruz	Male	16.17	0.00	39.71
**5011**	Exposure to secondhand smoke at home	Mazandaran	Noshahr	Male	25.07	0.00	52.78
**5012**	Exposure to secondhand smoke at home	Mazandaran	Nur	Male	14.57	0.00	31.38
**5013**	Exposure to secondhand smoke at home	Khuzestan	Omidiyeh	Male	39.40	17.82	62.14
**5014**	Exposure to secondhand smoke at home	Azarbayjan_West	Orumiyeh	Male	39.42	19.24	60.13
**5015**	Exposure to secondhand smoke at home	Azarbayjan_West	Oshnaviyeh	Male	48.85	24.48	73.99
**5016**	Exposure to secondhand smoke at home	Azararbayjan_East	Osku	Male	25.64	3.09	48.53
**5017**	Exposure to secondhand smoke at home	Tehran	Pakdasht	Male	11.04	0.00	27.17
**5018**	Exposure to secondhand smoke at home	Tehran	Pardis	Male	16.55	0.00	41.23
**5019**	Exposure to secondhand smoke at home	Ardebil	Parsabad	Male	23.34	0.00	48.35
**5020**	Exposure to secondhand smoke at home	Hormozgan	Parsian (Gavbandi)	Male	16.26	0.00	33.07
**5021**	Exposure to secondhand smoke at home	Fars	Pasargad	Male	37.06	14.04	61.69
**5022**	Exposure to secondhand smoke at home	Kermanshah	Paveh	Male	33.45	10.08	57.13
**5023**	Exposure to secondhand smoke at home	Azarbayjan_West	Piranshahr	Male	50.94	25.90	78.00
**5024**	Exposure to secondhand smoke at home	Tehran	Pishva	Male	20.76	0.40	41.98
**5025**	Exposure to secondhand smoke at home	Azarbayjan_West	Poldasht	Male	17.16	0.00	47.63
**5026**	Exposure to secondhand smoke at home	Lorestan	Poldokhtar	Male	31.93	7.22	56.47
**5027**	Exposure to secondhand smoke at home	Mazandaran	Qaemshahr	Male	11.69	0.00	29.20
**5028**	Exposure to secondhand smoke at home	Tehran	Qarchak	Male	17.98	0.00	44.60
**5029**	Exposure to secondhand smoke at home	Sistan and Balouchestan	Qasr qand	Male	30.17	5.32	55.44
**5030**	Exposure to secondhand smoke at home	Kermanshah	Qasr-e-Shirin	Male	28.87	0.00	58.82
**5031**	Exposure to secondhand smoke at home	Khorasan_South	Qayenat	Male	19.04	0.00	37.66
**5032**	Exposure to secondhand smoke at home	Qazvin	Qazvin	Male	39.85	19.53	61.34
**5033**	Exposure to secondhand smoke at home	Hormozgan	Qeshm	Male	21.20	1.47	39.95
**5034**	Exposure to secondhand smoke at home	Fars	Qirokarzin	Male	34.05	15.26	52.60
**5035**	Exposure to secondhand smoke at home	Qom	Qom	Male	26.83	0.00	54.93
**5036**	Exposure to secondhand smoke at home	Kordestan	Qorveh	Male	19.62	0.00	42.91
**5037**	Exposure to secondhand smoke at home	Khorasan_razavi	Quchan	Male	34.32	13.45	58.60
**5038**	Exposure to secondhand smoke at home	Kerman	Rabar	Male	46.90	21.36	74.79
**5039**	Exposure to secondhand smoke at home	Kerman	Rafsanjan	Male	35.23	12.97	59.71
**5040**	Exposure to secondhand smoke at home	Khuzestan	Ramhormoz	Male	36.63	15.52	56.52
**5041**	Exposure to secondhand smoke at home	Mazandaran	Ramsar	Male	19.87	4.66	37.04
**5042**	Exposure to secondhand smoke at home	Khuzestan	Ramshir	Male	58.01	35.56	77.59
**5043**	Exposure to secondhand smoke at home	Golestan	Ramyan	Male	25.56	0.36	51.28
**5044**	Exposure to secondhand smoke at home	Gilan	Rasht	Male	13.83	0.00	29.31
**5045**	Exposure to secondhand smoke at home	Khorasan_razavi	Rashtkhar	Male	21.19	0.00	42.00
**5046**	Exposure to secondhand smoke at home	Kermanshah	Ravansar	Male	32.38	7.97	56.66
**5047**	Exposure to secondhand smoke at home	Kerman	Ravar	Male	19.19	0.24	39.26
**5048**	Exposure to secondhand smoke at home	Khorasan_North	Raz va Jergolan	Male	22.33	0.00	55.89
**5049**	Exposure to secondhand smoke at home	Hamedan	Razan	Male	40.79	16.19	66.53
**5050**	Exposure to secondhand smoke at home	Tehran	Rey	Male	20.52	2.62	38.99
**5051**	Exposure to secondhand smoke at home	Kerman	Reygan	Male	57.57	31.38	87.35
**5052**	Exposure to secondhand smoke at home	Gilan	Rezvanshahr	Male	22.09	0.00	45.75
**5053**	Exposure to secondhand smoke at home	Tehran	Robatkarim	Male	10.92	0.00	28.27
**5054**	Exposure to secondhand smoke at home	Fars	Rostam	Male	41.04	16.80	64.70
**5055**	Exposure to secondhand smoke at home	Kerman	Roudbar-e-Jonub	Male	39.56	13.41	65.99
**5056**	Exposure to secondhand smoke at home	Hormozgan	Rudan	Male	11.44	0.00	27.78
**5057**	Exposure to secondhand smoke at home	Gilan	Rudbar	Male	24.58	6.54	44.22
**5058**	Exposure to secondhand smoke at home	Gilan	Rudsar	Male	13.60	0.00	28.40
**5059**	Exposure to secondhand smoke at home	Lorestan	Rumshekan	Male	33.03	3.15	63.90
**5060**	Exposure to secondhand smoke at home	Khorasan_razavi	Sabzevar	Male	17.08	0.14	33.27
**5061**	Exposure to secondhand smoke at home	Yazd	Sadugh	Male	37.27	20.43	52.29
**5062**	Exposure to secondhand smoke at home	Kermanshah	Sahneh	Male	32.30	10.53	53.74
**5063**	Exposure to secondhand smoke at home	Kermanshah	Salas-e-Babajani	Male	30.41	7.56	53.47
**5064**	Exposure to secondhand smoke at home	Azarbayjan_West	Salmas	Male	33.03	8.05	59.61
**5065**	Exposure to secondhand smoke at home	Chaharmahal	Saman	Male	32.82	3.64	61.89
**5066**	Exposure to secondhand smoke at home	Kordestan	Sanandaj	Male	41.70	21.31	62.45
**5067**	Exposure to secondhand smoke at home	Kordestan	Saqqez	Male	50.83	28.10	75.70
**5068**	Exposure to secondhand smoke at home	Kermanshah	Sar-e-Pol-e-Zohab	Male	25.97	4.64	46.89
**5069**	Exposure to secondhand smoke at home	Azararbayjan_East	Sarab	Male	29.13	6.21	53.10
**5070**	Exposure to secondhand smoke at home	Khorasan_razavi	Sarakhs	Male	30.45	10.25	50.73
**5071**	Exposure to secondhand smoke at home	Sistan and Balouchestan	Saravan	Male	42.61	20.08	66.02
**5072**	Exposure to secondhand smoke at home	Khorasan_South	Sarayan	Male	19.41	0.00	43.33
**5073**	Exposure to secondhand smoke at home	Sistan and Balouchestan	Sarbaz	Male	33.38	15.11	52.94
**5074**	Exposure to secondhand smoke at home	Khorasan_South	Sarbisheh	Male	22.69	0.00	47.83
**5075**	Exposure to secondhand smoke at home	Azarbayjan_West	Sardasht	Male	52.30	28.75	78.13
**5076**	Exposure to secondhand smoke at home	Ardebil	Sarein	Male	35.45	6.45	64.89
**5077**	Exposure to secondhand smoke at home	Mazandaran	Sari	Male	15.52	0.00	33.09
**5078**	Exposure to secondhand smoke at home	Kordestan	Sarvabad	Male	42.72	13.21	74.28
**5079**	Exposure to secondhand smoke at home	Fars	Sarvestan	Male	38.40	13.30	62.73
**5080**	Exposure to secondhand smoke at home	Mazandaran	Savadkuh	Male	18.49	0.00	42.36
**5081**	Exposure to secondhand smoke at home	Mazandaran	Savadkuh_North	Male	16.11	0.00	41.12
**5082**	Exposure to secondhand smoke at home	Markazi	Saveh	Male	33.86	9.92	57.18
**5083**	Exposure to secondhand smoke at home	Alborz	Savojbolagh	Male	26.59	9.72	43.88
**5084**	Exposure to secondhand smoke at home	Lorestan	Selseleh	Male	31.75	6.90	55.14
**5085**	Exposure to secondhand smoke at home	Isfahan	Semirom	Male	35.78	15.37	56.71
**5086**	Exposure to secondhand smoke at home	Isfahan	Semirom-e-Sofla	Male	31.84	8.05	57.98
**5087**	Exposure to secondhand smoke at home	Semnan	Semnan	Male	14.14	0.00	31.36
**5088**	Exposure to secondhand smoke at home	Fars	Sepidan	Male	39.53	17.19	62.35
**5089**	Exposure to secondhand smoke at home	Azararbayjan_East	Shabestar	Male	9.97	0.00	28.88
**5090**	Exposure to secondhand smoke at home	Khuzestan	Shadegan	Male	30.49	9.24	50.91
**5091**	Exposure to secondhand smoke at home	Gilan	Shaft	Male	15.19	0.00	31.72

**5092**	Exposure to secondhand smoke at home	Azarbayjan_West	Shahindezh	Male	46.76	22.70	71.30
**5093**	Exposure to secondhand smoke at home	Tehran	Shahr-e Qods	Male	19.78	0.79	38.85
**5094**	Exposure to secondhand smoke at home	Kerman	Shahr-e-Babak	Male	19.69	0.00	41.75
**5095**	Exposure to secondhand smoke at home	Chaharmahal	Shahr-e-Kord	Male	26.65	7.25	45.46
**5096**	Exposure to secondhand smoke at home	Isfahan	Shahreza	Male	31.94	11.28	51.67
**5097**	Exposure to secondhand smoke at home	Tehran	Shahriyar	Male	18.54	2.88	33.70
**5098**	Exposure to secondhand smoke at home	Semnan	Shahrud	Male	15.21	0.00	30.17
**5099**	Exposure to secondhand smoke at home	Markazi	Shazand	Male	46.30	20.27	74.27
**5100**	Exposure to secondhand smoke at home	Tehran	Shemiranat	Male	13.42	0.00	30.41
**5101**	Exposure to secondhand smoke at home	Fars	Shiraz	Male	33.60	17.34	50.11
**5102**	Exposure to secondhand smoke at home	Khorasan_North	Shirvan	Male	21.47	0.44	42.25
**5103**	Exposure to secondhand smoke at home	Ilam	Shirvan and Chard-e-Aval	Male	41.78	17.06	67.57
**5104**	Exposure to secondhand smoke at home	Azarbayjan_West	Showt	Male	16.03	0.00	46.94
**5105**	Exposure to secondhand smoke at home	Khuzestan	Shush	Male	43.02	23.70	65.34
**5106**	Exposure to secondhand smoke at home	Khuzestan	Shushtar	Male	32.93	13.63	51.07
**5107**	Exposure to secondhand smoke at home	Gilan	Siahkal	Male	27.12	11.17	42.79
**5108**	Exposure to secondhand smoke at home	Sistan and Balouchestan	Sib o Soran	Male	36.34	14.54	59.58
**5109**	Exposure to secondhand smoke at home	Mazandaran	Simorgh	Male	15.20	0.00	41.73
**5110**	Exposure to secondhand smoke at home	Hormozgan	Sirik	Male	47.63	25.64	65.69
**5111**	Exposure to secondhand smoke at home	Kerman	Sirjan	Male	31.14	10.12	51.99
**5112**	Exposure to secondhand smoke at home	Ilam	Sirvan	Male	38.56	7.44	69.88
**5113**	Exposure to secondhand smoke at home	Zanjan	Soltaniyeh	Male	37.04	11.64	63.68
**5114**	Exposure to secondhand smoke at home	Kermanshah	Sonqor	Male	31.28	11.17	51.98
**5115**	Exposure to secondhand smoke at home	Semnan	Sorkheh	Male	17.44	0.00	40.50
**5116**	Exposure to secondhand smoke at home	Gilan	Sume'eh Sara	Male	20.38	1.59	39.05
**5117**	Exposure to secondhand smoke at home	Khorasan_South	Tabas	Male	21.20	1.86	41.52
**5118**	Exposure to secondhand smoke at home	Azararbayjan_East	Tabriz	Male	23.13	6.39	39.67
**5119**	Exposure to secondhand smoke at home	Markazi	Tafresh	Male	26.84	0.53	51.81
**5120**	Exposure to secondhand smoke at home	Yazd	Taft	Male	24.45	8.36	41.51
**5121**	Exposure to secondhand smoke at home	Azarbayjan_West	Takab	Male	59.25	38.44	77.88
**5122**	Exposure to secondhand smoke at home	Qazvin	Takestan	Male	37.32	14.25	59.67
**5123**	Exposure to secondhand smoke at home	Khorasan_razavi	Takht-e-Jolgeh (Firuzeh)	Male	21.96	5.48	39.12
**5124**	Exposure to secondhand smoke at home	Alborz	Taleghan	Male	28.07	9.57	47.61
**5125**	Exposure to secondhand smoke at home	Boushehr	Tangestan	Male	38.59	14.19	64.18
**5126**	Exposure to secondhand smoke at home	Zanjan	Tarom	Male	38.91	17.33	61.66
**5127**	Exposure to secondhand smoke at home	Gilan	Tavalesh	Male	25.38	6.89	44.99
**5128**	Exposure to secondhand smoke at home	Khorasan_razavi	Taybad	Male	35.19	12.56	58.53
**5129**	Exposure to secondhand smoke at home	Tehran	Tehran	Male	16.11	2.57	30.24
**5130**	Exposure to secondhand smoke at home	Isfahan	Tiran and Karvan	Male	25.35	3.60	46.33
**5131**	Exposure to secondhand smoke at home	Mazandaran	Tonekabon	Male	27.27	4.99	50.23
**5132**	Exposure to secondhand smoke at home	Khorasan_razavi	Torbat-e-Heydariyeh	Male	21.60	3.62	39.05
**5133**	Exposure to secondhand smoke at home	Khorasan_razavi	Torbat-e-Jam	Male	30.82	10.42	53.18
**5134**	Exposure to secondhand smoke at home	Hamedan	Tuyserkan	Male	39.41	15.57	63.76
**5135**	Exposure to secondhand smoke at home	Tehran	Varamin	Male	18.36	2.18	35.20
**5136**	Exposure to secondhand smoke at home	Azararbayjan_East	Varzaqan	Male	18.73	0.00	44.36
**5137**	Exposure to secondhand smoke at home	Yazd	Yazd	Male	18.04	5.17	31.07
**5138**	Exposure to secondhand smoke at home	Sistan and Balouchestan	Zabol	Male	9.89	0.00	27.16
**5139**	Exposure to secondhand smoke at home	Sistan and Balouchestan	Zaboli (Mehrestan)	Male	31.99	6.61	57.67
**5140**	Exposure to secondhand smoke at home	Sistan and Balouchestan	Zahedan	Male	20.36	4.39	35.53
**5141**	Exposure to secondhand smoke at home	Zanjan	Zanjan	Male	33.11	14.32	51.10
**5142**	Exposure to secondhand smoke at home	Kerman	Zarand	Male	25.15	1.08	49.04
**5143**	Exposure to secondhand smoke at home	Markazi	Zarandiyeh	Male	37.87	10.78	67.56
**5144**	Exposure to secondhand smoke at home	Fars	Zarrindasht	Male	37.75	16.08	58.36
**5145**	Exposure to secondhand smoke at home	Khorasan_razavi	Zave	Male	18.41	0.46	35.89
**5146**	Exposure to secondhand smoke at home	Sistan and Balouchestan	Zehak	Male	13.55	0.00	40.55
**5147**	Exposure to secondhand smoke at home	Khorasan_South	Zir kuh	Male	21.56	0.00	48.54
**5148**	Past daily cigarette smoke	Khuzestan	Abadan	Female	0.23	0.00	3.12
**5149**	Past daily cigarette smoke	Fars	Abadeh	Female	0.71	0.00	7.53
**5150**	Past daily cigarette smoke	Yazd	Abarkuh	Female	1.59	0.00	11.41
**5151**	Past daily cigarette smoke	Mazandaran	Abbas abad	Female	1.15	0.00	8.79
**5152**	Past daily cigarette smoke	Ilam	Abdanan	Female	2.14	0.00	9.39
**5153**	Past daily cigarette smoke	Zanjan	Abhar	Female	0.49	0.00	3.78
**5154**	Past daily cigarette smoke	Hormozgan	Abumusa	Female	0.69	0.00	6.42
**5155**	Past daily cigarette smoke	Qazvin	Abyek	Female	1.16	0.00	7.07
**5156**	Past daily cigarette smoke	Azararbayjan_East	Ahar	Female	2.34	0.00	4.95
**5157**	Past daily cigarette smoke	Khuzestan	Ahvaz	Female	0.86	0.00	3.51
**5158**	Past daily cigarette smoke	Azararbayjan_East	Ajabshir	Female	1.34	0.00	4.52
**5159**	Past daily cigarette smoke	Qazvin	Alborz	Female	1.33	0.00	5.95
**5160**	Past daily cigarette smoke	Golestan	Aliabad	Female	1.76	0.00	9.21
**5161**	Past daily cigarette smoke	Lorestan	Aligudarz	Female	0.26	0.00	2.45
**5162**	Past daily cigarette smoke	Gilan	Amlash	Female	0.52	0.00	5.02
**5163**	Past daily cigarette smoke	Mazandaran	Amol	Female	1.05	0.00	6.34
**5164**	Past daily cigarette smoke	Kerman	Anar	Female	0.91	0.00	6.04
**5165**	Past daily cigarette smoke	Kerman	Anbarabad	Female	0.41	0.00	3.86
**5166**	Past daily cigarette smoke	Khuzestan	Andika	Female	0.68	0.00	4.47
**5167**	Past daily cigarette smoke	Khuzestan	Andimeshk	Female	0.55	0.00	3.27
**5168**	Past daily cigarette smoke	Golestan	Aq Qala	Female	0.71	0.00	6.22
**5169**	Past daily cigarette smoke	Khuzestan	Aqajari	Female	0.52	0.00	4.44
**5170**	Past daily cigarette smoke	Semnan	Aradan	Female	0.57	0.00	4.64
**5171**	Past daily cigarette smoke	Markazi	Arak	Female	1.38	0.00	3.38
**5172**	Past daily cigarette smoke	Isfahan	Aran and Bidgol	Female	0.62	0.00	4.83
**5173**	Past daily cigarette smoke	Ardebil	Ardabil	Female	1.48	0.00	4.97
**5174**	Past daily cigarette smoke	Yazd	Ardakan	Female	0.78	0.00	7.38
**5175**	Past daily cigarette smoke	Chaharmahal	Ardal	Female	0.15	0.00	1.83
**5176**	Past daily cigarette smoke	Isfahan	Ardestan	Female	0.94	0.00	6.72
**5177**	Past daily cigarette smoke	Fars	Arsanjan	Female	1.16	0.00	10.39
**5178**	Past daily cigarette smoke	Kerman	Arzouyeh	Female	0.75	0.00	5.47
**5179**	Past daily cigarette smoke	Hamedan	Asadabad	Female	0.84	0.00	2.59
**5180**	Past daily cigarette smoke	Boushehr	Asaluyeh	Female	1.01	0.00	8.16
**5181**	Past daily cigarette smoke	Markazi	Ashtiyan	Female	1.57	0.00	4.24
**5182**	Past daily cigarette smoke	Gilan	Astaneh-ye-Ashrafiyeh	Female	0.92	0.00	4.80
**5183**	Past daily cigarette smoke	Gilan	Astara	Female	0.89	0.00	5.85
**5184**	Past daily cigarette smoke	Qazvin	Avaj	Female	1.39	0.00	7.06
**5185**	Past daily cigarette smoke	Golestan	Azadshahr	Female	1.20	0.00	9.08
**5186**	Past daily cigarette smoke	Azararbayjan_East	Azarshahr	Female	0.76	0.00	3.17
**5187**	Past daily cigarette smoke	Lorestan	Azna	Female	0.61	0.00	3.90
**5188**	Past daily cigarette smoke	Mazandaran	Babol	Female	0.81	0.00	6.37
**5189**	Past daily cigarette smoke	Mazandaran	Babolsar	Female	0.64	0.00	8.46

**5190**	Past daily cigarette smoke	Ilam	Badreh	Female	1.94	0.00	10.00
**5191**	Past daily cigarette smoke	Yazd	Bafq	Female	0.90	0.00	7.72
**5192**	Past daily cigarette smoke	Kerman	Baft	Female	0.56	0.00	3.96
**5193**	Past daily cigarette smoke	Khuzestan	Baghemalek	Female	0.34	0.00	2.90
**5194**	Past daily cigarette smoke	Yazd	Bahabad	Female	1.33	0.00	11.13
**5195**	Past daily cigarette smoke	Hamedan	Bahar	Female	1.89	0.00	4.12
**5196**	Past daily cigarette smoke	Tehran	Baharestan (Golestan)	Female	1.99	0.00	7.29
**5197**	Past daily cigarette smoke	Kohkiluye and Bouyer Ahmad	Bahmani	Female	0.93	0.00	7.39
**5198**	Past daily cigarette smoke	Khorasan_razavi	Bajestan	Female	0.69	0.00	7.11
**5199**	Past daily cigarette smoke	Khorasan_razavi	Bakhraz	Female	0.29	0.00	5.23
**5200**	Past daily cigarette smoke	Kerman	Bam	Female	0.47	0.00	3.96
**5201**	Past daily cigarette smoke	Hormozgan	Bandar-e-Abbas	Female	0.68	0.00	4.35
**5202**	Past daily cigarette smoke	Gilan	Bandar-e-Anzali	Female	1.00	0.00	4.27
**5203**	Past daily cigarette smoke	Golestan	Bandar-e-Gaz	Female	1.29	0.00	10.05
**5204**	Past daily cigarette smoke	Hormozgan	Bandar-e-Jask	Female	0.32	0.00	5.79
**5205**	Past daily cigarette smoke	Hormozgan	Bandar-e-Lengeh	Female	1.15	0.00	5.49
**5206**	Past daily cigarette smoke	Khuzestan	Bandar-e-Mahshahr	Female	0.33	0.00	2.98
**5207**	Past daily cigarette smoke	Golestan	Bandar-e-Torkaman	Female	1.38	0.00	9.36
**5208**	Past daily cigarette smoke	Kordestan	Baneh	Female	5.45	0.79	10.19
**5209**	Past daily cigarette smoke	Khorasan_razavi	Bardeskan	Female	0.52	0.00	6.90
**5210**	Past daily cigarette smoke	Kerman	Bardsir	Female	3.35	0.00	6.97
**5211**	Past daily cigarette smoke	Hormozgan	Bashagerd	Female	0.30	0.00	5.55
**5212**	Past daily cigarette smoke	Kohkiluye and Bouyer Ahmad	Basht	Female	1.68	0.00	11.18
**5213**	Past daily cigarette smoke	Hormozgan	Bastak	Female	0.66	0.00	5.92
**5214**	Past daily cigarette smoke	Khuzestan	Bavi	Female	0.70	0.00	4.48
**5215**	Past daily cigarette smoke	Khuzestan	Behbahan	Female	0.30	0.00	2.98
**5216**	Past daily cigarette smoke	Mazandaran	Behshahr	Female	0.19	0.00	5.68
**5217**	Past daily cigarette smoke	Kordestan	Bijar	Female	2.79	0.00	8.33
**5218**	Past daily cigarette smoke	Ardebil	Bilehsavar	Female	2.62	0.00	7.75
**5219**	Past daily cigarette smoke	Khorasan_razavi	Binaloud	Female	0.46	0.00	7.28
**5220**	Past daily cigarette smoke	Khorasan_South	Birjand	Female	2.24	0.00	7.85
**5221**	Past daily cigarette smoke	Khorasan_North	Bojnurd	Female	0.99	0.00	5.14
**5222**	Past daily cigarette smoke	Chaharmahal	Bon	Female	0.30	0.00	2.86
**5223**	Past daily cigarette smoke	Azararbayjan_East	Bonab	Female	0.82	0.00	3.13
**5224**	Past daily cigarette smoke	Isfahan	Borkhar	Female	0.59	0.00	4.86
**5225**	Past daily cigarette smoke	Isfahan	Borkhar and Meymeh	Female	1.67	0.00	6.82
**5226**	Past daily cigarette smoke	Chaharmahal	Borujen	Female	0.16	0.00	1.85
**5227**	Past daily cigarette smoke	Lorestan	Borujerd	Female	0.33	0.00	2.49
**5228**	Past daily cigarette smoke	Khorasan_South	Boshruyeh	Female	1.57	0.00	9.17
**5229**	Past daily cigarette smoke	Azararbayjan_East	Bostanabad	Female	1.07	0.00	3.39
**5230**	Past daily cigarette smoke	Fars	Bovanat	Female	1.19	0.00	10.59
**5231**	Past daily cigarette smoke	Kohkiluye and Bouyer Ahmad	Boyer Ahmad	Female	0.96	0.00	7.33
**5232**	Past daily cigarette smoke	Qazvin	Boyinzahra	Female	0.76	0.00	4.76
**5233**	Past daily cigarette smoke	Isfahan	Buein va Miandasht	Female	1.28	0.00	7.35
**5234**	Past daily cigarette smoke	Azarbayjan_West	Bukan	Female	2.39	0.00	6.07
**5235**	Past daily cigarette smoke	Boushehr	Bushehr	Female	1.54	0.00	7.59
**5236**	Past daily cigarette smoke	Isfahan	Chadegan	Female	1.10	0.00	6.98
**5237**	Past daily cigarette smoke	Sistan and Balouchestan	Chahbahar	Female	0.88	0.00	19.93
**5238**	Past daily cigarette smoke	Azarbayjan_West	Chaipareh	Female	2.47	0.00	7.49
**5239**	Past daily cigarette smoke	Azarbayjan_West	Chaldoran	Female	2.39	0.00	7.44
**5240**	Past daily cigarette smoke	Mazandaran	Chalus	Female	0.46	0.00	5.76
**5241**	Past daily cigarette smoke	Azararbayjan_East	Charoimaq	Female	1.73	0.00	4.68
**5242**	Past daily cigarette smoke	Khorasan_razavi	Chenaran	Female	0.20	0.00	4.88
**5243**	Past daily cigarette smoke	Kohkiluye and Bouyer Ahmad	Cheram	Female	1.58	0.00	10.80
**5244**	Past daily cigarette smoke	Kermanshah	Dalaho	Female	1.55	0.00	6.10
**5245**	Past daily cigarette smoke	Lorestan	Dalfan	Female	0.21	0.00	2.29
**5246**	Past daily cigarette smoke	Sistan and Balouchestan	Dalgan	Female	1.78	0.00	28.59
**5247**	Past daily cigarette smoke	Tehran	Damavand	Female	0.46	0.00	5.36
**5248**	Past daily cigarette smoke	Semnan	Damghan	Female	0.28	0.00	3.11
**5249**	Past daily cigarette smoke	Fars	Darab	Female	1.91	0.00	10.29
**5250**	Past daily cigarette smoke	Khorasan_South	Darmian	Female	2.69	0.00	10.34
**5251**	Past daily cigarette smoke	Khorasan_razavi	Darrehgaz	Female	0.32	0.00	4.94
**5252**	Past daily cigarette smoke	Ilam	Darrehshahr	Female	0.99	0.00	7.32
**5253**	Past daily cigarette smoke	Khuzestan	Dasht-e-Azadegan	Female	0.47	0.00	3.25
**5254**	Past daily cigarette smoke	Boushehr	Dashtestan	Female	0.59	0.00	5.47
**5255**	Past daily cigarette smoke	Boushehr	Dashti	Female	0.50	0.00	5.44
**5256**	Past daily cigarette smoke	Khorasan_razavi	Davarzan	Female	0.47	0.00	7.30
**5257**	Past daily cigarette smoke	Boushehr	Dayyer	Female	0.96	0.00	8.60
**5258**	Past daily cigarette smoke	Kordestan	Dehgolan	Female	2.63	0.00	8.43
**5259**	Past daily cigarette smoke	Ilam	Dehloran	Female	3.16	0.00	10.16
**5260**	Past daily cigarette smoke	Markazi	Delijan	Female	1.51	0.00	3.93
**5261**	Past daily cigarette smoke	Kohkiluye and Bouyer Ahmad	Dena	Female	0.88	0.00	7.60
**5262**	Past daily cigarette smoke	Boushehr	Deylam	Female	0.96	0.00	8.14
**5263**	Past daily cigarette smoke	Khuzestan	Dezful	Female	0.48	0.00	3.16
**5264**	Past daily cigarette smoke	Kordestan	Divandarreh	Female	3.15	0.00	8.65
**5265**	Past daily cigarette smoke	Lorestan	Dorud	Female	0.34	0.00	2.59
**5266**	Past daily cigarette smoke	Lorestan	Doureh	Female	0.35	0.00	3.34
**5267**	Past daily cigarette smoke	Fars	Eqlid	Female	1.07	0.00	10.12
**5268**	Past daily cigarette smoke	Khorasan_North	Esfarayen	Female	0.86	0.00	5.00
**5269**	Past daily cigarette smoke	Alborz	Eshtehard	Female	0.91	0.00	4.79
**5270**	Past daily cigarette smoke	Kermanshah	Eslamabad-e-Gharb	Female	2.38	0.00	5.77
**5271**	Past daily cigarette smoke	Tehran	Eslamshahr	Female	0.63	0.00	5.52
**5272**	Past daily cigarette smoke	Fars	Estahban	Female	1.08	0.00	10.45
**5273**	Past daily cigarette smoke	Ilam	Eyvan	Female	2.82	0.00	10.89
**5274**	Past daily cigarette smoke	Kerman	Fahraj	Female	0.92	0.00	5.94
**5275**	Past daily cigarette smoke	Isfahan	Falavarjan	Female	0.58	0.00	4.95
**5276**	Past daily cigarette smoke	Hamedan	Famenin	Female	6.98	4.18	9.91
**5277**	Past daily cigarette smoke	Markazi	Farahan	Female	1.63	0.00	4.37
**5278**	Past daily cigarette smoke	Fars	Farashband	Female	0.87	0.00	9.40
**5279**	Past daily cigarette smoke	Alborz	Fardis	Female	0.88	0.00	4.78
**5280**	Past daily cigarette smoke	Isfahan	Faridan	Female	0.64	0.00	5.05
**5281**	Past daily cigarette smoke	Khorasan_razavi	Fariman	Female	0.28	0.00	4.90
**5282**	Past daily cigarette smoke	Khorasan_North	Faroj	Female	0.48	0.00	4.52
**5283**	Past daily cigarette smoke	Chaharmahal	Farsan	Female	0.14	0.00	1.87
**5284**	Past daily cigarette smoke	Kerman	Faryab	Female	0.75	0.00	5.51
**5285**	Past daily cigarette smoke	Fars	Fasa	Female	0.71	0.00	7.31
**5286**	Past daily cigarette smoke	Khorasan_South	Ferdows	Female	1.62	0.00	9.60
**5287**	Past daily cigarette smoke	Mazandaran	Fereydunkenar	Female	0.36	0.00	6.03

**5288**	Past daily cigarette smoke	Isfahan	Fereydunshahr	Female	1.18	0.00	7.09
**5289**	Past daily cigarette smoke	Fars	Firozabad	Female	0.49	0.00	7.02
**5290**	Past daily cigarette smoke	Tehran	Firuzkuh	Female	0.43	0.00	5.20
**5291**	Past daily cigarette smoke	Sistan and Balouchestan	Fonuj	Female	1.85	0.00	28.17
**5292**	Past daily cigarette smoke	Gilan	Fuman	Female	0.37	0.00	3.19
**5293**	Past daily cigarette smoke	Kohkiluye and Bouyer Ahmad	Gachsaran	Female	2.71	0.00	11.03
**5294**	Past daily cigarette smoke	Golestan	Galikesh	Female	0.69	0.00	6.14
**5295**	Past daily cigarette smoke	Mazandaran	Galugah	Female	0.41	0.00	8.79
**5296**	Past daily cigarette smoke	Semnan	Garmsar	Female	0.37	0.00	3.24
**5297**	Past daily cigarette smoke	Boushehr	Genaveh	Female	0.51	0.00	5.63
**5298**	Past daily cigarette smoke	Fars	Gerash	Female	0.87	0.00	10.67
**5299**	Past daily cigarette smoke	Khorasan_North	Germeh	Female	0.71	0.00	6.61
**5300**	Past daily cigarette smoke	Ardebil	Germi	Female	2.53	0.00	6.99
**5301**	Past daily cigarette smoke	Kerman	Ghaleye-Ganj	Female	0.32	0.00	3.82
**5302**	Past daily cigarette smoke	Kermanshah	Gilan-e-Gharb	Female	1.48	0.00	6.08
**5303**	Past daily cigarette smoke	Isfahan	Golpayegan	Female	1.72	0.00	7.31
**5304**	Past daily cigarette smoke	Golestan	Gomishan	Female	1.25	0.00	10.10
**5305**	Past daily cigarette smoke	Khorasan_razavi	Gonabad	Female	0.68	0.00	7.08
**5306**	Past daily cigarette smoke	Golestan	Gonbad-e-Kavus	Female	0.67	0.00	5.93
**5307**	Past daily cigarette smoke	Golestan	Gorgan	Female	2.33	0.00	8.96
**5308**	Past daily cigarette smoke	Khuzestan	Guotvand	Female	0.72	0.00	4.40
**5309**	Past daily cigarette smoke	Khuzestan	Haftgol	Female	0.66	0.00	4.22
**5310**	Past daily cigarette smoke	Hormozgan	Hajiabad	Female	0.37	0.00	3.85
**5311**	Past daily cigarette smoke	Hamedan	Hamadan	Female	1.43	0.00	3.20
**5312**	Past daily cigarette smoke	Khuzestan	Hamidiyeh	Female	0.80	0.00	4.63
**5313**	Past daily cigarette smoke	Sistan and Balouchestan	Hamoon	Female	2.35	0.00	30.05
**5314**	Past daily cigarette smoke	Azararbayjan_East	Haris	Female	2.53	0.00	5.29
**5315**	Past daily cigarette smoke	Kermanshah	Harsin	Female	1.46	0.00	6.45
**5316**	Past daily cigarette smoke	Azararbayjan_East	Hashtrud	Female	1.69	0.00	4.80
**5317**	Past daily cigarette smoke	Khuzestan	Hendijan	Female	0.51	0.00	4.32
**5318**	Past daily cigarette smoke	Sistan and Balouchestan	Hirmand	Female	1.06	0.00	20.22
**5319**	Past daily cigarette smoke	Khuzestan	Hoveizeh	Female	0.78	0.00	4.81
**5320**	Past daily cigarette smoke	Zanjan	Ijerod	Female	1.07	0.00	5.62
**5321**	Past daily cigarette smoke	Ilam	Ilam	Female	1.61	0.00	7.61
**5322**	Past daily cigarette smoke	Sistan and Balouchestan	Iranshahr	Female	1.23	0.00	18.61
**5323**	Past daily cigarette smoke	Isfahan	Isfahan	Female	0.96	0.00	5.33
**5324**	Past daily cigarette smoke	Khuzestan	Izeh	Female	0.34	0.00	3.00
**5325**	Past daily cigarette smoke	Fars	Jahrom	Female	0.53	0.00	6.92
**5326**	Past daily cigarette smoke	Khorasan_North	Jajarm	Female	0.96	0.00	6.50
**5327**	Past daily cigarette smoke	Boushehr	Jam	Female	0.51	0.00	5.52
**5328**	Past daily cigarette smoke	Kermanshah	Javanrud	Female	2.08	0.00	6.80
**5329**	Past daily cigarette smoke	Kerman	Jiroft	Female	0.45	0.00	3.81
**5330**	Past daily cigarette smoke	Khorasan_razavi	Joghatai	Female	0.56	0.00	7.37
**5331**	Past daily cigarette smoke	Azararbayjan_East	Jolfa	Female	2.33	0.00	5.52
**5332**	Past daily cigarette smoke	Khorasan_razavi	Jowayin	Female	0.58	0.00	7.12
**5333**	Past daily cigarette smoke	Mazandaran	Juybar	Female	0.50	0.00	8.36
**5334**	Past daily cigarette smoke	Hamedan	Kabudarahang	Female	1.04	0.00	2.77
**5335**	Past daily cigarette smoke	Kerman	Kahnuj	Female	0.68	0.00	5.30
**5336**	Past daily cigarette smoke	Golestan	Kalaleh	Female	1.91	0.00	9.29
**5337**	Past daily cigarette smoke	Khorasan_razavi	Kalat	Female	0.49	0.00	7.54
**5338**	Past daily cigarette smoke	Azararbayjan_East	Kaleibar	Female	9.45	5.82	13.24
**5339**	Past daily cigarette smoke	Kordestan	Kamyaran	Female	1.52	0.00	5.64
**5340**	Past daily cigarette smoke	Boushehr	Kangan	Female	1.83	0.00	8.66
**5341**	Past daily cigarette smoke	Kermanshah	Kangavar	Female	0.83	0.00	4.18
**5342**	Past daily cigarette smoke	Alborz	Karaj	Female	0.75	0.00	3.60
**5343**	Past daily cigarette smoke	Khuzestan	Karun	Female	0.53	0.00	4.64
**5344**	Past daily cigarette smoke	Isfahan	Kashan	Female	0.68	0.00	5.08
**5345**	Past daily cigarette smoke	Khorasan_razavi	Kashmar	Female	0.31	0.00	4.93
**5346**	Past daily cigarette smoke	Fars	Kavar	Female	0.86	0.00	10.24
**5347**	Past daily cigarette smoke	Fars	Kazerun	Female	0.56	0.00	7.21
**5348**	Past daily cigarette smoke	Mazandaran	Kelardasht	Female	0.91	0.00	8.48
**5349**	Past daily cigarette smoke	Kerman	Kerman	Female	0.81	0.00	4.15
**5350**	Past daily cigarette smoke	Kermanshah	Kermanshah	Female	1.55	0.00	4.85
**5351**	Past daily cigarette smoke	Khorasan_razavi	Khaf	Female	0.37	0.00	5.00
**5352**	Past daily cigarette smoke	Khorasan_razavi	Khalilabad	Female	0.68	0.00	7.61
**5353**	Past daily cigarette smoke	Ardebil	Khalkhal	Female	0.90	0.00	4.38
**5354**	Past daily cigarette smoke	Hormozgan	Khamir	Female	0.60	0.00	5.63
**5355**	Past daily cigarette smoke	Isfahan	Khansar	Female	1.23	0.00	7.41
**5356**	Past daily cigarette smoke	Sistan and Balouchestan	Khash	Female	1.21	0.00	18.96
**5357**	Past daily cigarette smoke	Yazd	Khatam	Female	2.21	0.00	11.45
**5358**	Past daily cigarette smoke	Fars	Kherameh	Female	1.17	0.00	10.05
**5359**	Past daily cigarette smoke	Azararbayjan_East	Khodaafarin	Female	3.04	0.00	6.38
**5360**	Past daily cigarette smoke	Zanjan	Khodabandeh	Female	1.26	0.00	4.65
**5361**	Past daily cigarette smoke	Markazi	Khomeyn	Female	0.94	0.00	2.88
**5362**	Past daily cigarette smoke	Isfahan	Khomeynishahr	Female	1.65	0.00	6.35
**5363**	Past daily cigarette smoke	Markazi	Khondab	Female	0.99	0.00	3.00
**5364**	Past daily cigarette smoke	Fars	Khonj	Female	0.86	0.00	9.70
**5365**	Past daily cigarette smoke	Isfahan	Khoor va Biabanak	Female	0.76	0.00	6.86
**5366**	Past daily cigarette smoke	Lorestan	Khorramabad	Female	0.37	0.00	2.54
**5367**	Past daily cigarette smoke	Fars	Khorrambid	Female	1.35	0.00	10.72
**5368**	Past daily cigarette smoke	Zanjan	Khorramdarreh	Female	0.49	0.00	3.81
**5369**	Past daily cigarette smoke	Khuzestan	Khorramshahr	Female	0.28	0.00	3.07
**5370**	Past daily cigarette smoke	Khorasan_razavi	Khoshab	Female	0.49	0.00	6.84
**5371**	Past daily cigarette smoke	Azarbayjan_West	Khoy	Female	1.26	0.00	4.83
**5372**	Past daily cigarette smoke	Khorasan_South	Khusef	Female	1.68	0.00	8.87
**5373**	Past daily cigarette smoke	Chaharmahal	Kiaar	Female	0.15	0.00	1.83
**5374**	Past daily cigarette smoke	Kohkiluye and Bouyer Ahmad	Kohgiluyeh	Female	2.56	0.00	10.54
**5375**	Past daily cigarette smoke	Markazi	Komeijan	Female	1.82	0.00	4.33
**5376**	Past daily cigarette smoke	Sistan and Balouchestan	Konarak	Female	1.63	0.00	28.83
**5377**	Past daily cigarette smoke	Golestan	Kordkuy	Female	1.30	0.00	9.27
**5378**	Past daily cigarette smoke	Ardebil	Kowsar	Female	0.85	0.00	5.16
**5379**	Past daily cigarette smoke	Kerman	Kuhbonan	Female	0.84	0.00	5.66
**5380**	Past daily cigarette smoke	Lorestan	Kuhdasht	Female	0.22	0.00	2.36
**5381**	Past daily cigarette smoke	Chaharmahal	Kuhrang	Female	0.30	0.00	2.56
**5382**	Past daily cigarette smoke	Gilan	Lahijan	Female	0.31	0.00	3.29
**5383**	Past daily cigarette smoke	Khuzestan	Lali	Female	0.68	0.00	4.52
**5384**	Past daily cigarette smoke	Fars	Lamard	Female	0.53	0.00	7.07
**5385**	Past daily cigarette smoke	Kohkiluye and Bouyer Ahmad	Landeh	Female	1.60	0.00	11.92

**5386**	Past daily cigarette smoke	Gilan	Langrud	Female	0.29	0.00	3.34
**5387**	Past daily cigarette smoke	Isfahan	Lanjan	Female	0.57	0.00	4.79
**5388**	Past daily cigarette smoke	Fars	Lar (Larestan)	Female	0.57	0.00	7.15
**5389**	Past daily cigarette smoke	Chaharmahal	Lordakan	Female	0.46	0.00	2.32
**5390**	Past daily cigarette smoke	Azarbayjan_West	Mahabad	Female	1.17	0.00	4.72
**5391**	Past daily cigarette smoke	Markazi	Mahalat	Female	1.38	0.00	3.69
**5392**	Past daily cigarette smoke	Mazandaran	Mahmudabad	Female	0.39	0.00	6.02
**5393**	Past daily cigarette smoke	Zanjan	Mahneshan	Female	1.21	0.00	5.87
**5394**	Past daily cigarette smoke	Khorasan_razavi	Mahvelat	Female	0.57	0.00	7.21
**5395**	Past daily cigarette smoke	Azarbayjan_West	Maku	Female	3.03	0.00	8.40
**5396**	Past daily cigarette smoke	Tehran	Malard	Female	0.64	0.00	5.71
**5397**	Past daily cigarette smoke	Hamedan	Malayer	Female	0.90	0.00	2.64
**5398**	Past daily cigarette smoke	Azararbayjan_East	Malekan	Female	0.80	0.00	3.16
**5399**	Past daily cigarette smoke	Ilam	Malekshahi	Female	1.86	0.00	10.47
**5400**	Past daily cigarette smoke	Fars	Mamasany	Female	0.61	0.00	7.06
**5401**	Past daily cigarette smoke	Khorasan_North	Maneh and Samalqan	Female	0.44	0.00	4.70
**5402**	Past daily cigarette smoke	Kerman	Manujan	Female	0.65	0.00	5.44
**5403**	Past daily cigarette smoke	Azararbayjan_East	Maragheh	Female	0.94	0.00	3.13
**5404**	Past daily cigarette smoke	Azararbayjan_East	Marand	Female	1.19	0.00	3.49
**5405**	Past daily cigarette smoke	Golestan	Maravehtapeh	Female	1.21	0.00	9.38
**5406**	Past daily cigarette smoke	Kordestan	Marivan	Female	2.50	0.00	7.05
**5407**	Past daily cigarette smoke	Fars	Marvdasht	Female	0.68	0.00	7.30
**5408**	Past daily cigarette smoke	Gilan	Masal	Female	0.72	0.00	4.86
**5409**	Past daily cigarette smoke	Khorasan_razavi	Mashhad	Female	0.86	0.00	5.49
**5410**	Past daily cigarette smoke	Khuzestan	Masjed Soleyman	Female	0.69	0.00	4.29
**5411**	Past daily cigarette smoke	Semnan	Mayamey	Female	0.41	0.00	4.55
**5412**	Past daily cigarette smoke	Semnan	Mehdishahr	Female	0.52	0.00	4.53
**5413**	Past daily cigarette smoke	Ilam	Mehran	Female	1.05	0.00	7.30
**5414**	Past daily cigarette smoke	Yazd	Mehriz	Female	1.55	0.00	10.73
**5415**	Past daily cigarette smoke	Ardebil	Meshkinshahr	Female	0.94	0.00	3.95
**5416**	Past daily cigarette smoke	Yazd	Meybod	Female	0.89	0.00	7.97
**5417**	Past daily cigarette smoke	Hormozgan	Minab	Female	0.25	0.00	3.80
**5418**	Past daily cigarette smoke	Golestan	Minudasht	Female	0.59	0.00	6.05
**5419**	Past daily cigarette smoke	Sistan and Balouchestan	Mirjaveh	Female	2.41	0.00	31.16
**5420**	Past daily cigarette smoke	Azarbayjan_West	Miyandoab	Female	1.15	0.00	4.43
**5421**	Past daily cigarette smoke	Mazandaran	Miyandorud	Female	0.46	0.00	9.03
**5422**	Past daily cigarette smoke	Azararbayjan_East	Miyaneh	Female	1.03	0.00	3.17
**5423**	Past daily cigarette smoke	Isfahan	Mobarakeh	Female	1.59	0.00	6.88
**5424**	Past daily cigarette smoke	Fars	Mohr	Female	1.02	0.00	10.41
**5425**	Past daily cigarette smoke	Hamedan	Nahavand	Female	0.82	0.00	2.59
**5426**	Past daily cigarette smoke	Isfahan	Najafabad	Female	1.75	0.00	7.00
**5427**	Past daily cigarette smoke	Ardebil	Namin	Female	0.87	0.00	5.65
**5428**	Past daily cigarette smoke	Azarbayjan_West	Naqadeh	Female	2.31	0.00	6.93
**5429**	Past daily cigarette smoke	Kerman	Narmashir	Female	0.76	0.00	5.72
**5430**	Past daily cigarette smoke	Isfahan	Natanz	Female	0.56	0.00	4.89
**5431**	Past daily cigarette smoke	Isfahan	Nayin	Female	0.96	0.00	6.80
**5432**	Past daily cigarette smoke	Alborz	Nazarabad	Female	1.13	0.00	4.37
**5433**	Past daily cigarette smoke	Ardebil	Neer	Female	1.04	0.00	5.27
**5434**	Past daily cigarette smoke	Khorasan_South	Nehbandan	Female	0.97	0.00	6.25
**5435**	Past daily cigarette smoke	Mazandaran	Neka	Female	0.24	0.00	5.97
**5436**	Past daily cigarette smoke	Fars	Neyriz	Female	2.23	0.00	10.04
**5437**	Past daily cigarette smoke	Khorasan_razavi	Neyshabur	Female	0.32	0.00	4.92
**5438**	Past daily cigarette smoke	Sistan and Balouchestan	Nikshahr	Female	0.64	0.00	18.30
**5439**	Past daily cigarette smoke	Sistan and Balouchestan	Nimruz	Female	1.77	0.00	27.94
**5440**	Past daily cigarette smoke	Mazandaran	Noshahr	Female	0.34	0.00	6.23
**5441**	Past daily cigarette smoke	Mazandaran	Nur	Female	0.33	0.00	5.77
**5442**	Past daily cigarette smoke	Khuzestan	Omidiyeh	Female	0.49	0.00	4.08
**5443**	Past daily cigarette smoke	Azarbayjan_West	Orumiyeh	Female	2.26	0.00	6.42
**5444**	Past daily cigarette smoke	Azarbayjan_West	Oshnaviyeh	Female	2.01	0.00	7.37
**5445**	Past daily cigarette smoke	Azararbayjan_East	Osku	Female	1.52	0.00	4.56
**5446**	Past daily cigarette smoke	Tehran	Pakdasht	Female	0.52	0.00	5.36
**5447**	Past daily cigarette smoke	Tehran	Pardis	Female	0.80	0.00	7.52
**5448**	Past daily cigarette smoke	Ardebil	Parsabad	Female	1.63	0.00	4.84
**5449**	Past daily cigarette smoke	Hormozgan	Parsian (Gavbandi)	Female	0.65	0.00	6.03
**5450**	Past daily cigarette smoke	Fars	Pasargad	Female	1.67	0.00	10.70
**5451**	Past daily cigarette smoke	Kermanshah	Paveh	Female	1.58	0.00	6.36
**5452**	Past daily cigarette smoke	Azarbayjan_West	Piranshahr	Female	1.92	0.00	6.97
**5453**	Past daily cigarette smoke	Tehran	Pishva	Female	0.46	0.00	5.37
**5454**	Past daily cigarette smoke	Azarbayjan_West	Poldasht	Female	3.01	0.00	8.11
**5455**	Past daily cigarette smoke	Lorestan	Poldokhtar	Female	0.20	0.00	2.44
**5456**	Past daily cigarette smoke	Mazandaran	Qaemshahr	Female	0.28	0.00	5.76
**5457**	Past daily cigarette smoke	Tehran	Qarchak	Female	0.98	0.00	8.02
**5458**	Past daily cigarette smoke	Sistan and Balouchestan	Qasr qand	Female	1.23	0.00	28.20
**5459**	Past daily cigarette smoke	Kermanshah	Qasr-e-Shirin	Female	1.40	0.00	6.67
**5460**	Past daily cigarette smoke	Khorasan_South	Qayenat	Female	2.17	0.00	8.29
**5461**	Past daily cigarette smoke	Qazvin	Qazvin	Female	1.44	0.00	5.79
**5462**	Past daily cigarette smoke	Hormozgan	Qeshm	Female	0.41	0.00	4.21
**5463**	Past daily cigarette smoke	Fars	Qirokarzin	Female	0.54	0.00	7.04
**5464**	Past daily cigarette smoke	Qom	Qom	Female	1.20	0.00	6.78
**5465**	Past daily cigarette smoke	Kordestan	Qorveh	Female	3.12	0.00	8.66
**5466**	Past daily cigarette smoke	Khorasan_razavi	Quchan	Female	0.88	0.00	5.63
**5467**	Past daily cigarette smoke	Kerman	Rabar	Female	1.48	0.00	5.92
**5468**	Past daily cigarette smoke	Kerman	Rafsanjan	Female	0.61	0.00	4.06
**5469**	Past daily cigarette smoke	Khuzestan	Ramhormoz	Female	0.35	0.00	2.96
**5470**	Past daily cigarette smoke	Mazandaran	Ramsar	Female	1.07	0.00	9.09
**5471**	Past daily cigarette smoke	Khuzestan	Ramshir	Female	0.73	0.00	4.28
**5472**	Past daily cigarette smoke	Golestan	Ramyan	Female	1.20	0.00	9.12
**5473**	Past daily cigarette smoke	Gilan	Rasht	Female	0.33	0.00	3.13
**5474**	Past daily cigarette smoke	Khorasan_razavi	Rashtkhar	Female	0.61	0.00	7.12
**5475**	Past daily cigarette smoke	Kermanshah	Ravansar	Female	1.53	0.00	6.28
**5476**	Past daily cigarette smoke	Kerman	Ravar	Female	0.56	0.00	3.99
**5477**	Past daily cigarette smoke	Khorasan_North	Raz va Jergolan	Female	0.82	0.00	6.99
**5478**	Past daily cigarette smoke	Hamedan	Razan	Female	1.05	0.00	2.82
**5479**	Past daily cigarette smoke	Tehran	Rey	Female	0.69	0.00	5.22
**5480**	Past daily cigarette smoke	Kerman	Reygan	Female	0.71	0.00	5.34
**5481**	Past daily cigarette smoke	Gilan	Rezvanshahr	Female	0.71	0.00	4.88
**5482**	Past daily cigarette smoke	Tehran	Robatkarim	Female	0.69	0.00	5.52
**5483**	Past daily cigarette smoke	Fars	Rostam	Female	0.52	0.00	7.31

**5484**	Past daily cigarette smoke	Kerman	Roudbar-e-Jonub	Female	0.35	0.00	3.83
**5485**	Past daily cigarette smoke	Hormozgan	Rudan	Female	0.31	0.00	3.86
**5486**	Past daily cigarette smoke	Gilan	Rudbar	Female	0.28	0.00	3.14
**5487**	Past daily cigarette smoke	Gilan	Rudsar	Female	0.96	0.00	4.34
**5488**	Past daily cigarette smoke	Lorestan	Rumshekan	Female	0.29	0.00	3.54
**5489**	Past daily cigarette smoke	Khorasan_razavi	Sabzevar	Female	0.29	0.00	4.89
**5490**	Past daily cigarette smoke	Yazd	Sadugh	Female	1.50	0.00	11.26
**5491**	Past daily cigarette smoke	Kermanshah	Sahneh	Female	1.45	0.00	6.01
**5492**	Past daily cigarette smoke	Kermanshah	Salas-e-Babajani	Female	1.48	0.00	6.14
**5493**	Past daily cigarette smoke	Azarbayjan_West	Salmas	Female	1.08	0.00	4.61
**5494**	Past daily cigarette smoke	Chaharmahal	Saman	Female	0.31	0.00	2.74
**5495**	Past daily cigarette smoke	Kordestan	Sanandaj	Female	2.77	0.00	7.18
**5496**	Past daily cigarette smoke	Kordestan	Saqqez	Female	3.64	0.00	9.06
**5497**	Past daily cigarette smoke	Kermanshah	Sar-e-Pol-e-Zohab	Female	0.81	0.00	4.24
**5498**	Past daily cigarette smoke	Azararbayjan_East	Sarab	Female	2.24	0.00	4.89
**5499**	Past daily cigarette smoke	Khorasan_razavi	Sarakhs	Female	0.45	0.00	7.15
**5500**	Past daily cigarette smoke	Sistan and Balouchestan	Saravan	Female	0.76	0.00	20.54
**5501**	Past daily cigarette smoke	Khorasan_South	Sarayan	Female	1.66	0.00	8.87
**5502**	Past daily cigarette smoke	Sistan and Balouchestan	Sarbaz	Female	1.10	0.00	18.65
**5503**	Past daily cigarette smoke	Khorasan_South	Sarbisheh	Female	2.35	0.00	9.69
**5504**	Past daily cigarette smoke	Azarbayjan_West	Sardasht	Female	1.27	0.00	4.85
**5505**	Past daily cigarette smoke	Ardebil	Sarein	Female	1.20	0.00	5.60
**5506**	Past daily cigarette smoke	Mazandaran	Sari	Female	0.25	0.00	5.46
**5507**	Past daily cigarette smoke	Kordestan	Sarvabad	Female	2.65	0.00	8.25
**5508**	Past daily cigarette smoke	Fars	Sarvestan	Female	0.95	0.00	10.12
**5509**	Past daily cigarette smoke	Mazandaran	Savadkuh	Female	0.56	0.00	8.05
**5510**	Past daily cigarette smoke	Mazandaran	Savadkuh_North	Female	0.54	0.00	7.84
**5511**	Past daily cigarette smoke	Markazi	Saveh	Female	1.04	0.00	2.98
**5512**	Past daily cigarette smoke	Alborz	Savojbolagh	Female	1.02	0.00	4.07
**5513**	Past daily cigarette smoke	Lorestan	Selseleh	Female	0.74	0.00	3.47
**5514**	Past daily cigarette smoke	Isfahan	Semirom	Female	0.47	0.00	4.80
**5515**	Past daily cigarette smoke	Isfahan	Semirom-e-Sofla	Female	0.98	0.00	7.23
**5516**	Past daily cigarette smoke	Semnan	Semnan	Female	0.93	0.00	4.44
**5517**	Past daily cigarette smoke	Fars	Sepidan	Female	0.54	0.00	7.35
**5518**	Past daily cigarette smoke	Azararbayjan_East	Shabestar	Female	1.08	0.00	3.27
**5519**	Past daily cigarette smoke	Khuzestan	Shadegan	Female	0.26	0.00	2.99
**5520**	Past daily cigarette smoke	Gilan	Shaft	Female	0.32	0.00	3.21
**5521**	Past daily cigarette smoke	Azarbayjan_West	Shahindezh	Female	6.49	2.39	10.44
**5522**	Past daily cigarette smoke	Tehran	Shahr-e Qods	Female	1.84	0.00	7.32
**5523**	Past daily cigarette smoke	Kerman	Shahr-e-Babak	Female	0.60	0.00	4.02
**5524**	Past daily cigarette smoke	Chaharmahal	Shahr-e-Kord	Female	0.16	0.00	1.82
**5525**	Past daily cigarette smoke	Isfahan	Shahreza	Female	1.00	0.00	5.41
**5526**	Past daily cigarette smoke	Tehran	Shahriyar	Female	1.20	0.00	6.31
**5527**	Past daily cigarette smoke	Semnan	Shahrud	Female	0.27	0.00	3.12
**5528**	Past daily cigarette smoke	Markazi	Shazand	Female	4.99	2.59	7.47
**5529**	Past daily cigarette smoke	Tehran	Shemiranat	Female	0.49	0.00	5.29
**5530**	Past daily cigarette smoke	Fars	Shiraz	Female	1.83	0.00	8.76
**5531**	Past daily cigarette smoke	Khorasan_North	Shirvan	Female	0.70	0.00	4.94
**5532**	Past daily cigarette smoke	Ilam	Shirvan and Chard-e-Aval	Female	1.23	0.00	7.51
**5533**	Past daily cigarette smoke	Azarbayjan_West	Showt	Female	2.49	0.00	6.70
**5534**	Past daily cigarette smoke	Khuzestan	Shush	Female	2.61	0.00	5.33
**5535**	Past daily cigarette smoke	Khuzestan	Shushtar	Female	1.10	0.00	4.27
**5536**	Past daily cigarette smoke	Gilan	Siahkal	Female	0.49	0.00	4.50
**5537**	Past daily cigarette smoke	Sistan and Balouchestan	Sib o Soran	Female	1.33	0.00	28.91
**5538**	Past daily cigarette smoke	Mazandaran	Simorgh	Female	0.48	0.00	8.41
**5539**	Past daily cigarette smoke	Hormozgan	Sirik	Female	0.35	0.00	6.07
**5540**	Past daily cigarette smoke	Kerman	Sirjan	Female	0.87	0.00	4.26
**5541**	Past daily cigarette smoke	Ilam	Sirvan	Female	1.84	0.00	10.06
**5542**	Past daily cigarette smoke	Zanjan	Soltaniyeh	Female	0.92	0.00	5.26
**5543**	Past daily cigarette smoke	Kermanshah	Sonqor	Female	1.88	0.00	6.10
**5544**	Past daily cigarette smoke	Semnan	Sorkheh	Female	0.50	0.00	4.38
**5545**	Past daily cigarette smoke	Gilan	Sume'eh Sara	Female	0.34	0.00	3.32
**5546**	Past daily cigarette smoke	Khorasan_South	Tabas	Female	0.99	0.00	6.22
**5547**	Past daily cigarette smoke	Azararbayjan_East	Tabriz	Female	1.82	0.00	4.21
**5548**	Past daily cigarette smoke	Markazi	Tafresh	Female	1.58	0.00	4.28
**5549**	Past daily cigarette smoke	Yazd	Taft	Female	2.43	0.00	11.65
**5550**	Past daily cigarette smoke	Azarbayjan_West	Takab	Female	1.29	0.00	4.89
**5551**	Past daily cigarette smoke	Qazvin	Takestan	Female	0.71	0.00	4.85
**5552**	Past daily cigarette smoke	Khorasan_razavi	Takht-e-Jolgeh (Firuzeh)	Female	0.47	0.00	7.40
**5553**	Past daily cigarette smoke	Alborz	Taleghan	Female	0.49	0.00	3.24
**5554**	Past daily cigarette smoke	Boushehr	Tangestan	Female	0.89	0.00	8.23
**5555**	Past daily cigarette smoke	Zanjan	Tarom	Female	0.56	0.00	3.77
**5556**	Past daily cigarette smoke	Gilan	Tavalesh	Female	0.55	0.00	3.58
**5557**	Past daily cigarette smoke	Khorasan_razavi	Taybad	Female	0.21	0.00	5.05
**5558**	Past daily cigarette smoke	Tehran	Tehran	Female	1.74	0.00	6.69
**5559**	Past daily cigarette smoke	Isfahan	Tiran and Karvan	Female	1.00	0.00	6.99
**5560**	Past daily cigarette smoke	Mazandaran	Tonekabon	Female	0.54	0.00	5.93
**5561**	Past daily cigarette smoke	Khorasan_razavi	Torbat-e-Heydariyeh	Female	1.05	0.00	6.03
**5562**	Past daily cigarette smoke	Khorasan_razavi	Torbat-e-Jam	Female	0.21	0.00	4.82
**5563**	Past daily cigarette smoke	Hamedan	Tuyserkan	Female	0.85	0.00	2.66
**5564**	Past daily cigarette smoke	Tehran	Varamin	Female	1.10	0.00	6.18
**5565**	Past daily cigarette smoke	Azararbayjan_East	Varzaqan	Female	2.52	0.00	5.67
**5566**	Past daily cigarette smoke	Yazd	Yazd	Female	1.11	0.00	8.13
**5567**	Past daily cigarette smoke	Sistan and Balouchestan	Zabol	Female	1.13	0.00	20.45
**5568**	Past daily cigarette smoke	Sistan and Balouchestan	Zaboli (Mehrestan)	Female	1.48	0.00	27.40
**5569**	Past daily cigarette smoke	Sistan and Balouchestan	Zahedan	Female	6.32	0.00	28.86
**5570**	Past daily cigarette smoke	Zanjan	Zanjan	Female	1.26	0.00	4.68
**5571**	Past daily cigarette smoke	Kerman	Zarand	Female	1.38	0.00	5.59
**5572**	Past daily cigarette smoke	Markazi	Zarandiyeh	Female	1.42	0.00	4.10
**5573**	Past daily cigarette smoke	Fars	Zarrindasht	Female	0.95	0.00	10.13
**5574**	Past daily cigarette smoke	Khorasan_razavi	Zave	Female	0.55	0.00	6.77
**5575**	Past daily cigarette smoke	Sistan and Balouchestan	Zehak	Female	2.23	0.00	30.53
**5576**	Past daily cigarette smoke	Khorasan_South	Zir kuh	Female	1.77	0.00	9.69
**5577**	Past daily cigarette smoke	Khuzestan	Abadan	Male	23.68	8.84	38.79
**5578**	Past daily cigarette smoke	Fars	Abadeh	Male	30.25	17.75	44.58
**5579**	Past daily cigarette smoke	Yazd	Abarkuh	Male	25.89	12.62	40.90
**5580**	Past daily cigarette smoke	Mazandaran	Abbas abad	Male	40.23	27.25	52.43
**5581**	Past daily cigarette smoke	Ilam	Abdanan	Male	14.66	4.44	24.47

**5582**	Past daily cigarette smoke	Zanjan	Abhar	Male	23.54	11.99	34.68
**5583**	Past daily cigarette smoke	Hormozgan	Abumusa	Male	20.89	3.62	37.83
**5584**	Past daily cigarette smoke	Qazvin	Abyek	Male	28.61	12.79	43.41
**5585**	Past daily cigarette smoke	Azararbayjan_East	Ahar	Male	26.55	13.96	39.34
**5586**	Past daily cigarette smoke	Khuzestan	Ahvaz	Male	23.87	13.33	35.18
**5587**	Past daily cigarette smoke	Azararbayjan_East	Ajabshir	Male	26.93	13.39	40.56
**5588**	Past daily cigarette smoke	Qazvin	Alborz	Male	31.42	15.93	47.28
**5589**	Past daily cigarette smoke	Golestan	Aliabad	Male	17.25	6.43	28.32
**5590**	Past daily cigarette smoke	Lorestan	Aligudarz	Male	24.93	11.13	38.27
**5591**	Past daily cigarette smoke	Gilan	Amlash	Male	26.76	11.68	41.77
**5592**	Past daily cigarette smoke	Mazandaran	Amol	Male	26.88	14.51	39.80
**5593**	Past daily cigarette smoke	Kerman	Anar	Male	26.53	10.54	42.90
**5594**	Past daily cigarette smoke	Kerman	Anbarabad	Male	20.72	6.78	33.77
**5595**	Past daily cigarette smoke	Khuzestan	Andika	Male	11.78	0.00	23.90
**5596**	Past daily cigarette smoke	Khuzestan	Andimeshk	Male	17.54	5.24	29.02
**5597**	Past daily cigarette smoke	Golestan	Aq Qala	Male	14.84	3.16	25.97
**5598**	Past daily cigarette smoke	Khuzestan	Aqajari	Male	21.94	6.45	38.26
**5599**	Past daily cigarette smoke	Semnan	Aradan	Male	23.49	8.43	38.83
**5600**	Past daily cigarette smoke	Markazi	Arak	Male	34.45	19.30	51.25
**5601**	Past daily cigarette smoke	Isfahan	Aran and Bidgol	Male	26.00	12.45	38.99
**5602**	Past daily cigarette smoke	Ardebil	Ardabil	Male	28.89	16.43	41.60
**5603**	Past daily cigarette smoke	Yazd	Ardakan	Male	21.13	8.68	32.29
**5604**	Past daily cigarette smoke	Chaharmahal	Ardal	Male	28.41	11.62	45.11
**5605**	Past daily cigarette smoke	Isfahan	Ardestan	Male	26.24	11.32	40.65
**5606**	Past daily cigarette smoke	Fars	Arsanjan	Male	29.95	16.09	46.39
**5607**	Past daily cigarette smoke	Kerman	Arzouyeh	Male	25.19	10.59	40.06
**5608**	Past daily cigarette smoke	Hamedan	Asadabad	Male	29.58	13.83	45.22
**5609**	Past daily cigarette smoke	Boushehr	Asaluyeh	Male	13.88	1.72	26.03
**5610**	Past daily cigarette smoke	Markazi	Ashtiyan	Male	32.09	13.64	49.97
**5611**	Past daily cigarette smoke	Gilan	Astaneh-ye-Ashrafiyeh	Male	28.06	14.24	42.52
**5612**	Past daily cigarette smoke	Gilan	Astara	Male	23.20	9.38	36.63
**5613**	Past daily cigarette smoke	Qazvin	Avaj	Male	31.44	13.88	48.76
**5614**	Past daily cigarette smoke	Golestan	Azadshahr	Male	14.69	2.48	26.49
**5615**	Past daily cigarette smoke	Azararbayjan_East	Azarshahr	Male	26.88	12.31	41.90
**5616**	Past daily cigarette smoke	Lorestan	Azna	Male	28.20	13.13	43.91
**5617**	Past daily cigarette smoke	Mazandaran	Babol	Male	23.75	11.40	35.97
**5618**	Past daily cigarette smoke	Mazandaran	Babolsar	Male	24.94	10.61	38.84
**5619**	Past daily cigarette smoke	Ilam	Badreh	Male	18.28	5.39	30.99
**5620**	Past daily cigarette smoke	Yazd	Bafq	Male	18.49	8.64	28.45
**5621**	Past daily cigarette smoke	Kerman	Baft	Male	25.41	12.18	39.16
**5622**	Past daily cigarette smoke	Khuzestan	Baghemalek	Male	20.73	7.71	32.78
**5623**	Past daily cigarette smoke	Yazd	Bahabad	Male	22.45	8.48	36.26
**5624**	Past daily cigarette smoke	Hamedan	Bahar	Male	34.09	19.50	50.41
**5625**	Past daily cigarette smoke	Tehran	Baharestan (Golestan)	Male	24.02	12.73	35.93
**5626**	Past daily cigarette smoke	Kohkiluye and Bouyer Ahmad	Bahmani	Male	18.30	5.18	30.34
**5627**	Past daily cigarette smoke	Khorasan_razavi	Bajestan	Male	18.67	4.83	32.22
**5628**	Past daily cigarette smoke	Khorasan_razavi	Bakhraz	Male	18.34	3.53	32.97
**5629**	Past daily cigarette smoke	Kerman	Bam	Male	23.97	10.15	37.90
**5630**	Past daily cigarette smoke	Hormozgan	Bandar-e-Abbas	Male	20.68	9.44	31.86
**5631**	Past daily cigarette smoke	Gilan	Bandar-e-Anzali	Male	26.72	12.80	40.73
**5632**	Past daily cigarette smoke	Golestan	Bandar-e-Gaz	Male	15.00	0.49	28.56
**5633**	Past daily cigarette smoke	Hormozgan	Bandar-e-Jask	Male	21.52	7.09	35.89
**5634**	Past daily cigarette smoke	Hormozgan	Bandar-e-Lengeh	Male	19.94	6.45	32.94
**5635**	Past daily cigarette smoke	Khuzestan	Bandar-e-Mahshahr	Male	23.54	10.47	37.39
**5636**	Past daily cigarette smoke	Golestan	Bandar-e-Torkaman	Male	12.56	1.23	23.00
**5637**	Past daily cigarette smoke	Kordestan	Baneh	Male	36.95	20.52	55.01
**5638**	Past daily cigarette smoke	Khorasan_razavi	Bardeskan	Male	19.21	5.02	33.89
**5639**	Past daily cigarette smoke	Kerman	Bardsir	Male	25.82	11.78	40.64
**5640**	Past daily cigarette smoke	Hormozgan	Bashagerd	Male	20.16	6.11	34.58
**5641**	Past daily cigarette smoke	Kohkiluye and Bouyer Ahmad	Basht	Male	16.18	5.22	27.58
**5642**	Past daily cigarette smoke	Hormozgan	Bastak	Male	20.55	6.18	35.08
**5643**	Past daily cigarette smoke	Khuzestan	Bavi	Male	23.26	10.86	35.07
**5644**	Past daily cigarette smoke	Khuzestan	Behbahan	Male	22.24	9.29	36.06
**5645**	Past daily cigarette smoke	Mazandaran	Behshahr	Male	19.54	4.74	33.32
**5646**	Past daily cigarette smoke	Kordestan	Bijar	Male	27.98	15.07	40.52
**5647**	Past daily cigarette smoke	Ardebil	Bilehsavar	Male	29.15	12.51	45.36
**5648**	Past daily cigarette smoke	Khorasan_razavi	Binaloud	Male	18.37	3.13	33.41
**5649**	Past daily cigarette smoke	Khorasan_South	Birjand	Male	12.54	2.57	22.42
**5650**	Past daily cigarette smoke	Khorasan_North	Bojnurd	Male	12.29	3.12	21.23
**5651**	Past daily cigarette smoke	Chaharmahal	Bon	Male	28.85	10.10	48.23
**5652**	Past daily cigarette smoke	Azararbayjan_East	Bonab	Male	28.32	15.95	41.70
**5653**	Past daily cigarette smoke	Isfahan	Borkhar	Male	26.04	10.96	40.56
**5654**	Past daily cigarette smoke	Isfahan	Borkhar and Meymeh	Male	28.22	14.72	41.65
**5655**	Past daily cigarette smoke	Chaharmahal	Borujen	Male	29.77	14.44	45.43
**5656**	Past daily cigarette smoke	Lorestan	Borujerd	Male	27.93	13.60	43.10
**5657**	Past daily cigarette smoke	Khorasan_South	Boshruyeh	Male	12.62	0.95	23.83
**5658**	Past daily cigarette smoke	Azararbayjan_East	Bostanabad	Male	27.44	14.64	40.82
**5659**	Past daily cigarette smoke	Fars	Bovanat	Male	20.66	7.17	33.46
**5660**	Past daily cigarette smoke	Kohkiluye and Bouyer Ahmad	Boyer Ahmad	Male	22.50	9.94	35.61
**5661**	Past daily cigarette smoke	Qazvin	Boyinzahra	Male	34.73	19.27	50.73
**5662**	Past daily cigarette smoke	Isfahan	Buein va Miandasht	Male	26.90	11.55	42.56
**5663**	Past daily cigarette smoke	Azarbayjan_West	Bukan	Male	36.22	20.20	52.91
**5664**	Past daily cigarette smoke	Boushehr	Bushehr	Male	15.01	3.27	26.40
**5665**	Past daily cigarette smoke	Isfahan	Chadegan	Male	27.57	13.18	42.60
**5666**	Past daily cigarette smoke	Sistan and Balouchestan	Chahbahar	Male	18.30	5.44	30.60
**5667**	Past daily cigarette smoke	Azarbayjan_West	Chaipareh	Male	38.69	20.38	57.15
**5668**	Past daily cigarette smoke	Azarbayjan_West	Chaldoran	Male	39.78	20.93	59.16
**5669**	Past daily cigarette smoke	Mazandaran	Chalus	Male	30.40	16.24	45.18
**5670**	Past daily cigarette smoke	Azararbayjan_East	Charoimaq	Male	25.90	12.74	38.78
**5671**	Past daily cigarette smoke	Khorasan_razavi	Chenaran	Male	19.43	4.75	34.93
**5672**	Past daily cigarette smoke	Kohkiluye and Bouyer Ahmad	Cheram	Male	23.40	13.32	33.20
**5673**	Past daily cigarette smoke	Kermanshah	Dalaho	Male	21.27	6.17	36.11
**5674**	Past daily cigarette smoke	Lorestan	Dalfan	Male	26.42	12.64	41.14
**5675**	Past daily cigarette smoke	Sistan and Balouchestan	Dalgan	Male	17.25	8.57	26.25
**5676**	Past daily cigarette smoke	Tehran	Damavand	Male	21.01	9.64	32.51
**5677**	Past daily cigarette smoke	Semnan	Damghan	Male	21.40	7.69	34.66
**5678**	Past daily cigarette smoke	Fars	Darab	Male	27.53	14.04	42.82
**5679**	Past daily cigarette smoke	Khorasan_South	Darmian	Male	12.55	1.18	23.84

**5680**	Past daily cigarette smoke	Khorasan_razavi	Darrehgaz	Male	18.49	5.27	31.85
**5681**	Past daily cigarette smoke	Ilam	Darrehshahr	Male	18.40	7.84	29.07
**5682**	Past daily cigarette smoke	Khuzestan	Dasht-e-Azadegan	Male	22.48	6.86	37.73
**5683**	Past daily cigarette smoke	Boushehr	Dashtestan	Male	15.22	4.67	25.49
**5684**	Past daily cigarette smoke	Boushehr	Dashti	Male	11.64	1.57	21.38
**5685**	Past daily cigarette smoke	Khorasan_razavi	Davarzan	Male	18.40	3.24	33.64
**5686**	Past daily cigarette smoke	Boushehr	Dayyer	Male	12.97	0.36	24.67
**5687**	Past daily cigarette smoke	Kordestan	Dehgolan	Male	30.11	15.69	44.97
**5688**	Past daily cigarette smoke	Ilam	Dehloran	Male	17.42	5.61	28.48
**5689**	Past daily cigarette smoke	Markazi	Delijan	Male	33.53	20.94	46.58
**5690**	Past daily cigarette smoke	Kohkiluye and Bouyer Ahmad	Dena	Male	22.20	8.45	35.93
**5691**	Past daily cigarette smoke	Boushehr	Deylam	Male	16.13	4.66	28.29
**5692**	Past daily cigarette smoke	Khuzestan	Dezful	Male	19.62	7.98	30.34
**5693**	Past daily cigarette smoke	Kordestan	Divandarreh	Male	37.38	22.68	54.16
**5694**	Past daily cigarette smoke	Lorestan	Dorud	Male	27.28	12.24	43.49
**5695**	Past daily cigarette smoke	Lorestan	Doureh	Male	24.86	11.10	38.40
**5696**	Past daily cigarette smoke	Fars	Eqlid	Male	26.35	11.96	41.51
**5697**	Past daily cigarette smoke	Khorasan_North	Esfarayen	Male	13.00	3.29	22.52
**5698**	Past daily cigarette smoke	Alborz	Eshtehard	Male	28.80	13.82	44.19
**5699**	Past daily cigarette smoke	Kermanshah	Eslamabad-e-Gharb	Male	19.43	6.64	31.91
**5700**	Past daily cigarette smoke	Tehran	Eslamshahr	Male	23.80	12.22	35.78
**5701**	Past daily cigarette smoke	Fars	Estahban	Male	25.69	9.91	41.89
**5702**	Past daily cigarette smoke	Ilam	Eyvan	Male	17.16	4.43	29.02
**5703**	Past daily cigarette smoke	Kerman	Fahraj	Male	24.60	10.44	39.19
**5704**	Past daily cigarette smoke	Isfahan	Falavarjan	Male	29.00	15.57	43.75
**5705**	Past daily cigarette smoke	Hamedan	Famenin	Male	30.87	14.31	47.82
**5706**	Past daily cigarette smoke	Markazi	Farahan	Male	32.37	13.79	50.24
**5707**	Past daily cigarette smoke	Fars	Farashband	Male	22.70	7.82	37.47
**5708**	Past daily cigarette smoke	Alborz	Fardis	Male	28.15	13.10	43.91
**5709**	Past daily cigarette smoke	Isfahan	Faridan	Male	28.69	14.75	43.85
**5710**	Past daily cigarette smoke	Khorasan_razavi	Fariman	Male	11.79	0.01	23.25
**5711**	Past daily cigarette smoke	Khorasan_North	Faroj	Male	11.46	2.76	20.10
**5712**	Past daily cigarette smoke	Chaharmahal	Farsan	Male	29.60	12.79	46.65
**5713**	Past daily cigarette smoke	Kerman	Faryab	Male	23.26	7.65	38.47
**5714**	Past daily cigarette smoke	Fars	Fasa	Male	27.26	13.19	42.44
**5715**	Past daily cigarette smoke	Khorasan_South	Ferdows	Male	10.54	0.34	20.16
**5716**	Past daily cigarette smoke	Mazandaran	Fereydunkenar	Male	23.94	9.71	38.00
**5717**	Past daily cigarette smoke	Isfahan	Fereydunshahr	Male	24.68	10.80	37.77
**5718**	Past daily cigarette smoke	Fars	Firozabad	Male	26.74	15.76	38.26
**5719**	Past daily cigarette smoke	Tehran	Firuzkuh	Male	18.59	7.07	29.57
**5720**	Past daily cigarette smoke	Sistan and Balouchestan	Fonuj	Male	18.69	4.90	32.55
**5721**	Past daily cigarette smoke	Gilan	Fuman	Male	25.12	12.58	37.19
**5722**	Past daily cigarette smoke	Kohkiluye and Bouyer Ahmad	Gachsaran	Male	20.99	7.77	34.04
**5723**	Past daily cigarette smoke	Golestan	Galikesh	Male	15.78	2.82	28.26
**5724**	Past daily cigarette smoke	Mazandaran	Galugah	Male	20.49	3.21	37.24
**5725**	Past daily cigarette smoke	Semnan	Garmsar	Male	23.14	10.01	36.56
**5726**	Past daily cigarette smoke	Boushehr	Genaveh	Male	13.88	3.08	24.32
**5727**	Past daily cigarette smoke	Fars	Gerash	Male	22.45	5.00	39.07
**5728**	Past daily cigarette smoke	Khorasan_North	Germeh	Male	14.46	2.05	27.07
**5729**	Past daily cigarette smoke	Ardebil	Germi	Male	31.85	17.48	46.73
**5730**	Past daily cigarette smoke	Kerman	Ghaleye-Ganj	Male	22.63	9.34	35.54
**5731**	Past daily cigarette smoke	Kermanshah	Gilan-e-Gharb	Male	21.38	10.28	32.47
**5732**	Past daily cigarette smoke	Isfahan	Golpayegan	Male	28.34	14.83	42.08
**5733**	Past daily cigarette smoke	Golestan	Gomishan	Male	14.61	0.26	28.25
**5734**	Past daily cigarette smoke	Khorasan_razavi	Gonabad	Male	16.96	3.38	30.42
**5735**	Past daily cigarette smoke	Golestan	Gonbad-e-Kavus	Male	16.32	4.52	28.22
**5736**	Past daily cigarette smoke	Golestan	Gorgan	Male	15.76	4.50	26.84
**5737**	Past daily cigarette smoke	Khuzestan	Guotvand	Male	21.24	7.37	35.18
**5738**	Past daily cigarette smoke	Khuzestan	Haftgol	Male	37.66	22.68	53.59
**5739**	Past daily cigarette smoke	Hormozgan	Hajiabad	Male	22.88	10.20	36.98
**5740**	Past daily cigarette smoke	Hamedan	Hamadan	Male	31.61	17.68	46.40
**5741**	Past daily cigarette smoke	Khuzestan	Hamidiyeh	Male	22.25	6.58	37.50
**5742**	Past daily cigarette smoke	Sistan and Balouchestan	Hamoon	Male	16.79	1.64	31.66
**5743**	Past daily cigarette smoke	Azararbayjan_East	Haris	Male	26.93	13.81	40.22
**5744**	Past daily cigarette smoke	Kermanshah	Harsin	Male	17.21	4.28	29.55
**5745**	Past daily cigarette smoke	Azararbayjan_East	Hashtrud	Male	27.24	14.00	40.78
**5746**	Past daily cigarette smoke	Khuzestan	Hendijan	Male	22.02	7.65	37.19
**5747**	Past daily cigarette smoke	Sistan and Balouchestan	Hirmand	Male	15.89	0.00	31.38
**5748**	Past daily cigarette smoke	Khuzestan	Hoveizeh	Male	22.60	6.67	38.50
**5749**	Past daily cigarette smoke	Zanjan	Ijerod	Male	27.60	15.59	40.21
**5750**	Past daily cigarette smoke	Ilam	Ilam	Male	18.77	6.85	30.13
**5751**	Past daily cigarette smoke	Sistan and Balouchestan	Iranshahr	Male	19.23	7.68	30.65
**5752**	Past daily cigarette smoke	Isfahan	Isfahan	Male	26.33	15.76	36.51
**5753**	Past daily cigarette smoke	Khuzestan	Izeh	Male	21.82	9.29	34.05
**5754**	Past daily cigarette smoke	Fars	Jahrom	Male	25.38	11.85	39.34
**5755**	Past daily cigarette smoke	Khorasan_North	Jajarm	Male	16.13	5.36	27.67
**5756**	Past daily cigarette smoke	Boushehr	Jam	Male	14.03	2.25	25.20
**5757**	Past daily cigarette smoke	Kermanshah	Javanrud	Male	23.61	7.34	39.03
**5758**	Past daily cigarette smoke	Kerman	Jiroft	Male	25.33	12.59	38.67
**5759**	Past daily cigarette smoke	Khorasan_razavi	Joghatai	Male	18.12	2.57	33.59
**5760**	Past daily cigarette smoke	Azararbayjan_East	Jolfa	Male	26.79	13.50	40.22
**5761**	Past daily cigarette smoke	Khorasan_razavi	Jowayin	Male	18.48	3.38	33.63
**5762**	Past daily cigarette smoke	Mazandaran	Juybar	Male	24.36	9.22	39.49
**5763**	Past daily cigarette smoke	Hamedan	Kabudarahang	Male	31.36	16.49	47.34
**5764**	Past daily cigarette smoke	Kerman	Kahnuj	Male	20.81	7.43	33.19
**5765**	Past daily cigarette smoke	Golestan	Kalaleh	Male	15.13	2.31	27.62
**5766**	Past daily cigarette smoke	Khorasan_razavi	Kalat	Male	17.94	1.28	34.22
**5767**	Past daily cigarette smoke	Azararbayjan_East	Kaleibar	Male	26.77	12.62	41.53
**5768**	Past daily cigarette smoke	Kordestan	Kamyaran	Male	29.51	13.06	45.82
**5769**	Past daily cigarette smoke	Boushehr	Kangan	Male	13.77	2.47	24.67
**5770**	Past daily cigarette smoke	Kermanshah	Kangavar	Male	31.42	19.16	45.61
**5771**	Past daily cigarette smoke	Alborz	Karaj	Male	27.95	17.14	38.68
**5772**	Past daily cigarette smoke	Khuzestan	Karun	Male	22.23	5.08	39.21
**5773**	Past daily cigarette smoke	Isfahan	Kashan	Male	27.57	14.07	40.94
**5774**	Past daily cigarette smoke	Khorasan_razavi	Kashmar	Male	14.95	3.26	26.26
**5775**	Past daily cigarette smoke	Fars	Kavar	Male	22.39	7.84	36.20
**5776**	Past daily cigarette smoke	Fars	Kazerun	Male	21.20	8.53	33.59
**5777**	Past daily cigarette smoke	Mazandaran	Kelardasht	Male	31.12	15.06	47.56

**5778**	Past daily cigarette smoke	Kerman	Kerman	Male	23.16	11.35	34.88
**5779**	Past daily cigarette smoke	Kermanshah	Kermanshah	Male	22.21	11.36	33.18
**5780**	Past daily cigarette smoke	Khorasan_razavi	Khaf	Male	10.65	0.00	22.07
**5781**	Past daily cigarette smoke	Khorasan_razavi	Khalilabad	Male	19.39	5.53	33.92
**5782**	Past daily cigarette smoke	Ardebil	Khalkhal	Male	29.31	15.94	43.09
**5783**	Past daily cigarette smoke	Hormozgan	Khamir	Male	27.91	15.57	41.84
**5784**	Past daily cigarette smoke	Isfahan	Khansar	Male	26.56	11.41	41.25
**5785**	Past daily cigarette smoke	Sistan and Balouchestan	Khash	Male	31.48	18.88	44.41
**5786**	Past daily cigarette smoke	Yazd	Khatam	Male	25.88	12.19	40.77
**5787**	Past daily cigarette smoke	Fars	Kherameh	Male	26.94	14.16	40.55
**5788**	Past daily cigarette smoke	Azararbayjan_East	Khodaafarin	Male	27.03	12.77	41.90
**5789**	Past daily cigarette smoke	Zanjan	Khodabandeh	Male	25.33	13.93	37.17
**5790**	Past daily cigarette smoke	Markazi	Khomeyn	Male	36.65	22.45	52.71
**5791**	Past daily cigarette smoke	Isfahan	Khomeynishahr	Male	28.91	15.58	43.62
**5792**	Past daily cigarette smoke	Markazi	Khondab	Male	28.61	11.78	43.94
**5793**	Past daily cigarette smoke	Fars	Khonj	Male	23.14	7.89	38.15
**5794**	Past daily cigarette smoke	Isfahan	Khoor va Biabanak	Male	24.10	9.03	39.14
**5795**	Past daily cigarette smoke	Lorestan	Khorramabad	Male	23.65	10.25	36.59
**5796**	Past daily cigarette smoke	Fars	Khorrambid	Male	26.94	12.17	42.65
**5797**	Past daily cigarette smoke	Zanjan	Khorramdarreh	Male	22.52	9.73	34.80
**5798**	Past daily cigarette smoke	Khuzestan	Khorramshahr	Male	24.55	9.57	40.57
**5799**	Past daily cigarette smoke	Khorasan_razavi	Khoshab	Male	17.84	2.64	32.56
**5800**	Past daily cigarette smoke	Azarbayjan_West	Khoy	Male	39.78	23.87	56.66
**5801**	Past daily cigarette smoke	Khorasan_South	Khusef	Male	14.14	2.16	26.07
**5802**	Past daily cigarette smoke	Chaharmahal	Kiaar	Male	29.88	13.90	47.36
**5803**	Past daily cigarette smoke	Kohkiluye and Bouyer Ahmad	Kohgiluyeh	Male	22.98	9.97	36.98
**5804**	Past daily cigarette smoke	Markazi	Komeijan	Male	32.43	14.65	49.96
**5805**	Past daily cigarette smoke	Sistan and Balouchestan	Konarak	Male	19.05	5.79	32.27
**5806**	Past daily cigarette smoke	Golestan	Kordkuy	Male	17.05	5.20	30.12
**5807**	Past daily cigarette smoke	Ardebil	Kowsar	Male	29.22	13.75	44.29
**5808**	Past daily cigarette smoke	Kerman	Kuhbonan	Male	22.94	7.46	37.56
**5809**	Past daily cigarette smoke	Lorestan	Kuhdasht	Male	25.32	10.77	39.88
**5810**	Past daily cigarette smoke	Chaharmahal	Kuhrang	Male	23.84	8.69	37.84
**5811**	Past daily cigarette smoke	Gilan	Lahijan	Male	29.33	17.50	41.92
**5812**	Past daily cigarette smoke	Khuzestan	Lali	Male	21.69	7.15	36.64
**5813**	Past daily cigarette smoke	Fars	Lamard	Male	14.13	1.54	26.63
**5814**	Past daily cigarette smoke	Kohkiluye and Bouyer Ahmad	Landeh	Male	21.82	4.74	39.15
**5815**	Past daily cigarette smoke	Gilan	Langrud	Male	25.65	12.44	38.67
**5816**	Past daily cigarette smoke	Isfahan	Lanjan	Male	28.73	15.15	42.73
**5817**	Past daily cigarette smoke	Fars	Lar (Larestan)	Male	24.53	10.74	39.04
**5818**	Past daily cigarette smoke	Chaharmahal	Lordakan	Male	29.60	16.16	44.73
**5819**	Past daily cigarette smoke	Azarbayjan_West	Mahabad	Male	36.05	19.60	52.40
**5820**	Past daily cigarette smoke	Markazi	Mahalat	Male	33.58	16.29	51.71
**5821**	Past daily cigarette smoke	Mazandaran	Mahmudabad	Male	24.64	11.34	37.60
**5822**	Past daily cigarette smoke	Zanjan	Mahneshan	Male	27.16	13.95	40.91
**5823**	Past daily cigarette smoke	Khorasan_razavi	Mahvelat	Male	17.74	2.95	32.61
**5824**	Past daily cigarette smoke	Azarbayjan_West	Maku	Male	40.44	21.92	59.70
**5825**	Past daily cigarette smoke	Tehran	Malard	Male	24.12	12.81	36.19
**5826**	Past daily cigarette smoke	Hamedan	Malayer	Male	30.20	15.46	44.86
**5827**	Past daily cigarette smoke	Azararbayjan_East	Malekan	Male	24.93	12.02	37.47
**5828**	Past daily cigarette smoke	Ilam	Malekshahi	Male	18.28	4.45	32.10
**5829**	Past daily cigarette smoke	Fars	Mamasany	Male	25.48	11.70	40.02
**5830**	Past daily cigarette smoke	Khorasan_North	Maneh and Samalqan	Male	14.82	4.26	25.46
**5831**	Past daily cigarette smoke	Kerman	Manujan	Male	22.78	7.65	38.21
**5832**	Past daily cigarette smoke	Azararbayjan_East	Maragheh	Male	29.15	18.27	41.61
**5833**	Past daily cigarette smoke	Azararbayjan_East	Marand	Male	26.00	12.85	38.54
**5834**	Past daily cigarette smoke	Golestan	Maravehtapeh	Male	16.14	1.55	30.02
**5835**	Past daily cigarette smoke	Kordestan	Marivan	Male	29.87	14.17	45.04
**5836**	Past daily cigarette smoke	Fars	Marvdasht	Male	24.50	11.47	36.92
**5837**	Past daily cigarette smoke	Gilan	Masal	Male	27.18	13.54	41.23
**5838**	Past daily cigarette smoke	Khorasan_razavi	Mashhad	Male	17.25	6.99	27.62
**5839**	Past daily cigarette smoke	Khuzestan	Masjed Soleyman	Male	22.04	8.62	35.54
**5840**	Past daily cigarette smoke	Semnan	Mayamey	Male	18.70	3.25	33.87
**5841**	Past daily cigarette smoke	Semnan	Mehdishahr	Male	22.14	8.06	35.81
**5842**	Past daily cigarette smoke	Ilam	Mehran	Male	17.93	8.69	27.01
**5843**	Past daily cigarette smoke	Yazd	Mehriz	Male	24.01	10.76	36.68
**5844**	Past daily cigarette smoke	Ardebil	Meshkinshahr	Male	30.27	16.55	44.83
**5845**	Past daily cigarette smoke	Yazd	Meybod	Male	28.84	15.38	43.85
**5846**	Past daily cigarette smoke	Hormozgan	Minab	Male	17.73	5.41	29.51
**5847**	Past daily cigarette smoke	Golestan	Minudasht	Male	13.97	2.06	25.18
**5848**	Past daily cigarette smoke	Sistan and Balouchestan	Mirjaveh	Male	20.76	5.95	36.51
**5849**	Past daily cigarette smoke	Azarbayjan_West	Miyandoab	Male	34.90	20.61	49.00
**5850**	Past daily cigarette smoke	Mazandaran	Miyandorud	Male	22.81	4.75	40.21
**5851**	Past daily cigarette smoke	Azararbayjan_East	Miyaneh	Male	25.08	13.04	36.75
**5852**	Past daily cigarette smoke	Isfahan	Mobarakeh	Male	28.96	15.28	44.18
**5853**	Past daily cigarette smoke	Fars	Mohr	Male	18.79	8.22	29.38
**5854**	Past daily cigarette smoke	Hamedan	Nahavand	Male	29.74	14.61	45.64
**5855**	Past daily cigarette smoke	Isfahan	Najafabad	Male	28.00	14.14	41.42
**5856**	Past daily cigarette smoke	Ardebil	Namin	Male	29.62	13.72	46.02
**5857**	Past daily cigarette smoke	Azarbayjan_West	Naqadeh	Male	37.28	21.29	54.17
**5858**	Past daily cigarette smoke	Kerman	Narmashir	Male	23.45	9.22	38.29
**5859**	Past daily cigarette smoke	Isfahan	Natanz	Male	26.76	12.17	41.31
**5860**	Past daily cigarette smoke	Isfahan	Nayin	Male	28.08	14.99	42.50
**5861**	Past daily cigarette smoke	Alborz	Nazarabad	Male	31.90	17.79	47.45
**5862**	Past daily cigarette smoke	Ardebil	Neer	Male	30.51	19.63	41.31
**5863**	Past daily cigarette smoke	Khorasan_South	Nehbandan	Male	9.88	0.33	19.32
**5864**	Past daily cigarette smoke	Mazandaran	Neka	Male	20.66	6.24	34.57
**5865**	Past daily cigarette smoke	Fars	Neyriz	Male	26.43	12.80	40.33
**5866**	Past daily cigarette smoke	Khorasan_razavi	Neyshabur	Male	14.79	3.90	25.41
**5867**	Past daily cigarette smoke	Sistan and Balouchestan	Nikshahr	Male	15.80	4.91	25.76
**5868**	Past daily cigarette smoke	Sistan and Balouchestan	Nimruz	Male	16.77	2.90	30.37
**5869**	Past daily cigarette smoke	Mazandaran	Noshahr	Male	28.88	12.20	45.67
**5870**	Past daily cigarette smoke	Mazandaran	Nur	Male	25.95	12.19	39.08
**5871**	Past daily cigarette smoke	Khuzestan	Omidiyeh	Male	23.02	9.55	36.80
**5872**	Past daily cigarette smoke	Azarbayjan_West	Orumiyeh	Male	36.65	22.28	50.77
**5873**	Past daily cigarette smoke	Azarbayjan_West	Oshnaviyeh	Male	38.25	20.61	56.32
**5874**	Past daily cigarette smoke	Azararbayjan_East	Osku	Male	26.15	13.54	38.68
**5875**	Past daily cigarette smoke	Tehran	Pakdasht	Male	21.58	10.33	32.67

**5876**	Past daily cigarette smoke	Tehran	Pardis	Male	21.59	7.50	35.00
**5877**	Past daily cigarette smoke	Ardebil	Parsabad	Male	29.82	14.50	45.52
**5878**	Past daily cigarette smoke	Hormozgan	Parsian (Gavbandi)	Male	18.88	7.62	30.33
**5879**	Past daily cigarette smoke	Fars	Pasargad	Male	17.62	5.99	29.77
**5880**	Past daily cigarette smoke	Kermanshah	Paveh	Male	24.59	9.26	40.27
**5881**	Past daily cigarette smoke	Azarbayjan_West	Piranshahr	Male	37.44	21.50	53.83
**5882**	Past daily cigarette smoke	Tehran	Pishva	Male	20.25	8.53	31.71
**5883**	Past daily cigarette smoke	Azarbayjan_West	Poldasht	Male	40.73	25.54	56.47
**5884**	Past daily cigarette smoke	Lorestan	Poldokhtar	Male	23.30	8.57	38.15
**5885**	Past daily cigarette smoke	Mazandaran	Qaemshahr	Male	25.17	11.51	39.09
**5886**	Past daily cigarette smoke	Tehran	Qarchak	Male	22.07	8.07	36.42
**5887**	Past daily cigarette smoke	Sistan and Balouchestan	Qasr qand	Male	18.69	4.39	32.58
**5888**	Past daily cigarette smoke	Kermanshah	Qasr-e-Shirin	Male	20.70	2.10	37.88
**5889**	Past daily cigarette smoke	Khorasan_South	Qayenat	Male	14.34	3.16	25.93
**5890**	Past daily cigarette smoke	Qazvin	Qazvin	Male	34.37	20.04	49.46
**5891**	Past daily cigarette smoke	Hormozgan	Qeshm	Male	20.68	7.15	33.99
**5892**	Past daily cigarette smoke	Fars	Qirokarzin	Male	26.60	15.93	37.13
**5893**	Past daily cigarette smoke	Qom	Qom	Male	24.16	9.99	38.25
**5894**	Past daily cigarette smoke	Kordestan	Qorveh	Male	28.03	13.25	41.95
**5895**	Past daily cigarette smoke	Khorasan_razavi	Quchan	Male	19.62	5.60	34.38
**5896**	Past daily cigarette smoke	Kerman	Rabar	Male	28.90	14.68	45.08
**5897**	Past daily cigarette smoke	Kerman	Rafsanjan	Male	25.53	12.46	39.07
**5898**	Past daily cigarette smoke	Khuzestan	Ramhormoz	Male	22.42	9.76	34.78
**5899**	Past daily cigarette smoke	Mazandaran	Ramsar	Male	29.61	14.98	44.31
**5900**	Past daily cigarette smoke	Khuzestan	Ramshir	Male	22.67	8.49	37.23
**5901**	Past daily cigarette smoke	Golestan	Ramyan	Male	16.00	3.64	28.68
**5902**	Past daily cigarette smoke	Gilan	Rasht	Male	25.45	14.72	35.72
**5903**	Past daily cigarette smoke	Khorasan_razavi	Rashtkhar	Male	16.14	3.37	28.46
**5904**	Past daily cigarette smoke	Kermanshah	Ravansar	Male	23.15	7.57	38.24
**5905**	Past daily cigarette smoke	Kerman	Ravar	Male	25.81	13.93	38.33
**5906**	Past daily cigarette smoke	Khorasan_North	Raz va Jergolan	Male	13.98	0.00	27.71
**5907**	Past daily cigarette smoke	Hamedan	Razan	Male	32.05	16.25	48.35
**5908**	Past daily cigarette smoke	Tehran	Rey	Male	22.82	11.32	34.76
**5909**	Past daily cigarette smoke	Kerman	Reygan	Male	23.75	9.63	38.41
**5910**	Past daily cigarette smoke	Gilan	Rezvanshahr	Male	28.20	15.52	41.91
**5911**	Past daily cigarette smoke	Tehran	Robatkarim	Male	20.04	9.07	30.71
**5912**	Past daily cigarette smoke	Fars	Rostam	Male	23.66	8.99	37.96
**5913**	Past daily cigarette smoke	Kerman	Roudbar-e-Jonub	Male	21.98	8.15	35.44
**5914**	Past daily cigarette smoke	Hormozgan	Rudan	Male	13.01	1.60	24.26
**5915**	Past daily cigarette smoke	Gilan	Rudbar	Male	26.67	13.43	39.50
**5916**	Past daily cigarette smoke	Gilan	Rudsar	Male	27.15	15.00	39.80
**5917**	Past daily cigarette smoke	Lorestan	Rumshekan	Male	23.92	6.88	40.78
**5918**	Past daily cigarette smoke	Khorasan_razavi	Sabzevar	Male	19.02	7.05	31.74
**5919**	Past daily cigarette smoke	Yazd	Sadugh	Male	40.42	24.67	57.85
**5920**	Past daily cigarette smoke	Kermanshah	Sahneh	Male	20.05	8.63	30.40
**5921**	Past daily cigarette smoke	Kermanshah	Salas-e-Babajani	Male	21.09	7.65	34.03
**5922**	Past daily cigarette smoke	Azarbayjan_West	Salmas	Male	40.86	24.28	59.67
**5923**	Past daily cigarette smoke	Chaharmahal	Saman	Male	29.42	12.03	47.83
**5924**	Past daily cigarette smoke	Kordestan	Sanandaj	Male	30.35	16.30	44.19
**5925**	Past daily cigarette smoke	Kordestan	Saqqez	Male	34.83	19.46	50.64
**5926**	Past daily cigarette smoke	Kermanshah	Sar-e-Pol-e-Zohab	Male	18.22	4.34	30.85
**5927**	Past daily cigarette smoke	Azararbayjan_East	Sarab	Male	25.95	13.59	38.30
**5928**	Past daily cigarette smoke	Khorasan_razavi	Sarakhs	Male	18.52	2.29	34.00
**5929**	Past daily cigarette smoke	Sistan and Balouchestan	Saravan	Male	23.99	11.19	38.37
**5930**	Past daily cigarette smoke	Khorasan_South	Sarayan	Male	13.49	1.50	25.61
**5931**	Past daily cigarette smoke	Sistan and Balouchestan	Sarbaz	Male	18.35	6.31	30.37
**5932**	Past daily cigarette smoke	Khorasan_South	Sarbisheh	Male	17.82	7.41	29.29
**5933**	Past daily cigarette smoke	Azarbayjan_West	Sardasht	Male	38.00	21.06	55.67
**5934**	Past daily cigarette smoke	Ardebil	Sarein	Male	29.64	14.21	45.36
**5935**	Past daily cigarette smoke	Mazandaran	Sari	Male	23.25	11.20	35.33
**5936**	Past daily cigarette smoke	Kordestan	Sarvabad	Male	30.02	11.95	47.52
**5937**	Past daily cigarette smoke	Fars	Sarvestan	Male	24.93	8.43	41.19
**5938**	Past daily cigarette smoke	Mazandaran	Savadkuh	Male	26.50	12.88	41.11
**5939**	Past daily cigarette smoke	Mazandaran	Savadkuh_North	Male	24.38	8.12	40.00
**5940**	Past daily cigarette smoke	Markazi	Saveh	Male	31.23	15.02	46.22
**5941**	Past daily cigarette smoke	Alborz	Savojbolagh	Male	30.36	16.43	45.40
**5942**	Past daily cigarette smoke	Lorestan	Selseleh	Male	26.02	10.54	41.53
**5943**	Past daily cigarette smoke	Isfahan	Semirom	Male	28.84	16.63	41.52
**5944**	Past daily cigarette smoke	Isfahan	Semirom-e-Sofla	Male	27.39	11.71	43.08
**5945**	Past daily cigarette smoke	Semnan	Semnan	Male	20.79	7.89	33.75
**5946**	Past daily cigarette smoke	Fars	Sepidan	Male	27.23	13.08	42.36
**5947**	Past daily cigarette smoke	Azararbayjan_East	Shabestar	Male	21.07	9.00	32.32
**5948**	Past daily cigarette smoke	Khuzestan	Shadegan	Male	17.95	5.62	29.73
**5949**	Past daily cigarette smoke	Gilan	Shaft	Male	27.13	16.00	38.71
**5950**	Past daily cigarette smoke	Azarbayjan_West	Shahindezh	Male	32.39	17.53	46.99
**5951**	Past daily cigarette smoke	Tehran	Shahr-e Qods	Male	22.45	10.83	34.25
**5952**	Past daily cigarette smoke	Kerman	Shahr-e-Babak	Male	25.26	10.69	39.66
**5953**	Past daily cigarette smoke	Chaharmahal	Shahr-e-Kord	Male	31.32	16.36	46.25
**5954**	Past daily cigarette smoke	Isfahan	Shahreza	Male	27.97	13.53	43.03
**5955**	Past daily cigarette smoke	Tehran	Shahriyar	Male	23.54	12.93	34.29
**5956**	Past daily cigarette smoke	Semnan	Shahrud	Male	20.80	8.33	34.01
**5957**	Past daily cigarette smoke	Markazi	Shazand	Male	35.14	19.54	52.04
**5958**	Past daily cigarette smoke	Tehran	Shemiranat	Male	20.26	7.88	31.69
**5959**	Past daily cigarette smoke	Fars	Shiraz	Male	24.81	13.33	36.59
**5960**	Past daily cigarette smoke	Khorasan_North	Shirvan	Male	12.79	2.07	23.28
**5961**	Past daily cigarette smoke	Ilam	Shirvan and Chard-e-Aval	Male	18.26	6.40	29.75
**5962**	Past daily cigarette smoke	Azarbayjan_West	Showt	Male	44.14	26.78	63.56
**5963**	Past daily cigarette smoke	Khuzestan	Shush	Male	21.59	9.39	33.86
**5964**	Past daily cigarette smoke	Khuzestan	Shushtar	Male	22.11	10.79	33.64
**5965**	Past daily cigarette smoke	Gilan	Siahkal	Male	27.50	14.27	40.93
**5966**	Past daily cigarette smoke	Sistan and Balouchestan	Sib o Soran	Male	17.78	5.45	29.23
**5967**	Past daily cigarette smoke	Mazandaran	Simorgh	Male	24.47	8.62	40.58
**5968**	Past daily cigarette smoke	Hormozgan	Sirik	Male	22.11	11.85	32.23
**5969**	Past daily cigarette smoke	Kerman	Sirjan	Male	24.61	11.82	37.65
**5970**	Past daily cigarette smoke	Ilam	Sirvan	Male	18.52	5.07	31.94
**5971**	Past daily cigarette smoke	Zanjan	Soltaniyeh	Male	26.12	12.10	40.10
**5972**	Past daily cigarette smoke	Kermanshah	Sonqor	Male	25.34	12.03	40.04
**5973**	Past daily cigarette smoke	Semnan	Sorkheh	Male	22.93	7.57	38.09

**5974**	Past daily cigarette smoke	Gilan	Sume'eh Sara	Male	29.06	17.92	40.96
**5975**	Past daily cigarette smoke	Khorasan_South	Tabas	Male	11.90	2.18	20.80
**5976**	Past daily cigarette smoke	Azararbayjan_East	Tabriz	Male	26.53	16.06	37.13
**5977**	Past daily cigarette smoke	Markazi	Tafresh	Male	28.74	11.58	45.03
**5978**	Past daily cigarette smoke	Yazd	Taft	Male	26.66	14.12	39.45
**5979**	Past daily cigarette smoke	Azarbayjan_West	Takab	Male	35.15	18.65	51.42
**5980**	Past daily cigarette smoke	Qazvin	Takestan	Male	32.44	15.73	49.61
**5981**	Past daily cigarette smoke	Khorasan_razavi	Takht-e-Jolgeh (Firuzeh)	Male	19.02	7.59	30.27
**5982**	Past daily cigarette smoke	Alborz	Taleghan	Male	32.40	18.49	47.49
**5983**	Past daily cigarette smoke	Boushehr	Tangestan	Male	14.54	1.36	27.11
**5984**	Past daily cigarette smoke	Zanjan	Tarom	Male	27.23	14.41	40.14
**5985**	Past daily cigarette smoke	Gilan	Tavalesh	Male	24.78	12.63	36.52
**5986**	Past daily cigarette smoke	Khorasan_razavi	Taybad	Male	19.42	4.68	35.12
**5987**	Past daily cigarette smoke	Tehran	Tehran	Male	20.69	12.08	29.51
**5988**	Past daily cigarette smoke	Isfahan	Tiran and Karvan	Male	27.83	13.54	42.51
**5989**	Past daily cigarette smoke	Mazandaran	Tonekabon	Male	30.84	16.43	45.45
**5990**	Past daily cigarette smoke	Khorasan_razavi	Torbat-e-Heydariyeh	Male	19.23	5.91	32.54
**5991**	Past daily cigarette smoke	Khorasan_razavi	Torbat-e-Jam	Male	16.66	5.07	28.17
**5992**	Past daily cigarette smoke	Hamedan	Tuyserkan	Male	27.05	13.07	40.56
**5993**	Past daily cigarette smoke	Tehran	Varamin	Male	22.94	12.25	34.23
**5994**	Past daily cigarette smoke	Azararbayjan_East	Varzaqan	Male	26.68	13.16	40.12
**5995**	Past daily cigarette smoke	Yazd	Yazd	Male	22.72	11.70	33.22
**5996**	Past daily cigarette smoke	Sistan and Balouchestan	Zabol	Male	11.59	0.16	22.64
**5997**	Past daily cigarette smoke	Sistan and Balouchestan	Zaboli (Mehrestan)	Male	18.90	10.47	27.64
**5998**	Past daily cigarette smoke	Sistan and Balouchestan	Zahedan	Male	20.35	9.93	31.48
**5999**	Past daily cigarette smoke	Zanjan	Zanjan	Male	27.71	16.75	39.33
**6000**	Past daily cigarette smoke	Kerman	Zarand	Male	22.11	8.30	35.02
**6001**	Past daily cigarette smoke	Markazi	Zarandiyeh	Male	35.11	18.30	52.36
**6002**	Past daily cigarette smoke	Fars	Zarrindasht	Male	25.62	9.35	41.73
**6003**	Past daily cigarette smoke	Khorasan_razavi	Zave	Male	26.93	13.96	42.04
**6004**	Past daily cigarette smoke	Sistan and Balouchestan	Zehak	Male	16.14	0.00	31.79
**6005**	Past daily cigarette smoke	Khorasan_South	Zir kuh	Male	13.14	0.50	25.75
