# Supplementary material for: Current Inequities in Smoking Prevalence on District Level in Iran: A Systematic Analysis on the STEPS Survey
Source: J Res Health Sci. 2021 Dec 28;22(1):e00540. doi: 10.34172/jrhs.2022.75 (PMC9315459; doi:10.34172/jrhs.2022.75)
Supplement: Supplementary file 3 — Decomposition of the gap in exposure to secondhand smoking at home between the first and fifth quintiles of wealth index among women. [file jrhs-22-e00540-s003.pdf]

Supplementary file 3: Decomposition of the gap in exposure to secondhand smoke at home between the first and fifth quintiles of wealth index among women

**Variables**	**Percent (95 % CI)**	***P*-value**
Prevalence among the wealthiest	27.4 (24.8, 29.9)	0.001
Prevalence among the least fortunate	31.1 (29.3, 32.9)	0.001
Differences (total gap)	3.7 (0.6, 6.8)	0.019
Due to endowments (explained)	4.7 (1.7, 7.7)	0.002
Years of schooling	1.2 (0.2, 2.2)	0.022
Urbanization	1.0 (-0.6, 2.7)	0.230
Government employment	0.7 (-0.4, 1.7)	0.223
Complementary insurance	1.8 (-.6, 4.2)	0.137
Due to coefficients (unexplained)	-1.0 (-5.1, 3.2)	0.654
Years of schooling	14.2 (-11.9, 40.3)	0.287
Urbanization	4.4 (-4.0,12.8)	0.306
Government employment	0.9 (-5.5, 7.3)	0.783
Complementary insurance	3.8 (-4.2, 11.9)	0.346
Constant	-24.3 (-46.8, -1.9)	0.034
