# Supplementary material for: Current Inequities in Smoking Prevalence on District Level in Iran: A Systematic Analysis on the STEPS Survey
Source: J Res Health Sci. 2021 Dec 28;22(1):e00540. doi: 10.34172/jrhs.2022.75 (PMC9315459; doi:10.34172/jrhs.2022.75)
Supplement: Supplementary file 4 — Decomposition of the gap in exposure to secondhand smoking between the first and fifth quintiles of education index among both men and women. [file jrhs-22-e00540-s004.pdf]

Supplementary file 4: Decomposition of the gap in exposure to secondhand smoke between the first and fifth quintiles of education-index among both men and women

**Variables**	**Percent (95 % CI)**	***P*-value**
Prevalence among the most educated	30.7 (29.0,32.3)	0.001
Prevalence among the least educated	23.9 (22.2,25.5)	0.001
Differences (total gap)	6.8 (4.5,9.1)	0.001
Due to endowments (explained)	3.1 (-0.1,6.2)	0.001
Wealth index	0.002 (-0.9,0.9)	0.996
Urbanization	-1.2 (-2.6,0.2)	0.092
Government employment	-0.1 (-0.5,0.3)	0.605
Complementary insurance	5.1 (3.2,7.0)	0.001
Due to coefficients (unexplained)	3.1 (-0.1,6.2)	0.056
Wealth index	9.9 (4.4,15.5)	0.001
Urbanization	9.2 (3.1,15.2)	0.003
Government employment	-2.6 (-6.2,1.0)	0.157
Complementary insurance	0.7 (-5.0,6.3)	0.811
Constant	-14.1 (-20.9,-7.3)	0.001
