# Supplementary material for: Current Inequities in Smoking Prevalence on District Level in Iran: A Systematic Analysis on the STEPS Survey
Source: J Res Health Sci. 2021 Dec 28;22(1):e00540. doi: 10.34172/jrhs.2022.75 (PMC9315459; doi:10.34172/jrhs.2022.75)
Supplement: Supplementary file 5 — Decomposition of the gap in current daily cigarette smoking between the first and fifth quintiles of urbanization index among both genders. [file jrhs-22-e00540-s005.pdf]

Supplementary File 5: Decomposition of the gap in current daily cigarette smoking between the first and fifth quintiles of urbanization index among both sexes

**Variables**	**Percent (95 % CI)**	***P*-value**
Prevalence among the most urbanized	8.5 (7.2, 9.8)	0.001
Prevalence among the least urbanized	10.6 (9.0, 12.1)	0.001
Differences (total gap)	-2.1 (-4.1, -0.2)	0.041
Due to endowments (explained)	-2.3 (-4.1, -0.5)	0.012
Years of schooling	1.2 (0.3, 2.1)	0.010
Wealth index	-3.0 (-4.0, -1.9)	0.001
Government employment	-0.9 (-2.4, 0.6)	0.236
Complementary insurance	0.3 (-0.6, 1.2)	0.518
Due to coefficients (unexplained)	0.3 (-1.7, 2.2)	0.798
Years of schooling	12.8 (-0.1, 25.7)	0.052
Wealth index	-1.8 (-5.3, 1.8)	0.321
Government employment	-3.7 (-6.4, -1.0)	0.008
Complementary insurance	1.6 (-2.8, 6.0)	0.483
Constant	-17.4 (-28.7, -6.1)	0.002
